# Anti-Inflammatory Effects of α-Galactosylceramide Analogs in Activated Microglia: Involvement of the p38 MAPK Signaling Pathway

**DOI:** 10.1371/journal.pone.0087030

**Published:** 2014-02-11

**Authors:** Yeon-Hui Jeong, Yongju Kim, Heebum Song, Young Sun Chung, Seung Bum Park, Hee-Sun Kim

**Affiliations:** 1 Department of Molecular Medicine and Global Top5 Research Program, Tissue Injury Defense Research Center, Ewha Womans University Medical School, Seoul, Republic of Korea; 2 Department of Chemistry, Seoul National University, Seoul, Republic of Korea; 3 Department of Counseling Psychology, Korea Soongsil Cyber University, Seoul, Republic of Korea; 4 Department of Biophysics and Chemical Biology/Bio-MAX Institute, Seoul National University, Seoul, Republic of Korea; University of Edinburgh, United Kingdom

## Abstract

Microglial activation plays a pivotal role in the development and progression of neurodegenerative diseases. Thus, anti-inflammatory agents that control microglial activation can serve as potential therapeutic agents for neurodegenerative diseases. Here, we designed and synthesized α-galactosylceramide (α-GalCer) analogs to exert anti-inflammatory effects in activated microglia. We performed biological evaluations of 25 α-GalCer analogs and observed an interesting preliminary structure-activity relationship in their inhibitory influence on NO release and TNF-α production in LPS-stimulated BV2 microglial cells. After identification of **4d** and **4e** as hit compounds, we further investigated the underlying mechanism of their anti-inflammatory effects using RT-PCR analysis. We confirmed that **4d** and **4e** regulate the expression of iNOS, COX-2, IL-1β, and IL-6 at the mRNA level and the expression of TNF-α at the post-transcriptional level. In addition, both **4d** and **4e** inhibited LPS-induced DNA binding activities of NF-κB and AP-1 and phosphorylation of p38 MAPK without affecting other MAP kinases. When we examined the anti-inflammatory effect of a p38 MAPK-specific inhibitor, SB203580, on microglial activation, we observed an identical inhibitory pattern as that of **4d** and **4e**, not only on NO and TNF-α production but also on the DNA binding activities of NF-κB and AP-1. Taken together, these results suggest that p38 MAPK plays an important role in the anti-inflammatory effects of **4d** and **4e** via the modulation of NF-κB and AP-1 activities.

## Introduction

As resident immune cells in the central nervous system, microglia move constantly across brain parenchyma and constitute an immune surveillance system. In the healthy brain, microglia interact and exchange molecular signals with surrounding neuronal and non-neuronal cells [1]. In addition, microglia are involved in the clearance of damaged neurons by phagocytosis and induce neuronal recovery. However, over-activation or persistent activation of microglia leads to neuronal death, which is associated with neurodegenerative diseases such as Parkinson's disease, Alzheimer's disease, and multiple sclerosis [2]–[4]. Recent studies report that systemic inflammation also plays a role in the progression of neurodegenerative diseases by inducing microglial activation [5]–[7]. Thus, the development of novel small molecules that can specifically modulate microglial activation has been proposed as one potential strategy for treating or preventing neurodegenerative diseases [8], [9].

α-Galactosylceramide (α-GalCer), a bioactive glycolipid derived from a marine sponge, has therapeutic potential for autoimmune diseases, cancer, and microbial infections [10], [11]. α-GalCer binds to CD1d on antigen-presenting cells (APCs), and the resulting α-GalCer-CD1d complex stimulates the semi-invariant T-cell receptor (TCR) of invariant natural killer T (iNKT) cells, leading to the production of signaling molecules called cytokines that initiate cellular communication. These cytokines subsequently activate other immune cells such as neutrophils, dendritic cells, and macrophages, thereby further modulating immune responses [12]–[14]. The ability of α-GalCer to control autoimmunity has been demonstrated in experimental models of type I diabetes, experimental allergic encephalomyelitis, arthritis, and systemic lupus erythematosus [15]–[18]. Polarization of immune response toward T helper 2 (T_H_2) cytokines has been suggested to play a crucial role in the protection and treatment of autoimmune disease [10], [16], [18].

Despite the immunomodulatory activities of α-GalCer in some pathological conditions, the role of α-GalCer in brain inflammation has not been examined. In the present study, we designed and synthesized 25 α-GalCer analogs in a systematic fashion and examined their effects in activated microglia. Based on a series of biological evaluations, we identified **4d** and **4e** as novel α-GalCer analogs that significantly inhibited the release of nitric oxide (NO) and the cellular production of tumor necrosis factor (TNF)-α in lipopolysaccharide (LPS)-stimulated microglial cells. An investigation of molecular mechanisms showed that the anti-inflammatory effects of these α-GalCer analogs might be caused by the specific modulation of p38 MAPK-NF-κB/AP-1 signaling pathways. Interestingly, we observed no inhibitory activity in microglia upon treatment with KRN7000, a representative α-GalCer, probably due to its simultaneous stimulation of both pro- and anti-inflammatory cytokines [19], [20]. We further confirmed that **4d** and **4e** inhibited LPS-induced the DNA binding activities of NF-κB and AP-1 and the phosphorylation of p38 MAPK without affecting other MAP kinases. Therefore, the specific inhibition of microglial activation by α-GalCer analogs such as **4d** and **4e** may potentially serve as a therapy for neurodegenerative diseases.

## Materials and Methods

### 1. Synthesis and characterization of α-GalCer analogs

All reactions for the synthesis of α-GalCer analogs were performed either in oven-dried glassware or a microwave vessel under dry argon atmosphere. Microwave reactions were performed using CEM Discovery Benchmate. Each product was purified by flash column chromatography on silica gel (230–400 mesh). ^1^H and ^13^C NMR spectra of all new compounds were obtained using a 500 MHz or 300 MHz NMR spectrophotometer. Mass analyses were performed using a LC/MS system with electron spray ionization (ESI) or atmospheric pressure chemical ionization (APCI). High resolution mass analyses were conducted using a mass spectrometer with fast atomic bombardment (FAB) ionization via direct injection at the Mass Spectrometry Laboratory of Seoul National University (see Supporting Information S1).

### 2. Reagents

Toluene and tetrahydrofuran (THF) were dried by distillation from sodium-benzophenone immediately prior to use. Dicholormethane (DCM) was dried by distillation from CaH_2_. Other solvents and organic reagents were purchased from commercial venders and used without further purification unless otherwise mentioned. All reagents used for cell culture were purchased from Gibco BRL (Grand Island, NY, USA). LPS was obtained from Sigma-Aldrich (St. Louis, MO, USA). All reagents and enzymes for RT-PCR were purchased from Promega (Madison, WI, USA). Antibodies against phospho-/total forms of MAP kinases were purchased from Cell Signaling Technology (Beverley, MA, USA). Synthesized α-GalCer analogs were dissolved in dimethyl sulfoxide (DMSO) and added to cells at less than 0.3% (v/v) final concentration to avoid effects on cell viability.

### 3. Microglial cell culture and cell viability test

Immortalized murine BV2 microglial cells [21] were grown and maintained in Dulbecco's Modified Eagle Medium (DMEM) supplemented with 10% heat-inactivated fetal bovine serum (FBS), streptomycin (10 µg/ml), and penicillin (10 U/ml) at 37°C. In general, BV2 cells were stimulated with LPS (100 ng/ml) under DMEM containing 10% FBS and antibiotics, and α-GalCer analogs were added 1 h prior to LPS treatment. Cell viability was determined by MTT reduction assay as previously described [22].

### 4. Measurement of nitric oxide (NO), TNF-α, and intracellular reactive oxygen species (ROS) levels

Microglial cells (1×10^5^ cells per well in a 24-well plate) were pre-treated with α-GalCer analogs for 1 h and stimulated with LPS (100 ng/ml) for 16 h. The supernatants of the cultured microglia were then collected, and accumulated nitrite was measured using Griess reagent (Promega). The concentration of TNF-α in the supernatants was measured by ELISA using procedures recommended by the supplier (BD Biosciences, San Jose, CA, USA). Intracellular accumulation of ROS was measured with H_2_DCF-DA (Sigma-Aldrich) by modifying previously reported methods [23]. In brief, microglial cells were stimulated with LPS for 16 h and stained with 20 µM H_2_DCF-DA in HBSS buffer for 1 h at 37°C. DCF fluorescence intensity was measured at 485-nm excitation and 535-nm emission using a fluorescence plate reader (Molecular Devices, CA).

### 5. Real-time PCR analysis

BV2 cells (7.5×10^5^ cells in a 6-well plate) were treated with LPS for 6 h in the presence or absence of **4d** or **4e**, and total RNA was extracted with TRI reagent (Invitrogen Corporation, Carlsbad, CA, USA). Total RNA (1 µg) was reverse-transcribed in a reaction mixture containing 1 U RNase inhibitor, 500 ng random primers, 3 mM MgCl_2_, 0.5 mM dNTP, and 10 U reverse transcriptase (Promega). Aliquots of diluted cDNA (1∶10) were amplified with SYBR® Green PCR Master Mix (Applied Biosystems, Foster City, CA, USA) in a final volume of 20 µl. PCR was performed in an ABI Prism 7000 sequence detector (Applied Biosystems). PCR cycles consisted of initial denaturation at 95°C for 5 min, followed by 40 cycles of 95°C for 30 s, 60°C for 30 s, and 72°C for 45 s. Cycling threshold (Ct) values of genes were normalized to the Ct values of GAPDH, and the relative expression level was calculated with the 2^(Ct test gene – Ct GAPDH)^. Primers used for real-time PCR reactions are specified in Table 1.

**Table 1:** DNA sequences of primers used in real-time PCR reactions, and expected product sizes.

	Forward Primer (5′→3′)	Reverse Primer (5′→3′)	Size
iNOS	CAGCTGGGCTGTACAAACCTT	CATTGGAAGTGAAGCGTTTCG	95 bp
TNF-α	CATCTTCTCAAAATTCGAGTGACAA	TGGGAGTAGACAAGGTACAACCC	175 bp
COX-2	CCTGCTGCCCGACACCTTCA	AGCAACCCGGCCAGCAATCT	139 bp
IL-1β	CAACCAACAAGTGATATTCTCCATG	GATCCACACTCTCCAGCTGCA	152 bp
IL-6	ACAACCACGGCCTTCCCTACTT	CACGATTTCCCAGAGAACATGTG	129 bp
GAPDH	TTCACCACCATGGAGAAGGC	GGCATGGACTGTGGTCATGA	236 bp

### 6. Electrophoretic mobility shift assay (EMSA)

Nuclear extracts from treated microglia were prepared as previously described [24]. DNA oligomers containing consensus sequences of NF-κB or AP-1 were end-labeled using T4 polynucleotide kinase (New England Biolabs, Beverly, MA, USA) in the presence of [γ-^32^P] ATP. Nuclear proteins (5 µg) were incubated with ^32^P-labled probes on ice for 30 min, resolved on a 5% polyacrylamide gel, and visualized by autoradiography.

### 7. Western blot analysis

Cells were appropriately treated, and total cell lysates were prepared as previously described [24]. Proteins (20–100 µg) were heated with 4× SDS sample buffer, separated by SDS-PAGE gel electrophoresis, and transferred to nitrocellulose membranes. After blocking, membranes were incubated with primary antibodies (1∶1000) followed by horseradish peroxidase-conjugated secondary antibodies (1∶2000 dilution in TBST; Amersham, Piscataway, NJ, USA). The resulting blots were developed using an enhanced chemiluminescence detection kit (Amersham).

### 8. Statistical analysis

Unless otherwise stated, all experiments were performed with triplicate samples and repeated at least three times. Data are presented as mean ± S.E.M., and statistical comparisons between groups were performed using Mann-Whitney U tests. A *P* value<0.05 was considered statistically significant.

## Results

### 1. Synthesis of α-galactosylceramide analogs

**Figure 1:**
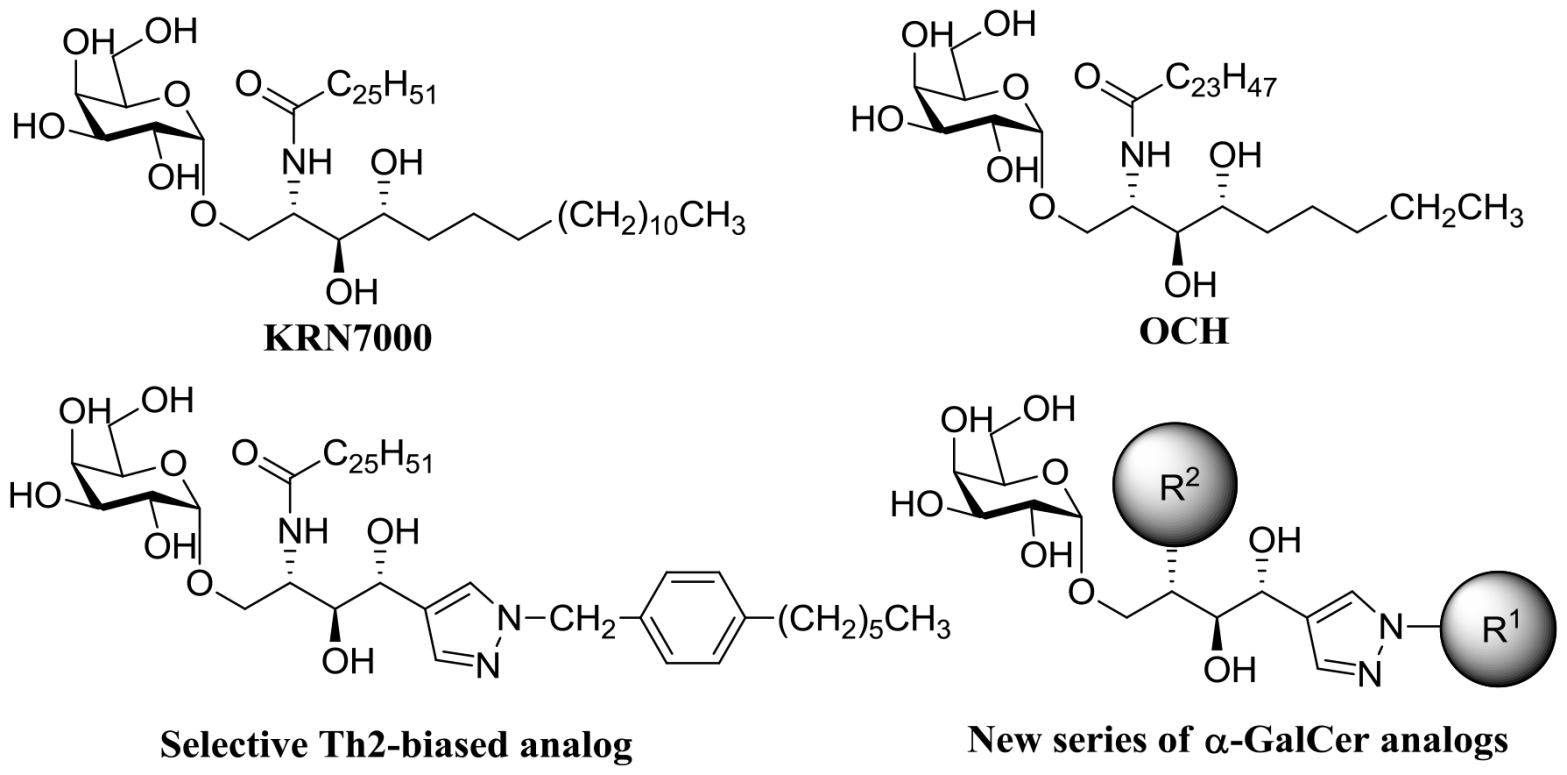
Chemical structures of bioactive α-GalCer derivatives and newly designed α-GalCer analogs.

Although KRN7000, a representative glycolipid of the α-GalCer family, is an excellent immunostimulatory compound, it has limited *in vivo* efficacy due to dual agonist effects on T_H_1 and T_H_2 cytokines. Therefore, we studied the systematic structural modification of α-GalCer to selectively polarize either the T_H_1 or T_H_2 immune response. For example, we recently described a series of α-GalCer analogs containing heterocyclic and phenyl moieties in the sphingosine backbone and found that treatment with a T_H_2-biased α-GalCer analog selectively stimulates the secretion of anti-inflammatory cytokines in iNKT cells (Figure 1) [25], [26]. With this selective T_H_2-biased α-GalCer analog in hand, we envisioned the development of new α-GalCer-based anti-inflammatory agents that control microglial activation as a potential therapy for neurodegenerative diseases. Therefore, we designed a new series of anti-inflammatory α-GalCer analogs with systematic changes in acyl chain and sphingosine backbone regions.

**Figure 2:**
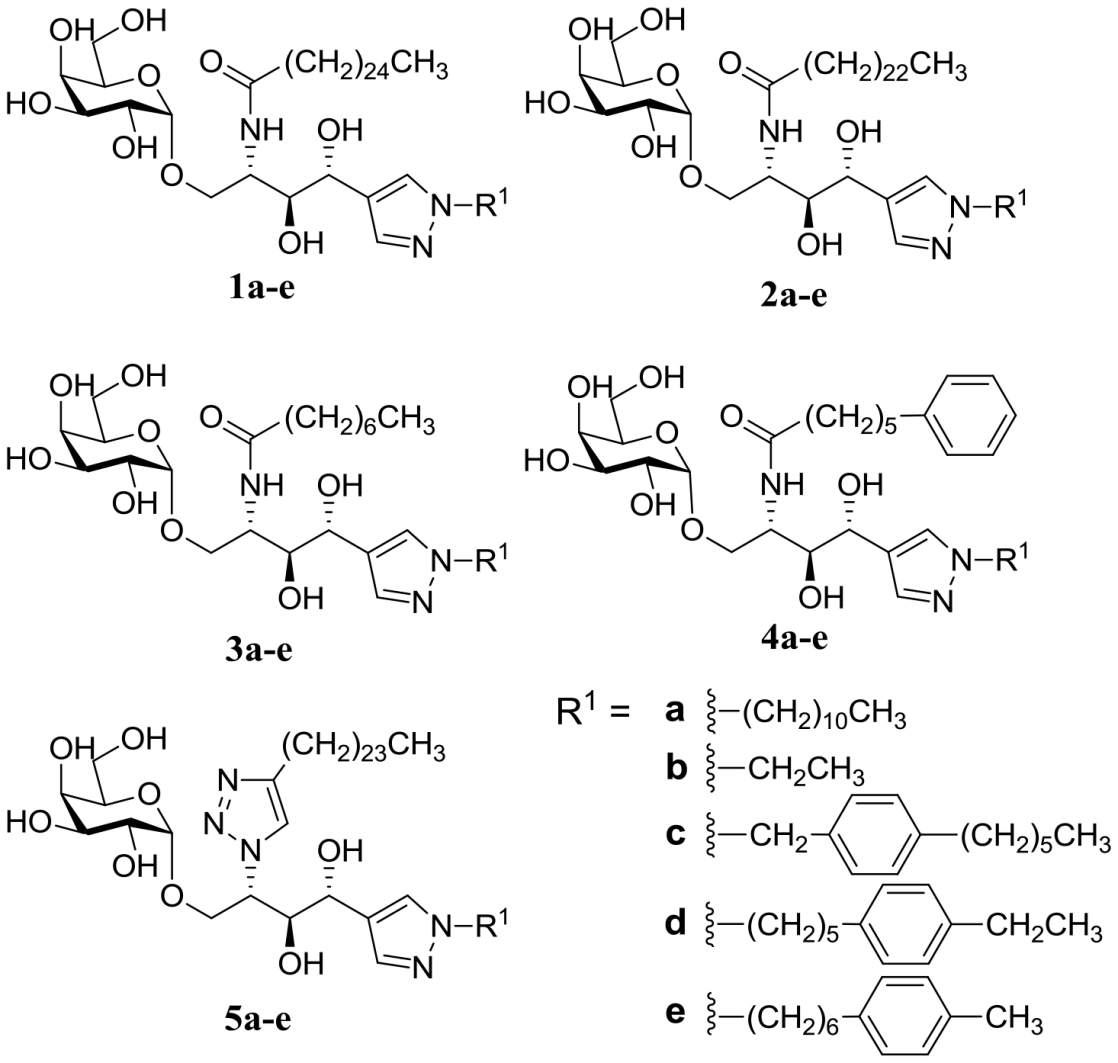
Chemical structures of newly designed α-GalCer analogs.

As shown in Figure 2, modifications in the acyl chain of α-GalCer contained four different carboxylic acids, including C_25_H_51_CO_2_H (**1**, from KRN7000), C_23_H_47_CO_2_H (**2**, from OCH, a known selective T_H_2 stimulator), octanoic acid (**3**, from T_H_2 selective glycolipid) [27], and 6-phenylhexanoic acid (**4**, from fatty acyl chain analogs with terminal phenyl group) [28]. Analogs containing 1,2,3-triazole, an isostere of amide bonds, had an identical acyl chain length as KRN7000 (**5**). In consideration of the biological activities of KRN7000, a representative immunomodulatory compound, we maintained the length of the sphingosine backbone of newly designed α-GalCer analogs as a 14-carbon-equivalent chain (**a**, **c**–**e**), similar to that of KRN7000. We also designed a different type of analog containing a 5-carbon-equivalent chain (**b**), which is similar to the sphigosine backbone of OCH.

**Figure 3:**
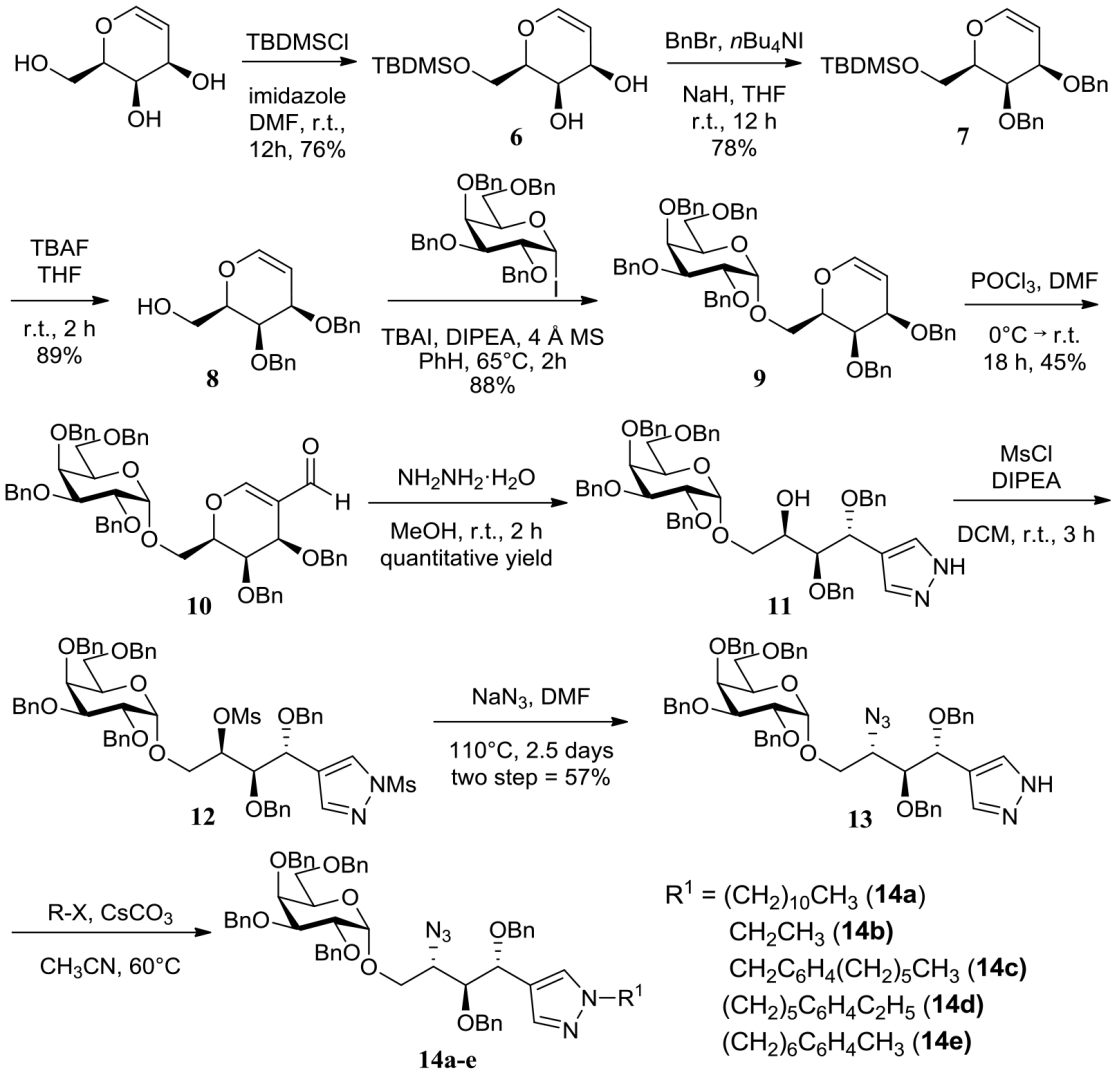
Synthetic scheme of key intermediates with late-stage diversification of the sphingosine backbone.

Unlike our previous report [25], we aimed to introduce various alkyl moieties in the sphingosine backbone at the late stage of synthesis via simple *N-*alkylation of pyrazole. The key intermediate **13** can be prepared from commercially available d-galactal in eight steps with a 12% overall yield (Figure 3). Diversification of the sphingosine backbone of key intermediate **13** was done through a substitution reaction of pyrazole nitrogen with five different kinds of alkyl halides in the presence of CsCO_3_. For example, *N-*alkylation of pyrazole on **13** with 1-bromoundecane or 1-bromoethane yielded compounds **14a** or **14b**, whose structure was designed to keep backbone length similar to KRN7000 or OCH, respectively. *N-*alkylation of **13** with 4-*n*-hexylbenzylbromide led to compound **14c** containing a phenyl group as well as pyrazole in its sphingosine backbone, originated from our T_H_2-biased α-GalCer analog [25]. The subsequent migration of the phenyl group in the sphingosine backbone yielded compounds **14d** and **14e**.

**Figure 4:**
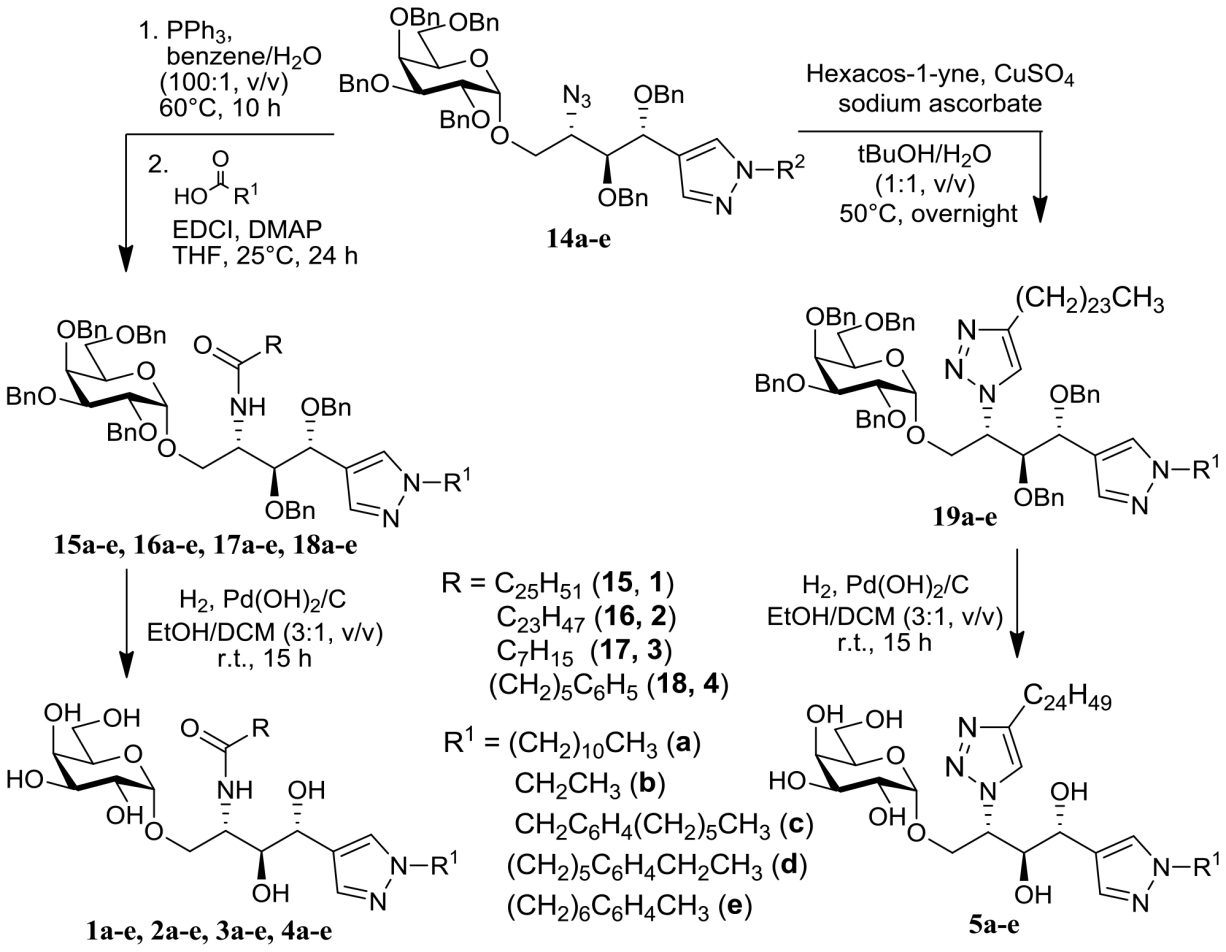
Synthetic scheme of final modifications of the acyl chain resulting in 25 unique α-GalCer analogs.

After systematic introduction of five unique sphingosine backbones in **14a**–**e**, we further diversified their acyl chains via a coupling reaction with various carboxylic acids after Staudinger reduction of the azide group or 1,3-dipolar cycloaddition of an azide group with a terminal alkyne (Figure 4). The EDCI-mediated amide coupling with either cerotic acid or lignoceric acid followed by global deprotection of six benzyl groups via catalytic hydrogenation in the presence of Pd(OH)_2_/C allowed the preparation of **1a**–**e** or **2a**–**e** as α-GalCer analogs with acyl chains of KRN7000 or OCH, respectively. Compounds **3a**–**e** were prepared by amide coupling with octanoic acid as α-GalCer analogs containing a short eight-carbon acyl chain. For compounds **4a–e**, the acyl chain region contains a 10-carbon-equivalent acyl chain with a terminal phenyl moiety for additional non-covalent interactions. As an isostere of amide bonds, triazole moiety was introduced via copper-mediated click reaction of the azide group in **14a**–**e** with hexacos-1-yne at the acyl chain position to yield compounds **5a–e**. Compound **1c** is our reported T_H_2-selective α-GalCer analog [25], and the systematic diversification of this compound provided a collection of 25 α-GalCer analogs (**1a**–**5e**) as a focused library to identify novel anti-inflammatory agents for the treatment of neurodegenerative diseases.

### 2. Effect of α-GalCer analogs on NO, ROS, and TNF-α production in LPS-stimulated microglia

**Figure 5:**
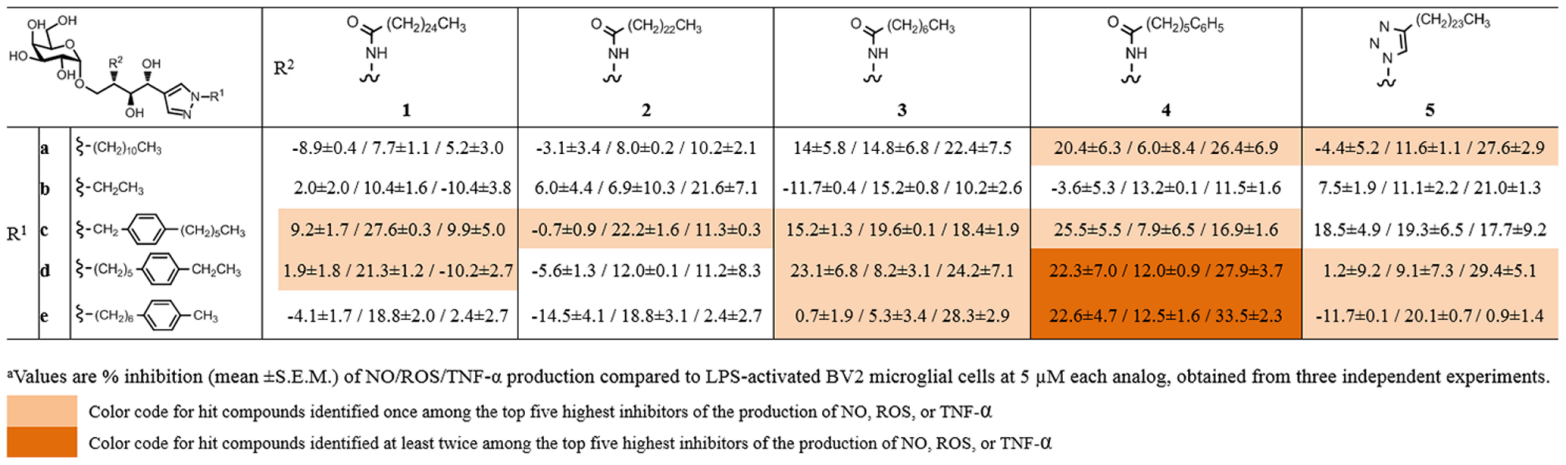
Inhibitory effect of α-GalCer analogs on NO/ROS/TNF-α production in LPS-stimulated microglial cells.^a^

To investigate the anti-inflammatory effects of systematically designed α-GalCer analogs in microglial activation, BV2 cells were treated with compounds 1 h prior to stimulation with LPS, and the inhibitory effects of each α-GalCer analog on LPS-induced production of NO, ROS, and TNF-α were examined. As shown in Figure 5, we measured the percent inhibition of NO, ROS, and TNF-α production upon treatment with 25 α-GalCer analogs at 5 µM concentration. It is worth mentioning that known α-GalCer that are known to be bioactive in iNKT cells showed no or marginal inhibitory effects on LPS-stimulated microglia. For example, KRN7000, a representative α-GalCer, showed an inhibitory effect only on ROS production (24.4% inhibition) but no effect on the production of NO (3.9% inhibition) or TNF-α (1.5% inhibition). In the case of OCH, a known immunomodulatory α-GalCer in iNKT cells, we observed increased production of NO (15.1%) and only slight inhibitory effects on the production of ROS (12.5% inhibition) and TNF-α (13.6% inhibition). When we tested our T_H_2-selective α-GalCer analog **1c** [25], there were marginal inhibitory activities in LPS-induced microglial cells.

**Figure 6:**
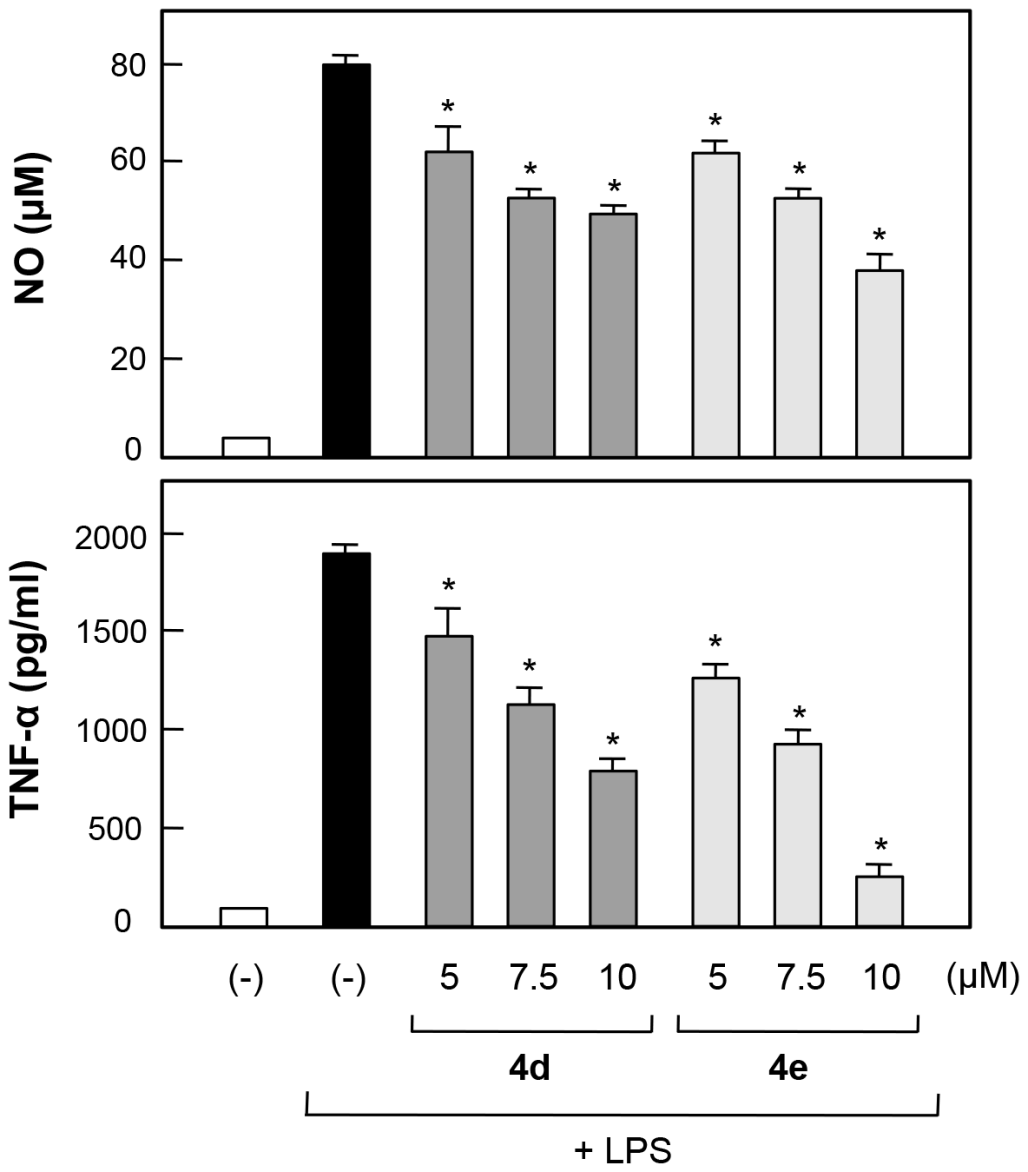
Dose-dependent inhibition of NO and TNF-α production by α-GalCer analogs 4d and 4e in LPS-stimulated BV2 microglial cells. Cells were incubated for 16 h with LPS in the absence or presence of α-GalCer analogs **4d** and **4e** (5–10 µM), and the amounts of released NO and TNF-α were measured in supernatants. Treatment with α-GalCer analogs alone did not affect NO or TNF-α production. Bars indicate the mean ± S.E.M. of three independent experiments. *P<0.05; significantly different from LPS-treated microglial cells.

To identify the best candidate for further *in vitro* study, we analyzed individual inhibition activities via sorting the most effective inhibitors in three independent assays. Anti-inflammatory compounds identified more than twice among the top five inhibitors of NO, ROS, or TNF-α production are highlighted in Figure 5 as dark orange, and compounds identified once among the top five inhibitors are highlighted in pale orange. This color code-based analysis revealed somewhat interesting patterns in structure-activity relationships of α-GalCer analogs in their inhibitory activity on NO, ROS, and TNF-α production. For instance, *N-*modifications of the pyrazole moiety with phenyl-containing alkyl chains in the sphingosine backbone (**c**, **d**, and **e**) showed general anti-inflammatory activity. In addition, α-GalCer analogs containing relatively short acyl chains (**3** and **4**) showed significant inhibitory activity on NO, ROS, and TNF-α release in LPS-induced BV2 microglial cells. In the case of α-GalCer analogs (**5**) containing triazole—an isostere of amide bonds—in acyl chains, we observed slight inhibitory activity on cellular production of NO, ROS, and TNF-α. Among the 25 α-GalCer analogs, **4d** and **4e**, which have relatively short acyl chains containing terminal phenyl rings and sphingosine backbones containing pyrazole and phenyl rings, showed the best efficacy in terms of percent inhibition of NO, ROS, and TNF-α production in LPS-induced microglia. We also confirmed the dose-dependent inhibition of **4d** and **4e** on NO and TNF-α production (Figure 6) and determined IC_50_ values; in the case of **4d**, IC_50_s for NO and TNF-α release were 13.8 and 9.1 µM, respectively, and in case of **4e**, IC_50_s for NO and TNF-α release were 10.2 and 6.3 µM. To exclude the possibility that the decrease in NO and cytokine levels was simply due to cell death, we assessed cell viability at various concentrations of **4d** and **4e**. MTT assay showed that **4d** and **4e** were not cytotoxic at concentrations up to 10 µM (data not shown). Furthermore, DMSO (up to 0.3%) did not affect NO, TNF-α, ROS production in LPS-stimulated BV2 cells, which confirmed that the cellular inhibitory activity is caused by the treatment of **4d** and **4e**, not due to DMSO (data not shown). Therefore, we further investigated the anti-inflammatory mechanism of **4d** and **4e** in activated microglia.

### 3. Compounds 4d and 4e suppressed mRNA expression of iNOS, COX-2, and pro-inflammatory cytokines in LPS-stimulated BV2 microglia

**Figure 7:**
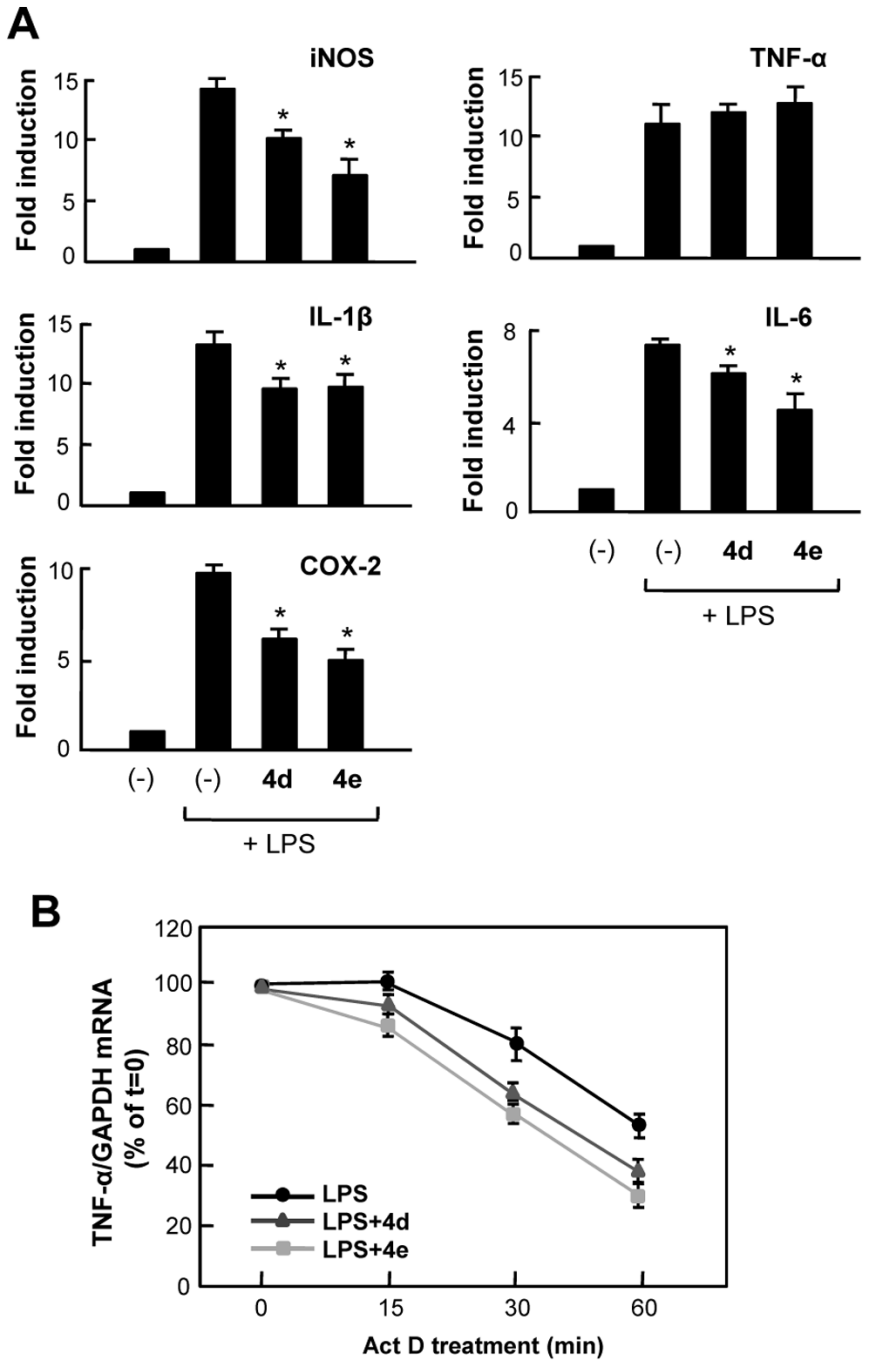
Effects of 4d and 4e on mRNA expressions of proinflammatory molecules in LPS-stimulated BV2 microglial cells. (A) Effect of **4d** and **4e** on mRNA expression of iNOS, COX-2 and pro-inflammatory cytokines in LPS-stimulated BV2 cells. Cell were pre-treated with **4d** or **4e** (5 µM) for 1 h prior to LPS stimulation. Total RNA was isolated after 6 h of LPS treatment. The mRNA levels for iNOS, TNF-α, IL-1β, IL-6 and COX-2 were measured by quantitative real-time PCR. The gene expression was normalized by GAPDH expression. Data are represented as mean ± S.E.M. of three independent experiments. *P<0.05; significantly different from LPS-treated cells. (B) BV2 cells were pre-treated with **4d** or **4e** (5 µM) for 1 h, and then stimulated with LPS for 3 h. Subsequently, 10 µg/ml actinomycin D (Act D) was added. After 0 min, 15 min, 30 min, and 1 h, cells were harvested and TNF-α mRNA expression was quantified by real-time PCR. The graphs shows mean TNF-α mRNA normalized to GAPDH mRNA expressed as a percentage of *t* = 0 Act D treatment ± S.E.M. of three independent experiments.

We examined the inhibitory effects of **4d** and **4e** on the mRNA expression of pro-inflammatory molecules such as iNOS, COX-2, and cytokines. Real-time PCR analysis showed that both **4d** and **4e** (5 µM) significantly inhibited the mRNA expression of iNOS, COX-2, IL-1β, and IL-6 in LPS-stimulated BV2 microglial cells (Figure 7A). On the other hand, mRNA level of TNF-α was not altered by treatment with either **4d** or **4e**. These results suggest that **4d** and **4e** regulate the expression of pro-inflammatory cytokines such as iNOS, COX-2, IL-1β, and IL-6 at the mRNA level, whereas they do not affect mRNA expression of TNF-α. Therefore, we hypothesized that **4d** and **4e** influence TNF-α at the post-transcriptional level. To address this possibility, we measured the disappearance of TNF-α mRNA after blocking new transcription with actinomycin D. Real-time PCR analysis showed that **4d**/**4e** induced more rapid degradation of TNF-α mRNA compared with vehicle-treated groups, which supports our hypothesis that **4d** and **4e** regulate TNF-α at the post-transcriptional level (Figure 7B).

**Figure 8:**
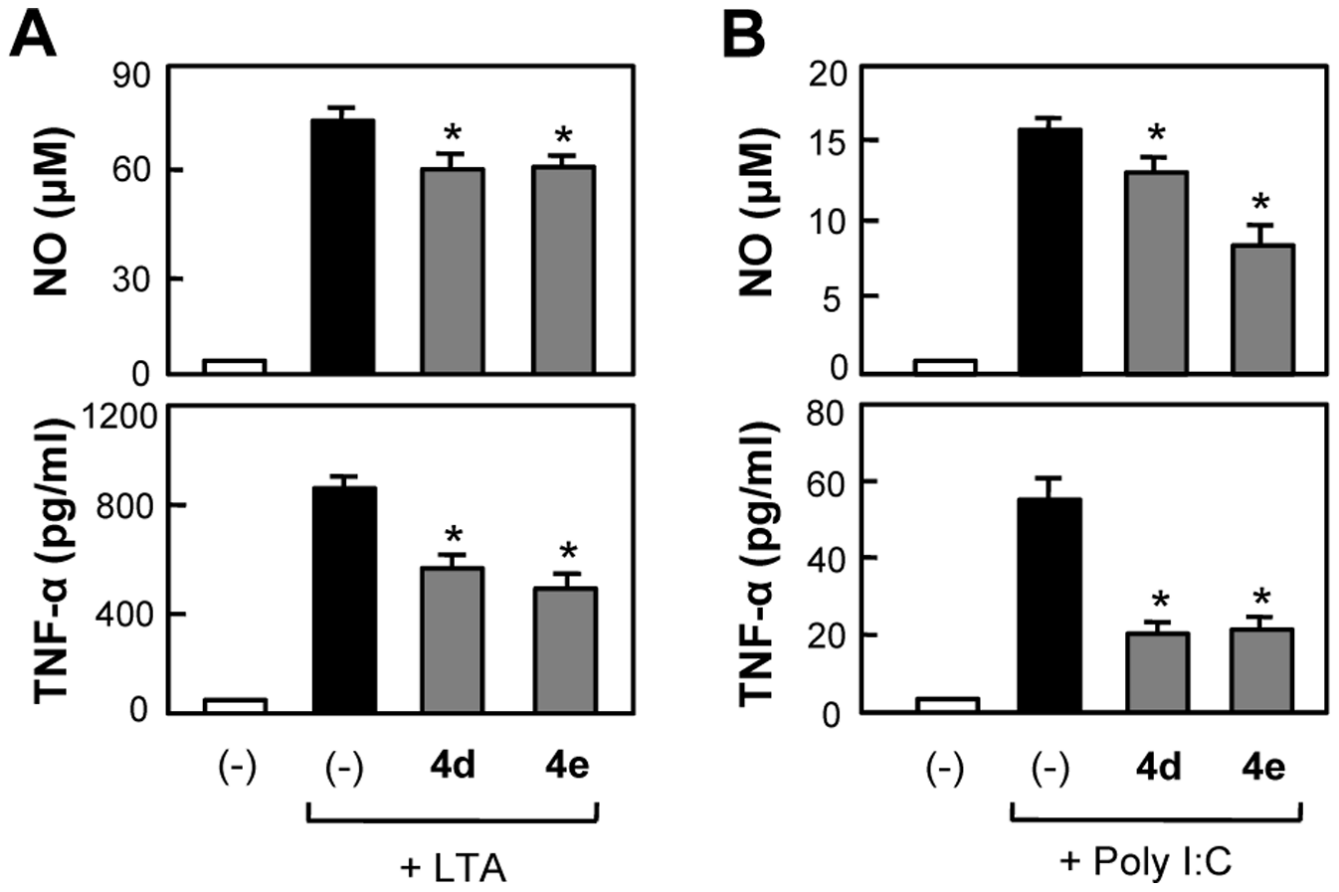
Inhibitory effects of 4d and 4e on NO and TNF-α production in LTA- or Poly I∶C-stimulated BV2 microglial cells. Cells were incubated for 16 h with LTA (10 µg/mL) (A) or Poly I∶C (10 µg/mL) (B) in the absence or presence of α-GalCer analogs **4d** and **4e** (5 µM), and the amounts of released NO and TNF-α were measured in supernatants. Treatment with α-GalCer analogs alone did not affect NO or TNF-α production. Bars indicate the mean ± S.E.M. of three independent experiments. *P<0.05; significantly different from stimulated microglial cells.

To demonstrate that anti-inflammatory effects of **4d** and **4e** are not confined to LPS stimulation, we examined the inhibitory effect of **4d** and **4e** in other agonist-stimulated conditions. As shown in Figure 8A and 8B, NO release and TNF-α production in lipoteichoic acid (LTA, TLR2 agonist)- [29], [30] or PolyI∶C (TLR3 agonist)-stimulated BV2 cells [31], [32] were also inhibited upon treatment with **4d** and **4e**, confirming that **4d** and **4e** exert anti-inflammatory effects in microglia under various inflammatory conditions.

### 4. Inhibitory effects of 4d and 4e on MAP kinases and NF-κB/AP-1, which are upstream signaling molecules in microglial activation

**Figure 9:**
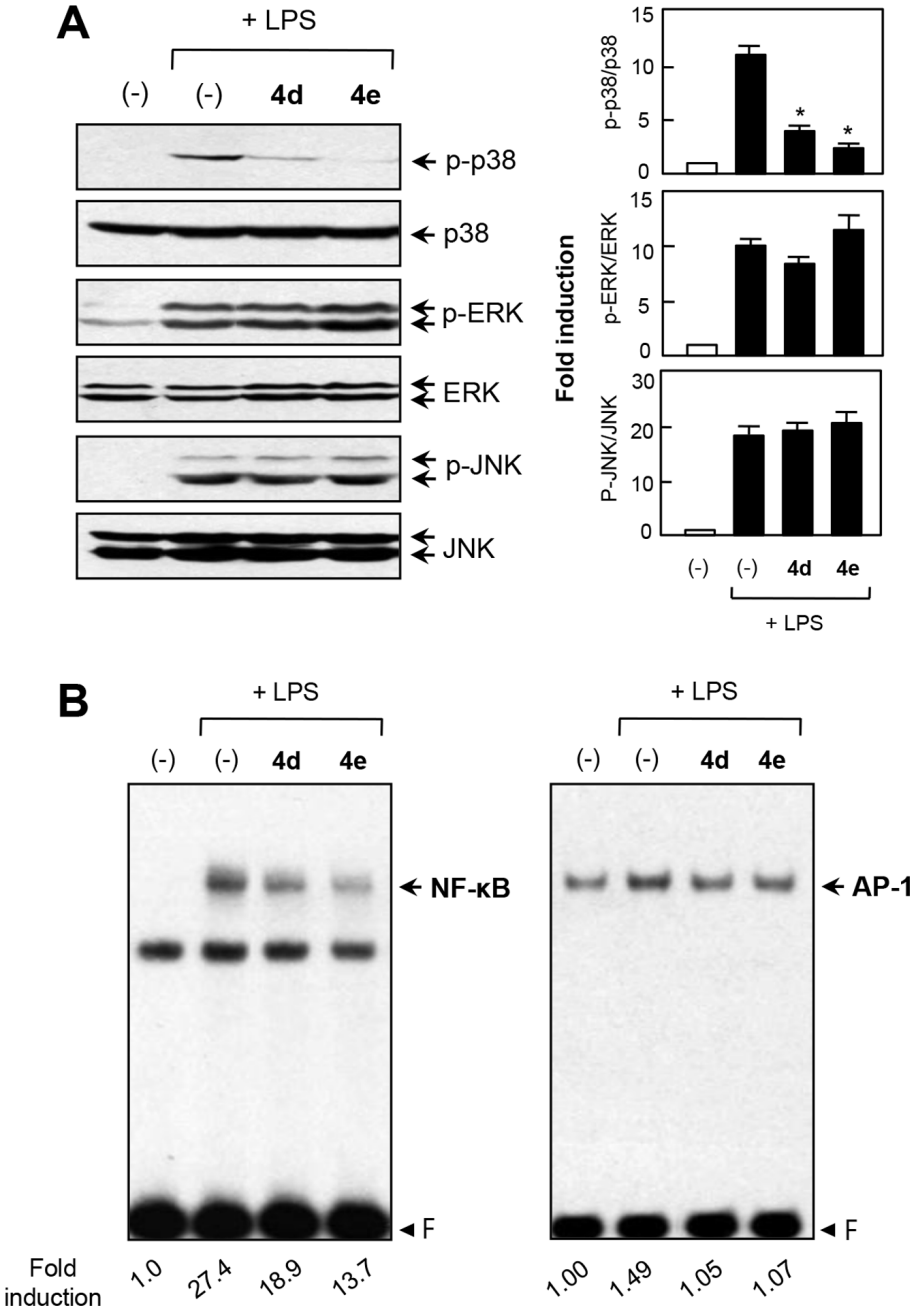
Effects of 4d and 4e on three types of MAP kinases and NF-κB/AP-1. (A) Cell extracts were prepared from BV2 microglial cells treated with LPS for 30 min in the absence or presence of **4d** or **4e** (5 µM) and then subjected to immunoblot analysis using antibodies against phospho- or total forms of three MAP kinases. Quantification of western blot data (right panel). Levels of the active forms of MAPKs were normalized to total forms and are expressed as fold changes versus untreated control samples, which were arbitrarily set to 1.0. Bars indicate mean ± S.E.M. of three independent experiments. *P<0.05; significantly different from LPS-treated cells. (B) EMSA was performed using nuclear extracts isolated from BV2 microglial cells treated with **4d** or **4e** in the presence of LPS for 3 h. Images are representative of at least three independent experiments. ‘F’ indicates free probe.

**Figure 10:**
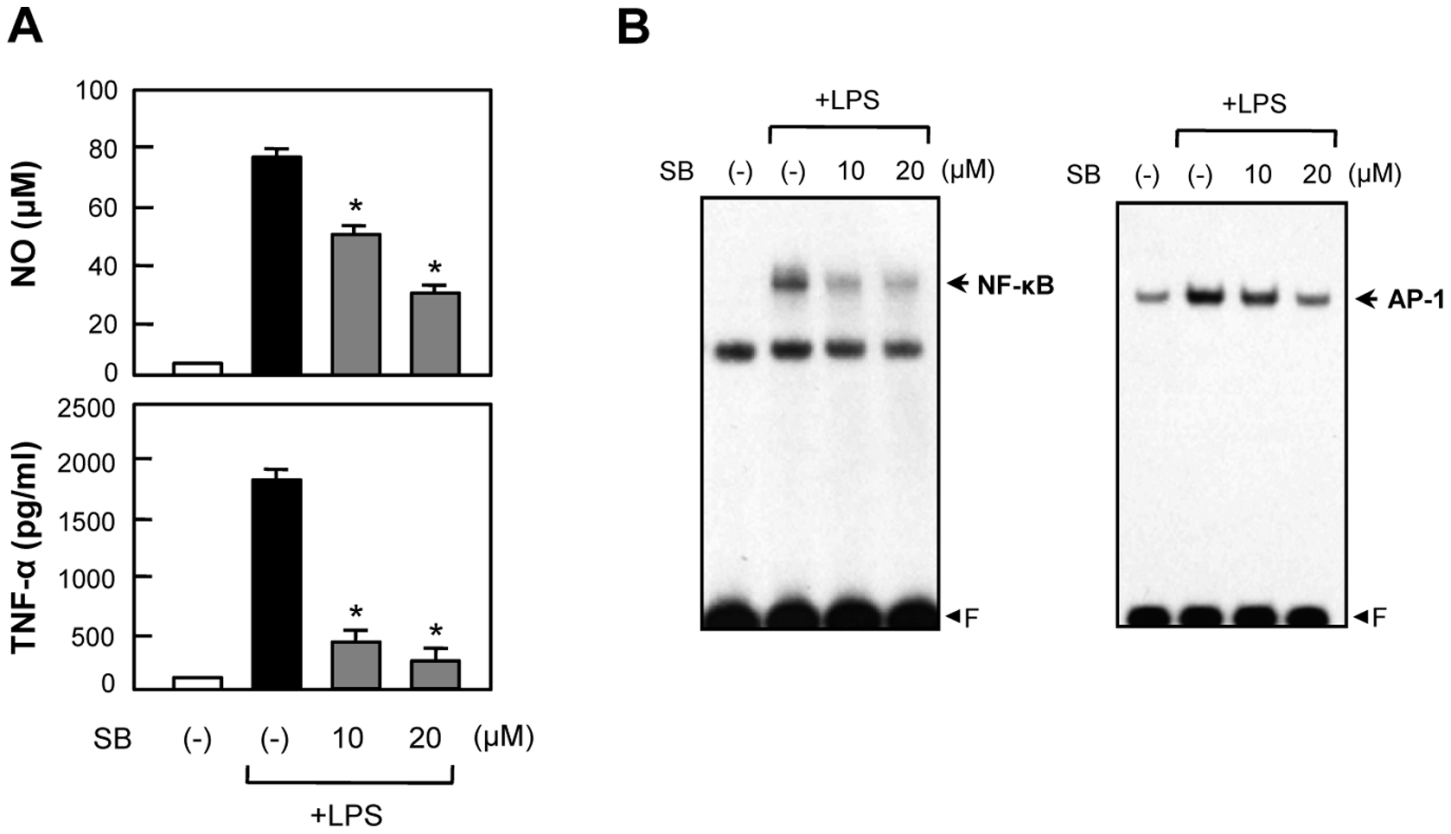
The p38 MAPK-specific inhibitor SB203580 inhibited NO and TNF-α production by suppressing NF-κB and AP-1 activities. (A) BV2 cells were treated with LPS for 16 h in the absence or presence of SB203580, and released NO and TNF-α were measured as previously described. Bars indicate the mean ± S.E.M. of three independent experiments. *P<0.05; significantly different from LPS-treated cells. SB indicates SB203580. (B) Effect of SB203580 on LPS-induced NF-κB and AP-1 DNA binding activities.

To analyze the molecular mechanism underlying the anti-inflammatory effects of **4d** and **4e**, we further examined their inhibitory effect on phosphorylation of MAP kinases, which are upstream signaling molecules in inflammatory responses. Western blot analysis revealed that **4d** and **4e** markedly inhibited LPS-induced p38 MAPK phosphorylation. However, **4d** and **4e** did not affect phosphorylation of ERK or JNK (Figure 9A). In addition, **4d** and **4e** inhibited the DNA binding activities of NF-κB and AP-1, which are key transcription factors for inflammatory gene expression in microglial cells (Figure 9B). To confirm an involvement of the p38 pathway in the anti-inflammatory effects of **4d** and **4e**, we examined the effect of a p38 MAPK-specific inhibitor, SB203580, on microglial activation. As shown in Figure 10A, SB203580, like **4d** and **4e**, significantly inhibited NO and TNF-α production in LPS-stimulated microglia. Furthermore, identical to **4d** and **4e**, SB203580 inhibited the DNA binding activities of NF-κB and AP-1 (Figure 10B). Thus, these data collectively suggest that p38 MAPK plays a key role in the anti-inflammatory effects of **4d** and **4e** via the modulation of NF-κB and AP-1 activities.

## Discussion

In the present study, we demonstrated the anti-inflammatory effect of systematically designed α-GalCer analogs in activated microglia. The anti-inflammatory activities of α-GalCer in brain inflammation have not been previously explored. After the construction of α-GalCer analogs via systematic diversification of acyl chains and sphingosine backbones, we tested their inhibitory activities against the production of NO, ROS, and TNF-α, which are key neurotoxic and pro-inflammatory molecules produced in activated microglia. Surprisingly, α-GalCer analogs KRN7000 and OCH, which are known to be bioactive in iNKT cells, showed no or marginal effects on LPS-stimulated microglia. Among five different sphingosine backbone structures of our α-GalCer analogs, an additional phenyl moiety resulted in the largest enhancement of inhibitory activity toward NO, ROS, and TNF-α production. Relatively short acyl chains also enhanced anti-inflammatory activity. In particular, **4d** and **4e**, with a terminal phenyl group in their acyl chains, prominently suppressed the production of pro-inflammatory molecules in a dose-dependent manner.

On the basis of molecular mechanistic analysis, we demonstrated that the p38 MAPK signaling pathway is largely involved in **4d**/**4e**-mediated anti-inflammatory effects in LPS-stimulated microglia. p38, one of the members of the MAPK family, plays an important role in a variety of biological systems [33]. In particular, p38 MAPK regulates inflammatory conditions in the central nervous system via modulating the activities of various transcription factors [34], [35], and inhibition of p38 blocks the *in vitro* and *in vivo* production of IL-4, IL-10, TNF-α, and other cytokines [36], [37]. In the present study, we found that **4d** and **4e** modulate TNF-α at the post-transcriptional level, which may be related to p38 MAPK inhibition by **4d**/**4e** because p38 MAPK is known to be involved in post-transcriptional regulation of TNF-α [38]. The TNF-α mRNA contains AU-rich regions in the 3′UTR that are normally occupied by AU-binding proteins. Under normal conditions, this leads to a blockade of translation and rapid turnover of transcripts. Following activation of p38 MAPK, these AU-binding proteins are phosphorylated, resulting in their release from AU-rich regions and allowing translation and secretion of TNF-α [39], [40]. Thus, inhibitors of p38 MAPK may target these events and inhibit translation of TNF-α. Therefore, **4d/4e**-mediated post-transcriptional control of TNF-α may be closely related with their inhibitory effect on p38. Collectively, our results suggest that the specific inhibition of p38 activity by **4d** and **4e** may have therapeutic benefits for neuroinflammatory disorders such as trauma, stroke, and multiple sclerosis [37], [41].

We recently demonstrated that cell-permeable short chain C2 ceramide exerts anti-inflammatory effects partly by interfering with the interaction of LPS and TLR-4 on cell surfaces [42]. LPS, a constituent of gram negative bacteria, binds to TLR4 and evokes intracellular inflammatory signaling cascades, including IL-1 receptor-associated kinase, IKK/NF-κB, and MAPK activation [43]. Based on the fact that the lipid A chain, the biologically active core of LPS, is structurally similar to ceramide, the agonistic or antagonistic influence of ceramide on TLR4 has been suggested [44], [45]. We have also described a series of α-GalCer analogs containing heterocyclic and phenyl moieties in the sphingosine backbone and demonstrated that treatment with a T_H_2-biased α-GalCer analog selectively stimulates the secretion of anti-inflammatory cytokines in iNKT cells [25], [26]. Therefore, it is possible that α-GalCer **4d** and **4e** may exert anti-inflammatory effects in stimulated microglia in part by interfering with the binding of LPS to TLR4 or by affecting other membranous or intracellular components after intercalating into membrane or cells. Further studies are necessary to investigate the detailed mechanism underlying the effect of α-GalCer analogs **4d** and **4e**. To address whether the anti-inflammatory effects of α-GalCer analogs are confined to microglia, we tested the effect of **4d** and **4e** in the Raw264.7 murine macrophage cell line. We observed that **4d** and **4e** suppressed NO production in LPS-stimulated Raw264.7 cells (unpublished data). However, they did not affect TNF-α release. The data suggest that the effects of **4d/4e** are somewhat different between cell types.

In conclusion, we report, for the first time, the anti-inflammatory effects of a novel series of α-GalCer analogs in activated microglia under various inflammatory conditions. Our study of molecular mechanisms revealed that α-GalCer analogs **4d** and **4e** inhibited the phosphorylation of p38 MAPK and the DNA binding activities of NF-κB and AP-1. Because microglial activation plays an important role in neurodegenerative diseases, the selective suppression of microglial activation by α-GalCer analogs **4d** and **4e** may have therapeutic potential for neuroinflammatory disorders.

## Supporting Information

Supporting Information S1 **Synthetic procedure and spectroscopic data of all new compounds.**
(PDF)
