# Supplementary material for: Anti-Inflammatory Effects of α-Galactosylceramide Analogs in Activated Microglia: Involvement of the p38 MAPK Signaling Pathway
Source: PLoS One. 2014 Feb 11;9(2):e87030. doi: 10.1371/journal.pone.0087030 (PMC3921125; doi:10.1371/journal.pone.0087030)
Supplement: Supporting Information S1 — Synthetic procedure and spectroscopic data of all new compounds. (PDF) [file pone.0087030.s001.pdf]

< Supporting Information >

Anti-inflammatory effects of α-galactosylceramide derivatives in activated microglia; involvement of p38 MAPK signaling pathway

Yeon-Hui Jeong,[1] Yongju Kim,[2] Heebum Song,[2] Young Sun Chung[3],
Seung Bum Park[2,4,*], and Hee-Sun Kim[1,*]

[1]*Department of Molecular Medicine, Tissue Injury Defense Research Center, Ewha Womans University Medical School, Seoul, Republic of Korea*

[2]*Department of* Chemistry, *Seoul National University, Seoul, Republic of Korea*

[3]*Department of Counseling Psychology, Korea Soongsil Cyber University, Seoul 110-340, Republic of Korea*

[4]*Department of* Biophysics and Chemical Biology/Bio-MAX Institute, *Seoul National University, Seoul, Republic of Korea*

[*]**Address correspondence and reprints requests to:** Hee-Sun Kim, Department of Molecular Medicine, Ewha Womans University, Mok-6-dong 911-1, Yangchun-gu, Seoul 158-710, South Korea, Tel: 82-2-2650-5823, Email: hskimp@ewha.ac.kr

Or

Seung Bum Park, Department of Chemistry, Seoul National University, 1 Gwanak-ro Gwanak-gu, Seoul 151-747, Korea. Tel: +82-2-880-9090, Email: sbpark@snu.ac.kr

List of contents

		page
I.	General experimental information	S2
II.	Synthetic procedure and spectroscopic data	S3-S74

I. General Experimental Information

All reactions were performed either in oven-dried glassware or microwave vessel under dry argon atmosphere. Toluene and tetrahydrofuran (THF) was dried by distillation from sodium–benzophenone immediately prior to use. Dichloromethane (DCM) was dried by distillation from CaH_2. Other solvents and organic reagents were purchased from commercial venders and used without further purification unless otherwise mentioned. All reagents used for cell culture were purchased from Gibco BRL (Grand Island, NY, USA). LPS was obtained from Sigma-Aldrich (St. Louis, MO, USA). All reagents and enzymes for RT-PCR were purchased from Promega (Madison, WI, USA). Antibodies against phospho-/total form of MAP kinases were purchased form Cell Signaling Technology (Beverley, MA, USA).

^{1}H and ^{13}C NMR spectra were obtained on Varian Inova-500 (Varian Assoc., Palo Alto, USA) or Bruker DRX-300 (Bruker Biospin, Germany). Chemical shifts were reported in ppm relative to the residual solvent peak ($CDCl_3$, ^{1}H: 7.24; ^{13}C: 77.23). Multiplicity was indicated as follows: s (singlet); d (doublet); t (triplet); q (quartet); quin (quintet); m (multiplet); dd (doublet of doublet); dt (doublet triplet); br s (broad singlet), etc. Coupling constants were reported in Hz. Routine mass analyses were performed on LC/MS system using electron spray ionization (ESI) or atmospheric pressure chemical ionization (APCI). The high resolution mass spectrometric analyses were conducted at the Mass Spectrometry Laboratory of Seoul National University using mass spectrometer by direct injection for fast atomic bombardment (FAB). The products were purified by flash column chromatography on silica gel (230–400 mesh). The eluent used for purification is reported in parentheses. Thin-layer chromatography (TLC) was performed on pre-coated glass-backed plates (silica gel 60 F_{254} 0.25mm), and components were visualized by observation under UV light (254 and 365 nm) or by treating the plates with anisaldehyde, $KMnO_4$, phosphomolybdic acid, and vanillin followed by heating. Distilled water was polished by ion exchange and filtration.

II. Synthetic Procedures and Spectroscopic Data

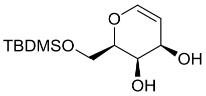

Compound 6. D-Galactal (1.0 g, 6.84 mmol), *tert*-butyldimethylsilyl chloride (1.13 g, 7.52 mmol) and imidazole (930 mg, 13.68 mmol) were stirred in anhydrous dimethylformamide (DMF, 10 mL) at room temperature for 12 h. After the completion of reaction monitored by TLC, DMF was evaporated under reduced pressure and the resulting mixture was diluted with ethyl acetate (EtOAc) and washed with saturated $NaHCO_3$(aq) and brine. The combined organic layer was dried over anhydrous Na_2SO_4 (s). The filtrate was condensed under reduced pressure and purified by silica-gel flash column chromatography (EtOAc:Hex = 1:1, v/v) to provide desired product **6** (1.35 g, 76%) as an amorphous white solid; $[\alpha]_D^{25}$ +4.8 (c = 2.55, $CHCl_3$); ^{1}H NMR (500 MHz, $CDCl_3$) δ 6.39 (dd, *J* = 6.3, 1.3 Hz, 1H), 4.73 (dt, *J* = 6.3, 2.0 Hz, 1H), 4.33–4.30 (m, 1H), 4.13–4.10 (m, 1H), 4.00–3.88 (m, 3H), 3.20 (d, *J* = 5.0 Hz, 1H), 2.73 (d, *J* = 10.5 Hz, 1H), 0.91 (s, 9H), 0.11 (s, 6H); LRMS (ESI$^+$) *m/z* calcd for $C_{12}H_{24}NaO_4Si$ [M + Na]$^+$: 283.13; Found: 283.23.

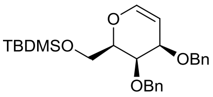

Compound 7. To a solution of compound **6** (1.21 g, 4.647 mmol) in anhydrous THF (25 mL) stirred at 0 ℃, was added sodium hydride (690 mg in 60% mineral oil, 17.19 mmol) over a 15 min period. The reaction mixture was stirred for 30 min and warm up to room temperature. Then, benzyl bromide (1.77 mL, 14.87 mmol) and tetra-*n*-butylammonium iodide (TBAI, 170 mg, 0.4647 mmol) were added carefully in dropwise. The mixture was stirred at room temperature for overnight under nitrogen atmosphere. After the completion of reaction monitored by TLC, the resultant was diluted with EtOAc and washed twice with brine. The combined organic layer was dried over anhydrous Na_2SO_4(s). The filtrate was condensed under reduced pressure and purified by silica-gel flash column chromatography (EtOAc:Hex = 1:3, v/v) to provide the desired product **7** (1.60 g, 78%) as an amorphous white solid; $[\alpha]_D^{25}$ –40.9 (c = 2.23, $CHCl_3$); ^{1}H NMR (500 MHz, $CDCl_3$) δ 7.38–7.25 (m, 10H), 6.35 (dd, *J* = 6.5, 1.5 Hz, 1H), 4.91 (dd, *J* = 11.5 Hz, 1H), 4.85 (dq, *J* = 3.0, 1.3 Hz, 1H), 4.70–4.60 (m, 3H), 4.22–4.20 (m, 1H), 4.02–3.97 (m, 2H), 3.90–3.79 (m, 2H), 0.89 (s, 9H), 0.05 (s, 6H); LRMS (ESI$^+$) *m/z* calcd for $C_{26}H_{37}O_4Si$ [M + H]$^+$: 441.25; Found: 441.30.

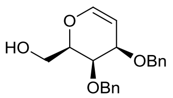

Compound 8. To a solution of compound **7** (800 mg, 1.815 mmol) in anhydrous tetrahydrofuran (THF, 15 mL) was added tetra-*n*-butylammonium fluoride (2.72 mL in 1.0 M THF solution, 2.72 mmol) over a 10 min period. The mixture was stirred at room temperature for 2 h under nitrogen atmosphere. After the completion of reaction monitored by TLC, the resultant was diluted with EtOAc and washed twice with brine. The combined organic layer was dried over anhydrous Na_2SO_4(s). The filtrate was condensed under reduced pressure and purified by silica-gel flash column chromatography (EtOAc:Hex = 1:2, v/v) to provide the desired products **8** (527 mg, 89%) as a colorless oil; $[\alpha]_D^{25}$ –101.9 (c = 2.15, $CHCl_3$); ^{1}H NMR (500 MHz, $CDCl_3$) δ 7.37–7.24 (m, 10H), 6.40 (d, *J* = 6.0 Hz, 1H), 4.86–4.82 (m, 2H), 4.72–4.62 (m, 3H), 4.18–4.16 (m, 1H), 4.11–4.09 (m, 1H), 3.99–3.95 (m, 2H), 3.75–3.72 (m, 1H), 2.54 (br s, 1H); LRMS (ESI^+) *m/z* calcd for $C_{20}H_{22}O_4$ $[M + H]^+$: 326.15; Found: 326.09.

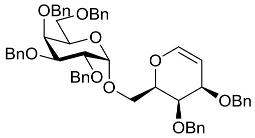

Compound 9. To a solution of 1-*O*-acetyl-2,3,4,6-tetra-*O*-benzyl-D-galactopyranoside (2.95 g, 5.055 mmol) in DCM (30 mL) at 0 °C was added trimethylsilyl iodide (TMSI, 719 μL, 5.055 mmol) and stirred for at 0 °C for 30 min. The reaction was stopped by adding 30 mL of anhydrous toluene and the residual TMSI was removed by azeotrope-evaporation with anhydrous toluene in three times. The slightly yellow residue was dissolved in anhydrous benzene (10 mL) and kept under argon atmosphere. In a separate round-bottom flask, molecular sieves (MS, 4 Å, 1 g), TBAI (5.6 g, 15.2 mmol), compound **8** (550 mg, 1.685 mmol), and diisopropylethylamine (880 μL, 5.055 mmol) were added into anhydrous benzene (10 mL). The reaction mixture was stirred under argon at 65 °C for 10 min. Upon the complete dissolution of TBAI, the glycosyl iodide in anhydrous benzene was added into this flask and the reaction mixture was stirred at 65 °C for 2 h. The reaction was stopped by adding EtOAc (100 mL) and cooled to 0 °C. The white precipitate and MS were removed by filtration through Celite® -packed glass filter with EtOAc-washing. The filtrate was washed with sat. $Na_2S_2O_3$(aq) (2 × 100 mL) and brine, dried over anhydrous Na_2SO_4. The filtrate was condensed under reduced pressure and purified by silica-gel flash column chromatography (EtOAc:Hex = 1:5,

v/v) to provide the desired product **9** (1.26 g, 88%) as a colorless oil; $[\alpha]_D^{19}$ +8.6 (c = 2.25, $CHCl_3$); ^{1}H NMR (500 MHz, $CDCl_3$) δ 7.39–7.22 (m, 30H), 6.24 (dd, *J* = 6.3, 1.2 Hz, 1H), 4.93 (d, *J* = 11.5 Hz, 1H), 4.83−4.66 (m, 5H), 4.65–4.54 (m, 5H), 4.44–4.37 (m, 2H), 4.28–4.26 (m, 1H), 4.14–4.11 (m, 1H), 4.04–3.95 (m, 5H), 3.91−3.90 (m, 1H), 3.71 (dd, *J* = 10.7, 4.7 Hz, 1H), 3.56–3.46 (m, 2H); ^{13}C NMR (75 MHz, $CDCl_3$) δ 144.3, 139.1, 138.9, 138.8, 138.8, 138.5, 138.2, 128.7, 128.7, 128.6, 128.5, 128.5, 128.4, 128.1, 128.1, 128.0, 127.9, 127.8, 127.8, 127.7, 127.6, 127.6, 99.9, 98.0, 79.3, 76.6, 75.2, 75.2, 75.0, 73.6, 73.2, 72.0, 71.0, 69.4, 69.0, 66.5, 53.6; LRMS (ESI$^+$) *m/z* calcd for $C_{54}H_{57}O_9$ $[M + H]^+$: 849.40; Found: 849.51.

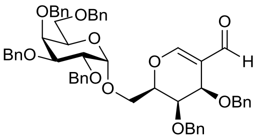

Compound 10. To a solution of compound **9** (1.2 g, 1.413 mmol) in DMF (14 mL) stirred at 0 ℃, was added $POCl_3$ (263 μL, 2.827 mmol) over a 30-min period. The reaction mixture was stirred for 1 h and warm up to room temperature. After 18 h, the resultant was diluted with EtOAc and washed with saturated $NaHCO_3$(aq) and brine. Then, the combined organic layer was dried over anhydrous Na_2SO_4(s). The filtrate was condensed under reduced pressure and purified by silica-gel flash column chromatography (EtOAc:Hex = 1:3, v/v) to provide the desired products **10** (556 mg, 45%) as a colorless oil; $[\alpha]_D^{25}$ +38.9 (c = 0.81, $CHCl_3$); ^{1}H NMR (500 MHz, $CDCl_3$) δ 8.96 (s, 1H), 7.41–7.22 (m, 30H), 6.94 (s, 1H), 4.96 (d, *J* = 11.5 Hz, 1H), 4.86 (d, *J* = 11.5 Hz, 1H), 4.83 (d, *J* = 12.0 Hz, 1H), 4.77–4.50 (m, 10H), 4.38–4.32 (m, 2H), 4.09–3.97 (m, 6H), 3.74 (dd, *J* = 5.2, 3.8 Hz, 1H), 3.47–3.42 (m, 2H); LRMS (ESI$^+$) *m/z* calcd for $C_{55}H_{57}O_{10}$ $[M + H]^+$: 877.40; Found: 877.59.

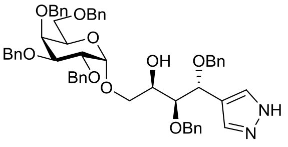

Compound 11. Compound **10** (552 mg, 0.629 mmol) and hydrazine monohydrate (61 μL, 1.259 mmol) were dissolved in MeOH (6 mL), and the mixture was stirred at room temperature for 2 h. After completion of reaction as indicated by TLC, the resulting mixture was condensed under reduced pressure without more purification to provide the desired product **11** (560 mg, quantitative yield) as a colorless oil; $[\alpha]_D^{19}$ +9.5 (c = 1.66, $CHCl_3$); ^{1}H NMR (500 MHz, $CDCl_3$)

δ 7.52 (s, 2H), 7.51–7.10 (m, 30H), 4.92 (d, *J* = 11.5 Hz, 1H), 4.83 (d, *J* = 4.0 Hz, 1H), 4.79 (d, *J* = 12.0 Hz, 1H), 4.75 (d, *J* = 12.0 Hz, 1H), 4.69 (d, *J* = 12.0 Hz, 1H), 4.66–4.64 (m, 2H), 4.55 (d, *J* = 11.0 Hz, 1H), 4.46–4.43 (m, 2H), 4.37–4.35 (m, 2H), 4.31–4.27 (m, 2H), 4.10–4.03 (m, 2H), 3.99 (m, 1H), 3.93–3.91 (m, 2H), 3.72 (dd, *J* = 10.5, 6.5 Hz, 1H), 3.66 (dd, *J* = 6.5, 3.0 Hz, 1H), 3.53–3.48 (m, 3H); LRMS (ESI$^+$) *m/z* calcd for $C_{55}H_{59}N_2O_9$ $[M + H]^+$: 891.42; Found: 891.45.

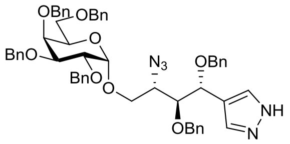

Compound 13. To a solution of compound **11** (560 mg, 0.628 mmol) and DIPEA (547 μL, 3.142 mmol) in dry DCM (5 mL) stirred at 0 ℃, was added MsCl (146 μL, 1.884 mmol) in dropwise. The reaction mixture was stirred for 3 h and the resultant was diluted with EtOAc and washed with saturated $NaHCO_3$(aq) and brine. Then, the combined organic layer was dried over anhydrous Na_2SO_4(s). The filtrate was condensed under reduced pressure to provide the desired crude product **12**. Crude compound **12** and NaN_3 (817 mg, 12.56 mmol) were dissolved in DMF (3 mL) and stirred at 120 ℃ for 2.5 days. After completion of reaction as indicated by TLC, the resulting mixture was diluted with EtOAc and washed with brine. The combined organic layer was dried over anhydrous Na_2SO_4(s). The filtrate was condensed under reduced pressure and purified by silica-gel flash column chromatography (EtOAc:Hex = 1:1, v/v) to provide the desired product **13** (328 mg, 57%) as a colorless oil; $[\alpha]_D^{25}$ +7.4 (c = 1.60, $CHCl_3$); ^1H NMR (500 MHz, $CDCl_3$) δ 7.56 (s, 2H), 7.38–7.10 (m, 30H), 4.93 (d, *J* = 11.0 Hz, 1H), 4.86 (d, *J* = 3.5 Hz, 1H), 4.83–4.78 (m, 2H), 4.73 (d, *J* = 11.5 Hz, 1H), 4.68–4.55 (m, 5H), 4.46 (d, *J* = 11.5 Hz, 1H), 4.42 (d, *J* = 12.0 Hz, 1H), 4.35 (d, *J* = 11.5 Hz, 1H), 4.31 (d, *J* = 11.5 Hz, 1H), 4.05 (dd, *J* = 10.0, 3.0 Hz, 1H), 3.98 (dd, *J* = 10.0, 2.5 Hz, 1H), 3.94–3.90 (m, 4H), 3.69 (dd, *J* = 10.5, 7.0 Hz, 1H), 3.48–3.42 (m, 3H); LRMS (ESI$^+$) *m/z* calcd for $C_{55}H_{58}N_5O_8$ $[M + H]^+$: 916.43; Found: 916.38.

General Procedure for the *N*-alkylation of pyrazole 14

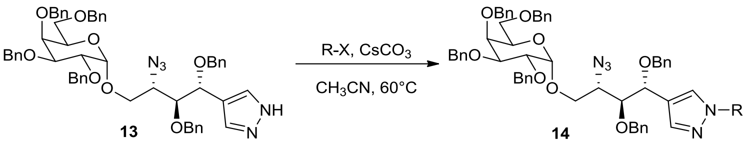

Compound **13**, alkylating agent (1.5 equiv.) and $CsCO_3$ (3 equiv.) were stirred in CH_3CN (0.05 M concentration) at 60℃ for 10 h. After the completion of reaction monitored by TLC, CH_3CN was evaporated under reduced pressure and the resulting mixture was diluted with EtOAc and washed with brine. The combined organic layer was dried over anhydrous Na_2SO_4(s). The filtrate was condensed under reduced pressure and purified by silica-gel flash column chromatography (EtOAc:Hex = 1:3, v/v) to provide desired product **14** (>90% yield) as a colorless oil.

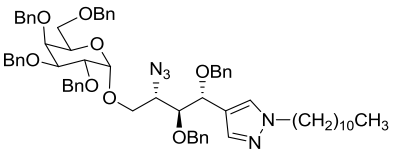

Compound **14a**; 92% yield; ^{1}H NMR (500 MHz, $CDCl_3$) δ 7.50 (s, 1H), 7.38–7.20 (m, 29H), 7.17–7.15 (m, 2H), 4.93 (d, *J* = 11.0 Hz, 1H), 4.87 (d, *J* = 3.5 Hz, 1H), 4.83–4.77 (m, 2H), 4.75–4.73 (m, 1H), 4.67 (d, *J* = 12.0 Hz, 1H), 4.61–4.54 (m, 4H), 4.48–4.43 (m, 2H), 4.36 (d, *J* = 12.0 Hz, 1H), 4.30 (d, *J* = 12.0 Hz, 1H), 4.08–4.06 (m, 3H), 3.98 (dd, *J* = 10.3 Hz, 2.5 Hz, 1H), 3.95–3.90 (m, 4H), 3.70 (dd, *J* = 11.0, 7.0 Hz, 1H), 3.51–3.45 (m, 3H), 1.82 (quin, *J* = 7.0 Hz, 2H), 1.30–1.24 (m, 18H), 0.87 (t, *J* = 7.0 Hz, 3H); ^{13}C NMR (75 MHz, $CDCl_3$) 139.5, 139.0, 138.9, 138.9, 138.5, 138.4, 138.2, 129.2, 128.6, 128.5, 128.5, 128.4, 128.4, 128.4, 128.4, 128.4, 128.1, 128.0, 127.9, 127.8, 127.7, 127.7, 127.7, 127.6, 127.5, 117.8, 98.8, 80.8, 79.1, 76.5, 75.3, 75.0, 74.9, 74.6, 73.6, 73.4, 73.3, 73.0, 70.5, 69.9, 69.2, 67.9, 62.3, 52.5, 32.1, 30.5, 29.8, 29.8, 29.7, 29.5, 29.3, 26.9, 22.9, 14.3.

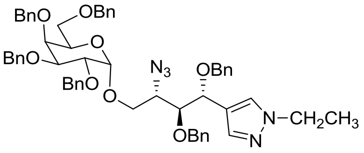

Compound **14b**; 91% yield; $[\alpha]_D^{25}$ +10.7 (c = 0.25, $CHCl_3$); ^{1}H NMR (500 MHz, $CDCl_3$) δ 7.51 (s, 1H), 7.38–7.35 (m, 2H), 7.33–7.21 (m, 27H), 7.17–7.15 (m, 2H), 4.93 (d, *J* = 11.5 Hz, 1H), 4.87 (d, *J* = 3.0 Hz, 1H), 4.83–4.78 (m, 2H), 4.73 (d, *J* = 12.0 Hz, 1H), 4.68 (d, *J* = 12.0 Hz, 1H), 4.62 (d, *J* = 1.5 Hz, 2H), 4.59 (d, *J* = 5.0 Hz, 1H), 4.56 (d, *J* = 11.0 Hz, 1H), 4.89–4.42 (m, 2H), 4.37–4.30 (m, 2H), 4.11 (q, *J* = 7.3 Hz, 2H), 4.05 (dd, *J* = 10.0, 4.0 Hz, 1H),

3.98 (dd, *J* = 10.0, 3.0 Hz, 1H), 3.94–3.91 (m, 4H), 3.69 (dd, *J* = 10.8, 6.8 Hz, 1H), 3.50–3.46 (m, 3H), 1.45 (t, *J* = 7.5 Hz, 3H); ^{13}C NMR (75 MHz, $CDCl_3$) δ 139.5, 139.0, 138.9, 138.8, 138.5, 138.7, 138.2, 128.6, 128.6, 128.5, 128.5, 128.5, 128.4, 128.4, 128.4, 128.4, 128.1, 128.0, 127.9, 127.9, 127.8, 127.8, 127.7, 127.6, 117.9, 98.8, 80.7, 79.1, 76.5, 75.2, 74.9, 74.9, 74.7, 73.6, 73.4, 73.3, 70.5, 69.9, 69.2, 68.0, 62.2, 60.6, 47.2, 15.6.

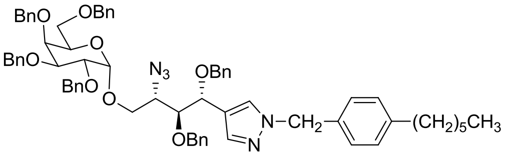

Compound **14c**; 93% yield; ^{1}H NMR (500 MHz, $CDCl_3$) δ 7.54 (s, 1H), 7.38–7.36 (m, 2H), 7.33–7.21 (m, 29H), 7.13–7.09 (m, 4H), 4.93 (d, *J* = 11.5 Hz, 1H), 4.86 (d, *J* = 3.5 Hz, 1H), 4.83–4.66 (m, 4H), 4.58–4.52 (m, 5H), 4.45–4.42 (m, 3H), 4.34 (d, *J* = 11.5 Hz, 1H), 4.27 (d, *J* = 11.5 Hz, 1H), 4.04 (dd, *J* = 3.5, 10.0 Hz, 1H), 3.97 (dd, *J* = 10.0, 2.5 Hz, 1H), 3.93–3.87 (m, 4H), 3.69 (dd, *J* = 10.8, 6.5 Hz, 1H), 3.54–3.52 (m, 1H), 3.47 (d, *J* = 6.0 Hz, 2H), 2.57 (t, *J* = 8.0 Hz, 2H), 1.57 (quin, *J* = 7.5 Hz, 2H), 1.34–1.27 (m, 6H), 0.87 (t, *J* = 6.8 Hz, 3H); ^{13}C NMR (75 MHz, $CDCl_3$) δ 143.1, 139.8, 139.0, 138.9, 138.9, 138.4, 138.3, 138.2, 129.6, 129.1, 128.6, 128.5, 128.4, 128.0, 128.0, 127.9, 127.9, 127.7, 127.7, 127.6, 118.7, 98.8, 80.8, 79.1, 76.5, 75.2, 74.9, 74.8, 74.4, 73.6, 73.4, 73.2, 70.5, 69.9, 69.2, 67.9, 62.3, 56.1, 35.8, 31.9, 31.6, 29.9, 29.2, 22.8, 14.3; LRMS (ESI^{+}) *m/z* calcd for $C_{68}H_{75}N_5O_8$ $[M + H]^{+}$: 1090.56; Found: 1090.57.

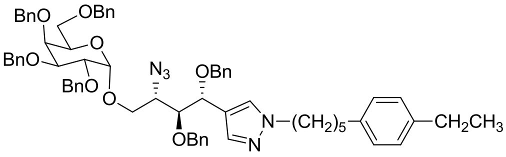

Compound **14d**; 92% yield; $[\alpha]_D^{25}$ +8.7 (c = 0.70, $CHCl_3$); ^{1}H NMR (500 MHz, $CDCl_3$) δ 7.50 (s, 1H), 7.38–7.21 (m, 29H), 7.17–7.15 (m, 2H), 7.09 (d, *J* = 8.0 Hz, 2H), 7.05 (d, *J* = 8.0 Hz, 2H), 4.93 (d, *J* = 11.5 Hz, 1H), 4.87 (d, *J* = 3.5 Hz, 1H), 4.83–4.78 (m, 2H), 4.72 (d, *J* = 11.5 Hz, 1H), 4.67 (d, *J* = 12.0 Hz, 1H), 4.61 (s, 2H), 4.58−4.54 (m, 2H), 4.47−4.42 (m, 2H), 4.35 (d, *J* = 12.0 Hz, 1H), 4.29 (d, *J* = 11.5 Hz, 1H), 4.06−4.02 (m, 3H), 3.97 (dd, *J* = 10.0, 2.5 Hz, 1H), 3.94−3.90 (m, 4H), 3.69 (dd, *J* = 10.0, 6.5 Hz, 1H), 3.49−3.46 (m, 3H), 2.60 (q, *J* = 7.7 Hz, 2H), 2.53 (t, *J* = 7.8 Hz, 2H), 1.85 (quin, *J* = 7.4 Hz, 2H), 1.61 (quin, *J* = 7.6 Hz, 2H), 1.31 (quin, *J* = 7.6 Hz, 2H), 1.21 (t, *J* = 7.5 Hz, 3H); ^{13}C NMR (75 MHz, $CDCl_3$) δ 141.8, 139.6, 139.6, 139.0, 138.9, 138.5, 138.4, 138.2, 129.3, 128.6, 128.5, 128.5, 128.4, 128.4, 128.1,

128.0, 127.9, 127.9, 127.8, 127.7, 127.6, 117.8, 98.8, 80.7, 79.1, 77.4, 76.5, 75.3, 75.0, 74.9, 74.6, 73.7, 73.4, 73.3, 70.5, 69.9, 69.2, 68.0, 62.3, 52.4, 35.5, 31.2, 30.4, 28.6, 26.5, 15.8; LRMS (ESI$^+$) *m/z* calcd for $C_{68}H_{76}N_5O_8$ [M + H]$^+$: 1090.57; Found: 1090.59.

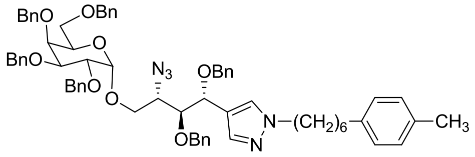

Compound **14e**; 91% yield; ^{1}H NMR (500 MHz, $CDCl_3$) δ 7.50 (s, 1H), 7.38–7.15 (m, 31H), 7.06 (d, *J* = 8.0 Hz, 2H), 7.02 (d, *J* = 8.0 Hz, 2H), 4.93 (d, *J* = 11.5 Hz, 1H), 4.86 (d, *J* = 3.0 Hz, 1H), 4.82 (d, *J* = 11.5 Hz, 1H), 4.78 (d, *J* = 12.0 Hz, 1H), 4.72 (d, *J* = 12.0 Hz, 1H), 4.67 (d, *J* = 12.0 Hz, 1H), 4.61−4.54 (m, 3H), 4.47−4.42 (m, 2H), 4.35 (d, *J* = 12.0 Hz, 1H), 4.30 (d, *J* = 12.0 Hz, 1H), 4.06−4.02 (m, 2H), 3.97 (dd, *J* = 10.0, 2.5 Hz, 1H), 3.94−3.90 (m, 3H), 3.69 (dd, *J* = 10.5, 7.0 Hz, 1H), 3.51−3.45 (m, 2H), 2.52 (t, *J* = 7.8 Hz, 2H), 2.30 (s, 3H), 1.81 (quin, *J* = 7.3 Hz, 2H), 1.56 (quin, *J* = 7.5 Hz, 2H), 1.36−1.24 (m, 4H); ^{13}C NMR (125 MHz, $CDCl_3$) δ 139.6, 138.9, 138.9, 138.5, 138.4, 138.2, 129.1, 128.6, 128.5, 128.4, 128.1, 128.0, 128.0, 127.9, 127.8, 127.7, 127.6, 117.9, 98.8, 80.7, 79.1, 77.4, 76.8, 76.5, 75.2, 75.0, 74.9, 74.6, 73.6, 73.4, 73.3, 70.5, 70.0, 69.2, 68.0, 62.2, 52.5, 35.5, 31.6, 30.5, 28.9, 26.7, 21.2; LRMS (ESI$^+$) *m/z* calcd for $C_{68}H_{76}N_5O_8$ [M + H]$^+$: 1090.57; Found: 1090.77.

General Procedure for the *N*-acylation at sphingosine backbone for the preparation of 15-18

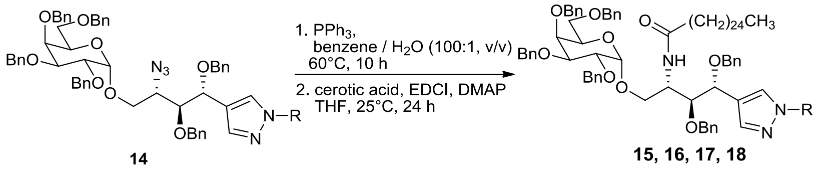

Compound **14** and PPh_3 (2 equiv.) were dissolved in benzene/H_2O co-solvent (100/1, 10 mM concentration). Then, the mixture was stirred at 60 °C for 10 h. After completion of reaction monitored by TLC, the reaction mixture was condensed under reduced pressure and re-dissolved in anhydrous THF (10 mM concentration). Carboxylic acid (1.5 equiv.), EDCI (3 equiv.), and DMAP (catalytic amount) were added to the corresponding mixture and stirred at room temperature for 24 h. After reaction completion, the resulting mixture was diluted with EtOAc and washed with brine. The combined organic layer was dried over anhydrous Na_2SO_4(s). The filtrate was condensed under reduced pressure and purified by silica-gel flash

column chromatography (EtOAc:Hex = 1:3, v/v) to provide the desired product **15–18** as a colorless oil.

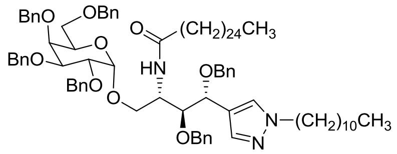

Compound **15a**; 55% yield; $[\alpha]_D^{25}$ -2.68 (c = 0.90, $CHCl_3$); ^1H NMR (500 MHz, $CDCl_3$) δ 7.53 (s, 1H), 7.41 (s, 1H), 7.37−7.35 (m, 2H), 7.33−7.21 (m, 28H), 6.16 (d, *J* = 9.5 Hz, 1H), 4.91 (m, 3H), 4.72 (q, *J* = 6.0 Hz, 2H), 4.63 (d, *J* = 11.5 Hz, 1H), 4.55 (d, *J* = 11.0 Hz, 2H), 4.49−4.45 (m, 3H), 4.36 (d, *J* = 12.0 Hz, 1H), 4.22 (d, *J* = 12.0 Hz, 1H), 4.10−4.06 (m, 1H), 4.05−4.01 (m, 3H), 3.95−3.91 (m, 2H), 3.89−3.86 (m, 3H), 3.55 (dd, *J* = 10.5, 3.5 Hz, 1H), 3.48 (dd, *J* = 9.3, 6.8 Hz, 1H), 3.35 (dd, *J* = 9.3, 6.3 Hz, 1H), 1.98−1.86 (m, 2H), 1.81 (quin, *J* = 7.0 Hz, 2H), 1.53−1.44 (m, 2H), 1.31−1.15 (m, 60H), 0.91−0.86 (m, 6H); ^{13}C NMR (75 MHz, $CDCl_3$) δ 173.0, 139.7, 138.8, 138.7, 138.6, 137.9, 129.1, 128.6, 128.6, 128.6, 128.5, 128.5, 128.5, 128.2, 128.1, 128.0, 127.9, 127.8, 127.7, 117.5, 99.7, 80.9, 79.0, 75.2, 74.9, 74.5, 73.7, 73.6, 73.1, 70.3, 70.1, 69.6, 69.4, 52.5, 50.2, 36.9, 32.1, 30.6, 29.9, 29.9, 29.8, 29.8, 29.7, 29.6, 29.6, 29.6, 29.5, 29.4, 26.9, 25.9, 24.7, 22.9, 14.3

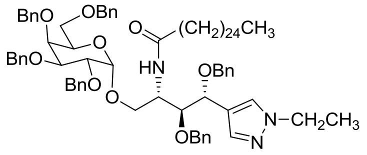

Compound **15b**; 80% yield; $[\alpha]_D^{25}$ -4.21 (c = 0.38, $CHCl_3$); ^1H NMR (500 MHz, $CDCl_3$) δ 7.54 (s, 1H), 7.44 (s, 1H), 7.37–7.21 (m, 30H), 4.91 (d, *J* = 11.5 Hz, 1H), 4.807–4.71 (m, 5H), 4.64–4.55 (m, 3H), 4.50–4.45 (m, 3H), 4.35 (d, *J* = 11.5 Hz, 1H), 4.23 (d, *J* = 12.0 Hz, 1H), 4.11 (q, *J* = 7.8 Hz, 2H), 4.08–4.01 (m, 2H), 3.99–3.92 (m, 2H), 3.90–3.86 (m, 3H), 3.53 (dd, *J* = 11.0, 3.5 Hz, 1H), 3.49 (dd, *J* = 9.5, 6.5 Hz, 1H), 3.34 (dd, *J* = 9.5, 6.0 Hz, 1H), 1.96–1.86 (m, 2H), 1.48 (quin, *J* = 7.0 Hz, 3H), 1.34–1.23 (m, 44H), 0.88 (t, *J* = 7.0 Hz, 3H); ^{13}C NMR (75 MHz, $CDCl_3$) δ 172.9, 139.7, 138.8, 138.6, 138.6, 137.8, 129.1, 128.6, 128.6, 128.5, 128.4, 128.2, 128.1, 128.1, 128.0, 127.9, 127.8, 127.6, 127.6, 117.6, 99.8, 80.8, 79.0, 75.3, 74.9, 74.9, 74.5, 73.7, 73.7, 73.1, 70.3, 70.2, 69.7, 69.4, 50.2, 47.1, 36.9, 32.1, 29.9, 29.9, 29.8, 29.7, 29.6, 29.6, 25.9, 22.9, 15.7, 14.3.

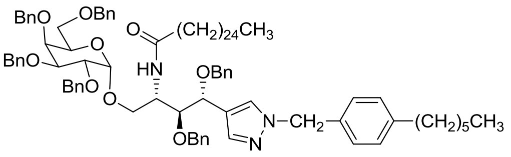

Compound **15c**; 78% yield; $[\alpha]_D^{25}$ –4.21 (c = 0.38, $CHCl_3$); ^{1}H NMR (500 MHz, $CDCl_3$) δ 7.58 (s, 1H), 7.43 (s, 1H), 7.36–7.18 (m, 30H), 1.12–7.08 (m, 4H), 6.07 (d, *J* = 9.0 Hz, 1H), 5.21 (d, *J* = 3.0 Hz, 2H), 4.91 (d, *J* = 11.5 Hz, 1H), 4.80–4.76 (m, 3H), 4.72–4.61 (m, 3H), 4.57–4.50 (m, 2H), 4.46–4.44 (m, 3H), 4.35 (d, *J* = 12.0 Hz, 1H), 4.20 (d, *J* = 12.0 Hz, 1H), 4.13–4.08 (m, 1H), 4.02 (dd, *J* = 10.0, 3.5 Hz, 1H), 3.94–3.85 (m, 5H), 3.55 (dd, *J* = 11.0, 3.5 Hz, 1H), 3.48 (dd, *J* = 9.0, 6.5 Hz, 1H), 3.35 (dd, *J* = 9.3, 6.3 Hz, 1H), 2.55 (t, *J* = 7.8 Hz), 1.95–1.83 (m, 2H), 1.56 (quin, *J* = 7.5 Hz, 2H), 1.46 (quin, *J* = 7.3 Hz, 2H), 1.33–1.23 (m, 50H), 0.89–0.85 (m, 6H); ^{13}C NMR (75 MHz, $CDCl_3$) δ172.9, 142.9, 140.7, 138.8, 138.7, 138.6, 137.9, 134.1, 130.1, 129.0, 128.6, 128.6, 128.5, 128.5, 128.4, 128.2, 128.1, 128.0, 128.0, 127.9, 127.8, 127.6, 118.4, 99.7, 81.0, 79.0, 75.0, 75.0, 74.9, 74.5, 73.7, 73.6, 73.1, 70.3, 70.2, 69.4, 56.0, 50.3, 36.9, 35.8, 32.1, 31.9, 31.6, 29.9, 29.9, 29.8, 29.7, 29.6, 29.2, 25.9, 22.9, 22.8, 14.3, 14.3.

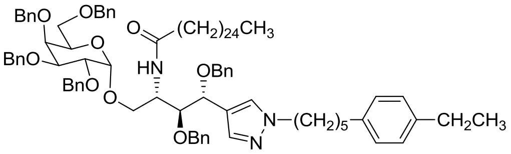

Compound **15d**; 65% yield; $[\alpha]_D^{25}$ –1.9 (c = 1.10, $CHCl_3$); ^{1}H NMR (500 MHz, $CDCl_3$) δ 7.54 (s, 1H), 7.41 (s, 1H), 7.37–7.23 (m, 30H), 7.09 (d, *J* = 7.5 Hz, 2H), 7.05 (d, *J* = 7.5 Hz, 2H), 6.16 (d, *J* = 9.0 Hz, 1H), 4.91 (d, *J* = 11.5 Hz, 1H), 4.80–4.71 (m, 5H), 4.63 (d, *J* = 12.0 Hz, 1H), 4.58–4.55 (m, 2H), 4.49–4.45 (m, 3H), 4.35 (d, *J* = 12.0 Hz, 1H), 4.22 (d, *J* = 12.0 Hz, 1H), 4.09–3.87 (m, 9H), 3.54 (dd, *J* = 11.0, 3.0 Hz, 1H), 3.48 (dd, *J* = 9.0, 6.5 Hz, 1H), 3.35 (dd, *J* = 9.3, 5.8 Hz, 1H), 2.60 (q, *J* = 7.5 Hz, 2H), 2.53 (t, *J* = 7.8 Hz, 2H), 1.96–1.82 (m, 4H), 1.65–1.57 (m, 4H), 1.48 (quin, *J* = 7.0 Hz, 2H), 1.28–1.24 (m, 49H), 0.88 (t, *J* = 6.8 Hz, 3H); LRMS(ESI$^+$) *m/z* calcd for $C_{94}H_{128}N_3O_9$ $[M+H]^+$: 1442.97; Found: 1442.93.

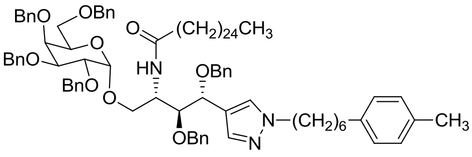

Compound **15e**; 75% yield; $[\alpha]_D^{25}$ –16.1 (c = 1.5, $CHCl_3$); ^{1}H NMR (500 MHz, $CDCl_3$) δ 7.54 (s, 1H), 7.41 (s, 1H), 7.37–7.21 (m, 30H), 7.06 (d, *J* = 8.0 Hz, 2H), 7.03 (d, *J* = 8.0 Hz, 2H), 6.17 (d, *J* = 9.0 Hz, 1H), 4.91 (d, *J* = 11.5 Hz, 1H), 4.80–4.71 (m, 5H), 4.62 (d, *J* = 12.0 Hz, 1H), 4.57–4.55 (m, 2H), 4.48–4.45 (m, 3H), 4.35 (d, *J* = 12.0 Hz, 1H), 4.22 (d, *J* = 12.0 Hz, 1H), 4.08–4.01 (m, 4H), 3.97–3.91 (m, 2H), 3.89–3.87 (m, 5H), 3.53 (dd, *J* = 11.0, 3.5 Hz, 1H), 3.48 (dd, *J* = 9.0, 7.0 Hz, 1H), 3.35 (dd, *J* = 9.0, 6.0 Hz, 1H), 2.52 (t, *J* = 7.8 Hz, 2H), 2.30 (s, 3H), 1.96–1.88 (m, 2H), 1.81 (quin, *J* = 7.1 Hz, 2H), 1.55 (quin, *J* = 7.4 Hz, 2H), 1.48 (quin, *J* = 7.1 Hz, 2H), 1.31–1.25 (m, 48H), 0.88 (t, *J* = 6.8 Hz, 3H); ^{13}C NMR (75 MHz, $CDCl_3$) δ 173.0, 139.7, 138.8, 138.7, 138.6, 137.9, 129.1, 128.6, 128.6, 128.6, 128.5, 128.5, 128.5, 128.2, 128.1, 128.0, 127.9, 127.8, 127.7, 117.6, 99.7, 81.0, 79.0, 77.4, 75.0, 74.9, 74.5, 73.7, 73.7, 73.1, 70.3, 70.2, 69.4, 52.4, 36.9, 35.5, 32.1, 31.6, 30.6, 29.9, 29.8, 29.7, 29.6, 29.6, 29.0, 26.7, 25.9, 22.9, 21.2, 14.3; LRMS(ESI$^+$) *m/z* calcd for $C_{94}H_{128}N_3O_9$ $[M+H]^+$: 1442.97; Found: 1443.11.

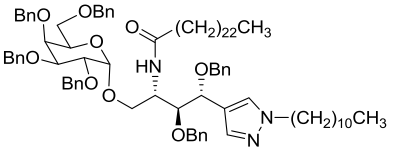

Compound **16a**; 58% yield; $[\alpha]_D^{25}$ -3.29 (c = 1.40, $CHCl_3$); ^{1}H NMR (500 MHz, $CDCl_3$) δ 7.54 (s, 1H), 7.41 (s, 1H), 7.37–7.22 (m, 30H), 6.13 (d, *J* = 9.0 Hz, 1H), 4.91 (d, *J* = 11.5 Hz, 1H), 4.80–4.76 (m, 3H), 6.72 (q, *J* = 5.5 Hz, 2H), 4.63 (d, *J* = 12.0 Hz, 1H), 4.56 (d, *J* = 11.5 Hz, 1H), 4.49–4.45 (m, 3H), 4.35 (d, *J* = 11.5 Hz, 1H), 4.22 (d, *J* = 12.0 Hz, 1H), 4.10–4.06 (m, 1H), 4.05–4.00 (m, 3H), 3.97–3.91 (m, 2H), 3.90–3.87 (m, 3H), 3.54 (dd, *J* = 10.8, 3.8 Hz, 1H), 3.47 (dd, *J* = 9.0, 6.5 Hz, 1H), 3.35 (dd, *J* = 9.3, 5.8 Hz, 1H), 1.97–1.86 (m, 2H), 1.81 (quin, *J* = 6.8 Hz, 2H), 1.48 (quin, *J* = 7.0 Hz, 2H), 1.30–1.1.16 (m, 56H), 0.89–0.86 (m, 6H).

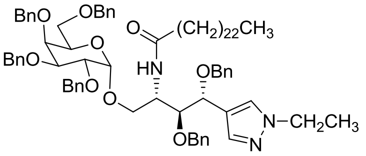

Compound **16b**; 59% yield; $[\alpha]_D^{25}$ -3.46 (c = 1.38, $CHCl_3$); ^{1}H NMR (500 MHz, $CDCl_3$) δ 7.54 (s, 1H), 7.44 (s, 1H), 7.35–7.23 (m, 30H), 6.16 (d, *J* = 9.5 Hz, 1H), 4.91 (d, *J* = 12.0 Hz, 1H), 4.81–4.71 (m, 5H), 4.64–4.55 (m, 3H), 4,50–4.45 (m, 3H), 4.35 (d, *J* = 12.0 Hz, 1H), 4.23 (d, *J* = 12.0 Hz, 1H), 4.09 (q, *J* = 7.3 Hz, 2H), 4.07–3.98 (m, 2H), 3.97–3.12 (m, 2H), 3.89–

3.87 (m, 3H), 3.53 (dd, *J* = 11.0, 3.5 Hz, 1H), 3.48 (dd, *J* = 9.3, 6.8 Hz, 1H), 3.34 (dd, *J* = 9.3, 5.8 Hz, 1H), 1.98–1.86 (m, 2H), 1.48 (quin, *J* = 7.0 Hz, 2H), 1.44 (t, *J* = 7.5 Hz, 3H), 1.33–1.16 (m, 40H), 0.88 (t, *J* = 6.8 Hz, 3H); ^{13}C NMR (75 MHz, $CDCl_3$) δ 173.0, 139.7, 138.8, 137.8, 129.2, 128.6, 128.6, 128.6, 128.5, 128.3, 128.1, 128.1, 128.0, 127.9, 127.8, 127.7, 127.6, 117.6, 99.8, 80.8, 79,0, 75.3, 74.9, 74.5, 73.7, 73.1, 70.4, 70.2, 69.7, 69.4, 50.2, 47.1, 37.5, 36.9, 32.1, 29.9, 29.9, 29.3, 29.7, 29.6, 25.9, 22.9, 15.7, 14.4.

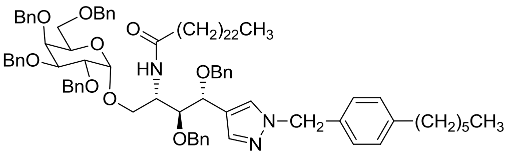

Compound **16c**; 82%; $[\alpha]_D^{25}$ –1.96 (c = 0.33, $CHCl_3$); ^{1}H NMR (500 MHz, $CDCl_3$) δ 7.58 (s, 1H), 7.43 (s, 1H), 7.34–7.35 (m, 2H), 7.33–7.18 (m, 28H), 7.12–7.09 (m, 4H), 5.21 (d, *J* = 3.0 Hz, 2H), 4.91 (d, *J* = 11.5 Hz, 1H), 4.80–4.76 (m, 3H), 4.72–4.61 (m, 3H), 4.57–4.50 (m, 2H), 4.47–4.44 (m, 3H), 4.35 (d, *J* = 12.0 Hz, 1H), 4.20 (d, *J* = 11.5 Hz, 1H), 4.14–4.09 (m, 1H), 4.02 (dd, *J* = 10.3, 3.8 Hz, 1H), 3.94–3.85 (m, 5H), 3.55 (dd, *J* = 10.8, 3.8 Hz, 1H), 3.48 (dd, *J* = 9.5, 6.5 Hz, 1H), 3.35 (dd, *J* = 9.3, 6.3 Hz, 1H), 2.55 (t, *J* = 7.8 Hz, 2H), 1.95–1.83 (m, 2H), 1.56 (quin, *J* = 7.5 Hz, 2H), 1.46 (quin, *J* = 7.3 Hz, 2H), 1.33–1.23 (m, 48H), 0.91–0.86 (m, 6H); ^{13}C NMR (75 MHz, $CDCl_3$) δ 172.9, 142.9,141.7 138.8, 138.7, 138.6, 137.9, 134.1, 130.9, 129.0, 128.6, 128.6, 128.5, 128.5, 128.5, 128.2, 128.1, 128.0, 128.0, 127.9, 127.8, 127.6, 118.4, 99.7, 81.0, 79.0, 75.0, 75.0, 74.9, 74.5, 73.7, 73.6, 73.1, 70.3, 70.2, 69.4, 55.9, 50.4, 35.8, 32.1, 31.9, 31.6, 29.9, 29.9, 29.7, 29.6, 29.6, 29.2, 25.9, 22.9, 22.8, 14.3, 14.3

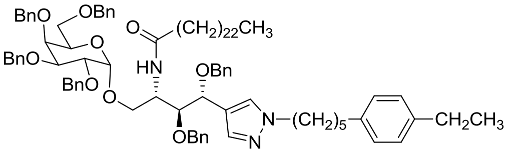

Compound **16d**; 81% yield; $[\alpha]_D^{25}$ –2.42 (c = 1.92, $CHCl_3$); ^{1}H NMR (500 MHz, $CDCl_3$) δ 7.54 (s, 1H), 7.41 (s, 1H), 7.37–7.35 (m, 2H), 7.33–7.22 (m, 28H), 7.10–7.04 (m, 4H), 4.91 (d, *J* = 11.5 Hz, 1H), 4.80–4.75 (m, 3H), 4.72 (d, *J* = 12.5 Hz, 2H), 4.63 (d, *J* = 11.5 Hz, 1H), 4.57 (d, *J* = 4.5 Hz, 1H), 4.55 (d, *J* = 3.0 Hz, 1H), 4.48–4.45 (m, 3H), 4.35 (d, *J* = 12.0 Hz, 1H), 4.22 (d, *J* = 11.5 Hz, 1H), 4.08–4.01 (m, 4H), 3.97–3.92 (m, 2H), 3.89–3.86 (m, 3H), 3.53 (dd, *J* = 11.0, 3.5 Hz, 1H), 3.48 (dd, *J* = 9.3, 6.8 Hz, 1H), 3.35 (dd, *J* = 9.5, 6.0 Hz, 1H), 2.60 (q, *J* = 7.8 Hz, 2H), 2.53 (t, *J* = 8.0 Hz, 2H), 1.98–1.82 (m, 4H), 1.60 (quin, *J* = 7.8 Hz, 2H), 1.48 (quin, *J* = 7.0 Hz, 2H), 1.33–1.19 (m, 45H), 0.88 (t, *J* = 7.0 Hz, 3H); ^{13}C NMR (75 MHz,

$CDCl_3$) δ 173.0, 141.7, 139.7, 138.8, 138.7, 138.6, 137.9, 129.8, 128.6, 128.6, 128.6, 128.5, 128.2, 128.1, 128.0, 128.0, 127.9, 127.8, 127.8, 127.7, 117.6, 99.7, 81.0, 79.0, 75.2, 75.0, 74.9, 74.5, 73.7, 73.7, 73.1, 70.3, 70.2, 69.5, 69.4, 52.4, 50.3, 36.9, 35.5, 32.1, 31.2, 30.5, 29.9, 29.8, 29.7, 29.6, 29.6, 28.6, 26.6, 25.9, 22.9, 15.8, 14.3.

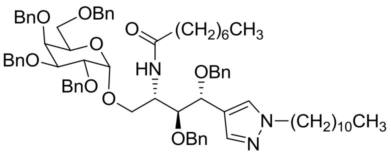

Compound **17a**; 69% yield; $[\alpha]_D^{25}$ -4.35 (c = 1.40, $CHCl_3$); ^{1}H NMR (500 MHz, $CDCl_3$) δ 7.54 (s, 1H), 7.41 (s, 1H), 7.37–7.22 (m, 30H), 6.13 (d, *J* = 9.5 Hz, 1H), 4.9. (d, *J* = 11.5 Hz, 1H), 4.80–4.77 (m, 3H), 4.73 (q, *J* = 5.8 Hz, 2H), 4.63 (*J* = 11.5 Hz, 1H), 4.56 (dd, *J* = 11.5, 1.5 Hz, 2H), 4.49–4.45 (m, 3H), 4.35 (d, *J* = 12.0 Hz, 1H), 4.22 (d, *J* = 12.0 Hz, 1H), 4.10–4.06 (m, 1H), 4.05–4.00 (m, 3H), 3.97–3.91 (m, 2H), 3.90–3.86 (m, 3H), 3.54 (dd, *J* = 11.0, 3.5 Hz, 1H), 3.48 (dd, *J* = 9.5, 6.5 Hz, 1H), 3.35 (dd, *J* = 9.5, 6.0 Hz, 1H), 1.98–1.86 (m, 2H), 1.81 (quin, *J* = 7.0 Hz, 2H), 1.48 (quin, *J* = 7.3 Hz, 2H), 1.28–1.23 (m, 24H), 0.88–0.85 (m, 6H).

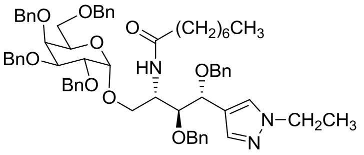

Compound **17b**; 84% yield; $[\alpha]_D^{25}$ -9.96 (c = 1.35, $CHCl_3$); ^{1}H NMR (500 MHz, $CDCl_3$) δ 7.54 (s, 1H), 7.44 (s, 1H), 7.37–7.23 (m, 30H), 6.22 (d, *J* = 9.0 Hz, 1H), 4.91 (d, *J* = 11.5 Hz, 1H), 4.80–4.71 (m, 5H), 4.64–4.55 (m, 3H), 4.50–4.45 (m, 3H), 4.35 (d, *J* = 12.0 Hz, 1H), 4.23 (d, *J* = 12.0 Hz, 1H), 4.12 (q, *J* = 7.3 Hz, 2H), 4.03–4.01 (m, 2H), 3.97–3.92 (m, 2H), 3.89–3.87 (m, 3H), 3.53 (dd, *J* = 11.0, 3.0 Hz, 1H), 3.48 (dd, *J* = 9.3, 6.8 Hz, 1H), 3.34 (dd, *J* = 9.3, 5.8 Hz, 1H), 2.34 (t, H = 7.5 Hz, 2H), 1.98–1.89 (m, 2H), 1.63 (quin, *J* = 7.3 Hz, 2H), 1.49 (quin, *J* = 7.5 Hz, 2H), 1.44 (t, *J* = 7.5 Hz, 3H), 1.31–1.23 (m, 4H); ^{13}C NMR (75 MHz, $CDCl_3$) δ 173.1,139.7, 138.8, 138.8, 138.6, 138.6, 137.9, 128.6, 128.6, 128.5, 128.5, 128.5, 128.2, 128.1, 128.0, 127.9, 127.8, 127.7, 127.6, 117.6, 99.7, 80.9, 79.0, 75.3, 75.0, 74.5, 73.7, 73.1, 70.4, 70.2, 69.6, 69.4, 50.3, 47.1, 36.9, 32.0, 31.8, 29.9, 29.5, 29.3, 29.1, 25.9, 25.0, 22.9, 22.8, 15.7, 14.3.

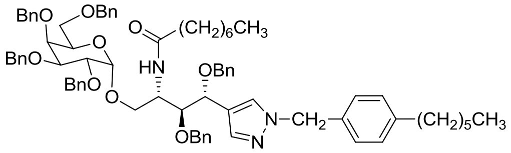

Compound **17c**; 85% yield; $[\alpha]_D^{25}$ –2.51 (c = 1.40, $CHCl_3$); ^1H NMR (500 MHz, $CDCl_3$) δ 7.58 (s, 1H), 7.43 (s, 1H), 7.36–7.35 (m, 2H), 7.33–7.18 (m, 28H), 7.12–7.08 (m, 4H), 5.21 (d, *J* = 3.0 Hz, 2H), 4.91 (d, *J* = 11.5 Hz, 1H), 4.78 (q, *J* = 5.8 Hz, 3H), 4.72–4.61 (m, 3H), 4.57–4.50 (m, 2H), 4.46–4.44 (m, 3H), 4.35 (d, *J* = 12.0 Hz, 1H), 4.20 (d, *J* = 12.0 Hz, 1H), 4.12–4.08 (m, 1H), 4.02 (dd, *J* = 10.5, 3.5 Hz, 1H), 3.94–3.85 (m, 5H), 3.55 (dd, *J* = 11.0, 3.5 Hz, 1H), 3.48 (dd, *J* = 9.0, 6.5 Hz, 1H), 3.35 (dd, *J* = 9.0, 6.0 Hz, 1H), 2.55 (t, *J* = 7.8 Hz, 2H), 1.95–1.83 (m, 2H), 1.56 (quin, *J* = 7.5 Hz, 2H), 1.47 (quin, *J* = 7.3 Hz, 2H), 1.33–1.16 (m, 14H), 0.91–0.8 (m, 6H); ^{13}C NMR (75 MHz, $CDCl_3$) δ 172.9, 142.9, 138.8, 138.7, 137.9, 134.1, 130.1, 129.0, 128.6, 128.6, 128.6, 128.5, 128.5, 128.2, 128.1, 128.1, 128.0, 127.9, 127.8, 127.8, 127.6, 118.5, 99.7, 81.0, 79.1, 75.0, 74.9, 74.5, 73.7, 73.6, 73.2, 70.3, 70.2, 69.4, 56.0, 50.3, 36.9, 35.8, 32.0, 31.9, 31.6, 29.9, 29.5, 29.3, 29.2, 25.9, 22.9, 22.8, 14.3.

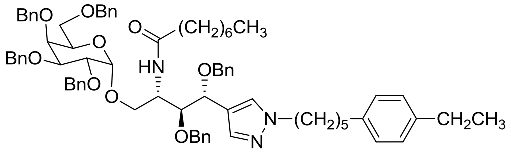

Compound **17d**; 91% yield; $[\alpha]_D^{25}$ +9.42 (c = 1.43, $CHCl_3$); ^1H NMR (500 MHz, $CDCl_3$) δ 7.56 (s, 1H), 7.43 (s, 1H), 7.39–7.37 (m, 2H), 7.35 (m, 28H), 7.11–7.06 (m, 4H), 6.19 (d, *J* = 9.0 Hz, 1H), 4.93 (d, *J* = 11.5 Hz, 1H), 4.82–4.77 (m, 3H), 4.75–4.72 (m, 2H), 4.64 (d, *J* = 11.5 Hz, 1H), 4.59 (d, *J* = 6.0 Hz, 1H), 4.47 (d, *J* = 8.5 Hz, 1H), 4.50–4.46 (m, 3H), 4.37 (d, *J* = 12.0 Hz, 1H), 4.23 (d, *J* = 11.5 Hz, 1H), 4.08–4.03 (m, 4H), 3.99–3.93 (m, 2H), 3.91–3.87 (m, 3H), 3.55 (dd, *J* = 10.5, 3.5 Hz, 1H), 3.50 (dd, *J* = 9.3, 6.8 Hz, 1H), 3.36 (dd, *J* = 9.0, 6.0 Hz, 1H), 2.61 (q, *J* = 7.8 Hz, 2H), 2.55 (t, *J* = 7.5 Hz, 2H), 2.00–1.84 (m, 4H), 1.62 (quin, *J* = 8.0 Hz, 2H), 1.50 (quin, *J* = 7.5 Hz, 2H), 1.36–1.19 (m, 13H), 0.88 (t, *J* = 7.3 Hz, 3H); ^{13}C NMR (75 MHz, $CDCl_3$) δ 172.9, 141.7, 139.7, 138.8, 138.7, 138.6, 137.8, 129.8, 128.6, 128.6, 128.5, 128.5, 128.2, 128.1, 128.1, 128.0, 128.0, 127.9, 127.8, 127.7, 117.5, 99.8, 80.9, 79.0, 75.2, 74.9, 74.5, 73.7, 73.7, 73.1, 70.3, 70.2, 69.6, 69.4, 52.4, 50.2, 36.9, 35.5, 32.0, 31.3, 30.5, 29.9, 29.5, 29.3, 28.6, 26.6, 25.9, 22.9, 15.9, 14.3.

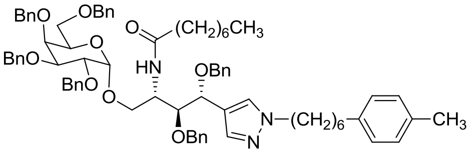

Compound **17e**; 79% yield; $[\alpha]_D^{25}$ +4.62 (c = 1.0, $CHCl_3$); ^{1}H NMR (500 MHz, $CDCl_3$) δ 7.53 (s, 1H), 7.40–7.19 (m, 31H), 7.07–7.02 (m, 4H), 6.16 (d, *J* = 9.5 Hz, 1H), 4.91 (d, *J* = 12.0 Hz, 1H), 4.78–4.67 (m, 5H), 4.62 (d, *J* = 12.0 Hz, 1H), 4.55 (dd, *J* = 11.3, 1.3 Hz, 2H), 4.48–4.45 (m, 3H), 4.37–4.32 (m, 1H), 4.22 (d, *J* = 12.0 Hz, 1H), 4.10–4.01 (m, 4H), 3.96–3.85 (m, 5H), 3.57 (dd, *J* = 10.8, 3.8 Hz, 1H), 3.48 (dd, *J* = 9.5, 6.5 Hz, 1H), 3.36 (dd, *J* = 9.5, 6.0 Hz, 1H), 2.52 (t, *J* = 7.8 Hz, 2H), 2.30 (s, 3H), 1.98–1.78 (m, 4H), 1.55 (quin, *J* = 7.5 H, 2H), 1.48 (quin, *J* = 7.5 Hz, 2H), 1.35–1.20 (m, 12H), 0.86 (t, *J* = 7.0 Hz, 3H).

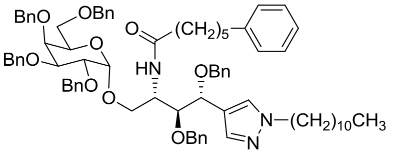

Compound **18a**; 61% yield; $[\alpha]_D^{25}$ –2.10 (c = 1.16, $CHCl_3$); ^{1}H NMR (500 MHz, $CDCl_3$) δ 7.53 (s, 1H), 7.40 (s, 1H), 7.35–7.22 (m, 32H), 7.17–7.13 (m, 3H), 1.91 (d, *J* = 11.5 Hz, 1H), 4.78–4.76 (m, 3H), 4.70 (d, *J* = 11.0 Hz, 2H), 4.62 (d, *J* = 11.5 Hz, 1H), 4.55 (dd, *J* = 11.3, 4.3 Hz, 2H), 4.48–4.53 (m, 3H), 4.34 (d, *J* = 12.0 Hz, 1H), 4.22 (d, *J* = 11.5 Hz, 1H), 4.11–4.06 (m, 1H), 4.05–4.01 (m, 3H), 3.95–3.86 (m, 5H), 3.54 (dd, *J* = 11.0, 3.0 Hz, 1H), 3.50–3.47 (m, 1H), 3.36 (dd, *J* = 8.8, 6.3 Hz, 1H), 2.56 (t, *J* = 7.8 Hz, 2H), 1.95–1.86 (m, 2H), 1.57 (quin, *J* = 7.5 Hz, 2H), 1.51 (quin, *J* = 7.5 Hz, 2H), 1.29–1.23 (m, 18H), 0.87 (t, *J* = 6.5 Hz, 3H).

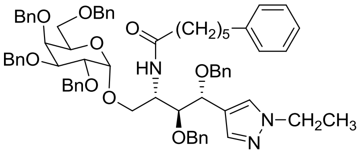

Compound **18b**; 75% yield; $[\alpha]_D^{25}$ +3.09 (c = 0.50, $CHCl_3$); ^{1}H NMR (500 MHz, $CDCl_3$) δ 7.54 (s, 1H), 7.44 (s, 1H), 7.36–7.22 (m, 29H), 7.18–7.13 (m, 4H), 6.16 (d, *J* = 9.0 Hz, 1H), 4.91 (d, *J* = 11.5 Hz, 1H), 4.79–4.70 (m, 5H), 4.62 (d, 11.5 Hz, 1H), 4.56 (t, *J* = 10.5 Hz, 2H), 4.49–4.43 (m, 3H), 4.33 (d, *J* = 12.0 Hz, 1H), 4.22 (d, *J* = 12.0 Hz, 1H), 4.11 (q, *J* = 7.3 Hz, 2H), 4.06–4.00 (m, 2H), 3.97 (dd, *J* = 11.0, 4.0 Hz, 1H), 3.92 (dd, *J* = 8.0, 3.5 Hz, 1H), 3.89–3.85 (m, 3H), 3.52 (dd, *J* = 11.0, 3.5 Hz, 1H), 3.48 (dd, *J* = 9.5, 7.0 Hz, 1H), 3.33 (dd, *J* = 9.3,

5.8 Hz, 1H), 2.56 (t, *J* = 7.8 Hz, 2H), 1.97–1.84 (m, 2H), 1.70–1.48 (m, 6H), 1.44 (t, *J* = 7.3 Hz, 3H).

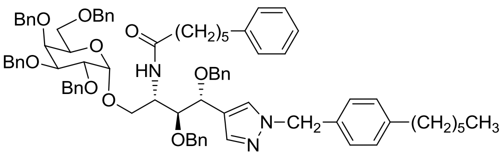

Compound **18c**; 82% yield; $[\alpha]_D^{25}$ –0.55 (c = 1.41, $CHCl_3$); ^{1}H NMR (500 MHz, $CDCl_3$) δ 7.58 (s, 1H), 7.42 (s, 1H), 7.35–7.34 (m, 2H), 7.32–7.14 (m, 28H), 7.11–7.01 (m, 4H), 6.06 (d, *J* = 9.0 Hz, 1H), 5.21 (d, *J* = 3.0 Hz, 2H), 4.90 (d, *J* = 11.5 Hz, 1H), 4.78–4.75 (m, 3H), 4.70 (d, *J* = 12.0 Hz, 1H), 4.63 (t, *J* = 12.0 Hz, 2H), 4.55 (d, *J* = 11.5 Hz, 1H), 4.49 (d, *J* = 11.0 Hz, 1H), 4.44 (dd, *J* = 11.0, 4.5 Hz, 3H), 4.33 (d, *J* = 12.0 Hz, 1H), 4.20 (d, *J* = 12.0 Hz, 1H), 4.16–4.11 (m, 1H), 4.01 (dd, *J* = 10.3, 3.3 Hz, 1H), 3.92–3.84 (m, 5H), 3.55 (dd, *J* = 10.8, 3.8 Hz, 1H), 3.48 (dd, *J* = 9.5, 3.5 Hz, 1H), 3.35 (dd, *J* = 9.5, 6.0 Hz, 1H), 2.55 (t, *J* = 8.0 Hz, 4H), 1.93–1.80 (m, 2H), 1.58–1.46 (m, 4H), 1.33–1.20 (m, 10H), 0.87 (t, *J* = 7.0 Hz, 3H).

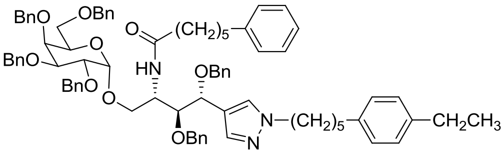

Compound **18d**; 82% yield; $[\alpha]_D^{25}$ –1.95 (c = 1.41, $CHCl_3$); ^{1}H NMR (500 MHz, $CDCl_3$) δ 7.54 (s, 1H), 7.40 (s, 1H), 7.35–7.34 (m, 2H), 7.32–7.21 (m, 30H), 7.17–7.14 (m, 3H), 7.09–7.04 (m, 4H), 4.90 (d, *J* = 11.5 Hz, 1H), 4.78–7.76 (m, 3H), 4.71 (t, *J* = 10.5 Hz, 2H), 4.62 (d, *J* = 11.5 Hz, 1H), 4.55 (d, *J* = 11.5 Hz, 2H), 4.48–4.43 (m, 3H), 4.34 (d, *J* = 12.0 Hz, 1H), 4.21 (d, *J* = 12.0, 1H), 4.09–3.96 (m, 4H), 3.95–3.85 (m, 5H), 3.53 (dd, *J* = 10.8, 3.8 Hz, 1H), 3.48 (dd, *J* = 9.5, 6.5 Hz, 1H), 3.35 (dd, *J* = 9.5, 6.0 Hz, 1H), 2.62–2.51 (m, 6H), 1.96–1.82 (m, 4H), 1.63–1.48 (m, 6H), 1.34–1.24 (m, 4H), 1.21 (t, *J* = 7.5 Hz, 3H); ^{13}C NMR (75 MHz, $CDCl_3$) δ 172.8, 142.7, 141.7, 139.7, 138.8, 138.7, 138.6, 137.9, 129.8, 128.6, 128.6, 128.5, 128.5, 128.2, 128.1, 128.0, 128.0, 127.9, 127.8, 127.8, 127.7, 125.9, 117.6, 99.8, 81.0, 89.0, 75.1, 74.9, 74.9, 74.5, 79.7, 73.1, 70.3, 70.2, 69.4, 50.4, 50.3, 36.7, 36.0, 35.5, 31.4, 31.3, 30.5, 29.9, 29.2, 28.6, 26.6, 25.7, 15.9.

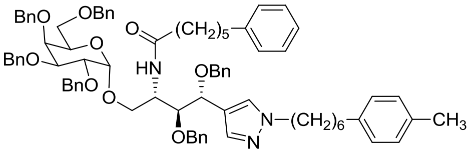

Compound **18e**; 76% yield; $[\alpha]_D^{25}$ -4.17 (c = 1.0, $CHCl_3$); ^1H NMR (500 MHz, $CDCl_3$) δ 7.53 (s, 1H), 7.39 (s, 1H), 7.35–7.21 (m, 32H), 7.17–7.13 (m, 3H), 7.07–7.01 (m, 4H), 6.14 (d, *J* = 9.0 Hz, 1H), 4.90 (d, *J* = 11.5 Hz, 1H), 4.78–4.76 (m, 3H), 4.73–4.69 (m, 2H), 4.62 (d, *J* = 11.5 Hz, 1H), 4.54 (dd, *J* = 11.3, 2.8 Hz, 2H), 4.48–4.43 (m, 3H), 4.34 (d, *J* = 12.0 Hz, 1H), 4.21 (d, *J* = 12.0 Hz, 1H), 4.10–4.00 (m, 4H), 3.95–3.95 (m, 5H), 3.53 (dd, *J* = 11.0, 3.5 Hz, 1H), 3.48 (dd, *J* = 9.5, 6.5 Hz, 1H), 3.35 (dd, *J* = 9.5, 6.0 Hz, 1H), 2.57–2.50 (m, 4H), 2.30 (s, 3H), 1.96–1.78 (m, 4H), 1.67–1.48 (m, 6H), 1.39–1.21 (m, 6H).

General procedure of click reaction for the preparation of 19

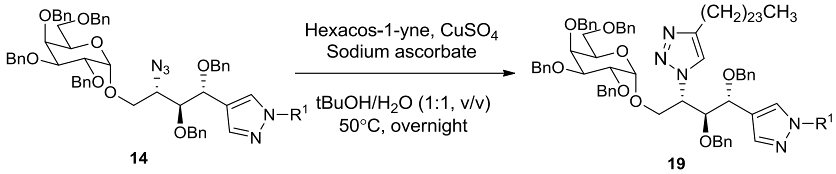

14 19

To a stirred solution of **19** (1.0 equiv.) in tBuOH/H_2O (1:1, v/v), hexacos-1-yne (1.5 equiv.), $CuSO_4$ (0.15 equiv.), and sodium ascorbate (0.6 equiv.) were added. The mixture was stirred at 50°C for overnight. After completion of reaction monitored by TLC, solvent was evaporated under reduced pressure and the resulting mixture was diluted with EtOAc and washed with brine. The combined organic layer was dried over anhydrous Na_2SO_4(s). The filtrate was condensed under reduced pressure and purified by silica-gel flash column chromatography (EtOAc:Hex = 1:4, v/v) to provide the desired product **19**.

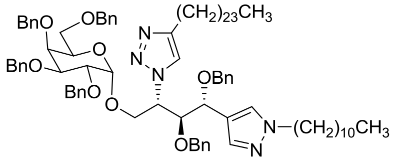

Compound **19a**; 75% yield; $[\alpha]_D^{25}$ -8.39 (c = 0.65, $CHCl_3$); ^1H NMR (500 MHz, $CDCl_3$) δ 7.74 (s, 1H), 7.38–7.20 (m, 30H), 7.15–7.13 (m, 2H), 4.89 (d, *J* = 11.5 Hz, 1H), 4.83–4.80 (m, 2H), 4.76 (d, 12.0 Hz, 1H), 4.69 (t, *J* = 11.5 Hz, 2H), 4.58 (d, *J* = 11.0 Hz, 1H), 4.53 (dd, *J* = 11.8, 4.8 Hz, 2H), 4.42–4.38 (m, 3H), 4.33–4.30 (m, 2H), 4.16 (d, *J* = 11.5 Hz, 1H), 4.11 (dd, *J* = 11.0, 6.5 Hz, 1H), 4.04 (t, *J* = 7.5 Hz, 2H), 4.01–3.98 (m, 2H), 3.95 (dd, *J* = 11.3, 2.8 Hz), 3.88 (d, *J* = 2.0 Hz, 1H), 3.81 (dd, *J* = 10.3, 2.8 Hz, 1H), 3.61 (t, *J* = 6.3 Hz, 1H), 3.45–3.38 (m, 2H), 2.62–2.51 (m, 2H), 1.81 (quin, *J* = 7.0 Hz, 2H), 1.55 (quin, *J* = 7.5 Hz, 2H), 1.33–1.23 (m, 58H), 0.89–0.83 (m, 6H); ^{13}C NMR (75 MHz, $CDCl_3$) δ 148.4, 139.6, 138.8, 138.7, 138.6, 138.3, 138.1, 129.5, 128.6, 128.5, 128.5, 128.4, 128.4, 128.0, 127.9,

127.8, 127.7, 127.6, 121.4, 117.2, 98.8, 81.1, 79.1, 76.3, 75.0, 74.9, 74.1, 73.6, 73.4, 73.0, 70.3, 69.9, 68.9, 66.6, 61.9, 52.6, 32.1, 32.1, 30.6, 29.9, 29.8, 29.7, 29.6, 29.5, 29.4, 26.9, 26.0, 22.9, 14.4.

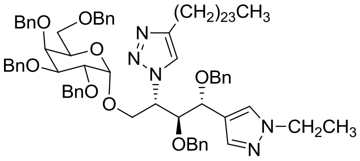

Compound **19b**; 88% yield; $[\alpha]_D^{25}$ +9.21 (c = 1.0, $CHCl_3$); ^{1}H NMR (500 MHz, $CDCl_3$) δ 7.44 (s, 1H), 7.37 (s, 1H), 7.35–7.21 (m, 31H), 7.16–7.14 (m, 2H), 4.89 (d, *J* = 11.5 Hz, 1H), 4.80–4.77 (m, 2H), 4.74–4.66 (m, 3H), 4.61 (d, *J* = 11.0 Hz, 1H), 4.53 (dd, *J* = 11.5, 5.0 Hz, 2H), 4.44–4.39 (m, 3H), 4.33–4.31 (m, 2H), 4.17 (d, *J* = 12.0 Hz, 1H), 4.11 (q, *J* = 7.0 Hz, 3H), 4.00–3.98 (m, 2H), 3.96 (dd, *J* = 11.3, 2.8 Hz, 1H), 3.88 (d, *J* = 2.0 Hz, 1H), 3.81 (dd, *J* = 10.0, 2.5 Hz, 1H), 3.62 (t, *J* = 6.5 Hz, 1H), 3.46–3.38 (m, 2H), 2.62–2.51 (m, 2H), 1.55 (quin, *J* = 7.5 Hz, 2H), 1.44 (t, *J* = 7.5 Hz, 3H), 1.30–1.23 (m, 42H), 0.88 (t, *J* = 6.8 Hz, 3H); ^{13}C NMR (75 MHz, $CDCl_3$) δ148.5, 139.6, 138.8, 138.6, 138.3, 138.2, 128.9, 128.6, 128.5, 128.5, 128.4, 128.4, 128.1, 128.0, 127.9, 127.8, 127.7, 127.6, 121.4, 117.3, 98.8, 81.0, 79.2, 76.3, 75.0, 74.9, 74.2, 73.6, 73.4, 73.0, 70.4, 69.9, 68.9, 66.7, 61.8, 47.2, 32.1, 29.9, 29.8, 29.7, 29.6, 26.0, 22.9, 15.7, 14.3.

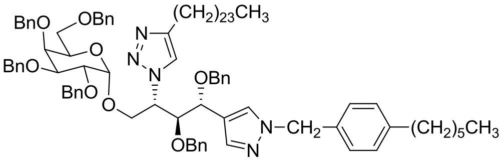

Compound **19c**; 60% yield; $[\alpha]_D^{25}$ +9.32 (c = 1.6, $CHCl_3$); ^{1}H NMR (500 MHz, $CDCl_3$) δ 7.49 (s, 1H), 7.36–7.76 (m, 29H), 7.10–7.07 (m, 6H), 5.20 (s, 2H), 4.89 (d, *J* = 11.0 Hz, 1H), 4.86–4.83 (m, 1H), 4.80 (d, *J* = 3.0 Hz, 1H), 4.76–7.65 (m, 3H), 4.53 (d, *J* = 11.5 Hz, 2H), 4.48 (d, *J* = 10.4 Hz, 1H), 4.41–4.26 (m, 5H), 4.16–4.09 (m, 2H), 4.00–4.39 (m, 3H), 3.87 (s, 1H), 3.80 (dd, *J* = 10.0, 2.0 Hz, 1H), 3.61 (t, *J* = 6.3 Hz, 1H), 3.42 (m, 2H), 2.57–2.53 (m, 4H), 1.59–1.51 (m, 4H), 1.27–1.23 (m, 48H), 0.89–0.86 (m, 6H); ^{13}C NMR (75 MHz, $CDCl_3$) 148.4, 143.1, 139.9, 138.8, 138.6, 138.2, 138.0, 133.7, 129.8, 129.1, 128.6, 128.5, 128.4, 128.4, 128.0, 127.9, 127.8, 127.7, 127.6, 121.4, 118.2, 98.8, 81.3, 79.1, 76.4, 75.0, 74.9, 74.0, 73.6, 73.4, 73.0, 70.4, 69.9, 69.0, 66.6, 61.9, 60.6, 56.1, 53.6, 35.8, 32.1, 31.9, 31.6, 29.9, 29.7, 29.7, 29.6, 29.2, 26.0, 22.9, 22.8, 21.3, 14.4, 14.3, 14.3.

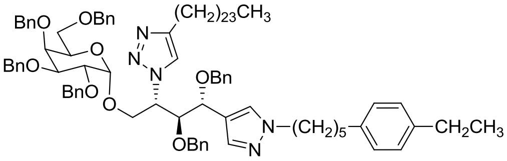

Compound **19d**; 72% yield; $[\alpha]_D^{25}$ +6.73 (c = 1.85, $CHCl_3$); ^{1}H NMR (500 MHz, $CDCl_3$) δ 7.44 (s, 1H), 7.37–7.20 (m, 30H), 7.15–7.13 (m, 2H), 7.09–7.04 (m, 4H), 4.88 (d, *J* = 11.5 Hz, 1H), 4.81–4.79 (m, 2H), 4.75 (d, *J* = 11.5 Hz, 1H), 4.71–4.66 (m, 2H), 4.59 (d, *J* = 11.0 Hz, 1H), 4.53 (dd, *J* = 1.5, 6.0 Hz, 2H), 4.42–4.38 (m, 3H), 4.33–4.30 (m, 2H), 4,16 (d, *J* = 12.0 Hz, 1H), 4.11 (dd, *J* = 11.3, 6.8 Hz, 1H), 4.04–3.98 (m, 4H), 3.95 (dd, *J* = 10.5, 3.5 Hz, 1H), 3.87 (d, *J* = 1.5 Hz, 1H), 3.80 (dd, *J* = 10.3, 2.8 Hz, 1H), 3.62 (t, *J* = 6.0 Hz, 1H), 3.46–3.38 (m, 2H), 2.62–2.51 (m, 6H), 1.85 (quin, *J* = 7.5 Hz, 2H), 1.64–1.52 (m, 4H), 1.34–1.19 (m, 47H), 0.88 (t, *J* = 7.0 Hz, 3H); ^{13}C NMR (75 MHz, $CDCl_3$) 148.5, 141.8, 139.6, 138.8, 138.6, 138.3, 138.2, 129.6, 128.6, 128.5, 128.5, 128.4, 128.4, 128.0, 128.0, 127.9, 127.8, 127.7, 127.6, 121.6, 117.3, 98.8, 81.1, 79.2, 76.4, 75.0, 74.9, 74.1, 73.6, 73.4, 73.0, 70.4, 69.9, 68.9, 66.6, 61.9, 61.9, 52.5, 35.5, 32.1, 31.2, 30.4, 29.9, 29.8, 29.7, 29.6, 28.6, 26.5, 26.0, 22.9, 15.9, 14.3.

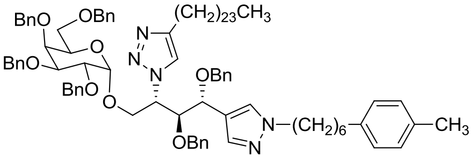

Compound **19e**; 56% yield; $[\alpha]_D^{25}$ +6.75 (c = 1.0, $CHCl_3$); ^{1}H NMR (500 MHz, $CDCl_3$) δ 7.43 (s, 1H), 7.38–7.20 (m, 30H), 7.14–7.13 (m, 2H), 7.07–7.01 (m, 4H), 4.89 (d, *J* = 11.0 Hz, 1H), 4.81–4.79 (m, 2H), 4.77–4.66 (m, 3H), 4.58 (d, *J* = 11.0 Hz, 1H), 4.53 (dd, *J* = 11.8, 5.3 Hz, 2H), 4.42–4.38 (m, 3H), 4.31 (dd, *J* = 11.3, 3.8 Hz, 2H), 4.16 (d, *J* = 11.5 Hz, 1H), 4.11 (dd, *J* = 11.3, 6.8 Hz, 1H), 4.04–3.98 (m, 4H), 3.95 (dd, *J* = 11.0, 2.0 H, 1H), 3.61 (t, *J* = 6.5 Hz, 1H), 3.46–3.38 (m, 2H), 2.60–2.51 (m, 4H), 2.30 (s, 3H), 1.81 (quin, *J* = 7.3 Hz, 2H), 1.56–1.52 (m, 4H), 1.30–1.23 (m, 49H), 0.88 (t, *J* = 6.5 Hz, 3H).

General Procedure for the benzyl deprotection via hydrogenation for final compounds 1–5

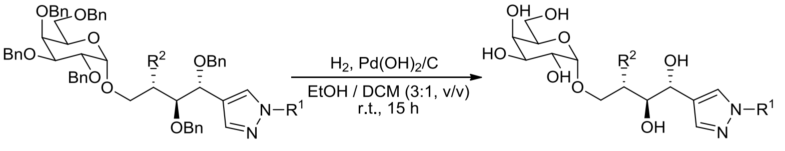

15, 16, 17, 18, 19 **1, 2, 3, 4, 5**

Compound **15**–**19** and $Pd(OH)_2/C$ (equal wt. quantity to starting material, 20 wt. % loading) were dissolved in EtOH/DCM co-solvent (1 mM concentration). Then, the mixture was stirred under H_2 environment at atmospheric pressure for 15 h. After completion of reaction monitored by TLC, the catalyst was removed by filtration through 0.45 μm PTFE syringe filter and washed with MeOH/DCM (1:1, v/v) solution. The filtrate was concentrated under reduced pressure to provide the desired product **1**–**5** as an amorphous white solid.

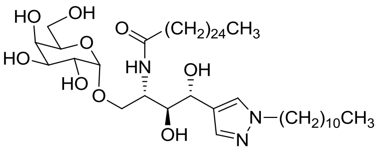

Compound **1a**; 54% yield; $[\alpha]_D^{25}$ +41.9 (c = 0.56, $CHCl_3$/MeOH = 1:1); ^{1}H NMR (500 MHz, CD_3OD; reference peak : CD_3OD at 3.31 ppm) δ 7.61 (s, 1H), 7.59 (s, 1H), 4.93 (d, *J* = 4.0 Hz, 1H), 4.69 (d, *J* = 4.5 Hz, 1H), 4.11 (t, *J* = 7.2 Hz, 2H), 4.05 (m, 1H), 3.94 (d, *J* = 2.5 Hz, 1H), 3.88–3.81 (m, 4H), 3.78–3.72 (m, 4H), 2.18 (t, *J* = 7.5 Hz, 2H), 1.86–1.82 (m, 2H), 1.65–1.59 (m, 2H), 1.35–1.22 (m, 60H), 0.85 (t, *J* = 7.0 Hz, 6H).

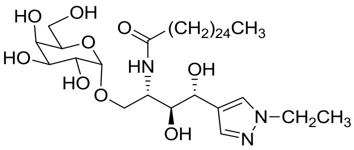

Compound **1b**; 59% yield; $[\alpha]_D^{25}$ +37.6 (c = 1.0, $CHCl_3$/MeOH = 1:1); ^{1}H NMR (500 MHz, $CDCl_3/CD_3OD$ = 1:1; reference peak : CD_3OD at 3.31 ppm) δ 7.69 (d, *J* = 9.0 Hz, 1H), 7.51 (s, 1H), 7.48 (s, 1H), 4.89 (d, *J* = 3.5 Hz, 1H), 4.11 (q, *J* = 7.3 Hz, 2H), 4.04 (m, 1H), 3.91 (d, *J* = 1.5 Hz, 1H), 3.85–3.70 (m, 8H), 2.18 (t, *J* = 7.5 Hz, 2H), 1.60–1.57 (m, 2H), 1.43 (t, *J* = 7.3 Hz, 3H), 1.31–1.24 (m, 44H), 0.85 (t, *J* = 6.8 Hz, 3H); ^{13}C NMR (75 MHz, $CDCl_3$) δ 175.4, 138.7, 128.8, 122.3, 100.6, 78.5, 74.8, 71.7, 71.0, 70.5, 69.7, 67.9, 62.4, 51.3, 47.4, 37.1, 32.6, 30.4, 30.3, 30.1, 30.1, 26.6, 23.3, 20.7, 15.9, 14.5; HRMS (FAB+) *m/z* calcd for $C_{41}H_{78}N_3O_9$ $[M+H]^+$: 756.5738; Found: 756.5742.

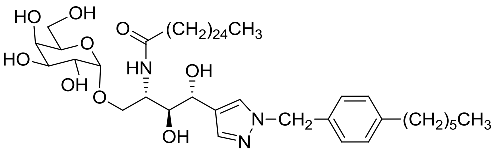

Compound **1c**; 78% yield; $[\alpha]_D^{25}$ +34.1 (c = 1.1, $CHCl_3$/MeOH = 1:1); ^{1}H NMR (500 MHz, $CDCl_3/CD_3OD$ = 1:1; reference peak : CD_3OD at 3.31 ppm) δ 7.58 (d, *J* = 8.5 Hz, 1H), 7.52

(s, 1H), 7.50 (s, 1H), 7.11 (s, 4H), 5.20 (s, 2H), 4.89 (d, *J* = 3.5 Hz, 1H), 4.64 (m, 1H), 4.06 (m, 1H), 3.90 (d, *J* = 2.5 Hz, 1H), 3.84-3.76 (m, 4H), 3.74–3.67 (m, 4H), 2.55 (t, *J* = 7.5 Hz, 2H), 2.15 (m, 2H), 1.56-1.52 (m, 4H), 1.26–1.24 (m, 50H), 0.87–0.84 (m, 6H); ^{13}C NMR (75 MHz, $CDCl_3$) δ 175.3, 143.6, 139.0, 134.3, 129.6, 129.4, 128.4, 123.0, 100.5, 71.6, 70.9, 70.5, 69.7, 67.8, 62.4, 56.2, 51.2, 37.0, 36.2, 32.6, 32.4, 32.1, 30.3, 30.1, 29.6, 26.6, 23.3, 23.2, 14.5.

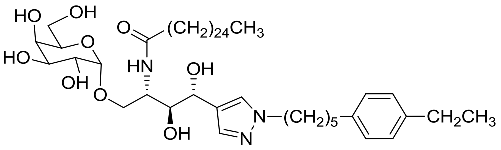

Compound **1d**; 95% yield; $[\alpha]_D^{25}$ +42.14 (c = 0.70, $CHCl_3$); ^{1}H NMR (500 MHz, $CDCl_3$) δ 7.50 (s, 1H), 7.49 (s, 1H), 7.06 (d, *J* = 7.5 Hz, 2H), 7.04 (d, *J* = 8.0 Hz, 2H), 4.89 (d, *J* = 4.0 Hz, 1H), 4.66 (d, *J* = 6.0 Hz, 1H), 4.07–4.01 (m, 3H), 3.90 (d, *J* = 3.0 Hz, 1H), 3.84–3.67 (m, 8H), 2.60–2.52 (m, 4H), 2.18 (t, *J* = 7.7 Hz, 2H), 1.84 (quin, *J* = 7.5 Hz, 2H), 1.64–1.58 (m, 5H), 1.33–1.24 (m, 46H), 1.19 (t, *J* = 7.7 Hz, 3H), 0.86 (t, *J* = 7.0 Hz, 3H); HRMS (FAB+) *m/z* calcd for $C_{52}H_{92}N_3O_9$ $[M+H]^+$: 902.6834; Found: 902.6845.

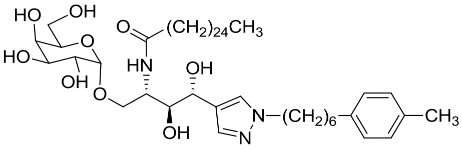

Compound **1e**; 96% yield; $[\alpha]_D^{25}$ +38.5 (c = 0.85, $CHCl_3$/MeOH = 1:1); ^{1}H NMR (500 MHz, $CDCl_3$/CD_3OD = 1:1; reference peak : CD_3OD at 3.31 ppm) δ 7.61 (s, 1H), 7.57 (s, 1H), 7.03 (d, *J* = 8.0 Hz, 2H), 7.00 (d, *J* = 8.0 Hz, 2H), 4.89 (d, *J* = 3.5 Hz, 1H), 4.13−4.09 (m, 2H), 4.00−3.98 (m, 1H), 3.90 (d, *J* = 3.5 Hz, 1H), 3.83−3.61(m, 8H), 2.52 (t, *J* = 7.5 Hz, 2H), 1.60−1.57 (m, 2H), 2.28 (s, 3H), 2.24−2.17 (m, 2H), 1.82 (quin, *J* = 7.1 Hz, 2H), 1.60−1.54 (m, 4H), 1.32−1.24(m, 48H), 0.85 (t, *J* = 7.0 Hz, 3H); ^{13}C NMR (75 MHz, $CDCl_3$) δ 175.4, 138.7, 128.8, 122.3, 100.6, 78.5, 74.8, 71.7, 71.0, 70.5, 69.7, 67.9, 62.4, 51.3, 47.4, 37.1, 32.6, 30.4, 30.3, 30.1, 30.1, 26.6, 23.3, 20.7, 15.9, 14.5; HRMS (FAB+) *m/z* calcd for $C_{52}H_{92}N_3O_9$ $[M+H]^+$: 902.6834; Found: 902.6827.

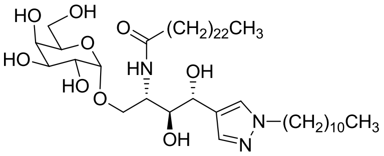

Compound **2a**; 95% yield; $[\alpha]_D^{25}$ +49.1 (c = 0.77, $CHCl_3$/MeOH = 1:1); ^{1}H NMR (500 MHz, CD_3OD; reference peak : CD_3OD at 3.31 ppm) δ 7.71 (d, *J* = 9.0 Hz, 1H), 7.50 (s, 1H), 7.49 (s, 1H), 4.89 (d, *J* = 4.0 Hz, 1H), 4.67 (d, *J* = 5.5 Hz, 1H), 4.06 (t, *J* = 7.3 Hz, 2H), 4.02 (m, 1H), 3.88 (d, *J* = 24.5 Hz, 1H), 3.84–3.75 (m, 4H), 3.74–3.68 (m, 4H), 2.18 (t, *J* = 7.5 Hz, 2H), 1.84–1.78 (m, 2H), 1.62–1.57 (m, 2H), 1.30–1.12 (m, 56H), 0.87 (t, *J* = 7.0 Hz, 6H); ^{13}C NMR (75 MHz, $CDCl_3$) δ 175.5, 138.8, 129.6, 122.5, 100.8, 75.0, 72.0, 71.2, 70.7, 70.0, 68.1, 62.5, 52.8, 51.6, 50.0, 37.2, 32.8, 31.3, 30.5, 30.4, 30.3, 30.2, 30.1, 27.5, 26.8, 23.5, 14.5.

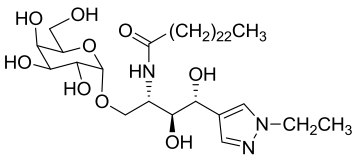

Compound **2b**; 87% yield; $[\alpha]_D^{25}$ +41.9 (c = 0.64, $CHCl_3$/MeOH = 1:1); ^{1}H NMR (500 MHz, $CDCl_3$/CD_3OD = 1:1; reference peak : CD_3OD at 3.31 ppm) δ 7.51 (s, 1H), 7.49 (s, 1H), 4.89 (d, *J* = 4 Hz, 1H), 4.66 (d, *J* = 6.0 Hz, 1H), 4.12 (q, *J* = 7.3 Hz, 2H), 4.05–4.02 (m, 1H), 3.91 (d, *J* = 2.5 Hz, 1H), 3.84–3.77 (m, 4H), 3.74-3.69 (m, 4H), 2.18 (t, *J* = 7.5 Hz, 2H), 1.59 (m, 2H), 1.43 (t, *J* = 7.3 Hz, 3H), 1.29–1.24 (m, 40H), 0.86 (t, *J* = 7.0 Hz, 3H); ^{13}C NMR (75 MHz, $CDCl_3$) δ 175.4, 138.7, 128.8, 122.4, 100.6, 78.5, 74.9, 71.7, 71.0, 70.5, 69.7, 67.9, 62.4, 51.3, 47.4, 37.1, 32.6, 30.4, 30.1, 30.1, 26.6, 23.4, 15.9, 14.5.

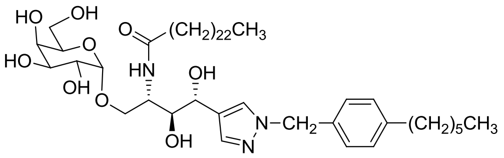

Compound **2c**; 88% yield; $[\alpha]_D^{25}$ +48.6 (c = 1.4, $CHCl_3$/MeOH = 1:1); ^{1}H NMR (500 MHz, $CDCl_3$/CD_3OD = 1:1; reference peak : CD_3OD at 3.31 ppm) δ 7.52 (s, 1H), 7.50 (s, 1H), 7.11 (s, 4H), 5.20 (s, 2H), 4.89 (d, *J* = 4.0 Hz, 1H), 4.64 (m, 1H), 4.05 (m, 1H), 3.90 (d, *J* = 2.5 Hz, 1H), 3.84–3.77 (m, 4H), 3.74–3.67 (m, 4H), 2.54 (t, *J* = 7.5 Hz, 2H), 2.14 (m, 2H), 1.55 (m, 4H), 1.26–1.23 (m, 46H), 0.86–0.84 (m, 6H); ^{13}C NMR (75 MHz, $CDCl_3$) δ 175.3, 143.6, 139.0, 134.3, 129.6, 129.4, 128.4, 123.0, 100.5, 74.9, 71.6, 70.9, 70.4, 69.7, 67.8, 62.4, 56.2, 51.2, 37.0, 36.2, 32.6, 32.4, 32.1, 31.1, 30.3, 30.2, 30.1, 30.0, 29.6, 26.6, 23.3, 23.2, 14.5.

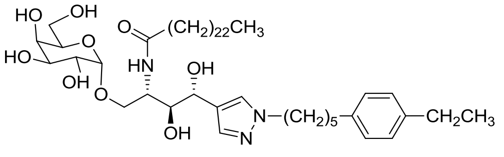

Compound **2d**; 99% yield; $[\alpha]_D^{25}$ +42.0 (c = 1.03, $CHCl_3$/MeOH = 1:1); ^1H NMR (500 MHz, $CDCl_3$/CD_3OD = 1:1; reference peak : CD_3OD at 3.31 ppm) δ 7.59 (s, 1H), 7.58 (s, 1H), 7.07–7.03 (m, 4H), 4.89 (d, *J* = 4.0 Hz, 1H), 4.66 (d, *J* = 5.5 Hz, 1H), 4.09 (t, *J* = 7.3 Hz, 2H), 4.02–3.99 (m, 1H), 3.90 (d, *J* = 3.0 Hz, 1H), 3.84–3.77 (m, 4H), 3.74–3.67 (m, 4H), 2.60–2.53 (m, 4H), 2.18 (dt, *J* = 7.3, 2.0 Hz, 2H), 1.85 (quin, *J* = 7.5 Hz, 2H), 1.65–1.57 (m, 4H), 1.35–1.19 (m, 42H), 1.19 (t, *J* = 7.5 Hz, 3H), 0.85 (t, *J* = 7.0 Hz, 3H); δ 175.4, 142.4, 140.1, 138.0, 129.0, 128.4, 122.9, 100.6, 74.8, 71.8, 71.0, 70.5, 69.8, 67.8, 62.4, 52.7, 51.3, 37.1, 35.9, 32.6, 31.8, 30.9, 30.4, 30.1, 30.1, 29.9, 29.1, 26.8, 26.6, 23.4, 16.1, 14.5.

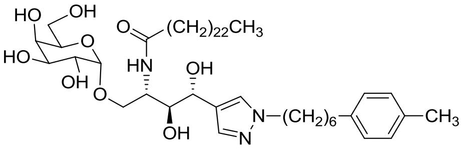

Compound **2e**; 94% yield; $[\alpha]_D^{25}$ +50.2 (c = 0.82, $CHCl_3$/MeOH = 1:1); ^1H NMR (500 MHz, $CDCl_3$/CD_3OD = 1:1; reference peak : CD_3OD at 3.31 ppm) δ 7.60 (s, 1H), 7.56 (s, 1H), 7.02 (q, *J* = 7.5 Hz, 4H), 4.89 (d, *J* = 4.0 Hz, 1H), 4.66 (m, 1H), 4.07 (t, *J* = 7.3 Hz, 2H), 4.03–4.00 (m, 1H), 3.90 (d, *J* = 3.0 Hz, 1H), 3.84–3.76 (m, 4H), 3.74–3.67 (m, 4H), 2.52 (t, *J* = 7.8 Hz, 2H), 2.27 (s, 3H), 2.18 (t, *J* = 8.0 Hz, 2H), 1.81 (quin, *J* = 6.5 Hz, 2H), 1.60–1.54 (m, 4H), 1.32–1.24 (m, 44H), 0.86 (t, *J* = 7.0 Hz, 3H).

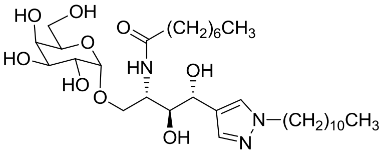

Compound **3a**; 93% yield; $[\alpha]_D^{25}$ +58.0 (c = 0.79, $CHCl_3$/MeOH = 1:1); ^1H NMR (500 MHz, $CDCl_3$/CD_3OD = 1:1; reference peak : CD_3OD at 3.31 ppm) δ 7.54 (d, *J* = 9.0 Hz, 1H), 7.47 (s, 1H), 7.47 (s, 1H), 4.89 (d, *J* = 4.0 Hz, 1H), 4.65 (d, *J* = 6.0 Hz, 1H), 4.09–4.02 (m, 1H), 4.03 (t, *J* = 7.5 Hz, 2H), 3.91 (d, *J* = 3.0 Hz, 1H), 3.84–3.77 (m, 4H), 3.74–3.67 (m, 4H), 2.16 (dt, *J* = 7.5, 2.5 Hz, 2H), 1.82–1.76 (m, 2H), 1.60–1.55 (m, 2H), 1.29–1.23 (m, 24H), 0.86–0.83 (m, 6H); ^{13}C NMR (75 MHz, $CDCl_3$) δ 175.2, 138.4, 129.1, 122.2, 100.4, 74.9, 71.5, 70.8, 70.3, 69.6, 67.8, 62.3, 52.7, 51.1, 36.9, 32.5, 32.3, 31.0, 30.2, 30.1, 29.9, 29.8, 29.6, 27.2, 26.4, 23.2, 23.2, 14.4, 14.4.

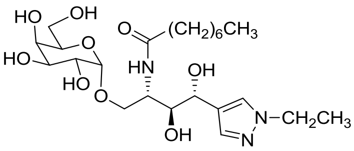

Compound **3b**; 96% yield; $[\alpha]_D^{25}$ +69.9 (c = 0.51, $CHCl_3$/MeOH = 1:1); ^{1}H NMR (500 MHz, $CDCl_3$/CD_3OD = 1:1; reference peak : CD_3OD at 3.31 ppm) δ 7.75 (d, *J* = 9.0 Hz, 1H), 7.52 (s, 1H), 7.49 (s, 1H), 4.89 (d, *J* = 3.5 Hz, 1H), 4.66 (d, *J* = 5.5 Hz, 1H), 4.12 (q, *J* = 7.3 Hz, 2H), 4.04 (m, 1H), 3.90 (d, *J* = 3 Hz, 1H), 3.81 (m, 4H), 3.72 (m, 4H), 2.19 (t, *J* = 7.5 Hz, 2H), 1.60 (m, 2H), 1.43 (t, *J* = 7.3 Hz, 3H), 1.29 (m, 10H), 0.87 (t, *J* = 7.0 Hz, 3H).

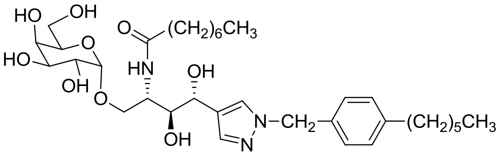

Compound **3c**; 72% yield; $[\alpha]_D^{25}$ +52.4 (c = 0.20, $CHCl_3$/MeOH = 1:1); ^{1}H NMR (500 MHz, $CDCl_3$/CD_3OD = 1:1; reference peak : CD_3OD at 3.31 ppm) δ 7.52 (s, 1H), 7.51 (s, 1H), 7.11 (s, 4H), 7.03 (d, *J* = 2.0 Hz, 1H), 5.20 (s, 2H), 4.89 (d, *J* = 3.5 Hz, 1H), 4.64 (d, *J* = 6.0 Hz, 1H), 4.07–4.04 (m, 1H), 3.90 (d, *J* = 2.5 Hz, 1H), 3.84–3.74 (m, 4H), 3.73–3.67 (m, 4H), 2.55 (t, *J* = 7.8 Hz, 2H), 2.16 (td, *J* = 7.5, 2.0 Hz, 2H), 1.58–1.52 (m, 4H), 1.32–1.24 (m, 14H), 0.88–0.82 (m, 6H).

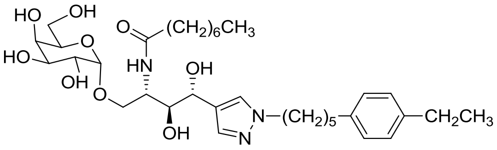

Compound **3d**; 98% yield; $[\alpha]_D^{25}$ +52.3 (c = 0.75, $CHCl_3$/MeOH = 1:1); ^{1}H NMR (500 MHz, $CDCl_3$/CD_3OD = 1:1; reference peak : CD_3OD at 3.31 ppm) δ 7.50 (s, 2H), 7.07–7.03 (m, 4H), 4.89 (d, *J* = 4.0 Hz, 1H), 4.66 (d, *J* = 5.5 Hz, 1H), 4.06–4.02 (m, 3H), 3.90 (d, *J* = 3.0 Hz, 1H), 3.84–3.78 (m 4H), 3.74-3.68 (m, 4H), 2.60–2.52 (m, 4H), 2.18 (dt, *J* = 7.5, 1.5 Hz, 2H), 1.84 (quin, *J* = 7.5 Hz, 2H), 1.64–1.58 (m, 4H), 1.35–1.25 (m, 10H), 1.19 (t, *J* = 7.5 Hz, 3H), 0.86 (t, *J* = 6.8 Hz, 3H).

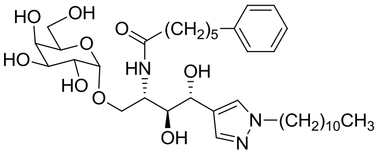

Compound **4a**; 95% yield; $[\alpha]_D^{25}$ +58.0 (c = 0.60, $CHCl_3$/MeOH = 1:1); ^{1}H NMR (500 MHz, $CDCl_3$/CD_3OD = 1:1; reference peak : CD_3OD at 3.31 ppm) δ 7.72 (d, *J* = 9.0 Hz, 1H), 7.49–7.47 (m, 2H), 7.23–7.20 (m, 2H), 7.15–7.10 (m, 3H), 4.88 (d, *J* = 3.5 Hz, 1H), 4.65 (d, *J* = 5.5 Hz, 1H), 4.07–4.02 (m, 3H), 3.89 (d, *J* = 3.5 Hz, 1H), 3.84–3.76 (m, 4H), 3.75–3.66 (m, 4H), 2.59 (t, *J* = 7.8 Hz, 2H), 2.18 (dt, *J* = 6.3, 2.5 Hz, 2H), 1.83–1.70 (m, 2H), 1.66–1.60 (m, 4H), 1.38–1.32 (m, 2H), 1.27–1.24 (m, 16H), 0.86 (t, 3H).

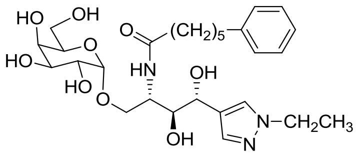

Compound **4b**; 94% yield; $[\alpha]_D^{25}$ +65.2 (c = 0.75, $CHCl_3$/MeOH = 1:1); ^{1}H NMR (500 MHz, $CDCl_3$/CD_3OD = 1:1; reference peak : CD_3OD at 3.31 ppm) δ 7.70 (d, *J* = 9.0H, 1H), 7.50 (s, 1H), 7.48 (s, 1H), 7.22 (t, *J* = 7.5 Hz, 2H), 7.15–7.10 (m, 3H), 4.89 (d, *J* = 4.0 Hz, 1H), 4.65 (d, *J* = 5.5 Hz, 1H), 4.12 (q, *J* = 6.5 Hz, 2H), 4.05–4.02 (m, 1H), 3.90 (d, *J* = 3.0 Hz, 1H), 3.83–3.76 (m, 4H), 3.75–3.66 (m, 4H), 2.59 (t, *J* = 7.5 Hz, 2H), 2.18 (dt, *J* = 7.5, 2.0 Hz, 2H), 1.66–1.58 (m, 4H), 1.43 (t, 7.3 Hz, 3H), 1.38–1.32 (m, 2H).

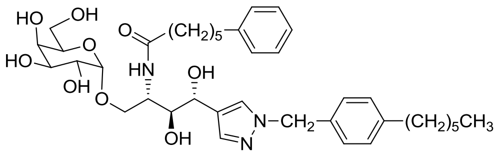

Compound **4c**; 95% yield; $[\alpha]_D^{25}$ +58.2 (c = 0.32, $CHCl_3$/MeOH = 1:1); ^{1}H NMR (500 MHz, $CDCl_3$/CD_3OD = 1:1; reference peak : CD_3OD at 3.31 ppm) δ 7.64 (d, *J* = 0.5 Hz, 1H), 7.53 (s, 2H), 7.21 (t, *J* = 7.5 Hz, 2H), 7.14–7.09 (m, 6H), 5.21 (s, 2H), 4.88 (d, *J* = 4.0 Hz, 1H), 4.65 (d, *J* = 5.5 Hz, 1H), 4.06–4.03 (m, 1H), 3.89 (d, *J* = 3.0 Hz, 1H), 3.85–3.77 (m, 4H), 3.74-3.66 (m, 4H), 2.57 (t, *J* = 7.8 Hz, 2H), 2.54 (t, *J* = 7.5 Hz, 2H), 2.16 (dt, *J* = 7.5, 3.0 Hz, 2H), 1.65–1.58 (m, 4H), 1.56–1.52 (m, 2H), 1.37–1.31 (m, 2H), 1.30–1.25 (m, 4H), 0.84 (t, *J* = 6.8, 3H).

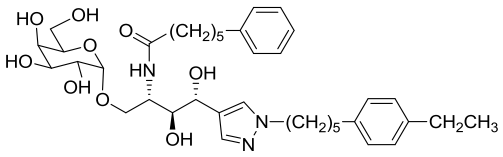

Compound **4d**; 97% yield; $[\alpha]_D^{25}$ +60.8 (c = 0.60, $CHCl_3$/MeOH = 1:1); ^{1}H NMR (500 MHz, $CDCl_3$/CD_3OD = 1:1; reference peak : CD_3OD at 3.31 ppm) δ 7.64 (d, *J* = 9.0 Hz, 1H), 7.48

(d, *J* = 5.5 Hz, 2H), 7.21 (t, *J* = 6.8 Hz, 2H), 7.14–7.10 (m, 3H), 7.07–7.02 (m, 4H), 4.89 (d, *J* = 4.0 Hz, 1H), 4.64 (d, *J* = 3.5 Hz, 1H), 4.07–4.02 (m, 3H), 3.90 (d, *J* = 3.0 Hz, 1H), 3.84–3.77 (m, 4H), 3.73–3.68 (m, 4H), 2.60–2.52 (m, 6H), 2.18 (dt, *J* = 7.5, 2.5 Hz, 2H), 1.83 (quin, *J* = 7.5 Hz, 2H), 1.65–1.57 (m, 6H), 1.38–1.28 (m, 4H), 1.18 (t, *J* = 7.8 Hz, 3H).

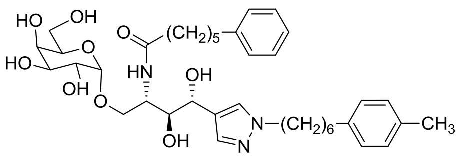

Compound **4e**; 97% yield; $[\alpha]_D^{25}$ +52.5 (c = 0.45, $CHCl_3$/MeOH = 1:1); ^{1}H NMR (500 MHz, $CDCl_3$/CD_3OD = 1:1; reference peak : CD_3OD at 3.31 ppm) δ 7.49 (s, 1H), 7.49 (s, 1H), 7.23–7.20 (m, 2H), 7.14–7.01 (m, 3H), 7.03–6,99 (m, 4H), 4.88 (d, *J* =4.0 Hz, 1H), 4.65 (d, *J* = 5.0 Hz, 1H), 4.07–4.00 (m, 3H), 3.89 (d, *J* = 3.0 Hz, 1H), 3.84–3.3.76 (m, 4H), 3.74–3.66 (m, 4H), 2.59 (t, *J* = 7.5 Hz, 2H), 2.51 (t, *J* = 7.8 Hz, 2H), 2.26 (s, 3H), 2.18 (dt, *J* = 7.5, 1.8 Hz, 2H), 1.80 (quin, *J* = 6.0 Hz, 2H), 1.66–1.60 (m, 4H), 1.59–1.53 (m, 2H), 1.38–1.25 (m, 8H).

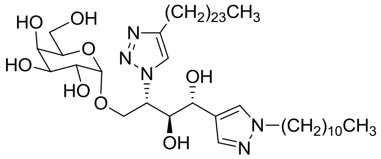

Compound **5a**; 59% yield; $[\alpha]_D^{25}$ +32.5 (c = 0.65, $CHCl_3$/MeOH = 1:1); ^{1}H NMR (500 MHz, $CDCl_3$/CD_3OD = 1:1; reference peak : CD_3OD at 3.31 ppm) δ 8.12 (s, 1H), 7.64 (s, 1H), 7.58 (s, 1H), 5.08 (m, 1H), 5.09–5.06 (m, 1H), 4.86 (d, *J* = 3.5 Hz, 1H), 4.52 (d, *J* = 6.5 Hz, 1H), 4.30-4.27 (m, 1H), 4.16–4.09 (m, 4H), 3.87 (d, *J* = 3.0 Hz, 1H), 3.79–3.73 (m, 1H), 3.73–3.62 (m, 4H), 2.75 (t, *J* = 7.8 Hz, 2H), 1.85–1.79 (m, 2H), 1.71–1.65 (m, 2H), 1.39–1.23 (m, 58H), 0.87–0.83 (m, 6H).

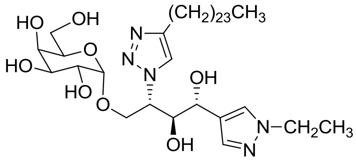

Compound **5b**; 88% yield; $[\alpha]_D^{25}$ +39.8 (c = 0.29, $CHCl_3$/MeOH = 1:1); ^{1}H NMR (500 MHz, $CDCl_3$/CD_3OD = 1:1; reference peak : CD_3OD at 3.31 ppm) δ 8.11 (s, 1H), 7.65 (s, 1H), 7.59 (s, 1H), 5.10–5.06 (m, 1H), 4.86 (d, *J* = 4.0 Hz, 1H), 4.52 (d, *J* = 6.5 Hz, 1H), 4.30–4.27 (m,

1H), 4.21–4.08 (m, 4H), 3.87 (d, J = 3 Hz, 1H), 3.79–3.62 (m, 5H), 2.74 (t, J = 7.8 Hz, 2H), 1.70–1.65 (m, 2H), 1.46 (t, J = 3.5 Hz, 3H), 1.35–1.22 (m, 42H), 0.85 (t, J = 6.8 Hz, 3H).

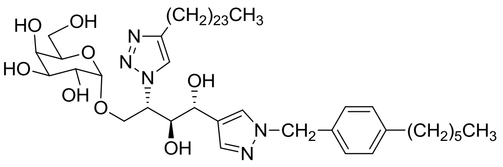

Compound **5c**; 79% yield; $[\alpha]_D^{25}$ +41.2 (c = 0.60, $CHCl_3$/MeOH = 1:1); ^{1}H NMR (500 MHz, $CDCl_3$/CD_3OD = 1:1; reference peak : CD_3OD at 3.31 ppm) δ 8.08 (s, 1H), 7.98 (s, 1H), 7.58 (s, 1H), 7.55 (s, 1H), 7.15–7.09 (m, 3H), 5.24 (s, 2H), 5.08 (m, 1H), 4.85 (d, J = 3.5 Hz, 1H), 4.52 (d, J = 7.0 Hz, 1H), 4.31–4.26 (m, 1H), 4.15–4.08 (m, 2H), 3.88–3.86 (m, 1H), 3.81–3.62 (m 5H), 2.75–2.71 (m, 2H), 2.53 (t, J = 7.8 Hz, 2H), 1.69–1.63 (m 2H), 1.57–1.51 (m, 2H), 1.36–1.18 (m 48H), 0.86–0.83 (m, 3H).

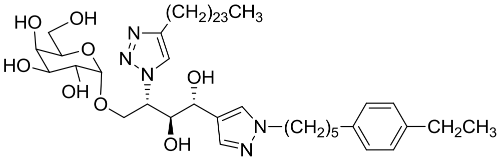

Compound **5d**; 88% yield; $[\alpha]_D^{25}$ +41.1 (c = 1.0, $CHCl_3$/MeOH = 1:1); ^{1}H NMR (500 MHz, $CDCl_3$/CD_3OD = 1:1; reference peak : CD_3OD at 3.31 ppm) δ 7.83 (s, 1H), 7.49 (s, 1H), 7.47 (s, 1H), 7.07–7.02 (m, 4H), 4.91 (m, 1H), 4.85 (d, J = 4.0 Hz, 1H), 4.43 (d, J = 6.0 Hz, 1H), 4.22–4.17 (m, 2H), 4.08–4.05 (m, 3H), 3.86 (d, J = 3.0 Hz, 1H), 3.77 (dd, J = 10.0, 4.0 Hz, 1H), 3.71–3.63 (m, 3H), 3.60–3.57 (m, 1H), 2.69 (t, J = 7.8 Hz, 2H), 2.59–2.52 (m, 4H), 1.83 (quin, J = 7.5 Hz, 2H), 1.66–1.58 (m, 4H), 1.36–1.21 (m, 44H), 1.18 (t, J = 7.5 Hz, 3H), 0.86 (t, J = 7.0 Hz, 3H).

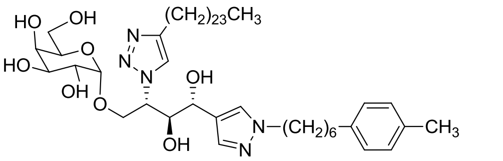

Compound **5e**; 93% yield; $[\alpha]_D^{25}$ +42.0 (c = 0.40, $CHCl_3$/MeOH = 1:1); ^{1}H NMR (500 MHz, $CDCl_3$/CD_3OD = 1:1; reference peak : CD_3OD at 3.31 ppm) δ 7.71 (s, 1H), 7.45 (s, 1H), 7.44 (s, 1H), 7.02 (q, J = 7.5 Hz, 4H), 4.85–4.82 (m, 2H), 4.39 (d, J = 5.0 Hz, 1H), 4.22–4.16 (m, 2H), 4.05–4.02 (m, 3H), 3.86 (d, J = 3.0 Hz, 1H), 3.77 (dd, J = 10.0, 4.0 Hz, 1H), 3.70–3.62 (m, 3H), 3.58–3.55 (m, 1H), 2.66 (t, J = 7.5 Hz, 2H), 2.52 (t, J = 7.5 Hz, 2H), 2.26 (s, 3H),

1.80 (quin, J = 7.5 Hz, 2H), 1.64 (quin, J = 7.5 Hz, 2H), 1.56 (quin, J = 7.5 Hz, 2H), 1.36–1.20 (m, 46H), 0.86 (t, J = 7.0 Hz, 3H).

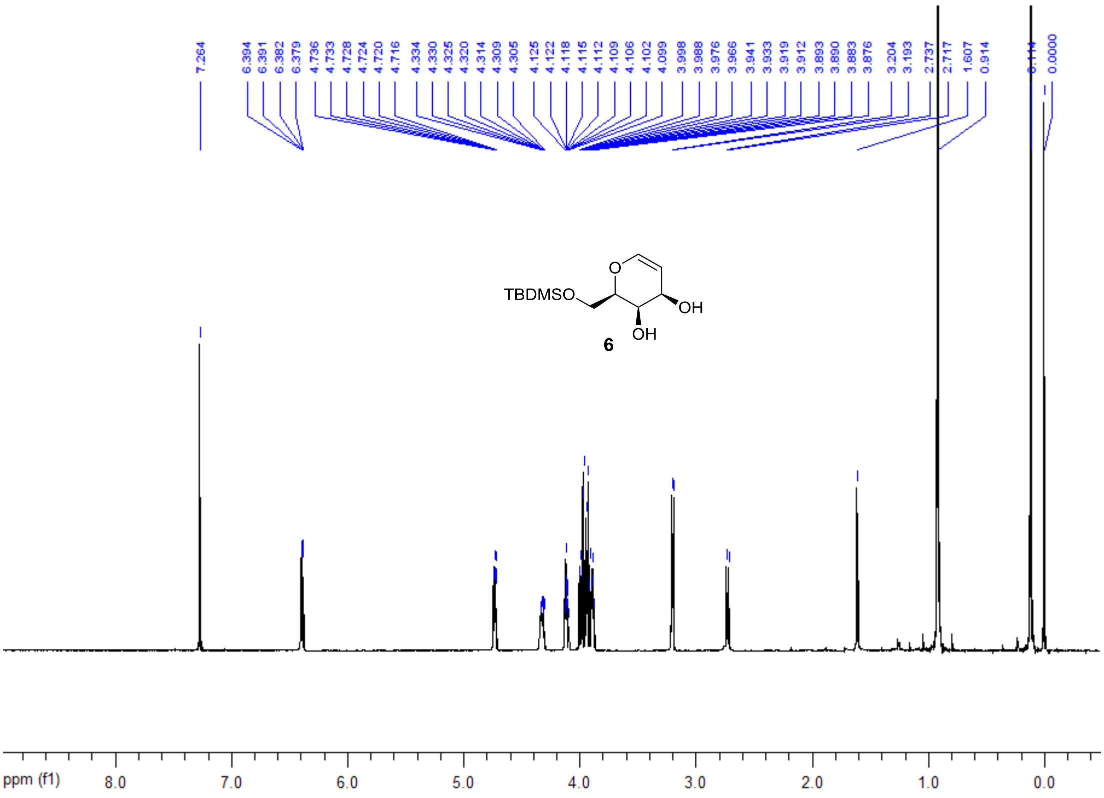

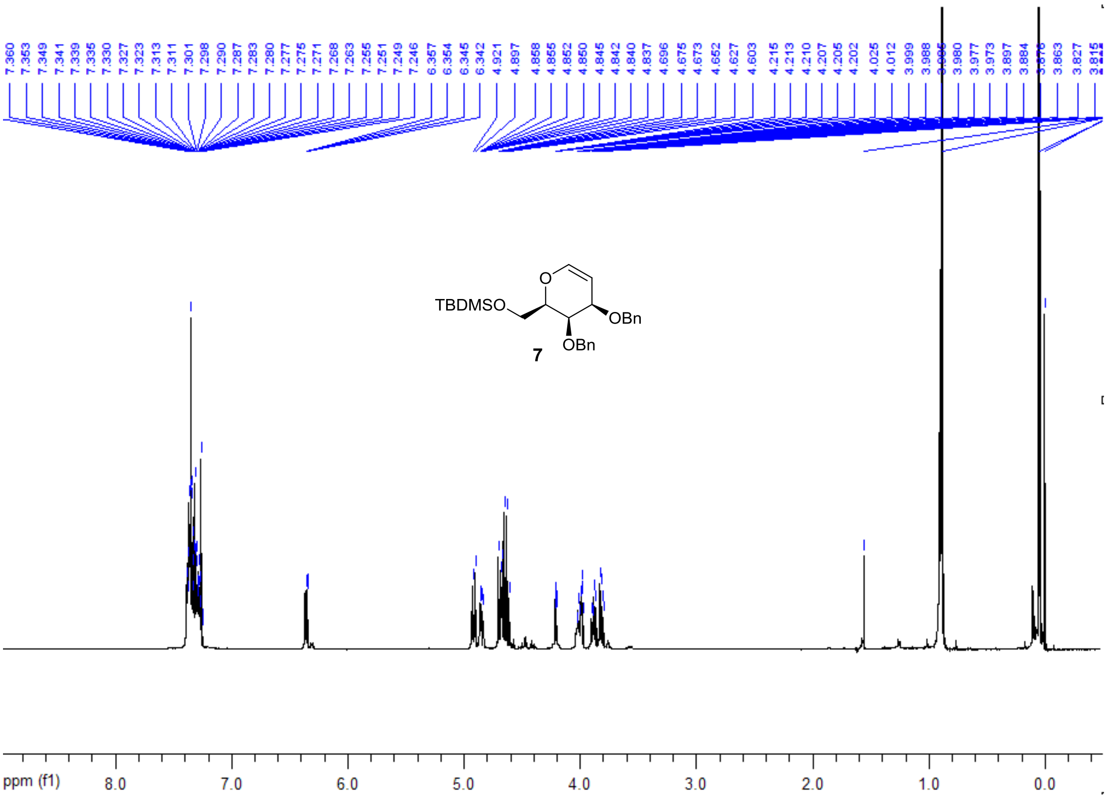
TBDMSO
O
OBn
OBn
7
ppm (f1)
8.0
7.0
6.0
5.0
4.0
3.0
2.0
1.0
0.0

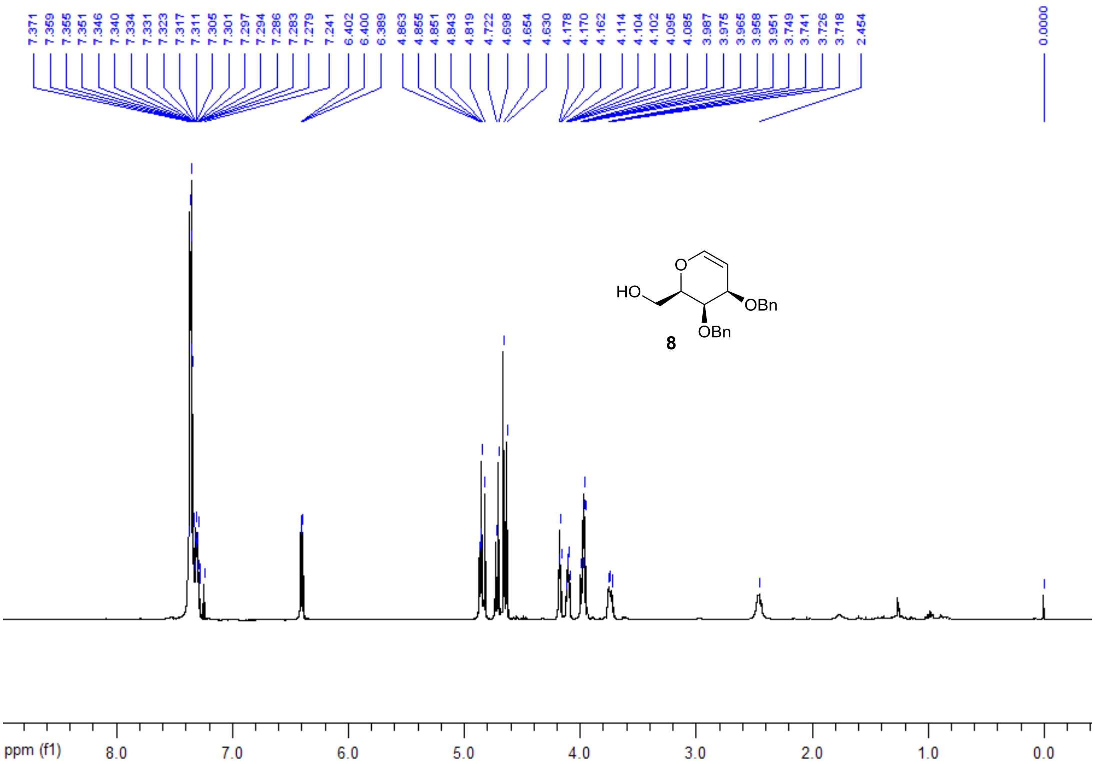
HO
O
OBn
OBn
8
ppm (f1)
8.0
7.0
6.0
5.0
4.0
3.0
2.0
1.0
0.0

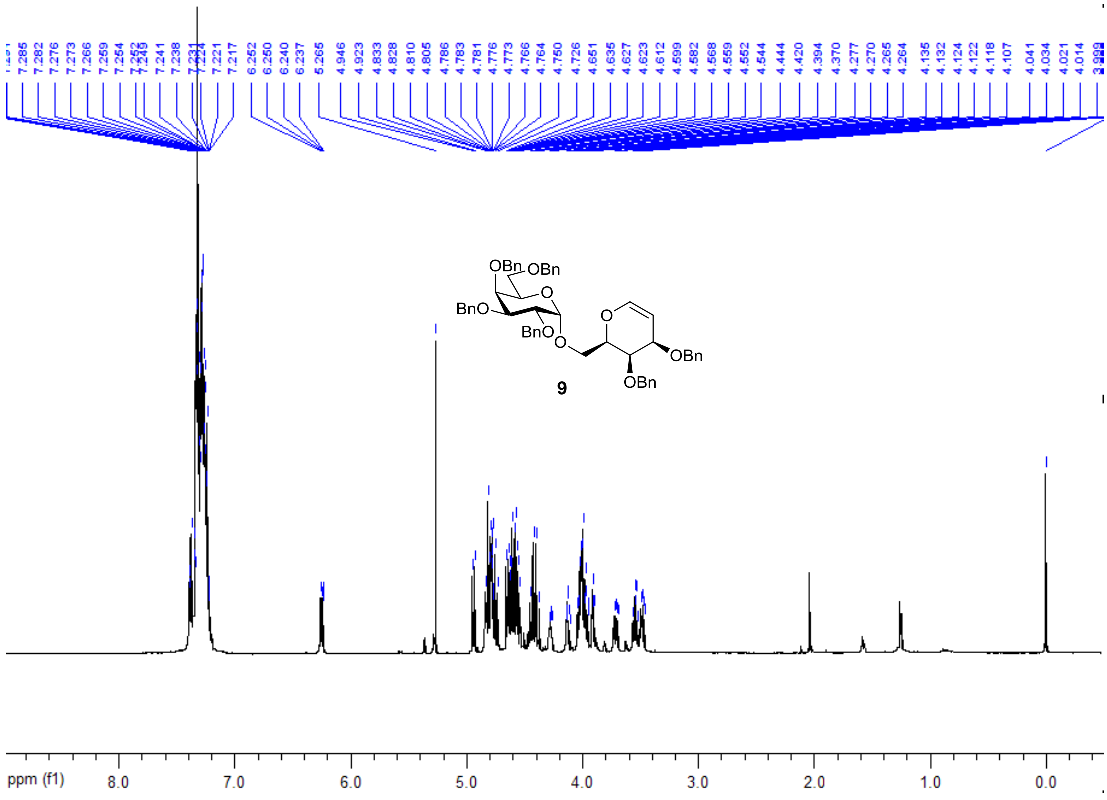
7.285
7.282
7.276
7.273
7.266
7.259
7.254
7.241
7.238
7.231
7.224
7.221
7.217
6.252
6.250
6.240
6.237
5.265
4.946
4.923
4.833
4.828
4.810
4.805
4.786
4.783
4.781
4.776
4.773
4.766
4.764
4.750
4.726
4.651
4.635
4.627
4.623
4.612
4.599
4.582
4.568
4.559
4.552
4.544
4.444
4.420
4.394
4.370
4.277
4.270
4.265
4.264
4.135
4.132
4.124
4.122
4.118
4.107
4.041
4.034
4.021
4.014
OBn
OBn
BnO
O
BnO
O
O
OBn
OBn
9
ppm (f1)
8.0
7.0
6.0
5.0
4.0
3.0
2.0
1.0
0.0

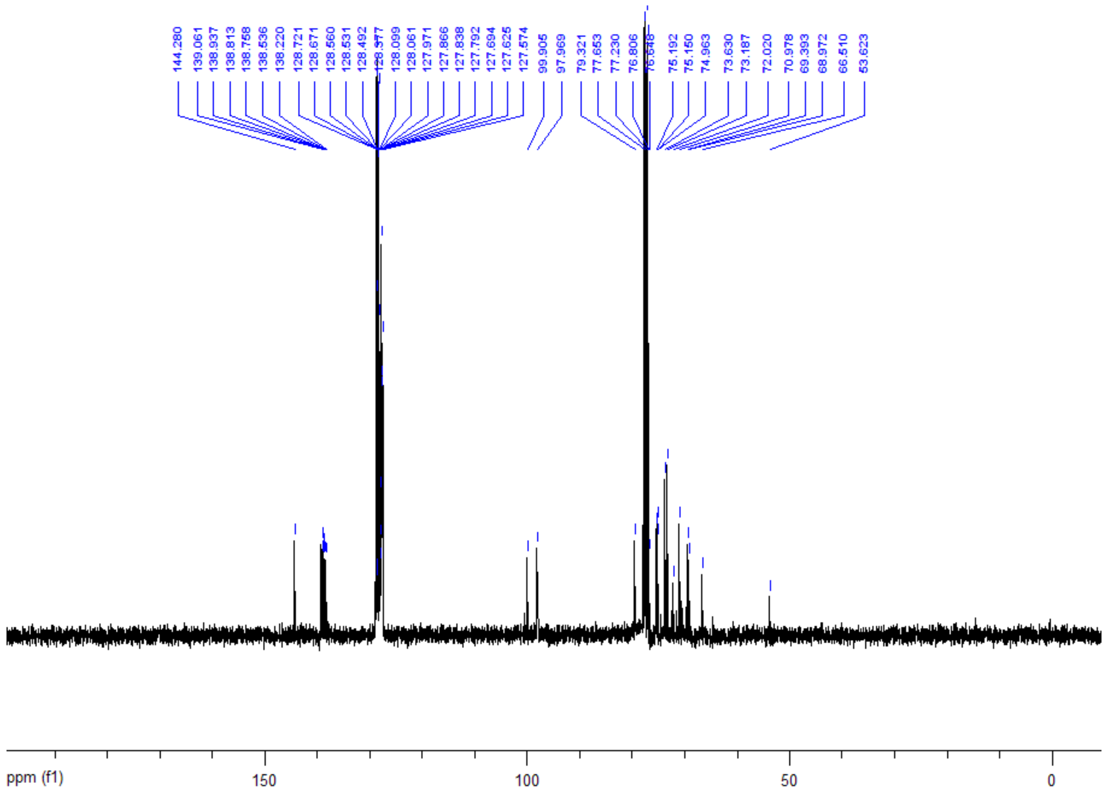
144.280
139.061
138.937
138.813
138.758
138.536
138.220
128.721
128.671
128.560
128.531
128.492
128.099
128.061
127.971
127.866
127.838
127.792
127.694
127.625
127.574
99.905
97.969
79.321
77.653
77.230
76.806
75.192
75.150
74.963
73.630
73.187
72.020
70.978
69.393
68.972
66.510
53.623
ppm (f1)
150
100
50
0

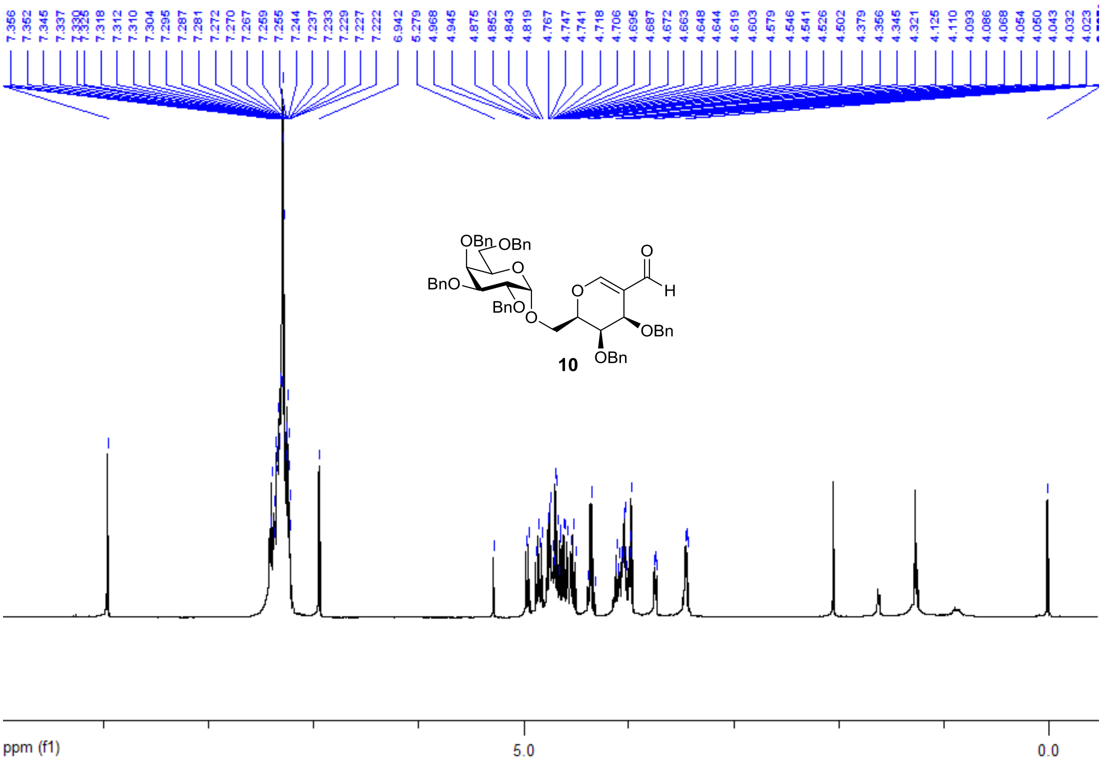
OBn
OBn
BnO
BnO
O
O
O
H
OBn
OBn
10
ppm (f1)
5.0
0.0

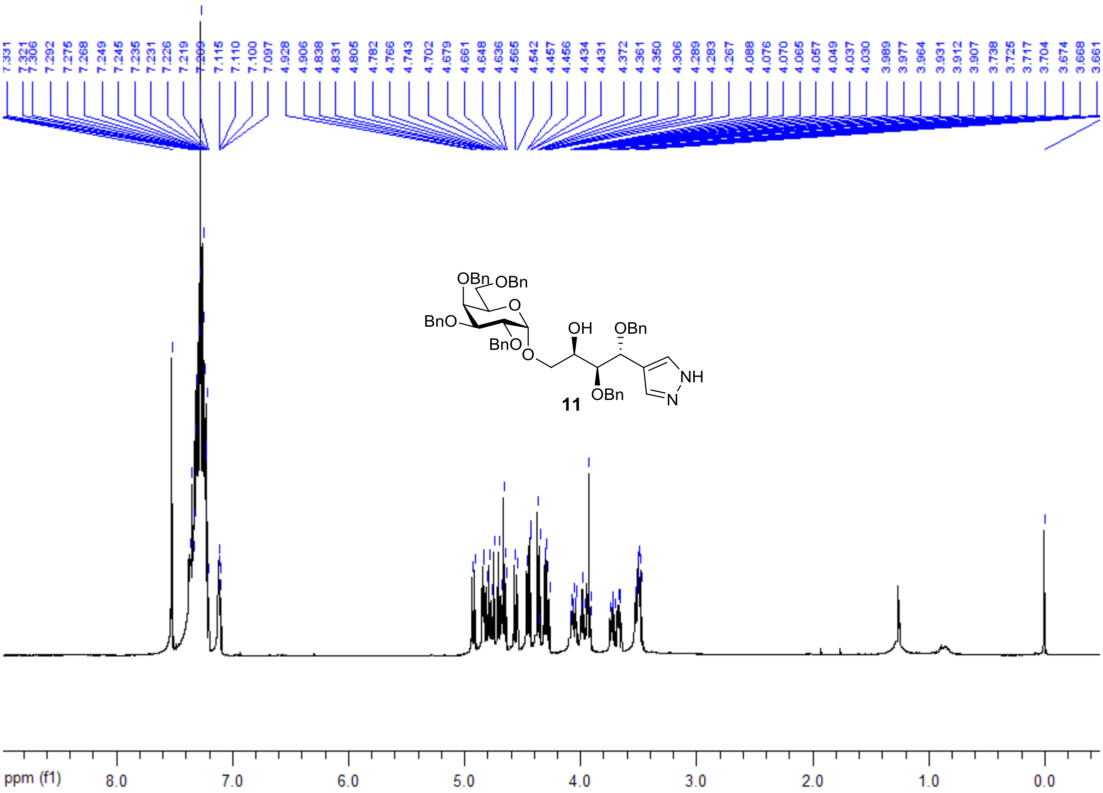
OBn
OBn
BnO
BnO
O
OH
OBn
NH
N
OBn
11
ppm (f1)
8.0
7.0
6.0
5.0
4.0
3.0
2.0
1.0
0.0

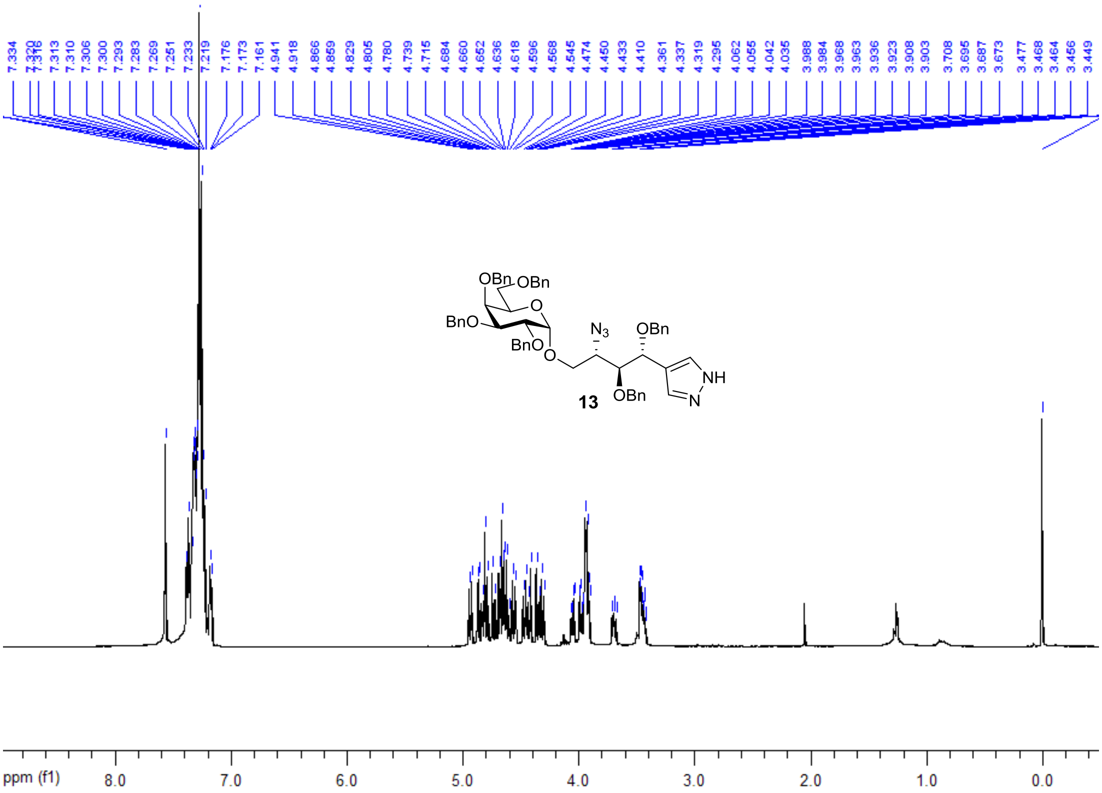
7.334
7.320
7.318
7.313
7.310
7.306
7.300
7.293
7.283
7.269
7.251
7.233
7.219
7.176
7.173
7.161
4.941
4.918
4.866
4.859
4.829
4.805
4.780
4.739
4.715
4.684
4.660
4.652
4.636
4.618
4.596
4.568
4.545
4.474
4.450
4.433
4.410
4.361
4.337
4.319
4.295
4.062
4.055
4.042
4.035
3.988
3.984
3.968
3.963
3.936
3.923
3.908
3.903
3.708
3.695
3.687
3.673
3.477
3.468
3.464
3.456
3.449
OBn
OBn
BnO
BnO
O
O
N3
OBn
NH
N
OBn
13
ppm (f1)
8.0
7.0
6.0
5.0
4.0
3.0
2.0
1.0
0.0

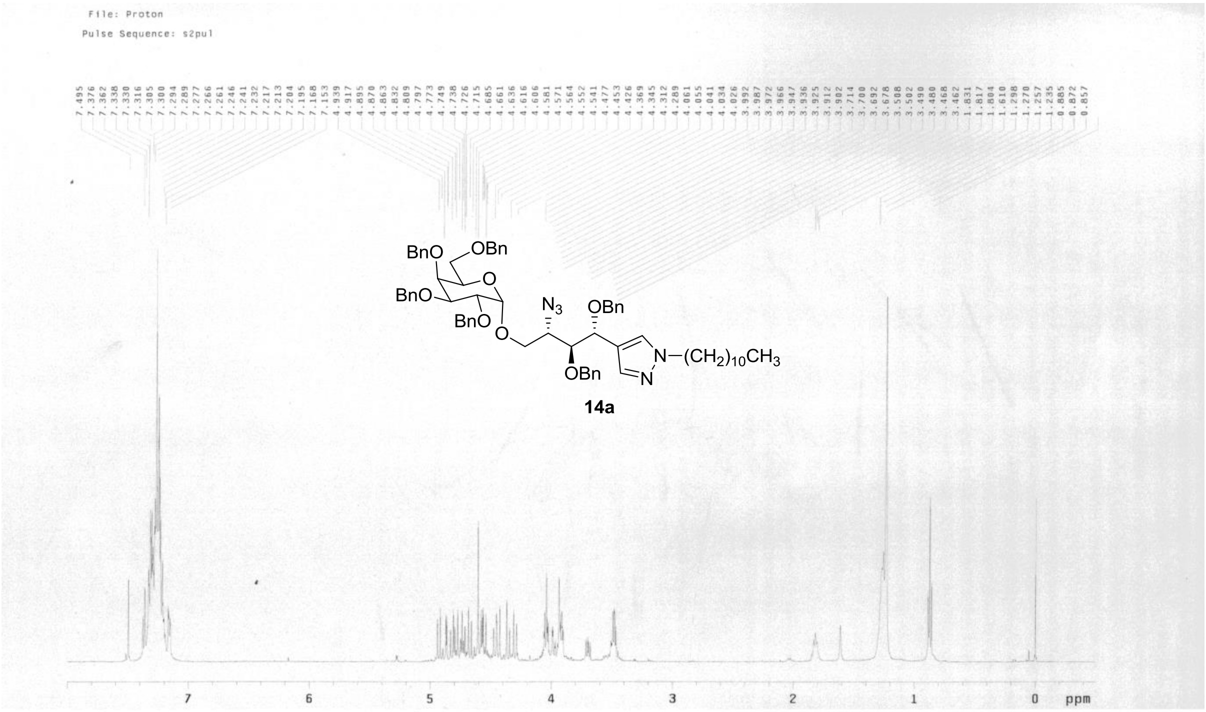
BnO
OBn
O
BnO
BnO
O
N3
OBn
N—(CH2)10CH3
N
OBn
14a
7
6
5
4
3
2
1
0
ppm

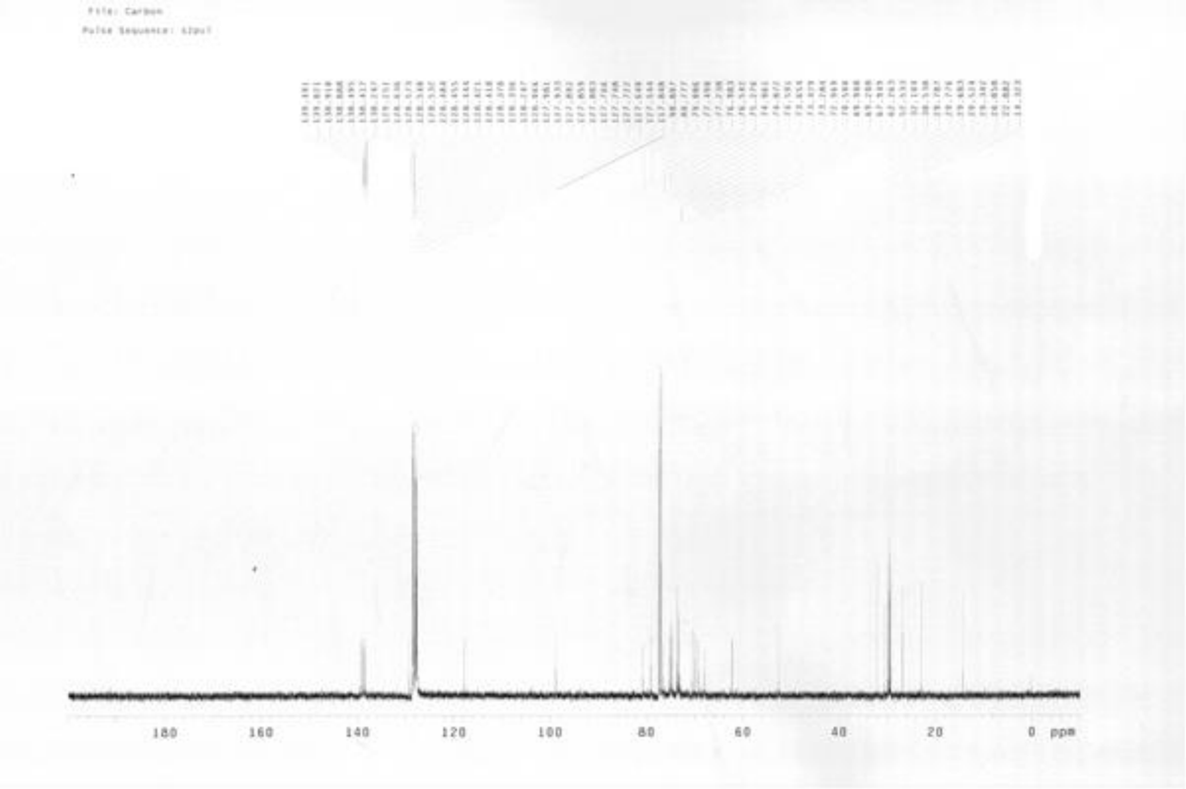
File: Carbon
Pulse Sequence: s2pul
180
160
140
120
100
80
60
40
20
0 ppm

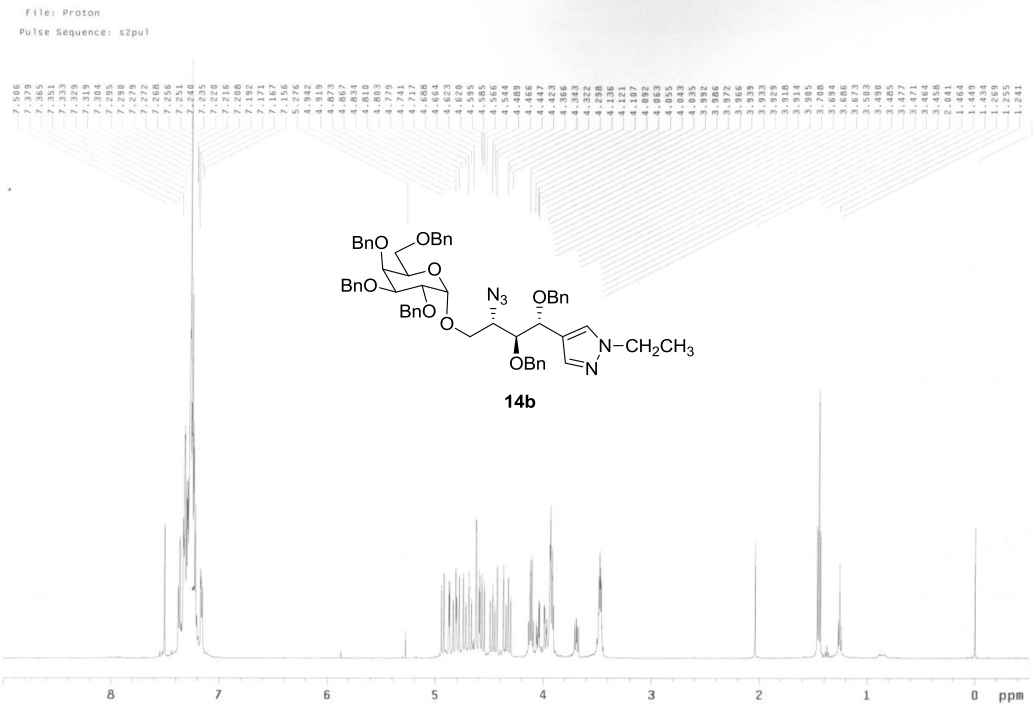
File: Proton
Pulse Sequence: s2pul
BnO
OBn
BnO
BnO
N3
OBn
O
OBn
N
N–CH2CH3
14b
8
7
6
5
4
3
2
1
0 ppm

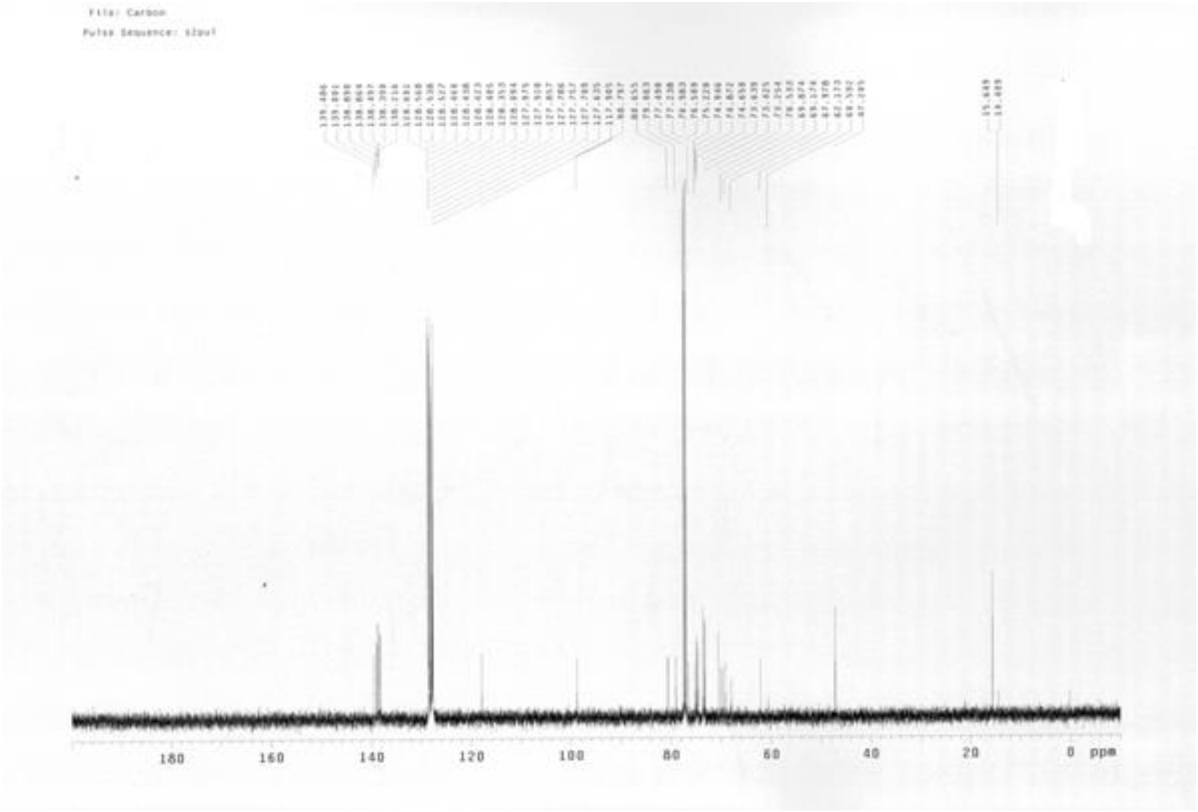
File: Carbon
Pulse Sequence: s2pul
180
160
140
120
100
80
60
40
20
0 ppm

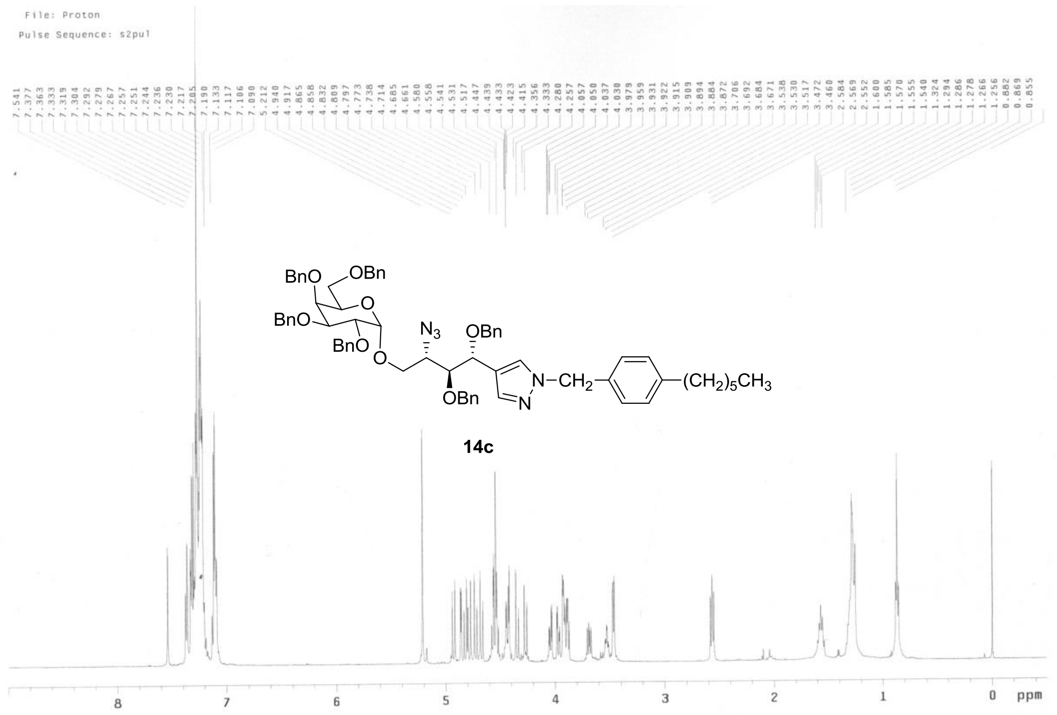
File: Proton
Pulse Sequence: s2pul
BnO
OBn
BnO
BnO
O
O
N3
OBn
OBn
N
N
N–CH2
(CH2)5CH3
8
7
6
5
4
3
2
1
0 ppm

14c

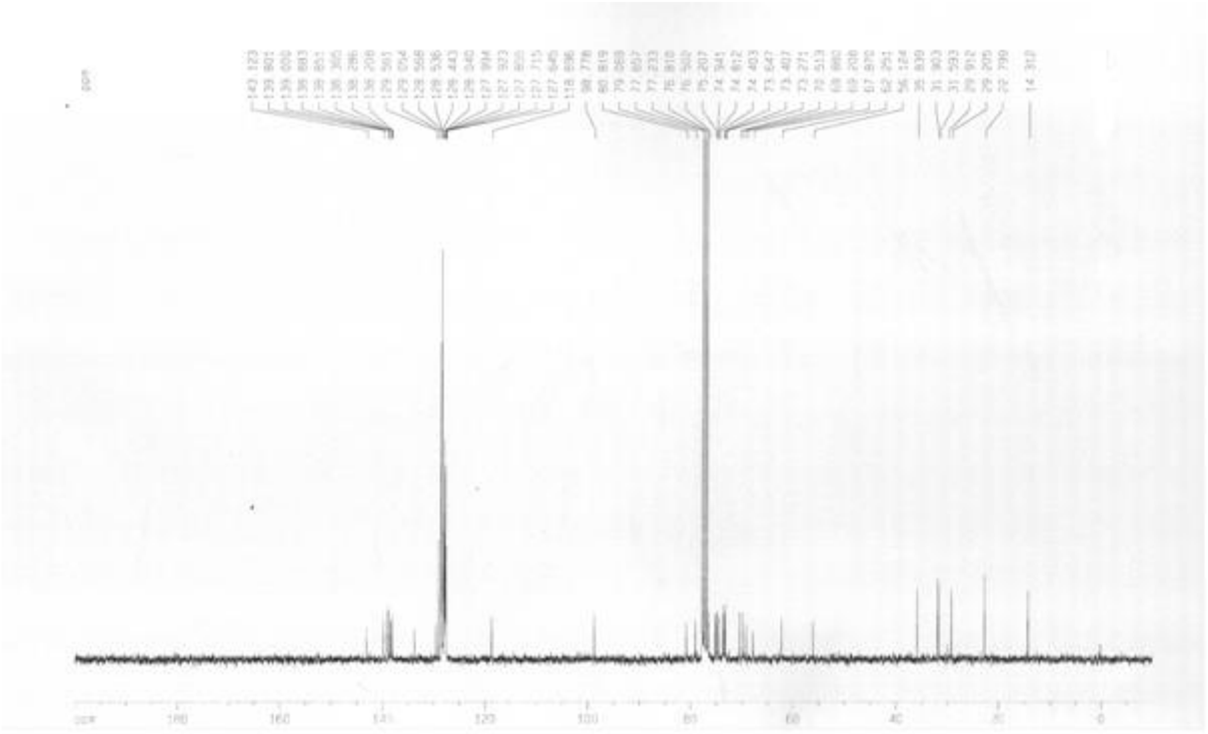

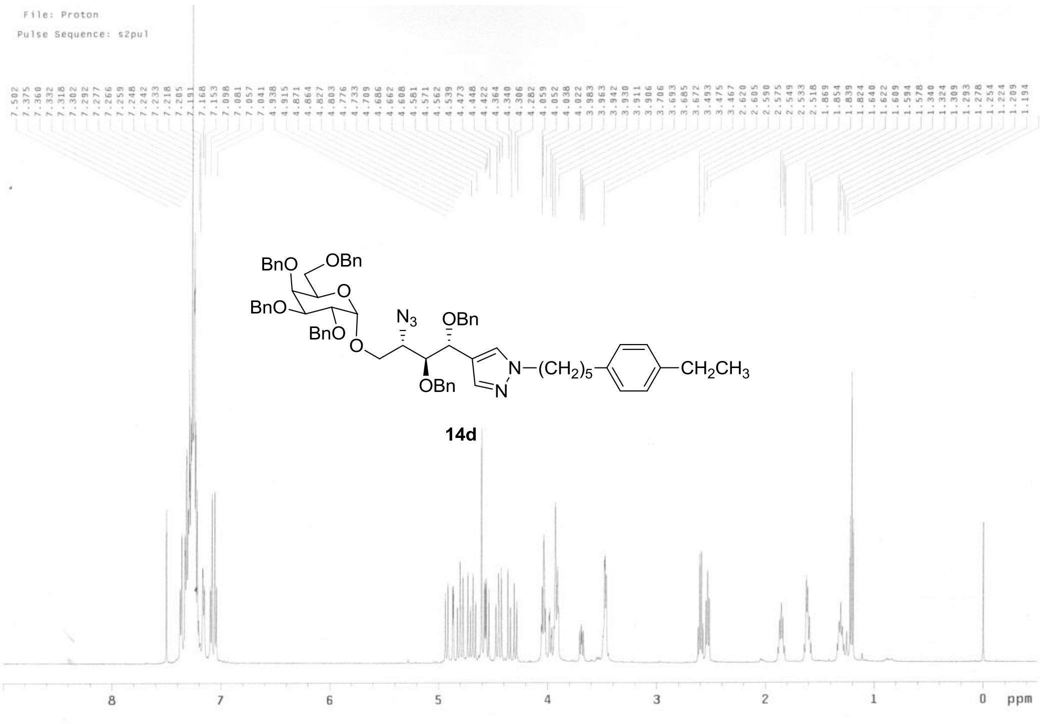

File: Proton
Pulse Sequence: s2pul
BnO
OBn
BnO
BnO
O
O
N3
OBn
OBn
N
N–(CH2)5
CH2CH3
14d
ppm

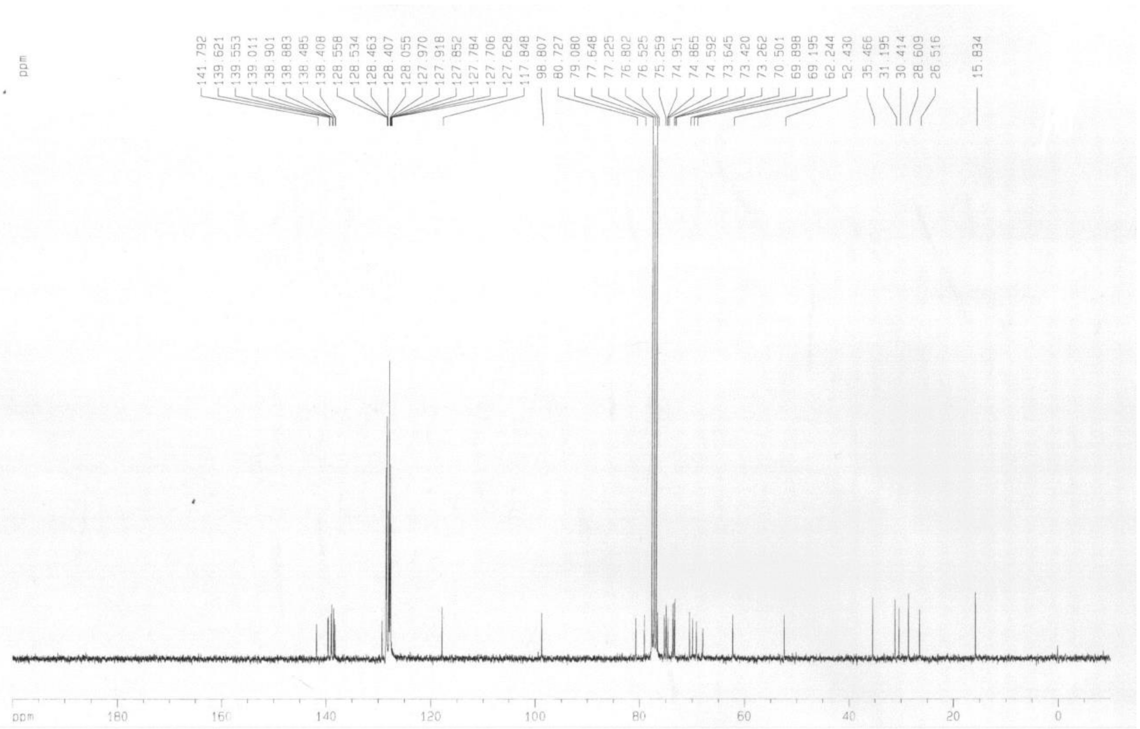
ppm
141.792
139.621
139.553
139.011
138.901
138.883
138.485
138.408
128.558
128.534
128.463
128.407
128.055
127.970
127.918
127.852
127.784
127.706
127.628
117.848
98.807
80.727
79.080
77.648
77.225
76.802
76.525
75.259
74.951
74.865
74.592
73.645
73.420
73.262
70.501
69.898
69.195
62.244
52.430
35.466
31.195
30.414
28.609
26.516
15.834
ppm 180 160 140 120 100 80 60 40 20 0

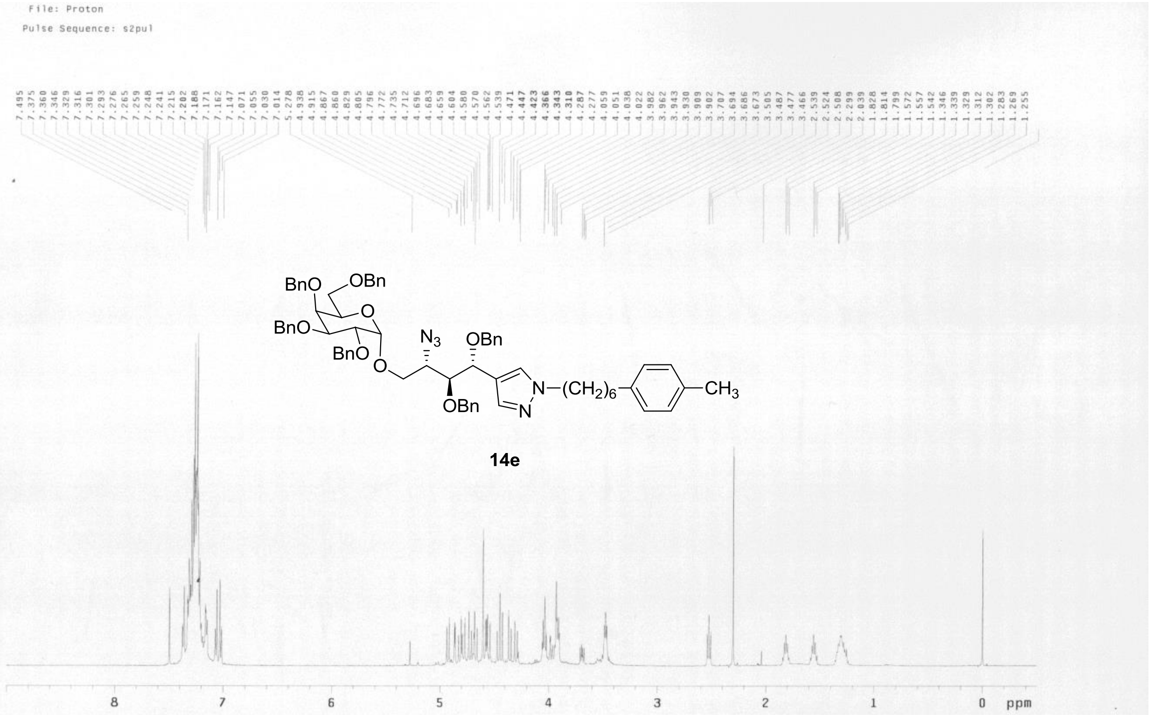
File: Proton
Pulse Sequence: s2pul
7.495
7.375
7.360
7.346
7.329
7.316
7.301
7.293
7.276
7.265
7.259
7.248
7.241
7.215
7.202
7.188
7.171
7.162
7.147
7.071
7.055
7.030
7.014
5.278
4.938
4.915
4.867
4.860
4.829
4.805
4.796
4.772
4.735
4.712
4.696
4.683
4.659
4.604
4.580
4.570
4.562
4.539
4.471
4.447
4.423
4.366
4.343
4.310
4.287
4.277
4.059
4.051
4.038
4.022
3.982
3.962
3.943
3.930
3.909
3.902
3.707
3.694
3.686
3.673
3.505
3.487
3.477
3.466
2.539
2.524
2.508
2.299
2.039
1.828
1.814
1.799
1.572
1.557
1.542
1.346
1.339
1.329
1.312
1.302
1.283
1.269
1.255
BnO
OBn
BnO
BnO
O
N3
OBn
OBn
N–(CH2)6
N
CH3
14e
8 7 6 5 4 3 2 1 0 ppm

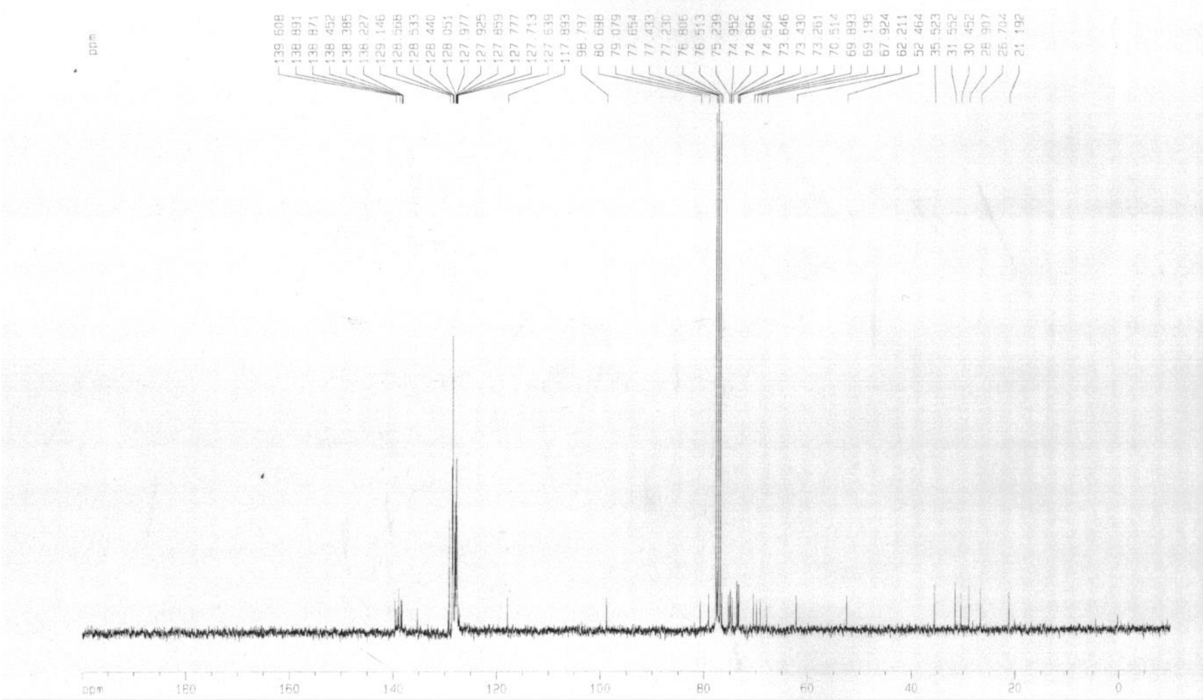
ppm
180
160
140
120
100
80
60
40
20
0

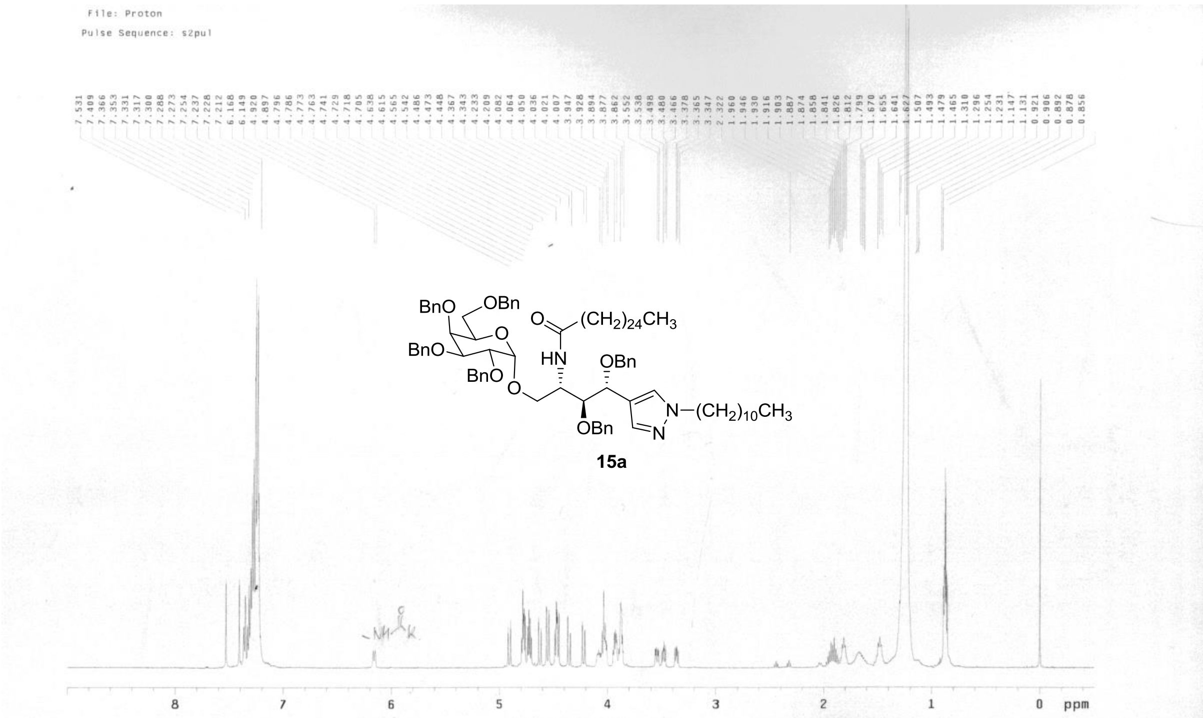
File: Proton
Pulse Sequence: s2pul
BnO
OBn
BnO
BnO
O
O
HN
O
(CH2)24CH3
OBn
OBn
N
N
(CH2)10CH3
15a
8
7
6
5
4
3
2
1
0
ppm

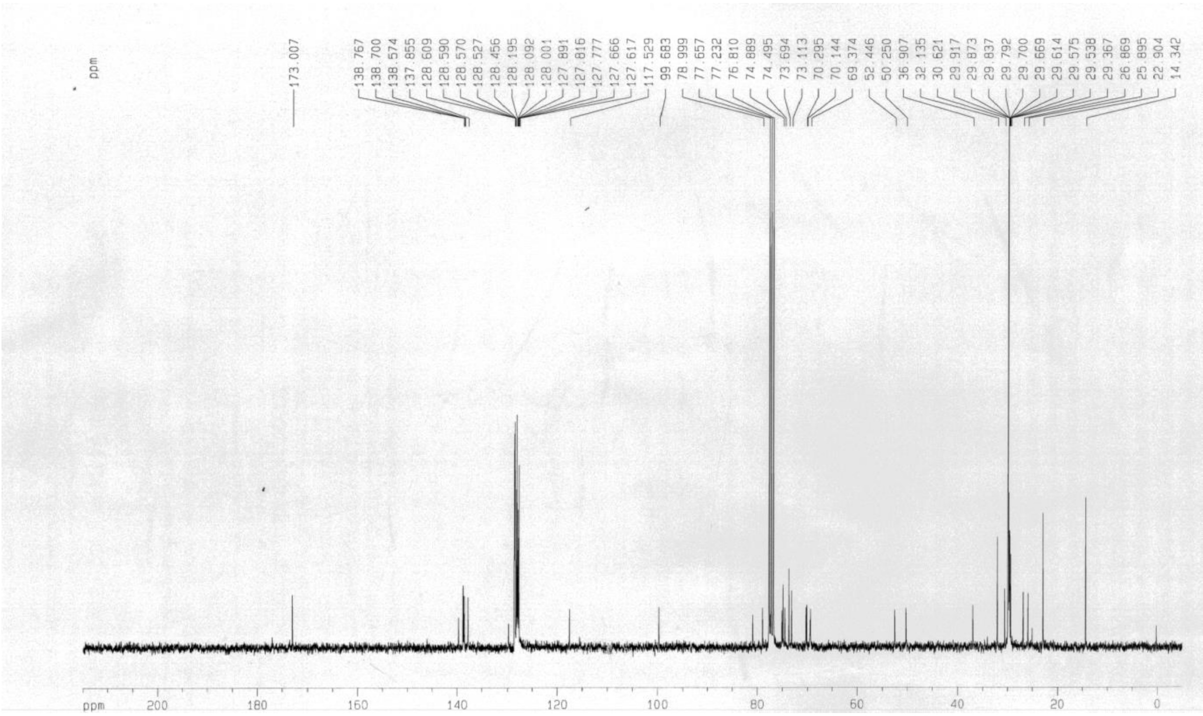
ppm
173.007
138.767 138.700 138.574 137.855 128.609 128.590 128.570 128.527 128.456 128.195 128.092 128.001 127.891 127.816 127.777 127.666 127.617 117.529
99.683
78.999 77.657 77.232 76.810 74.889 74.495 73.694 73.113 70.295 70.144 69.374
52.446 50.250
36.907 32.135 30.621 29.917 29.873 29.837 29.792 29.700 29.669 29.614 29.575 29.538 29.367 26.869 25.895 22.904 14.342
ppm 200 180 160 140 120 100 80 60 40 20 0

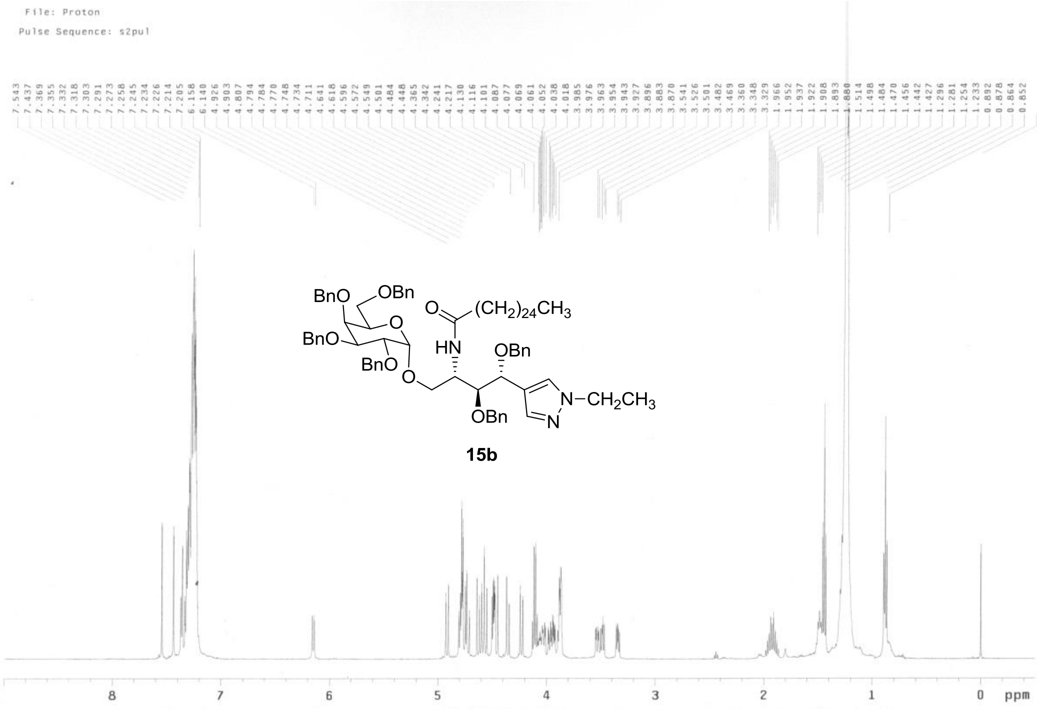
File: Proton
Pulse Sequence: s2pul
7.543 7.437 7.369 7.355 7.332 7.318 7.303 7.291 7.273 7.258 7.245 7.234 7.226 7.214 7.205 6.158 6.140 4.926 4.903 4.807 4.794 4.784 4.770 4.748 4.734 4.711 4.641 4.618 4.596 4.572 4.549 4.501 4.484 4.448 4.365 4.342 4.241 4.217 4.130 4.116 4.101 4.087 4.077 4.069 4.061 4.052 4.038 4.018 3.985 3.976 3.963 3.954 3.943 3.927 3.896 3.883 3.870 3.541 3.526 3.501 3.482 3.469 3.360 3.348 3.329 1.966 1.952 1.937 1.922 1.908 1.893 1.880 1.514 1.498 1.484 1.470 1.456 1.442 1.427 1.296 1.281 1.254 1.233 0.892 0.878 0.864 0.852
BnO
OBn
O
(CH2)24CH3
BnO
HN
OBn
BnO
O
N-CH2CH3
OBn
N
15b
8 7 6 5 4 3 2 1 0 ppm

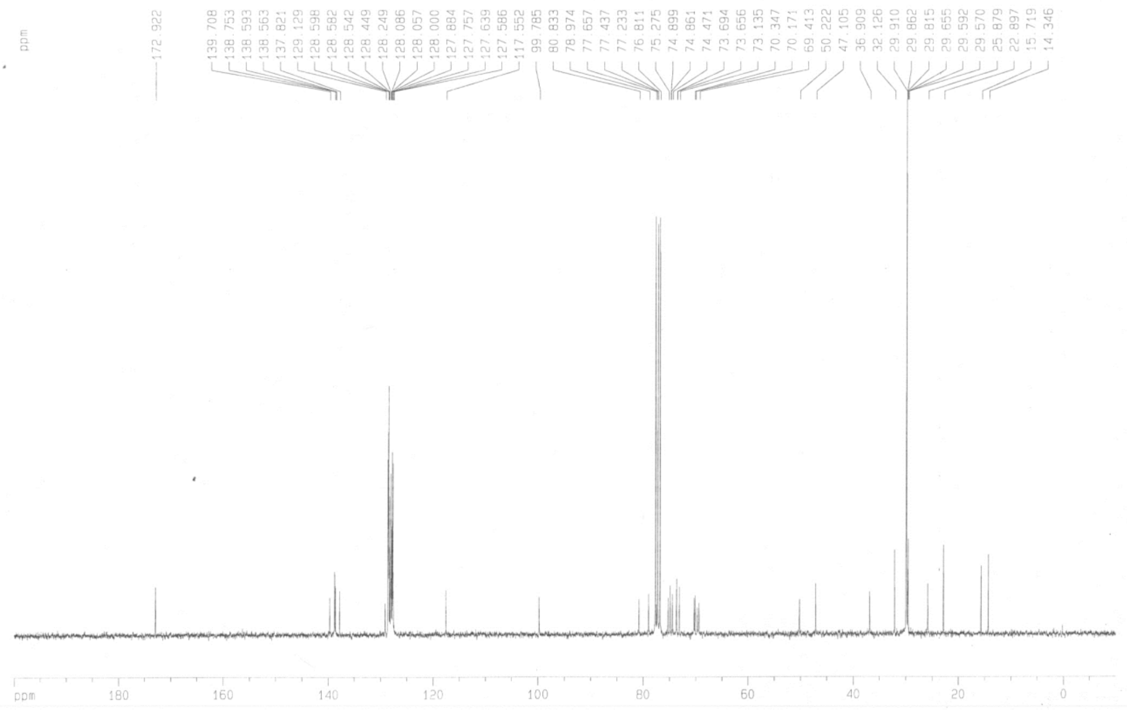
ppm
172.922
139.708
138.753
138.593
138.563
137.821
129.129
128.598
128.582
128.542
128.449
128.249
128.086
128.057
128.000
127.884
127.757
127.639
127.586
117.552
99.785
80.833
78.974
77.657
77.437
77.233
76.811
75.275
74.899
74.861
74.471
73.694
73.656
73.135
70.347
70.171
69.413
50.222
47.105
36.909
32.126
29.910
29.862
29.815
29.655
29.592
29.570
25.879
22.897
15.719
14.346
ppm 180 160 140 120 100 80 60 40 20 0

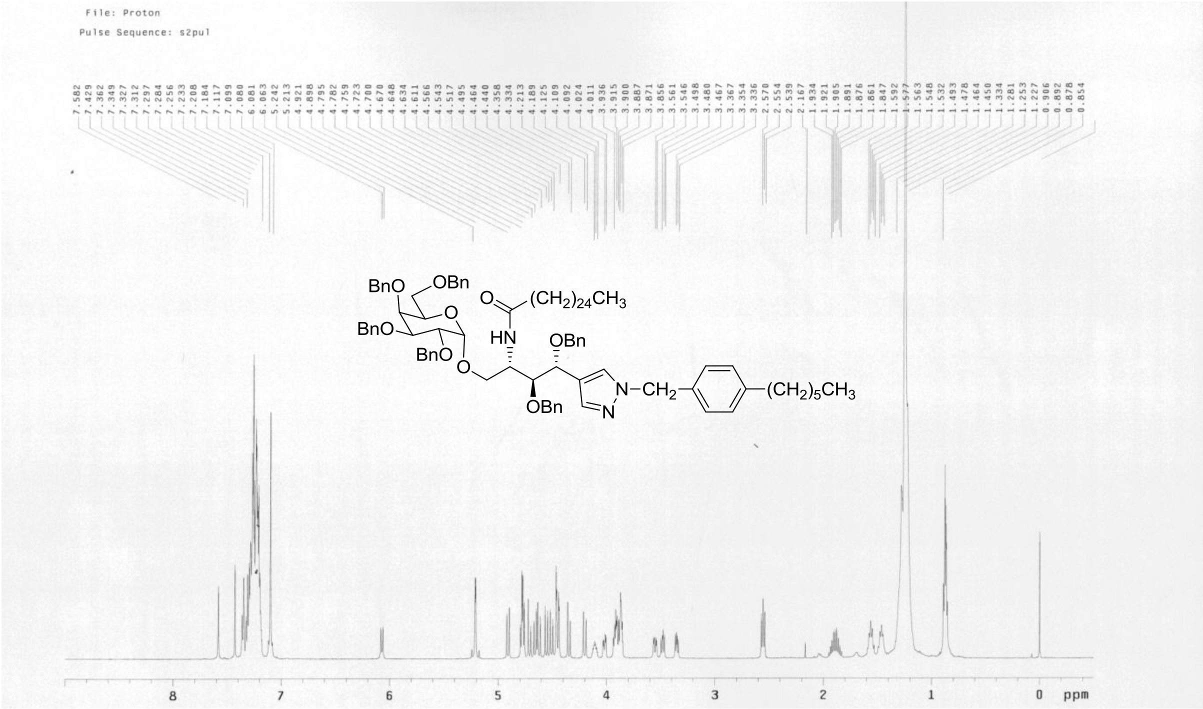
File: Proton
Pulse Sequence: s2pul
BnO
OBn
BnO
BnO
O
O
O
(CH2)24CH3
HN
OBn
OBn
N–CH2
N
(CH2)5CH3
8 7 6 5 4 3 2 1 0 ppm

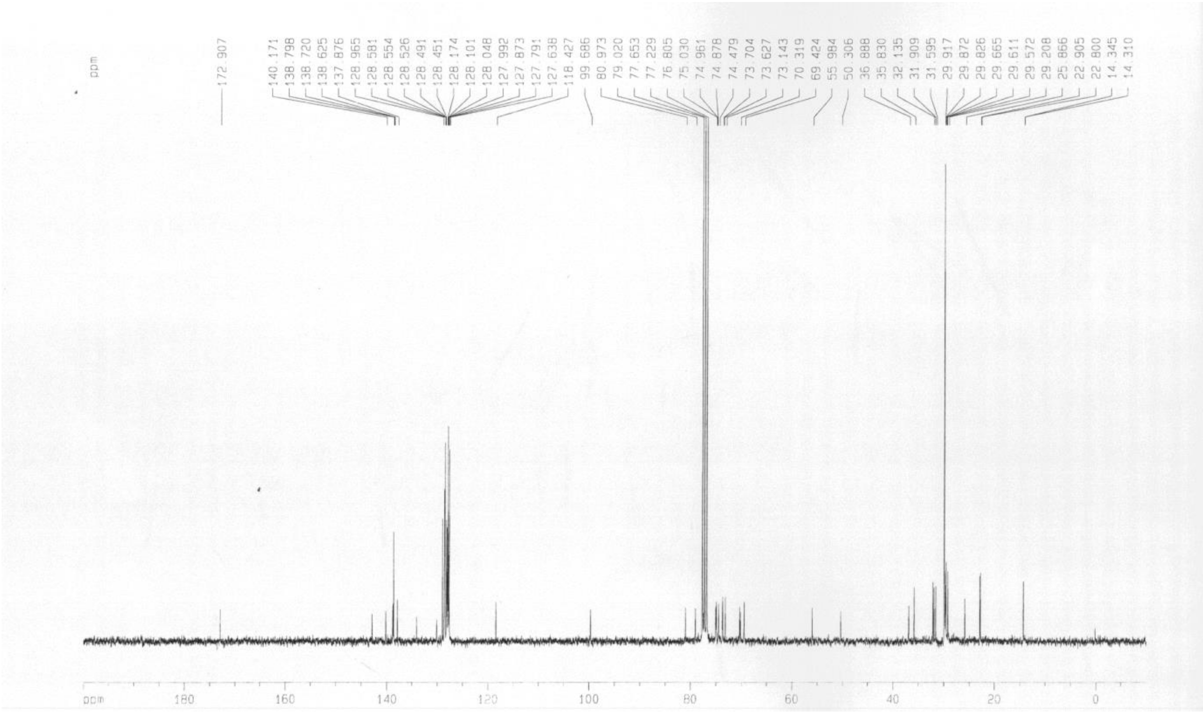
ppm
172.907
140.171
138.798
138.720
138.625
137.876
128.965
128.581
128.554
128.526
128.491
128.451
128.174
128.101
128.048
127.992
127.873
127.791
127.638
118.427
99.686
80.973
79.020
77.653
77.229
76.805
75.030
74.961
74.878
74.479
73.704
73.627
73.143
70.319
69.424
55.984
50.306
36.888
35.830
32.135
31.909
31.595
29.917
29.872
29.826
29.665
29.611
29.572
29.208
25.866
22.905
22.800
14.345
14.310
ppm 180 160 140 120 100 80 60 40 20 0

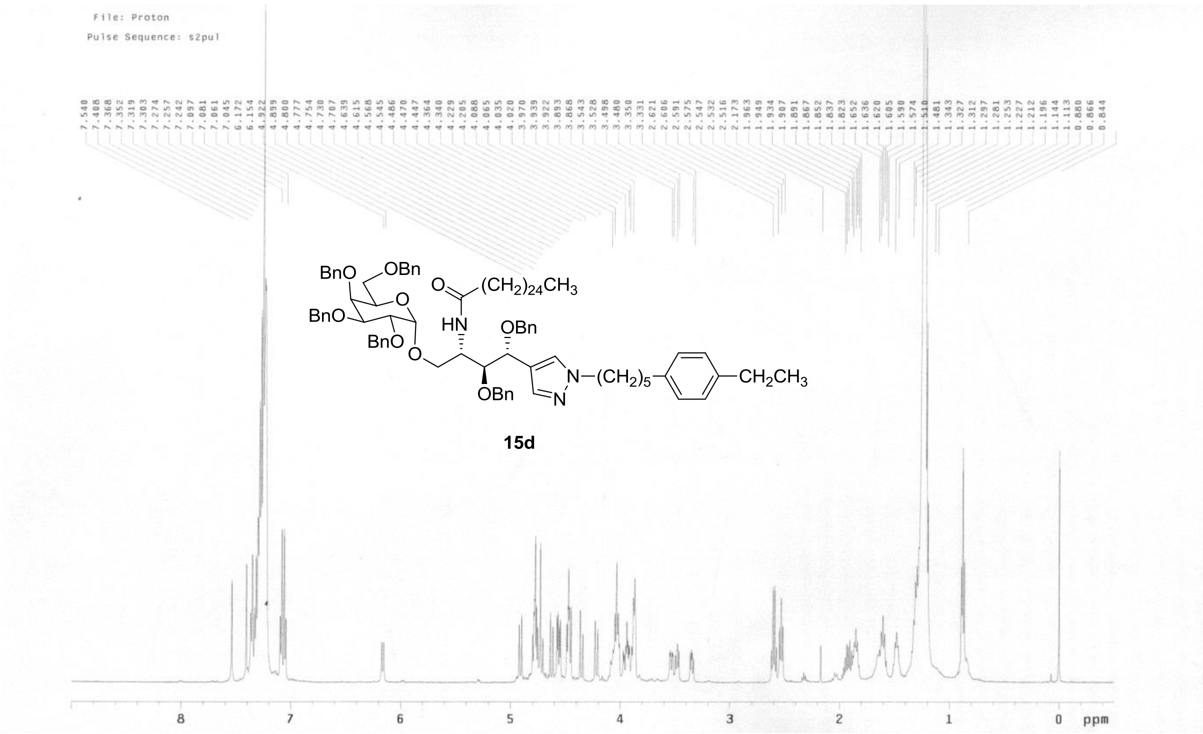
File: Proton
Pulse Sequence: s2pul
7.540
7.408
7.368
7.352
7.319
7.303
7.274
7.257
7.242
7.097
7.081
7.061
7.045
6.172
6.154
4.922
4.899
4.800
4.777
4.754
4.730
4.707
4.639
4.615
4.568
4.545
4.486
4.470
4.447
4.364
4.340
4.229
4.205
4.088
4.065
4.035
4.020
3.970
3.939
3.922
3.893
3.868
3.543
3.528
3.498
3.480
3.350
3.331
2.621
2.606
2.591
2.575
2.547
2.532
2.516
2.173
1.963
1.949
1.934
1.907
1.891
1.867
1.852
1.837
1.823
1.652
1.636
1.620
1.605
1.590
1.574
1.510
1.481
1.343
1.327
1.312
1.297
1.281
1.253
1.227
1.212
1.196
1.144
1.113
0.880
0.866
0.844
BnO
OBn
BnO
BnO
O
O
HN
(CH2)24CH3
OBn
OBn
N
N
(CH2)5
CH2CH3
8 7 6 5 4 3 2 1 0 ppm

15d

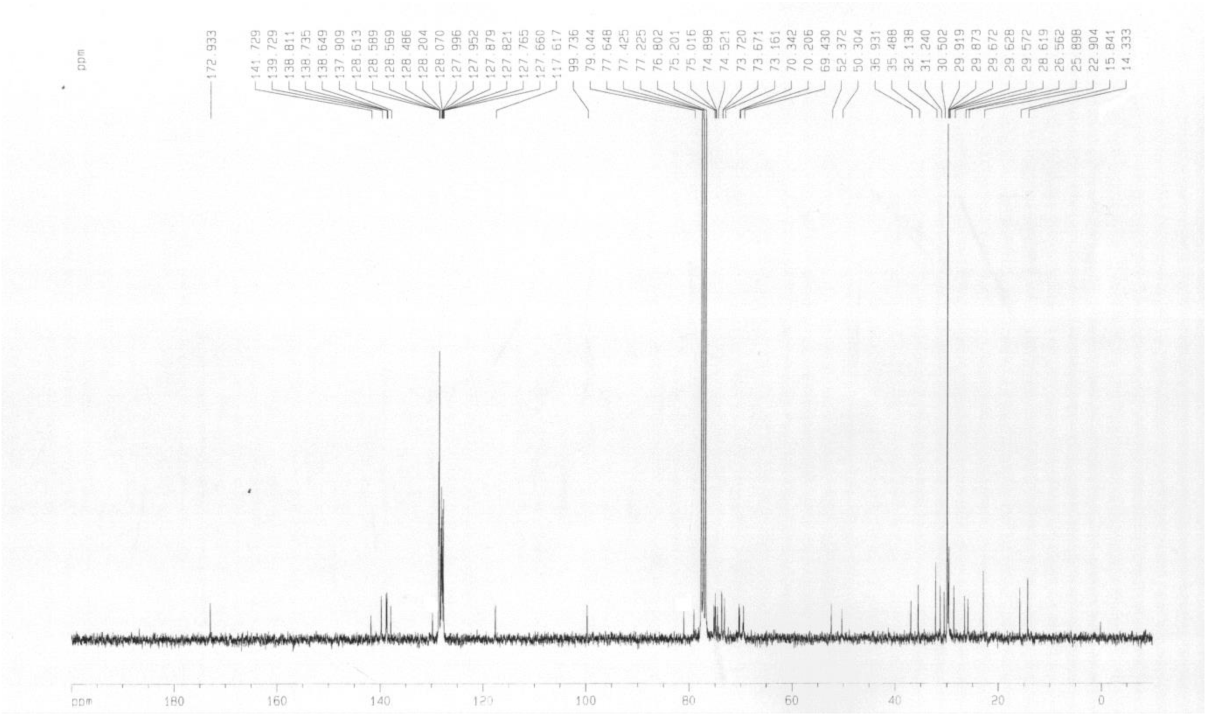
ppm
172.933
141.729
139.729
138.811
138.735
138.649
137.909
128.613
128.589
128.569
128.486
128.204
128.070
127.996
127.952
127.879
127.821
127.765
127.660
117.617
99.736
79.044
77.648
77.425
77.225
76.802
75.201
75.016
74.898
74.521
73.720
73.671
73.161
70.342
70.206
69.430
52.372
50.304
36.931
35.488
32.138
31.240
30.502
29.919
29.873
29.672
29.628
29.572
28.619
26.562
25.898
22.904
15.841
14.333
ppm 180 160 140 120 100 80 60 40 20 0

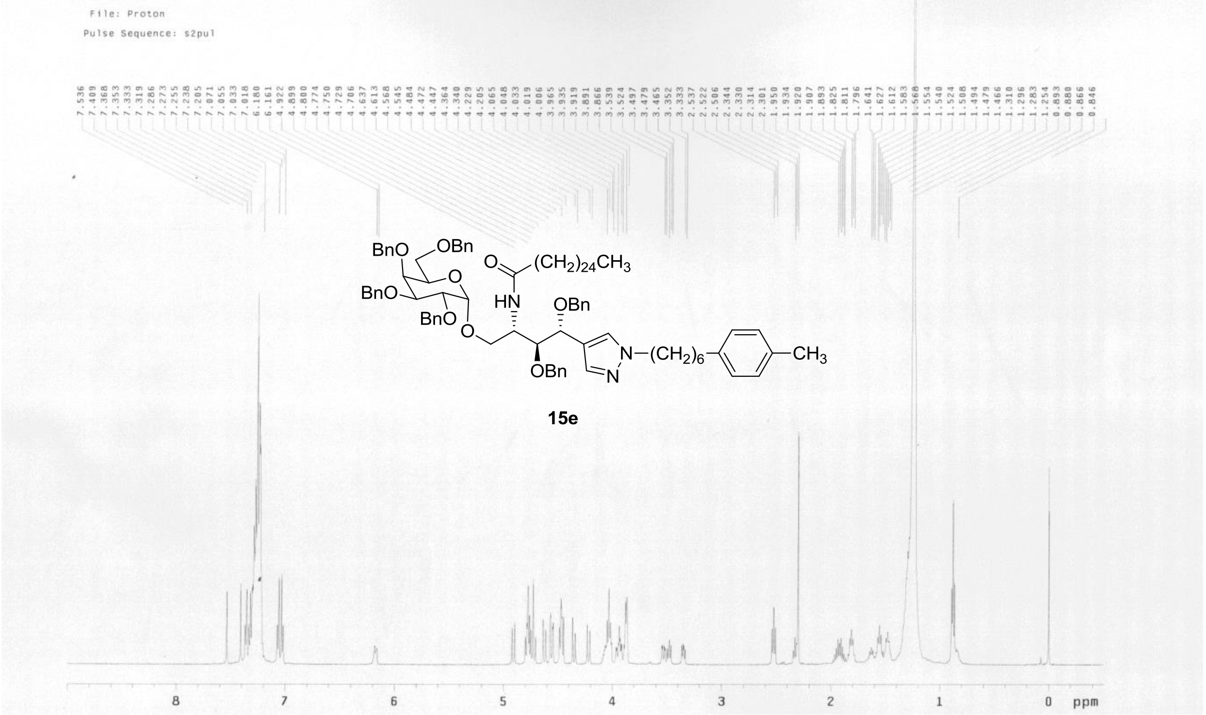
File: Proton
Pulse Sequence: s2pul
BnO
OBn
BnO
BnO
O
O
(CH2)24CH3
HN
OBn
OBn
N
N
(CH2)6
CH3
15e
8 7 6 5 4 3 2 1 0 ppm

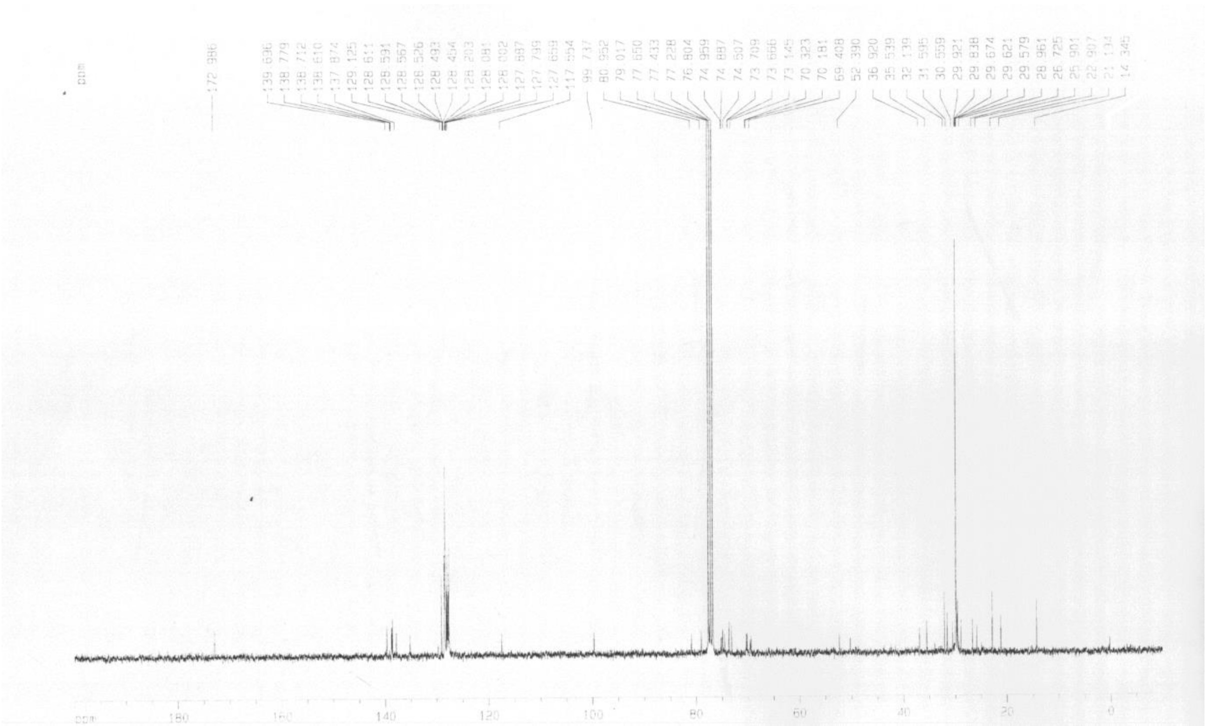

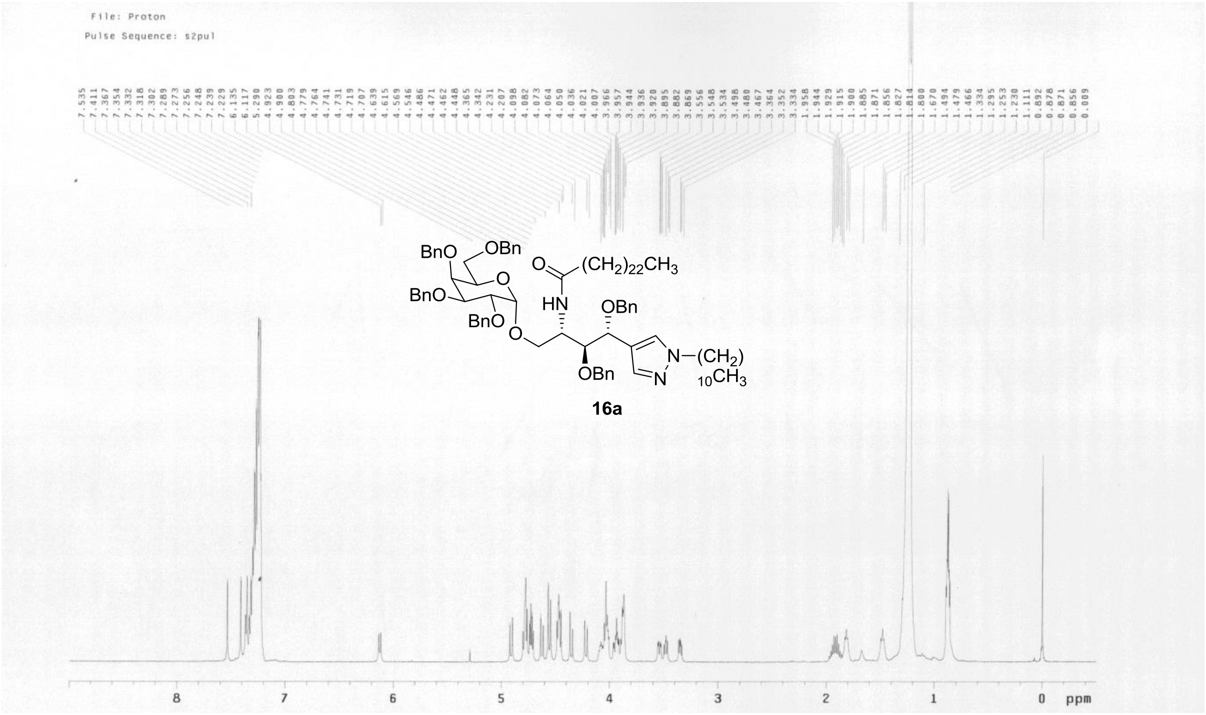
File: Proton
Pulse Sequence: s2pul
BnO
OBn
BnO
O
BnO
O
(CH2)22CH3
HN
OBn
OBn
N
N
(CH2)
10CH3
16a
ppm

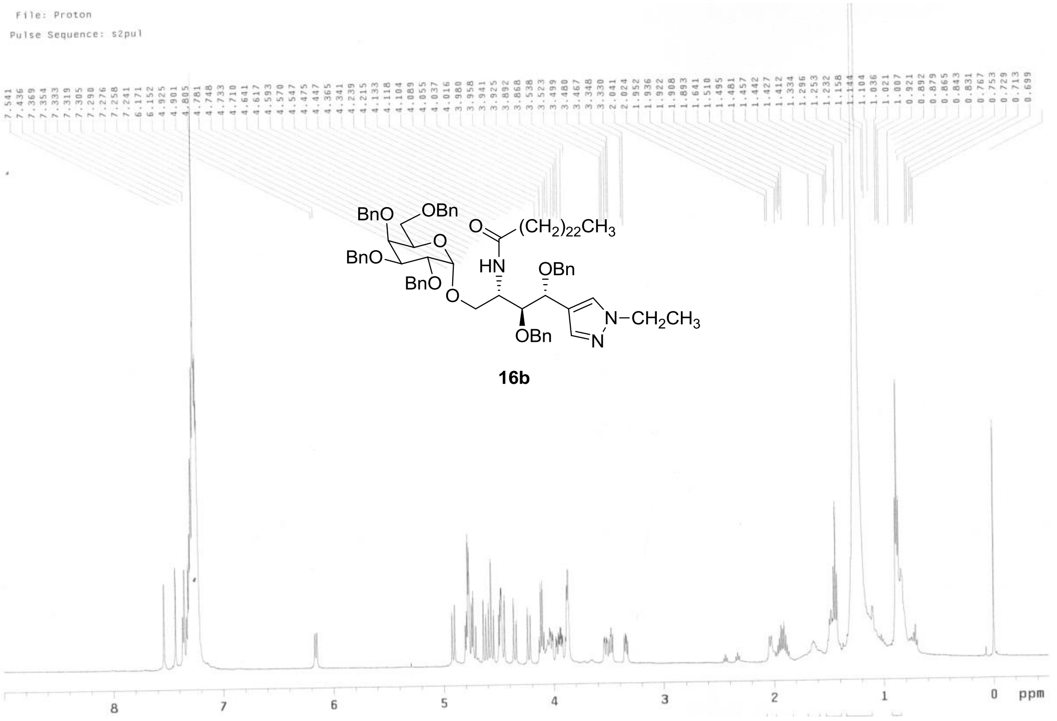
File: Proton
Pulse Sequence: s2pul
7.541 7.436 7.369 7.354 7.333 7.319 7.305 7.290 7.276 7.258 7.241 6.171 6.152 4.925 4.901 4.805 4.781 4.748 4.733 4.710 4.641 4.617 4.593 4.570 4.547 4.475 4.447 4.365 4.341 4.239 4.215 4.133 4.118 4.104 4.089 4.055 4.037 4.016 3.980 3.958 3.941 3.925 3.892 3.868 3.538 3.523 3.499 3.480 3.467 3.348 3.330 2.041 2.024 1.952 1.936 1.922 1.908 1.893 1.641 1.510 1.495 1.481 1.457 1.442 1.427 1.412 1.334 1.296 1.253 1.232 1.158 1.144 1.104 1.036 1.021 1.007 0.921 0.892 0.879 0.865 0.843 0.831 0.767 0.753 0.729 0.713 0.699
BnO
OBn
BnO
BnO
O
O
(CH2)22CH3
HN
OBn
OBn
N–CH2CH3
N
16b
8 7 6 5 4 3 2 1 0 ppm

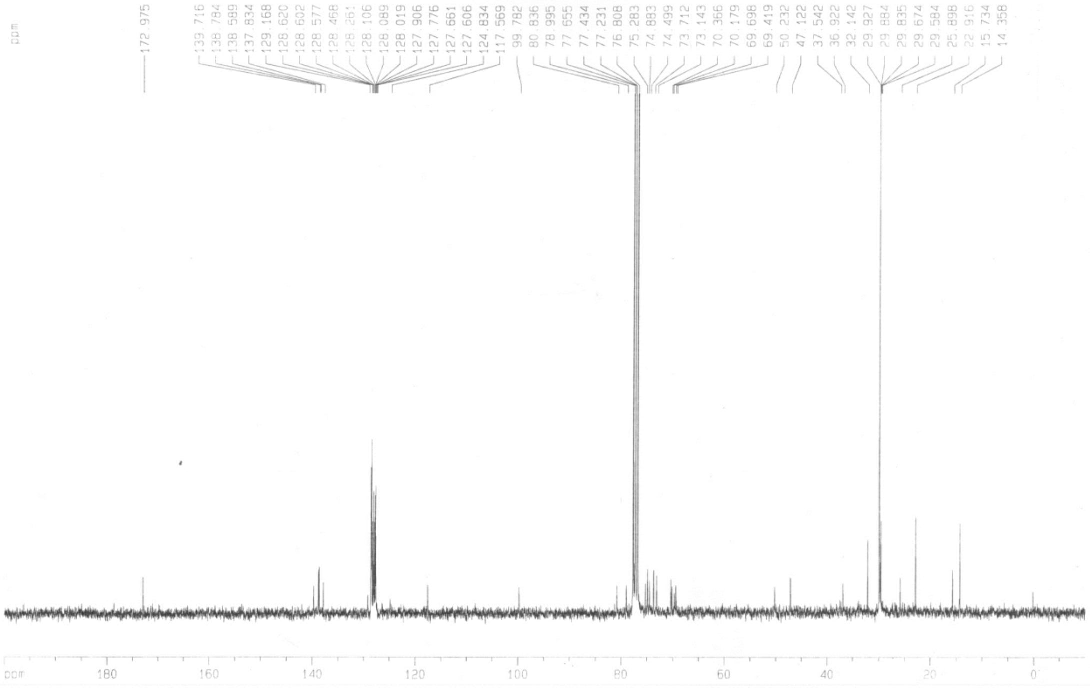
ppm
172.975
139.716 138.784 138.589 137.834 129.168 128.620 128.602 128.577 128.458 128.261 128.106 128.089 128.019 127.906 127.776 127.661 127.606 124.834 117.569 99.782 80.836 78.995 77.655 77.434 77.231 76.808 75.283 74.883 74.499 73.712 73.143 70.366 70.179 69.698 69.419 50.232 47.122 37.542 36.922 32.142 29.927 29.884 29.835 29.674 29.584 25.898 22.916 15.734 14.358
ppm 180 160 140 120 100 80 60 40 20 0

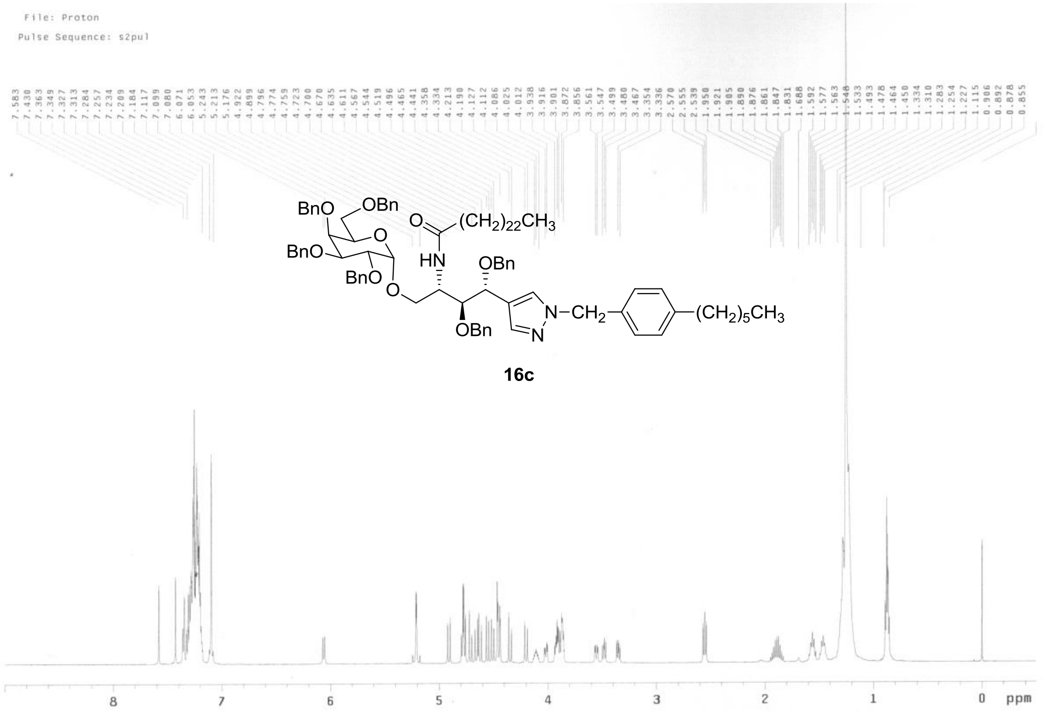
File: Proton
Pulse Sequence: s2pul
BnO
OBn
BnO
BnO
O
O
HN
O
(CH2)22CH3
OBn
OBn
N
N
CH2
(CH2)5CH3
16c
8
7
6
5
4
3
2
1
0
ppm

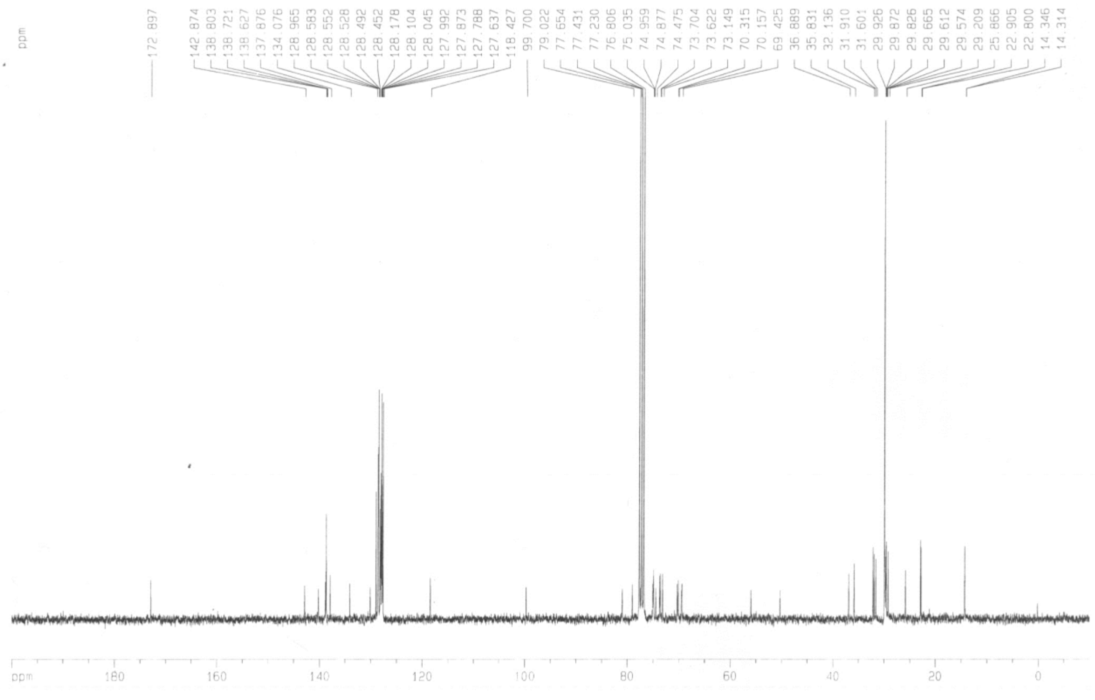
ppm
172.897
142.874
138.803
138.721
138.627
137.876
134.076
128.965
128.583
128.552
128.528
128.492
128.452
128.178
128.104
128.045
127.992
127.873
127.788
127.637
118.427
99.700
79.022
77.654
77.431
77.230
76.806
75.035
74.959
74.877
74.475
73.704
73.622
73.149
70.315
70.157
69.425
36.889
35.831
32.136
31.910
31.601
29.926
29.872
29.826
29.665
29.612
29.574
29.209
25.866
22.905
22.800
14.346
14.314
ppm
180
160
140
120
100
80
60
40
20
0

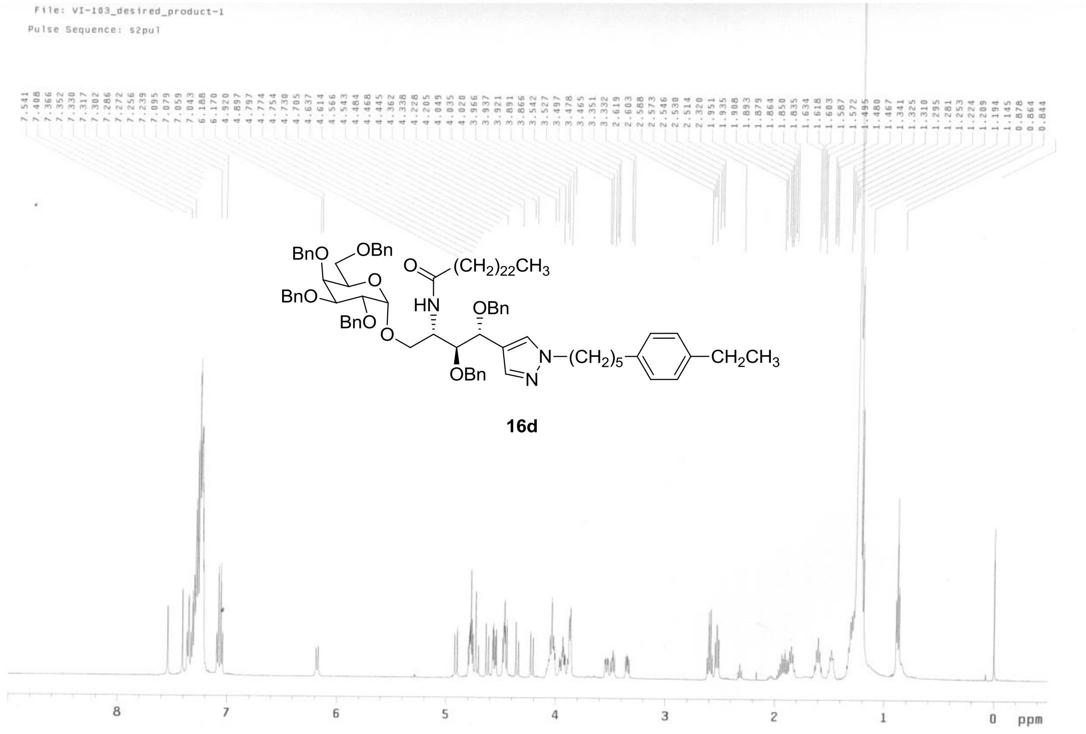
File: VI-103_desired_product-1
Pulse Sequence: s2pul
16d
ppm

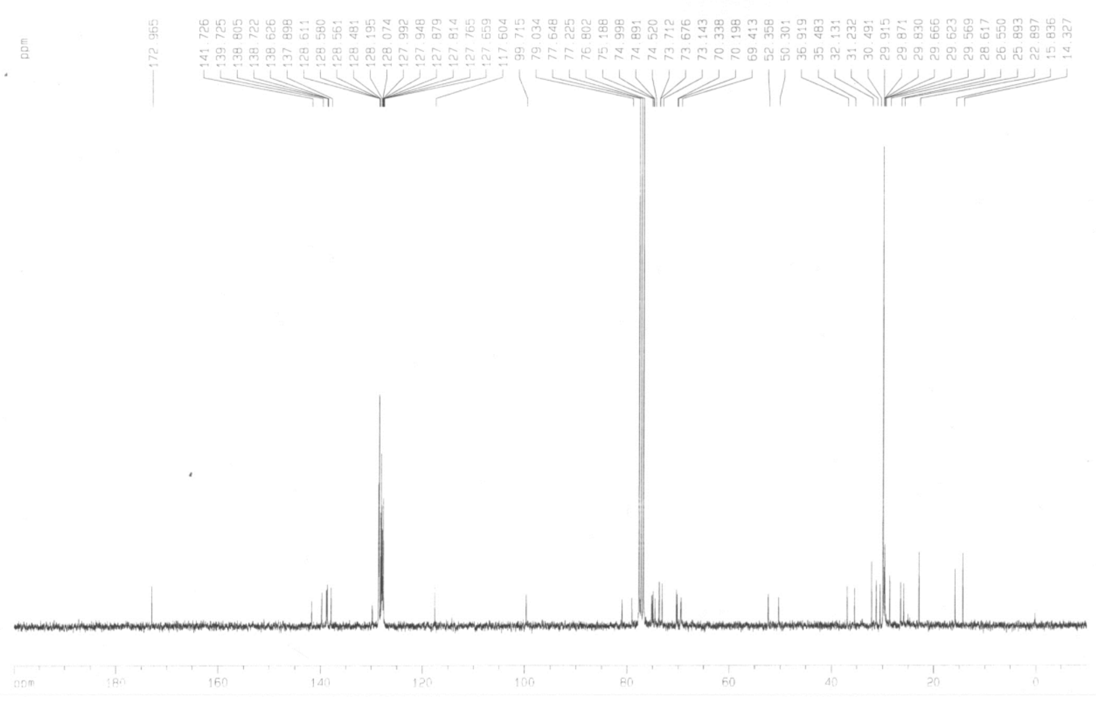
ppm

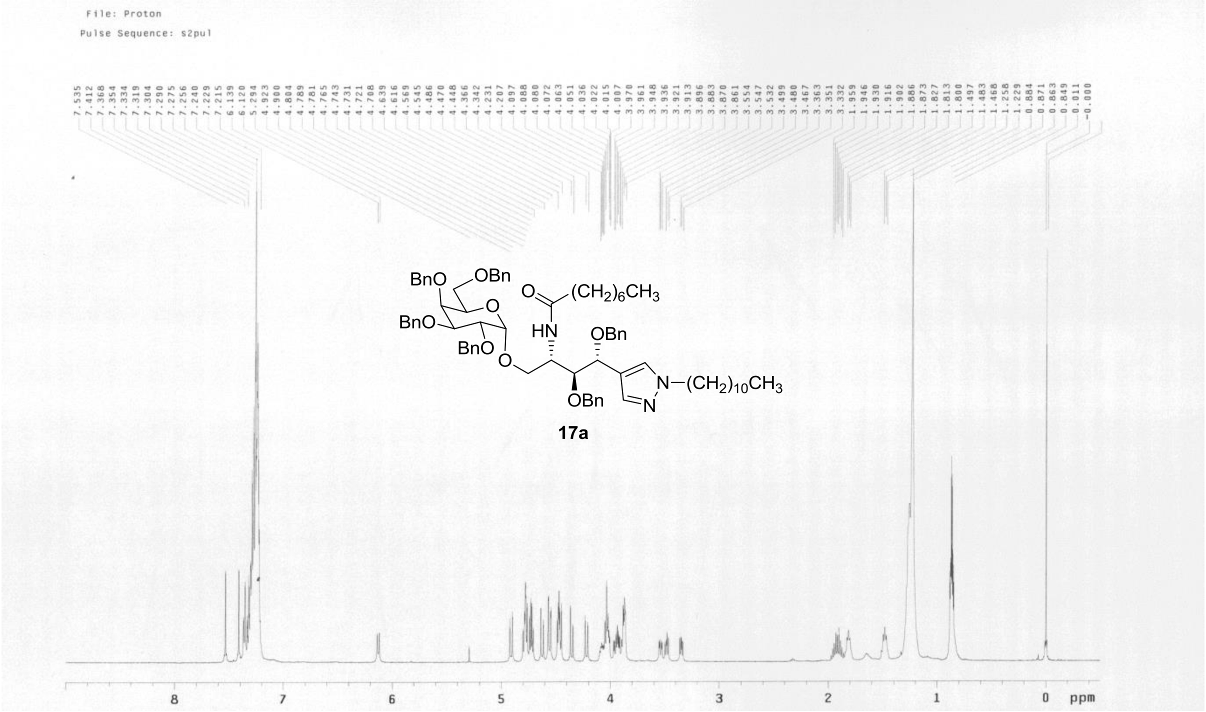
File: Proton
Pulse Sequence: s2pul
17a
ppm

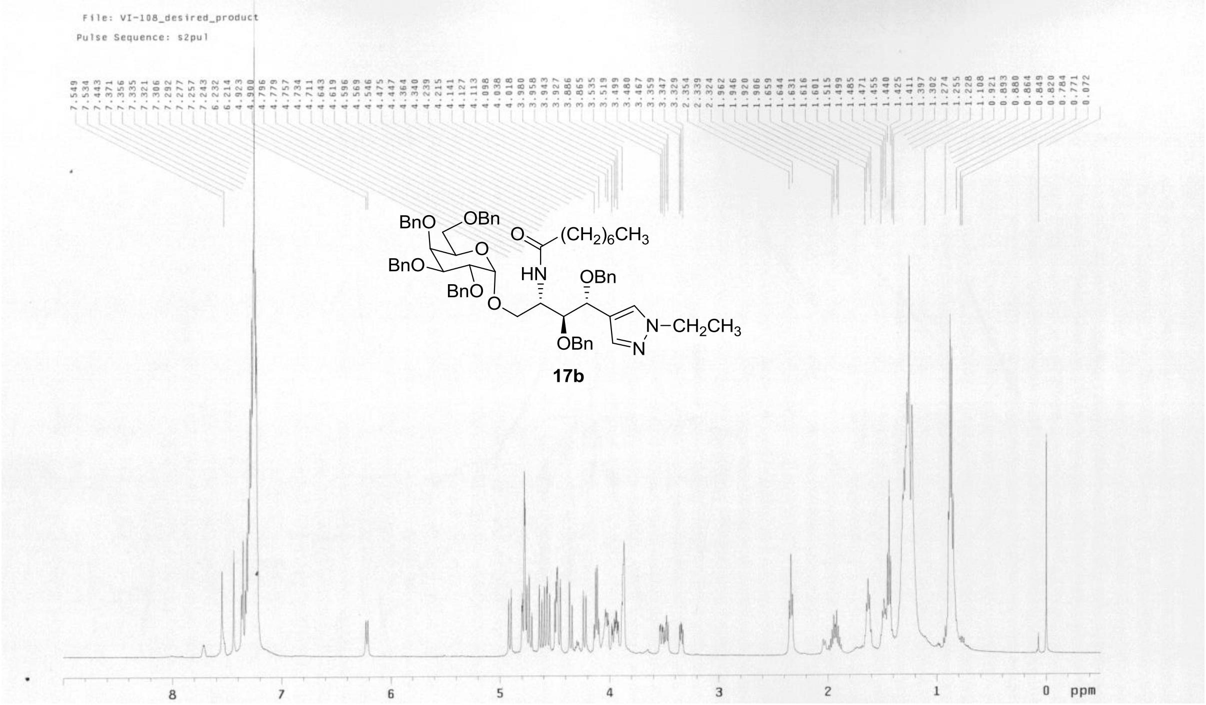
File: VI-108_desired_product
Pulse Sequence: s2pul
17b
ppm

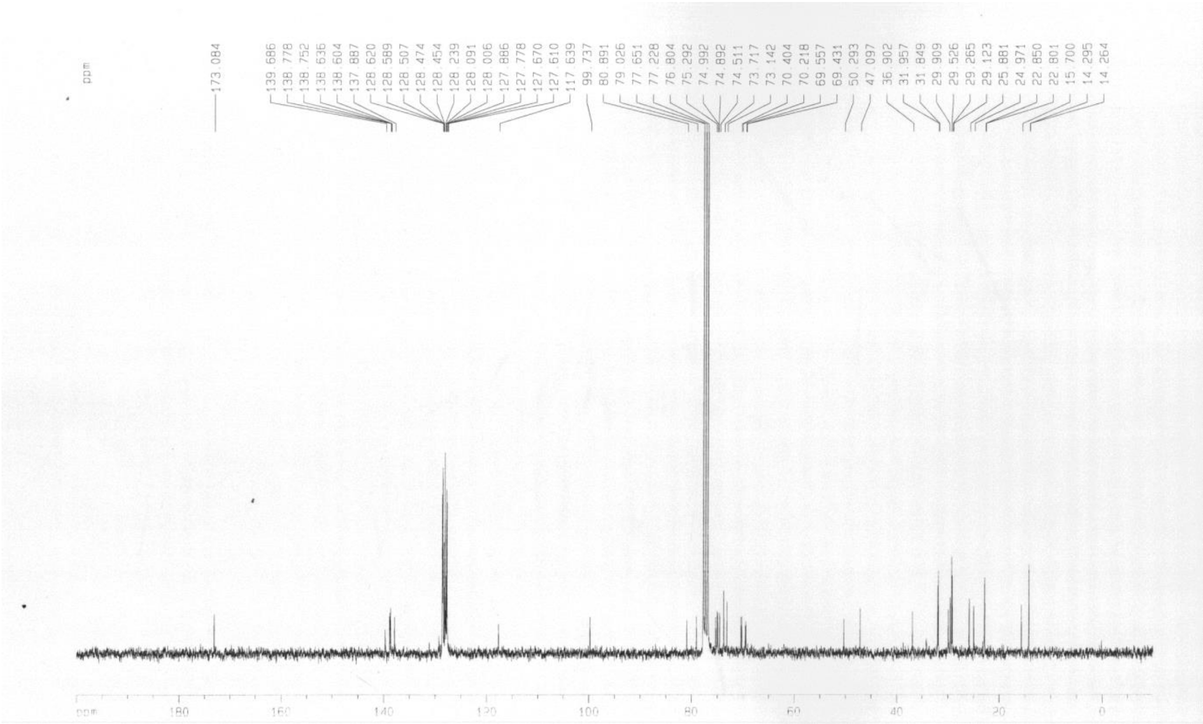
ppm
173.084
139.686
138.778
138.752
138.636
138.604
137.887
128.620
128.589
128.507
128.474
128.454
128.239
128.091
128.006
127.886
127.778
127.670
127.610
117.639
99.737
80.891
79.026
77.651
77.228
76.804
75.292
74.992
74.892
74.511
73.717
73.142
70.404
70.218
69.557
69.431
50.293
47.097
36.902
31.957
31.849
29.909
29.526
29.265
29.123
25.881
24.971
22.850
22.801
15.700
14.295
14.264
ppm 180 160 140 120 100 80 60 40 20 0

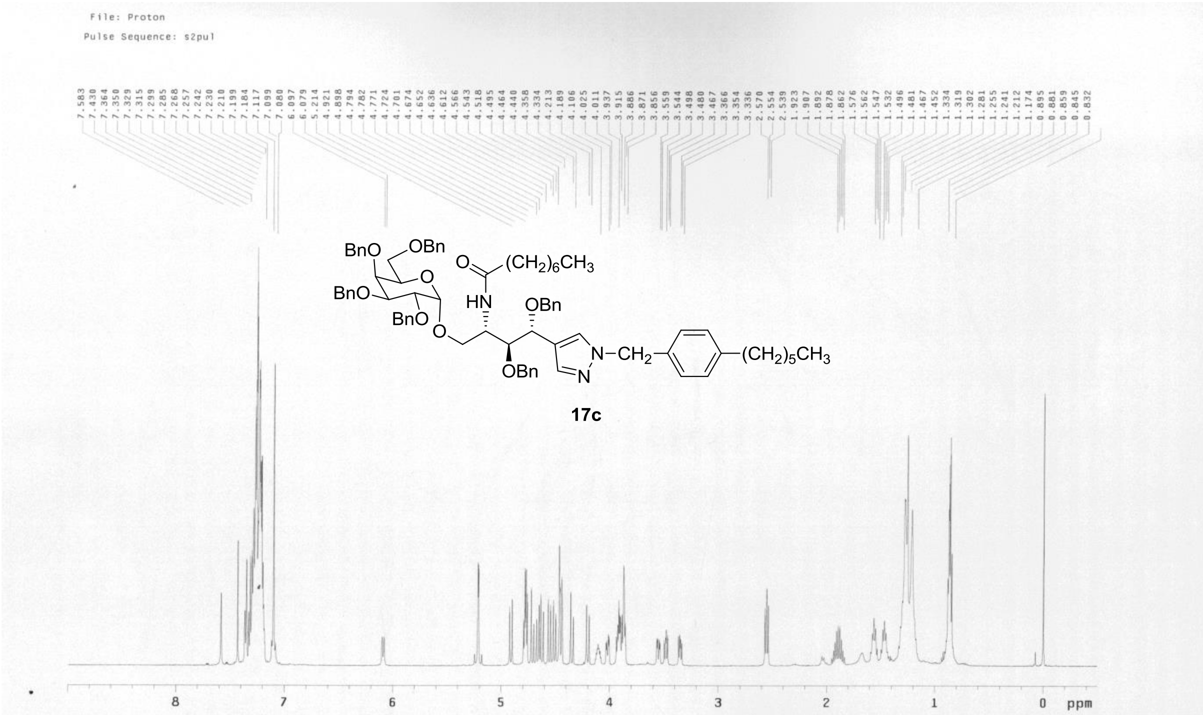
File: Proton
Pulse Sequence: s2pul
7.583
7.430
7.364
7.350
7.329
7.315
7.299
7.285
7.268
7.257
7.242
7.230
7.210
7.199
7.184
7.117
7.099
7.080
6.097
6.079
5.214
4.921
4.898
4.794
4.782
4.771
4.724
4.701
4.674
4.652
4.636
4.612
4.566
4.543
4.518
4.495
4.464
4.440
4.358
4.334
4.213
4.189
4.106
4.025
4.011
3.937
3.915
3.886
3.871
3.856
3.559
3.544
3.498
3.480
3.367
3.354
3.336
2.570
2.554
2.539
1.923
1.907
1.892
1.878
1.862
1.576
1.562
1.547
1.532
1.496
1.481
1.467
1.452
1.334
1.319
1.302
1.281
1.255
1.241
1.212
1.174
0.895
0.881
0.859
0.845
0.832
BnO
OBn
BnO
BnO
O
O
HN
(CH2)6CH3
OBn
OBn
N-CH2
N
(CH2)5CH3
17c
8 7 6 5 4 3 2 1 0 ppm

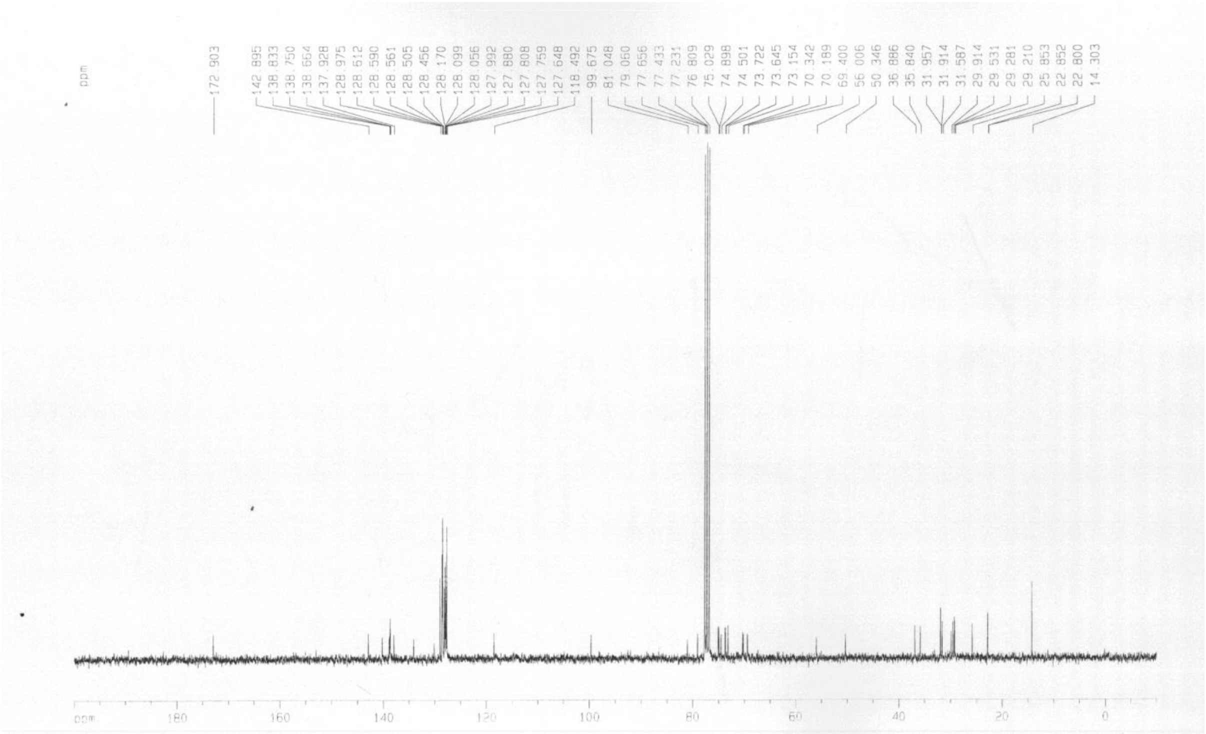
ppm
172.903
142.895
138.833
138.750
138.664
137.928
128.975
128.612
128.590
128.561
128.505
128.456
128.170
128.099
128.056
127.992
127.880
127.808
127.759
127.648
118.492
99.675
81.048
79.060
77.656
77.433
77.231
76.809
75.029
74.898
74.501
73.722
73.645
73.154
70.342
70.189
69.400
56.006
50.346
36.886
35.840
31.957
31.914
31.587
29.914
29.531
29.281
29.210
25.853
22.852
22.800
14.303
ppm 180 160 140 120 100 80 60 40 20 0

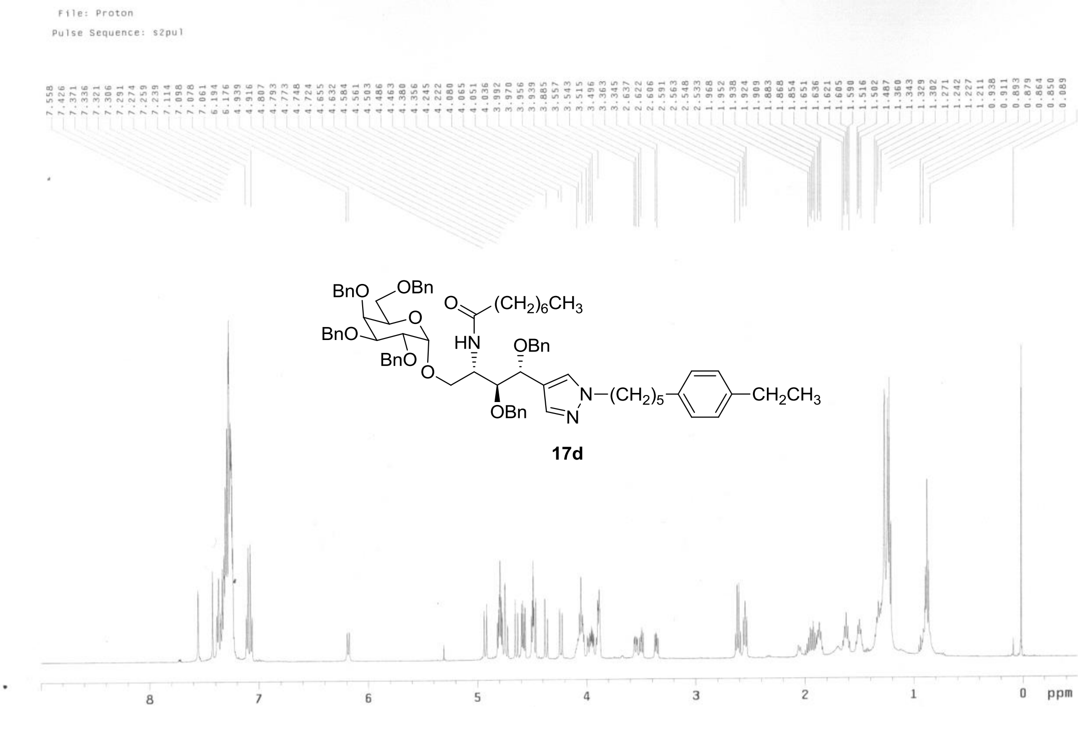
File: Proton
Pulse Sequence: s2pul
7.558
7.426
7.371
7.336
7.321
7.306
7.291
7.274
7.259
7.239
7.114
7.098
7.078
7.061
6.194
6.176
4.939
4.916
4.807
4.793
4.773
4.748
4.724
4.655
4.632
4.584
4.561
4.503
4.486
4.463
4.380
4.356
4.245
4.222
4.080
4.065
4.051
4.036
3.992
3.970
3.956
3.939
3.885
3.557
3.543
3.515
3.496
3.363
3.345
2.637
2.622
2.606
2.591
2.563
2.548
2.533
1.968
1.952
1.938
1.924
1.909
1.883
1.868
1.854
1.651
1.636
1.621
1.605
1.590
1.516
1.502
1.487
1.360
1.343
1.329
1.302
1.271
1.242
1.227
1.211
0.938
0.911
0.893
0.879
0.864
0.850
0.089
BnO
OBn
BnO
BnO
O
O
O
$(CH_2)_6CH_3$
HN
OBn
OBn
N
N
$(CH_2)_5$
CH_2CH_3
17d
8 7 6 5 4 3 2 1 0 ppm

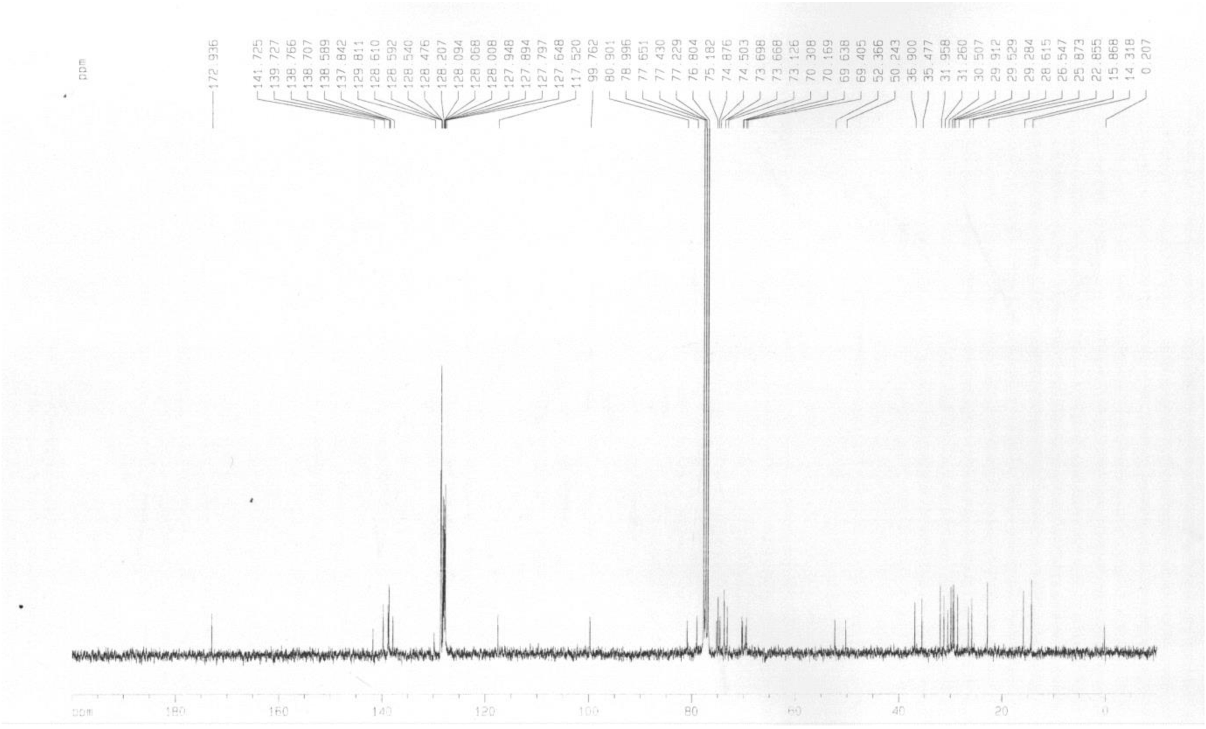
ppm
180
160
140
120
100
80
60
40
20
0

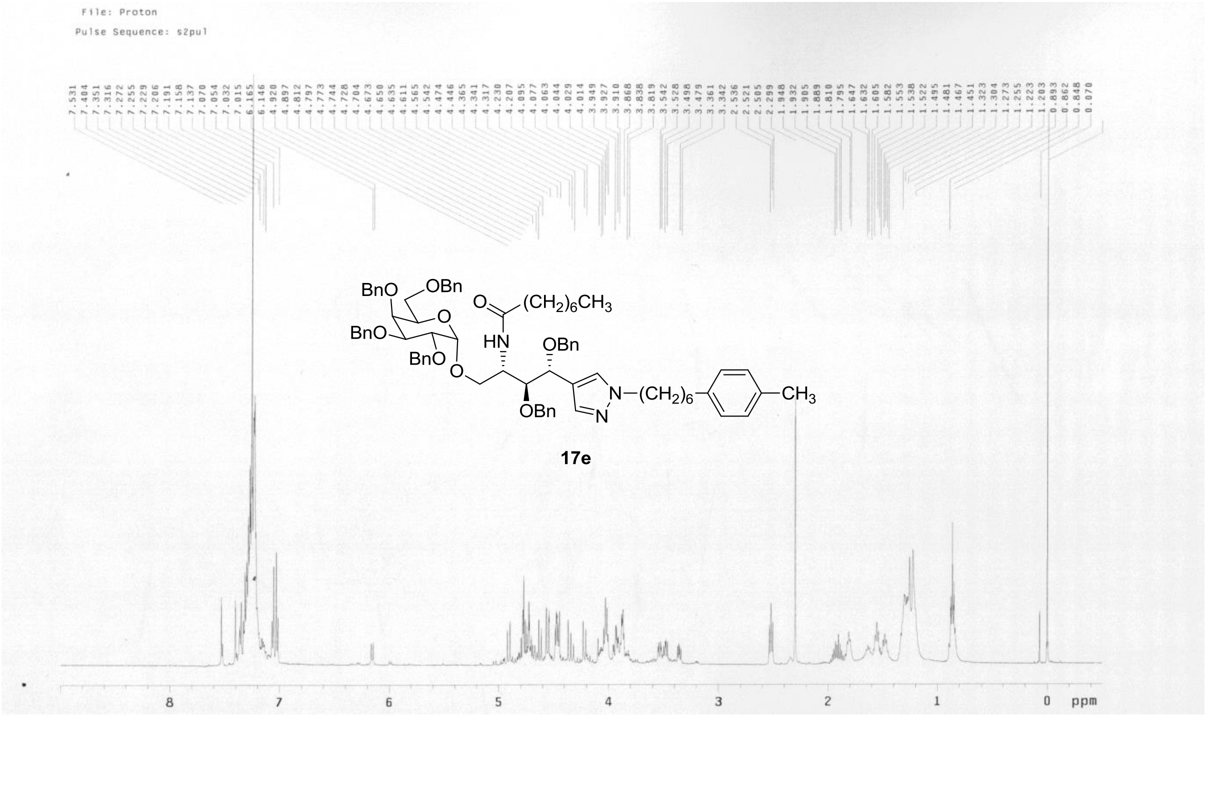
File: Proton
Pulse Sequence: s2pul
8
7
6
5
4
3
2
1
0
ppm

17e

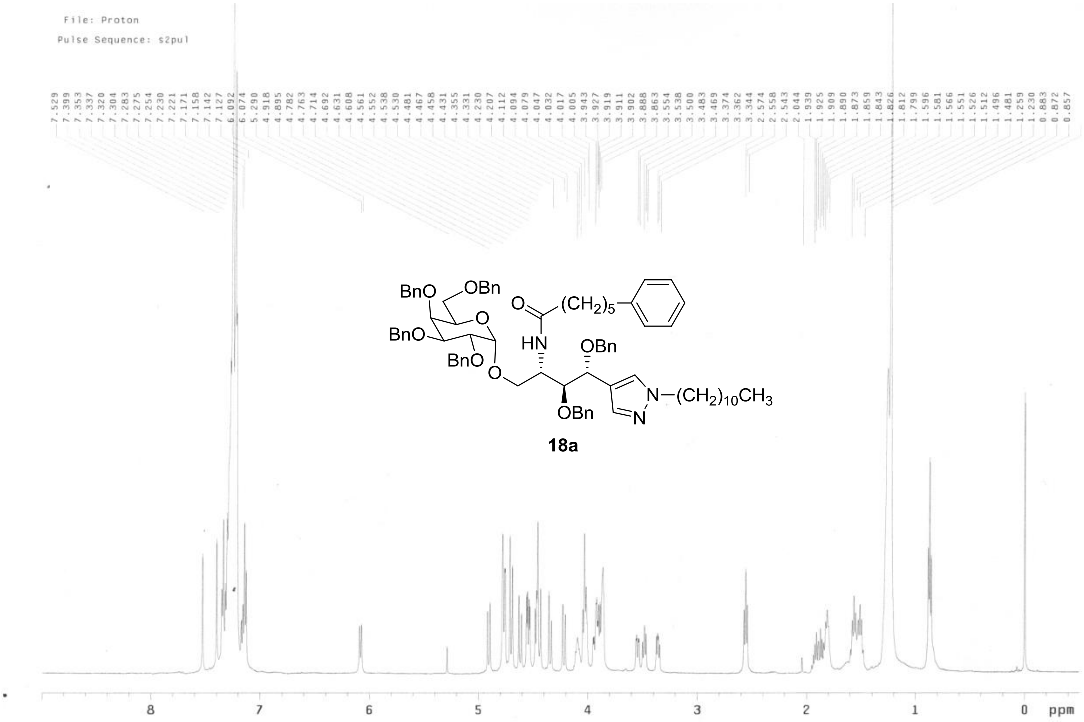
File: Proton
Pulse Sequence: s2pul
18a
8 7 6 5 4 3 2 1 0 ppm

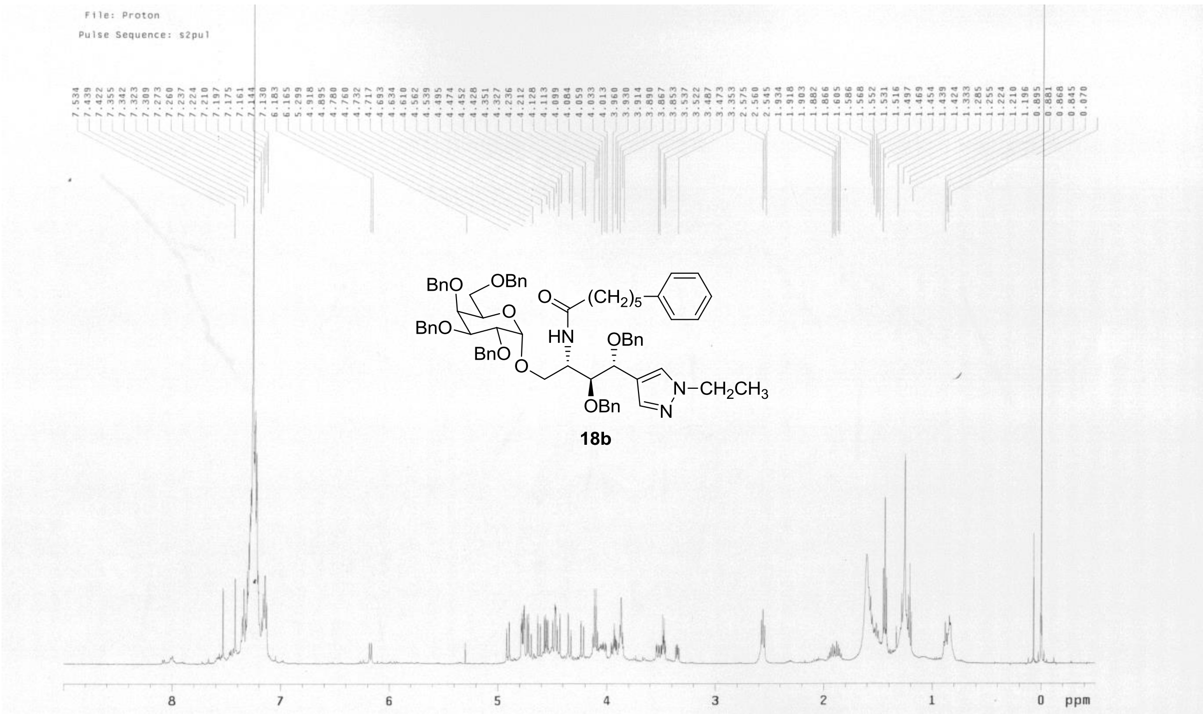
File: Proton
Pulse Sequence: s2pul
18b
8 7 6 5 4 3 2 1 0 ppm

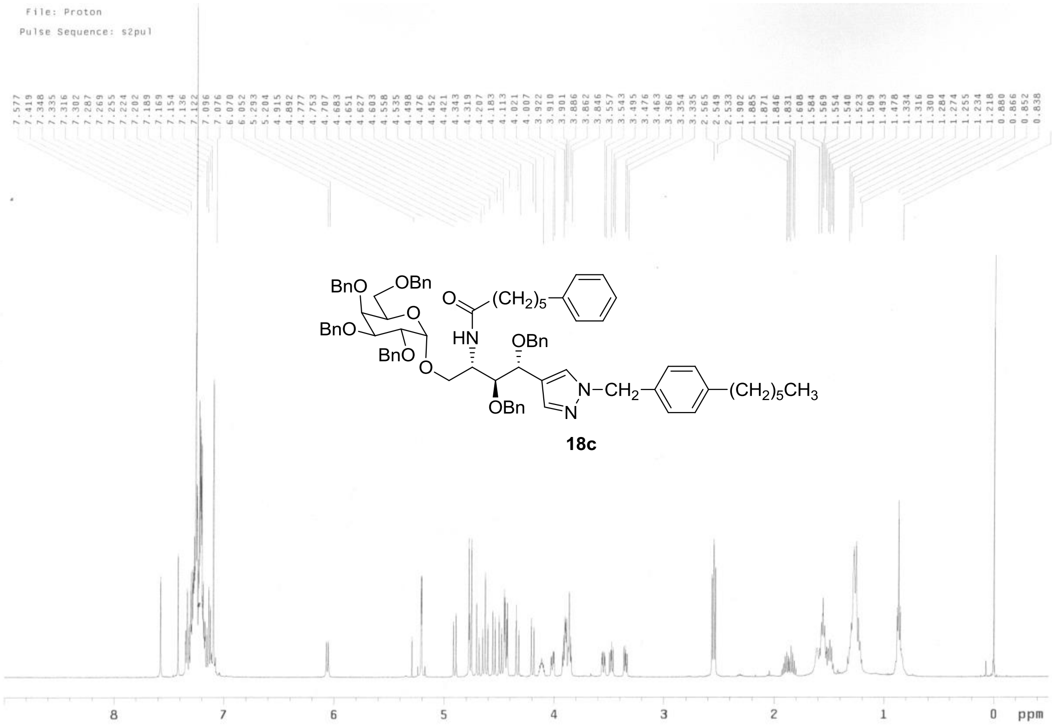
File: Proton
Pulse Sequence: s2pul
18c
ppm

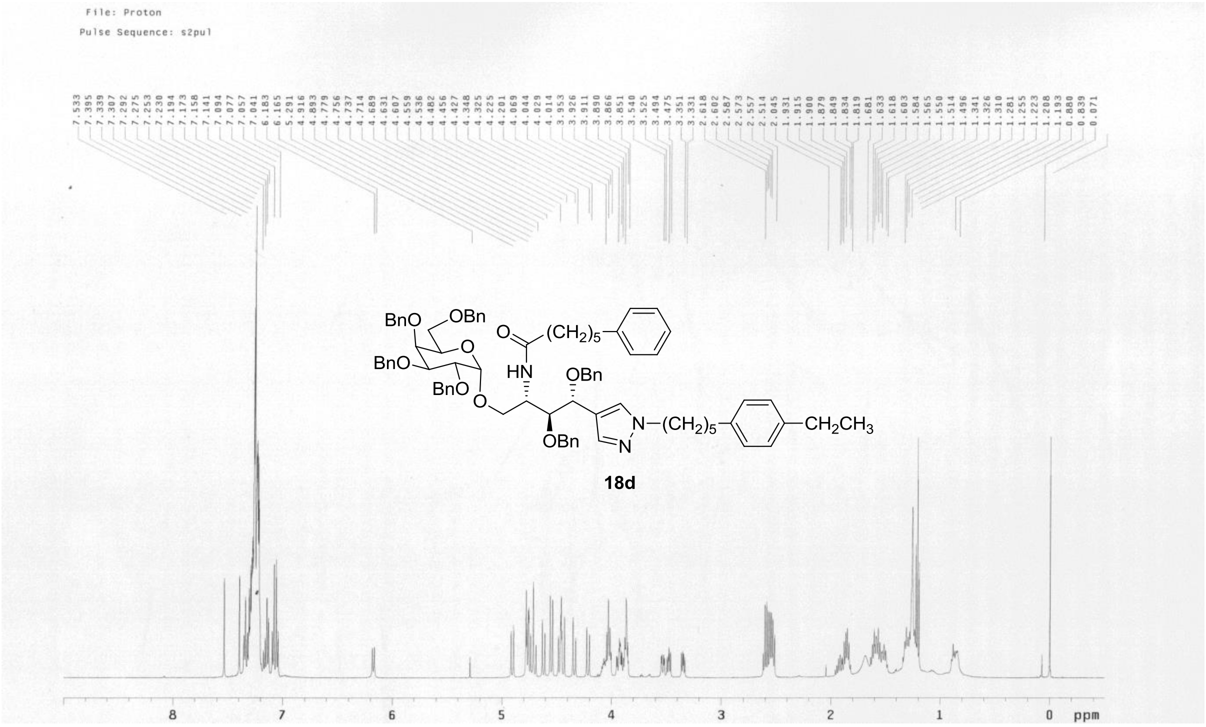
File: Proton
Pulse Sequence: s2pul
18d
ppm

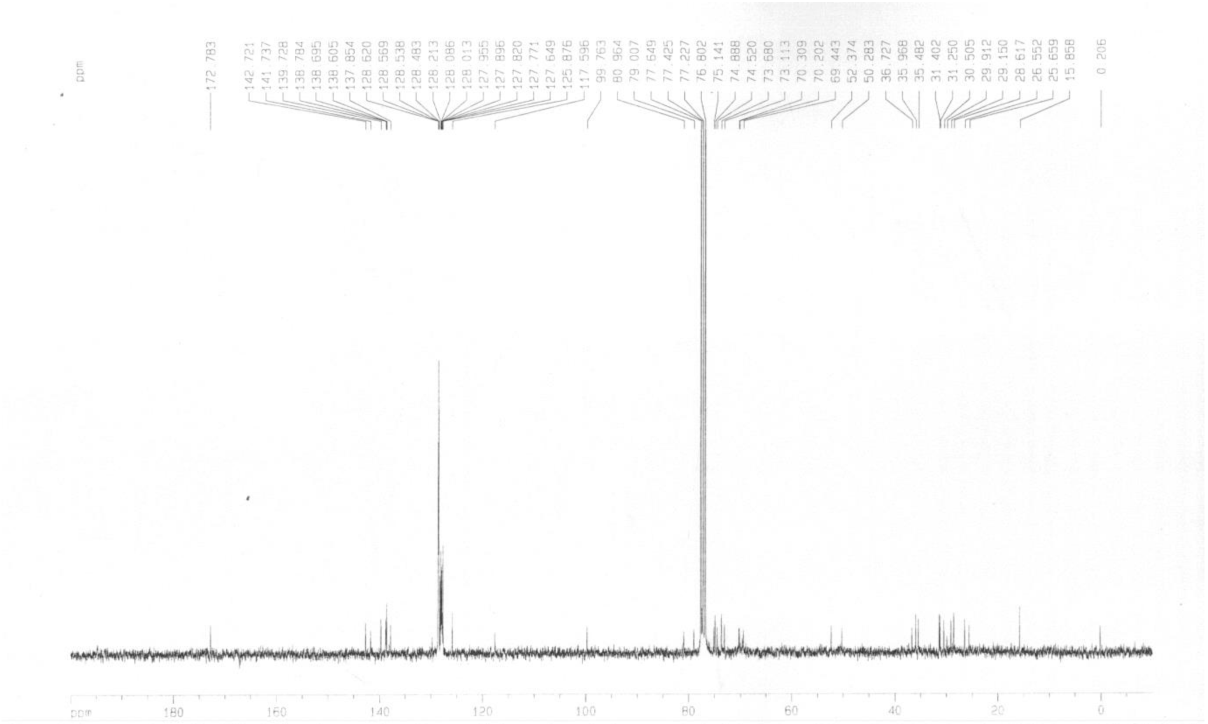
ppm
172.783
142.721
141.737
139.728
138.784
138.695
138.605
137.854
128.620
128.569
128.538
128.483
128.213
128.086
128.013
127.955
127.896
127.820
127.771
127.649
125.876
117.596
99.763
80.964
79.007
77.649
77.425
77.227
76.802
75.141
74.888
74.520
73.680
73.113
70.309
70.202
69.443
52.374
50.283
36.727
35.968
35.482
31.402
31.250
30.505
29.912
29.150
28.617
26.552
25.659
15.858
0.206
ppm 180 160 140 120 100 80 60 40 20 0

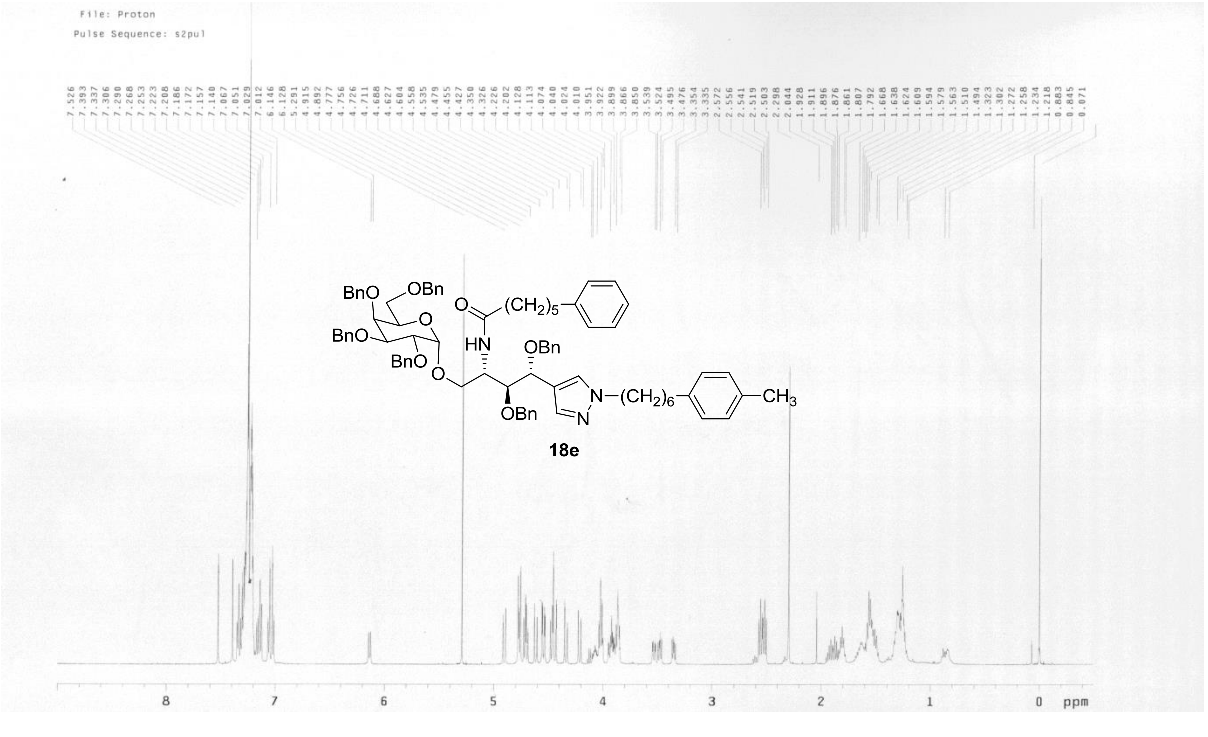
File: Proton
Pulse Sequence: s2pul
7.526
7.393
7.337
7.306
7.290
7.268
7.253
7.223
7.208
7.186
7.172
7.157
7.140
7.067
7.051
7.029
7.012
6.146
6.128
5.291
4.915
4.892
4.777
4.756
4.726
4.711
4.688
4.627
4.604
4.558
4.535
4.479
4.455
4.427
4.350
4.326
4.226
4.202
4.128
4.113
4.074
4.040
4.024
4.010
3.951
3.922
3.899
3.866
3.850
3.539
3.524
3.495
3.476
3.354
3.335
2.572
2.556
2.541
2.519
2.503
2.298
2.044
1.928
1.911
1.896
1.876
1.861
1.807
1.792
1.668
1.638
1.624
1.609
1.594
1.579
1.563
1.510
1.494
1.323
1.302
1.272
1.258
1.234
1.218
0.883
0.845
0.071
BnO
OBn
BnO
BnO
O
O
O
HN
(CH2)5
OBn
OBn
N
N
(CH2)6
CH3
18e
8 7 6 5 4 3 2 1 0 ppm

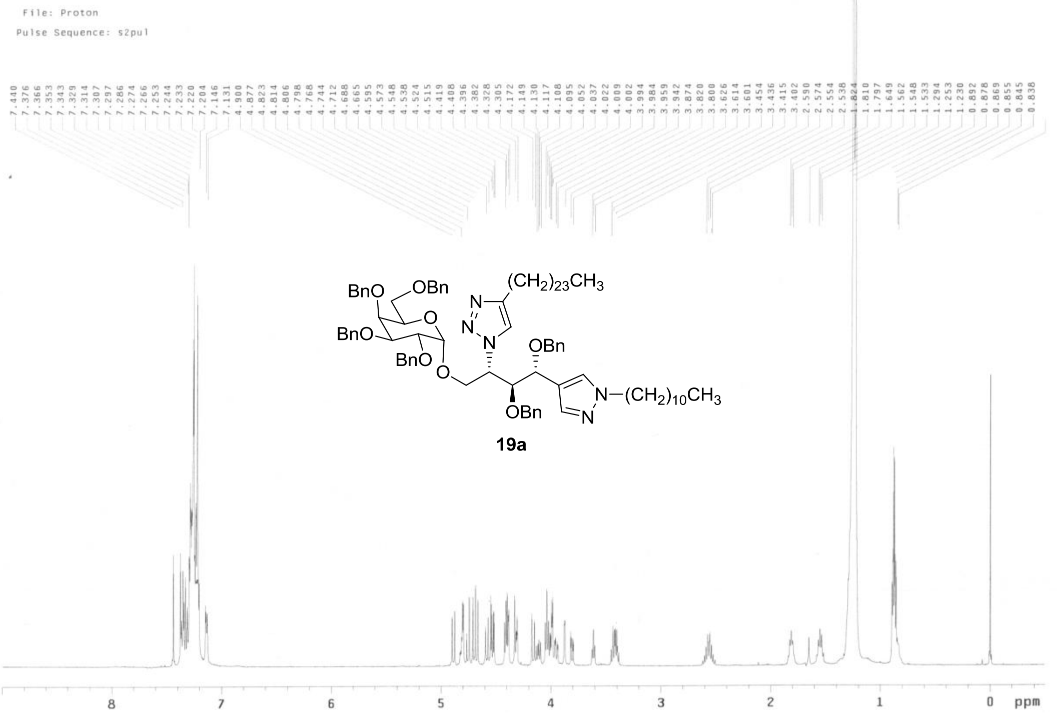
File: Proton
Pulse Sequence: s2pul
BnO
OBn
BnO
BnO
N
N
N
(CH2)23CH3
OBn
O
OBn
N
N
(CH2)10CH3
19a
8
7
6
5
4
3
2
1
0
ppm

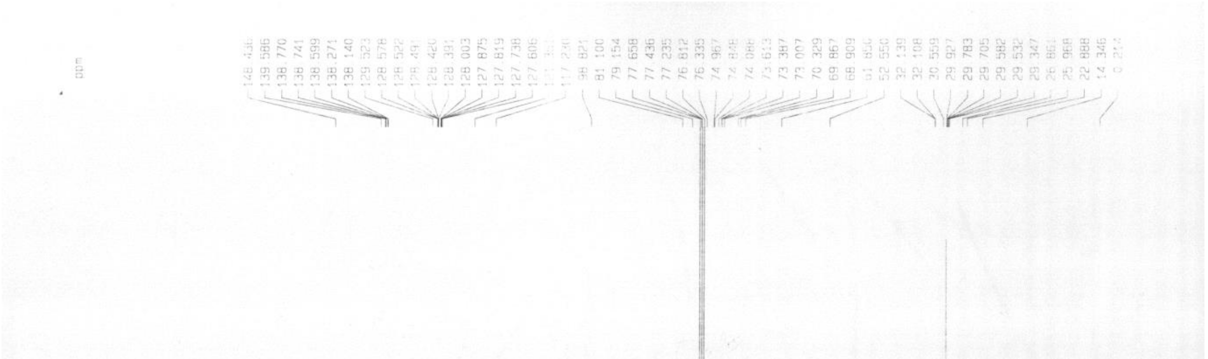
ppm
180
160
140
120
100
80
60
40
20
0

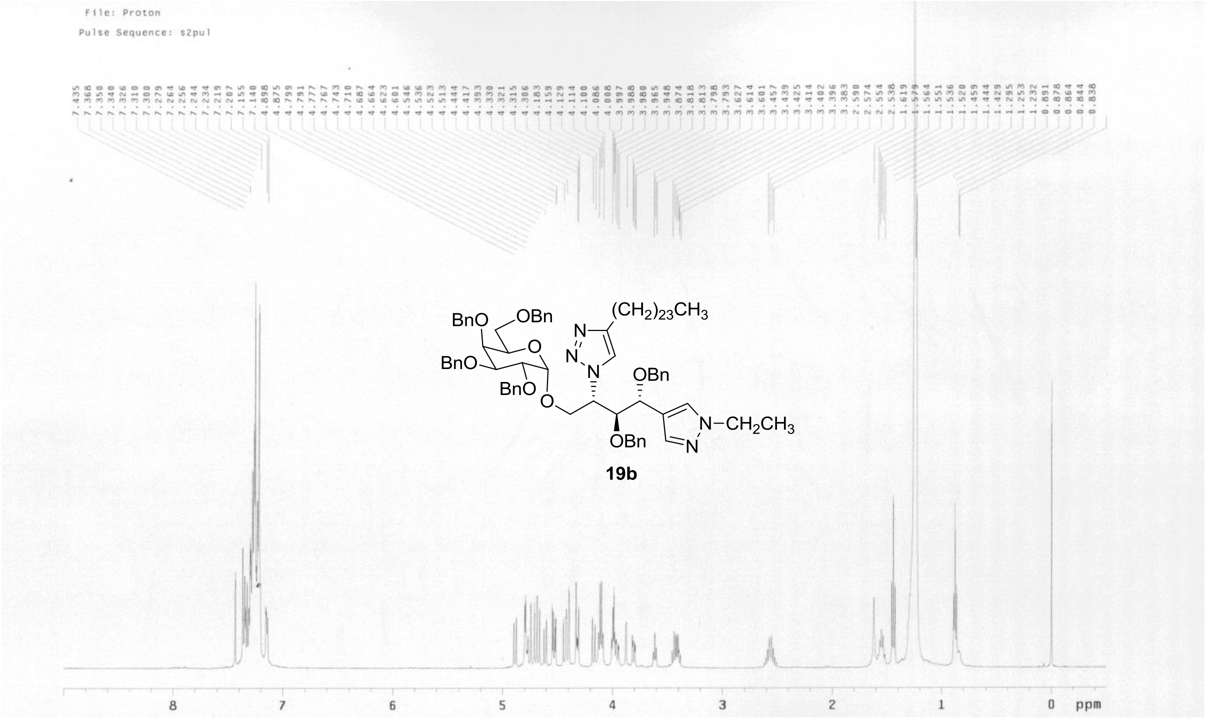
File: Proton
Pulse Sequence: s2pul
7.435 7.368 7.350 7.340 7.326 7.310 7.300 7.279 7.264 7.256 7.244 7.234 7.219 7.207 7.155 7.140 4.898 4.875 4.799 4.791 4.777 4.767 4.743 4.710 4.687 4.664 4.623 4.601 4.546 4.536 4.523 4.513 4.444 4.417 4.393 4.330 4.321 4.315 4.306 4.183 4.159 4.129 4.114 4.100 4.086 4.008 3.997 3.988 3.980 3.965 3.948 3.874 3.818 3.813 3.798 3.793 3.627 3.614 3.601 3.457 3.439 3.425 3.414 3.402 3.396 3.383 2.590 2.574 2.554 2.538 1.619 1.579 1.564 1.551 1.536 1.520 1.459 1.444 1.429 1.295 1.253 1.232 0.891 0.878 0.864 0.844 0.838
BnO
OBn
BnO
BnO
BnO
O
O
N
N
N
$(CH_2)_{23}CH_3$
OBn
OBn
N
N
$N-CH_2CH_3$
19b
8 7 6 5 4 3 2 1 0 ppm

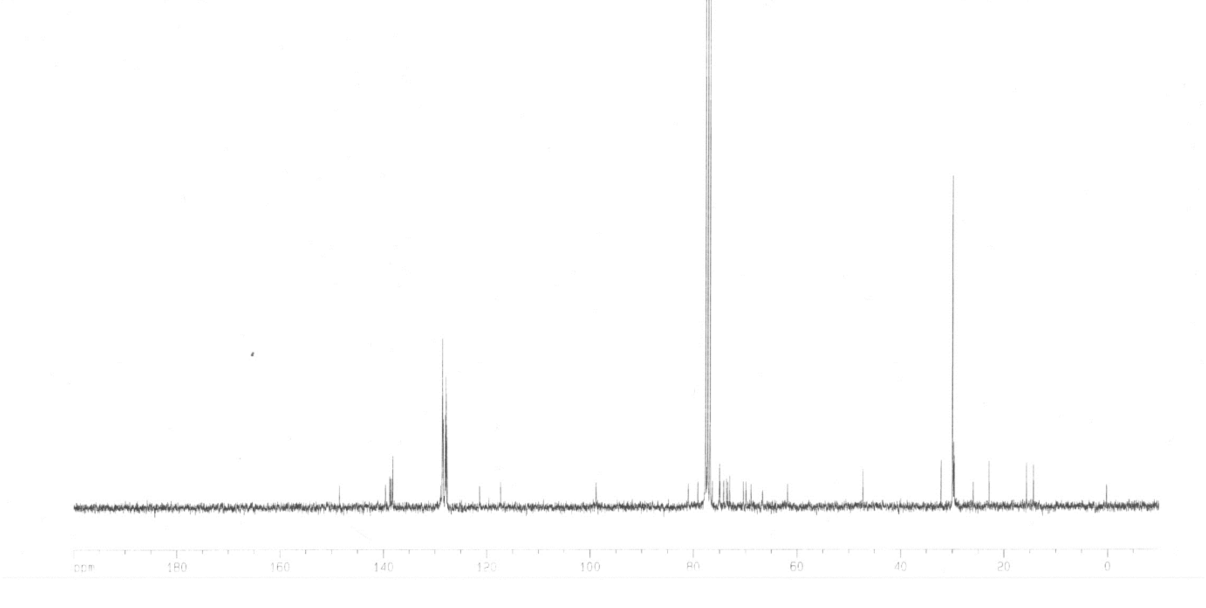
ppm
148.456 139.595 138.752 138.603 138.304 138.154 128.886 128.581 128.528 128.495 128.427 128.386 128.045 127.998 127.884 127.789 127.747 127.614 121.365 117.304 98.830 81.000 79.155 77.655 77.433 77.231 76.809 76.349 74.976 74.874 74.185 73.619 73.406 73.013 70.415 69.882 68.918 66.690 61.843 47.222 32.140 29.927 29.752 29.704 29.580 25.965 22.908 15.663 14.346
ppm 180 160 140 120 100 80 60 40 20 0

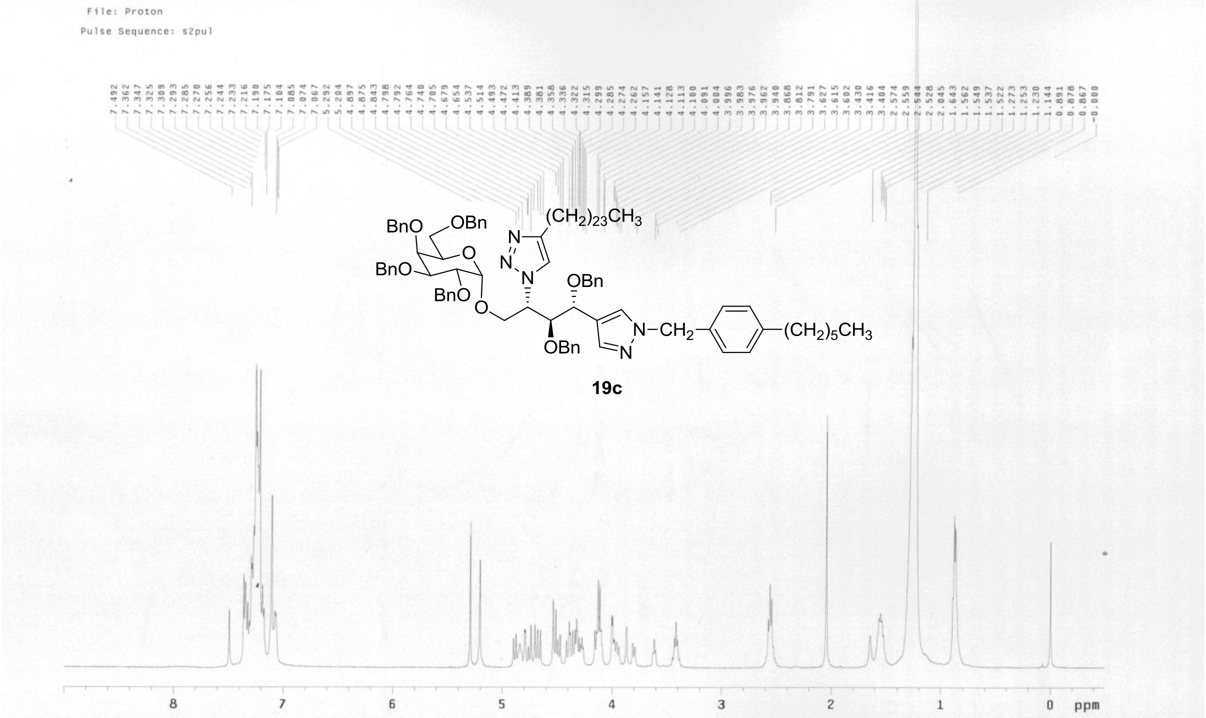
File: Proton
Pulse Sequence: s2pul
BnO
OBn
BnO
BnO
OBn
OBn
N-CH2
(CH2)23CH3
(CH2)5CH3
19c
ppm

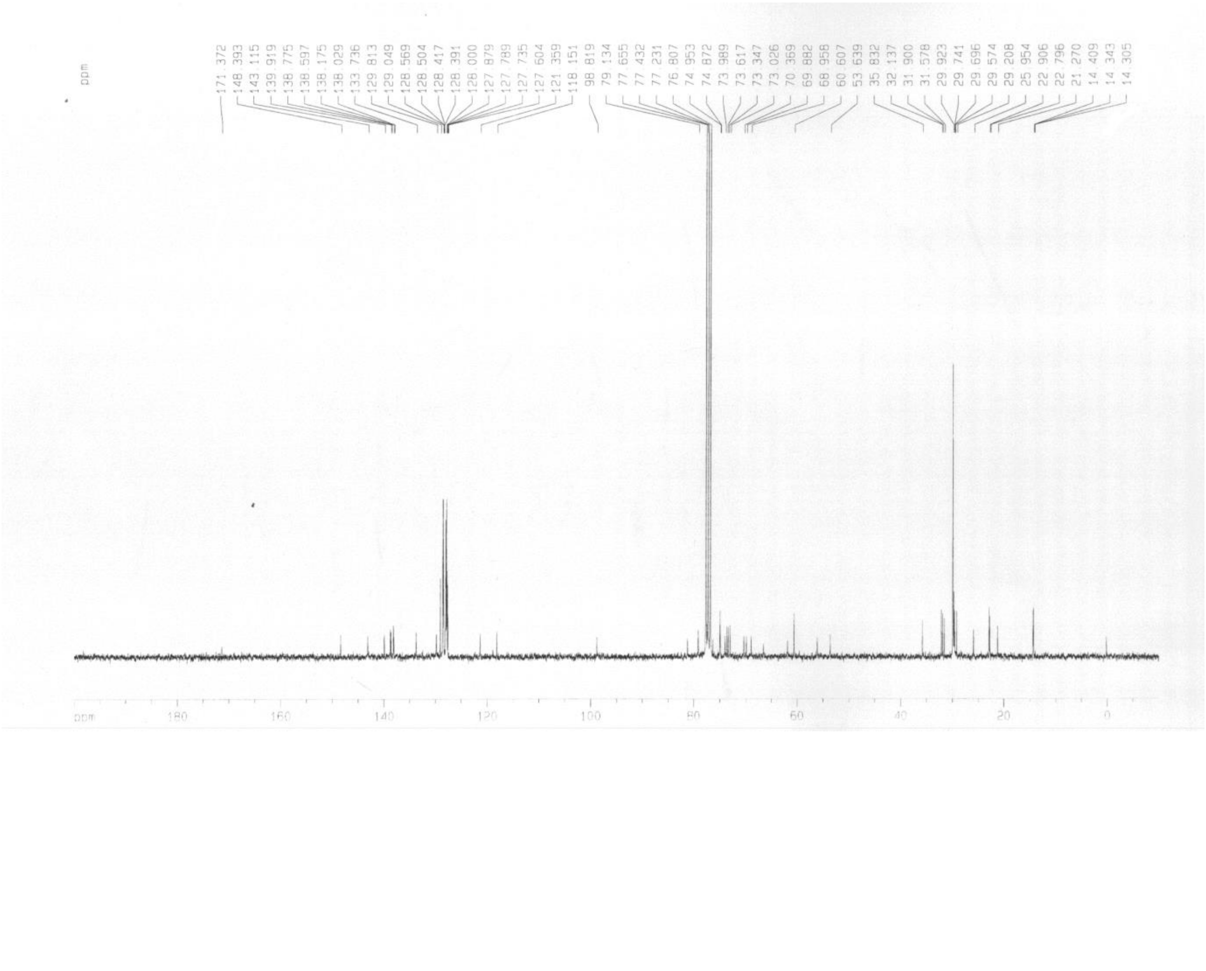
ppm

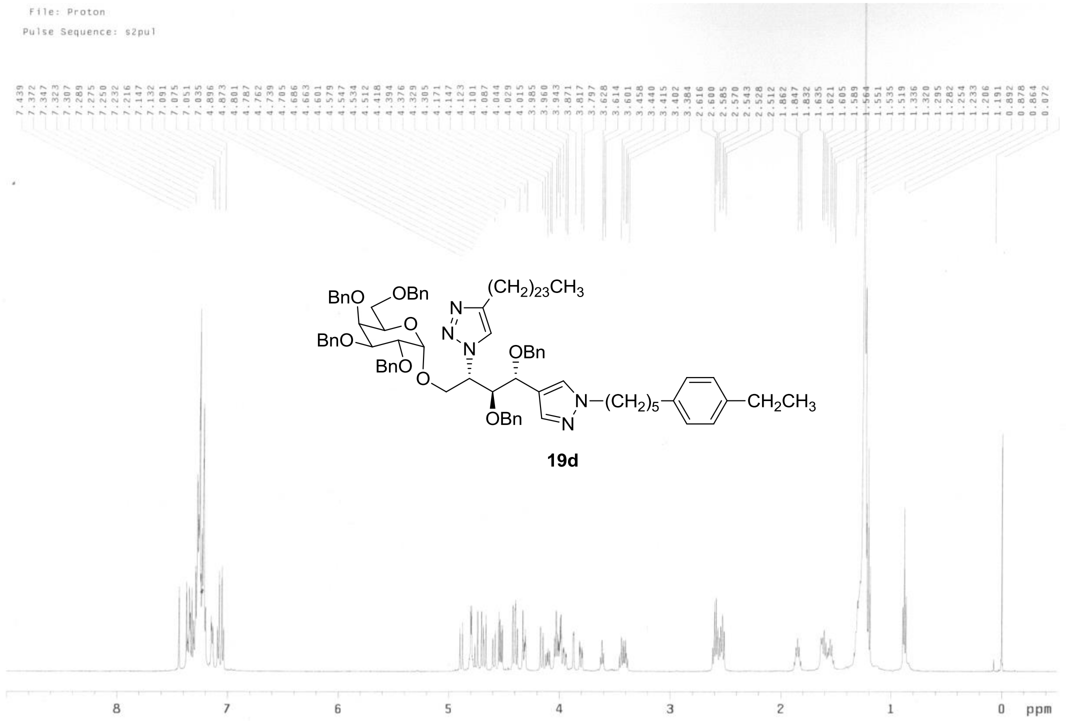
File: Proton
Pulse Sequence: s2pul
BnO
OBn
BnO
BnO
O
(CH2)23CH3
OBn
OBn
N—(CH2)5
CH2CH3
19d
8
7
6
5
4
3
2
1
0
ppm

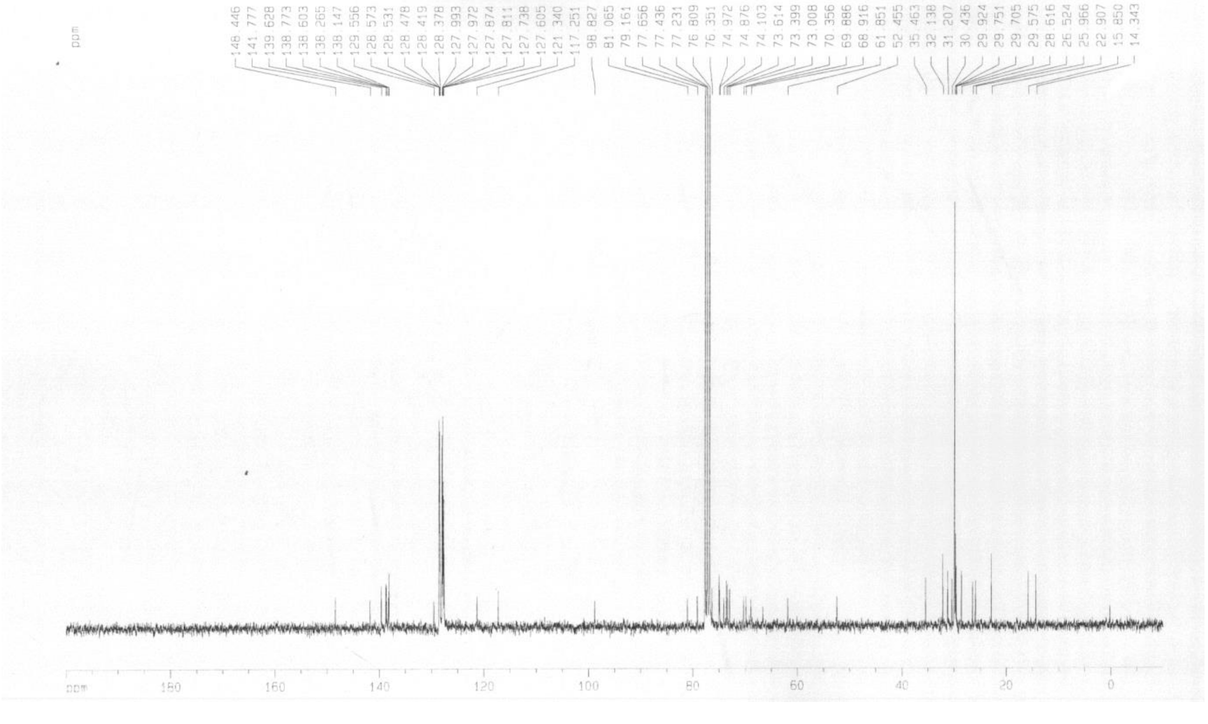
ppm
180
160
140
120
100
80
60
40
20
0

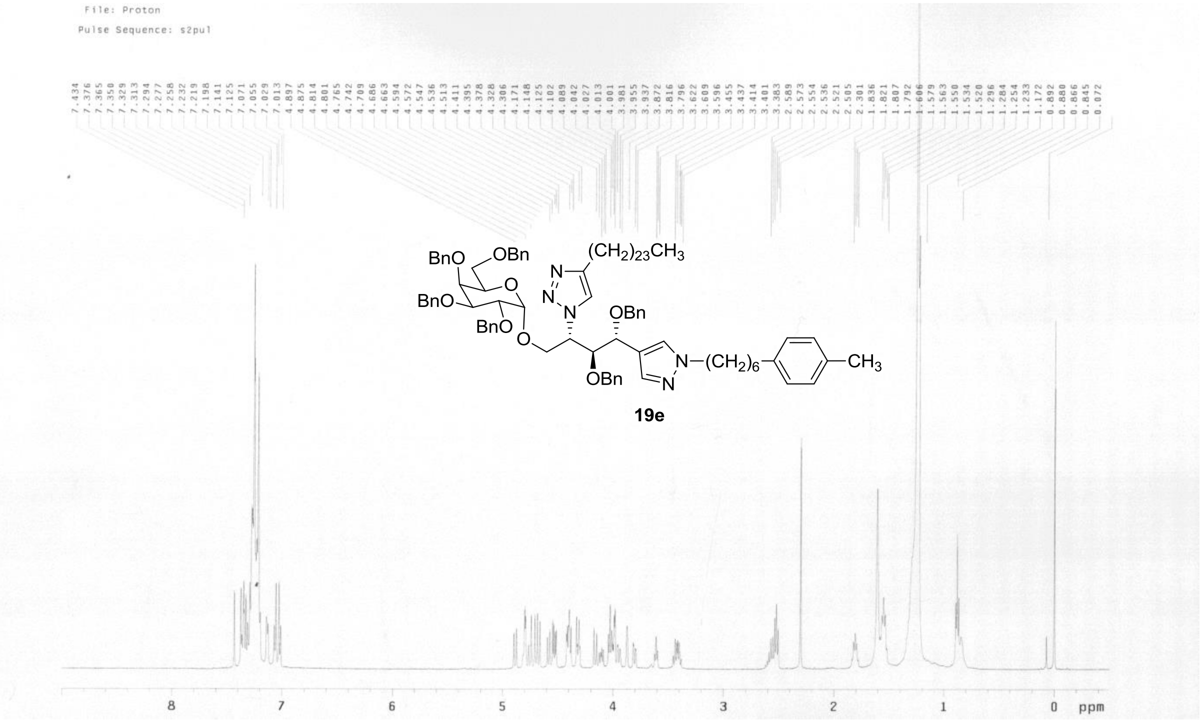
File: Proton
Pulse Sequence: s2pul
19e
ppm

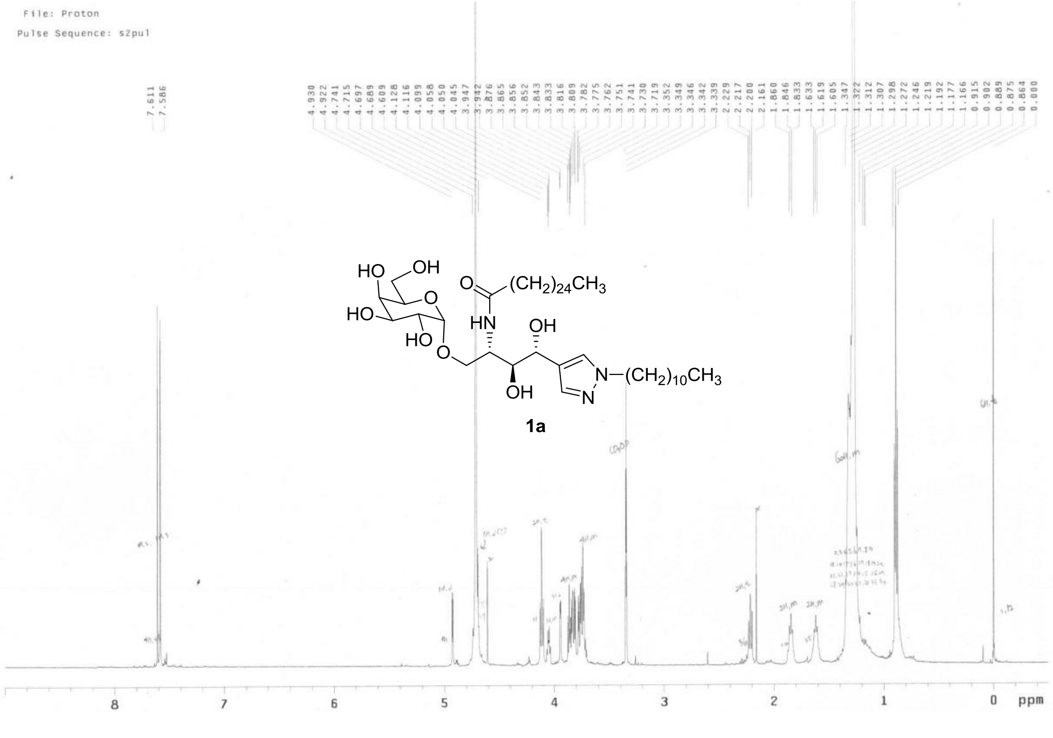
File: Proton
Pulse Sequence: s2pul
1a
ppm

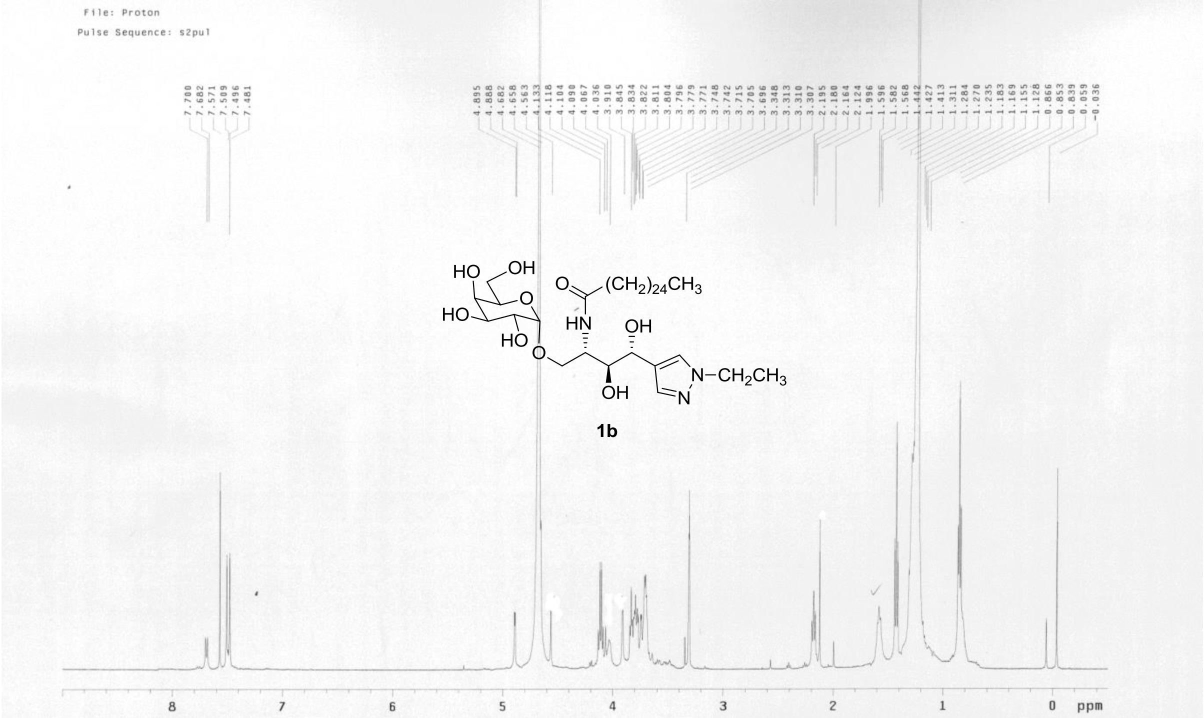
File: Proton
Pulse Sequence: s2pul
7.700 7.682 7.571 7.509 7.496 7.481
4.895 4.888 4.682 4.658 4.563 4.133 4.118 4.104 4.090 4.067 4.036 3.910 3.845 3.834 3.822 3.811 3.804 3.796 3.779 3.771 3.748 3.742 3.715 3.705 3.696 3.348 3.313 3.310 3.307 2.195 2.180 2.164 2.124 1.996 1.596 1.582 1.568 1.442 1.427 1.413 1.311 1.284 1.270 1.235 1.183 1.169 1.155 1.128 0.866 0.853 0.839 0.059 -0.036
HO
OH
HO
HO
O
O
(CH2)24CH3
HN
OH
OH
N-CH2CH3
N
1b
8 7 6 5 4 3 2 1 0 ppm

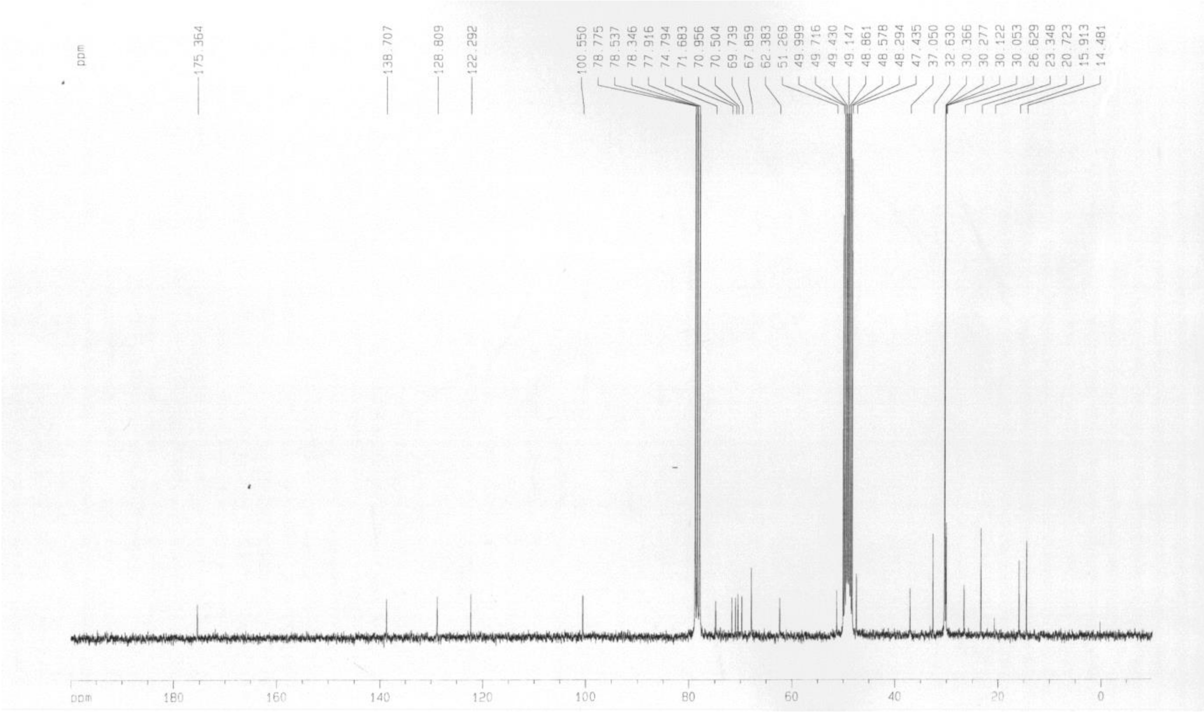
ppm
175.364
138.707
128.809
122.292
100.550 78.775 78.537 78.346 77.916 74.794 71.683 70.956 70.504 69.739 67.859 62.383 51.269 49.999 49.716 49.430 49.147 48.861 48.578 48.294 47.435 37.050 32.630 30.366 30.277 30.122 30.053 26.629 23.348 20.723 15.913 14.481
ppm 180 160 140 120 100 80 60 40 20 0

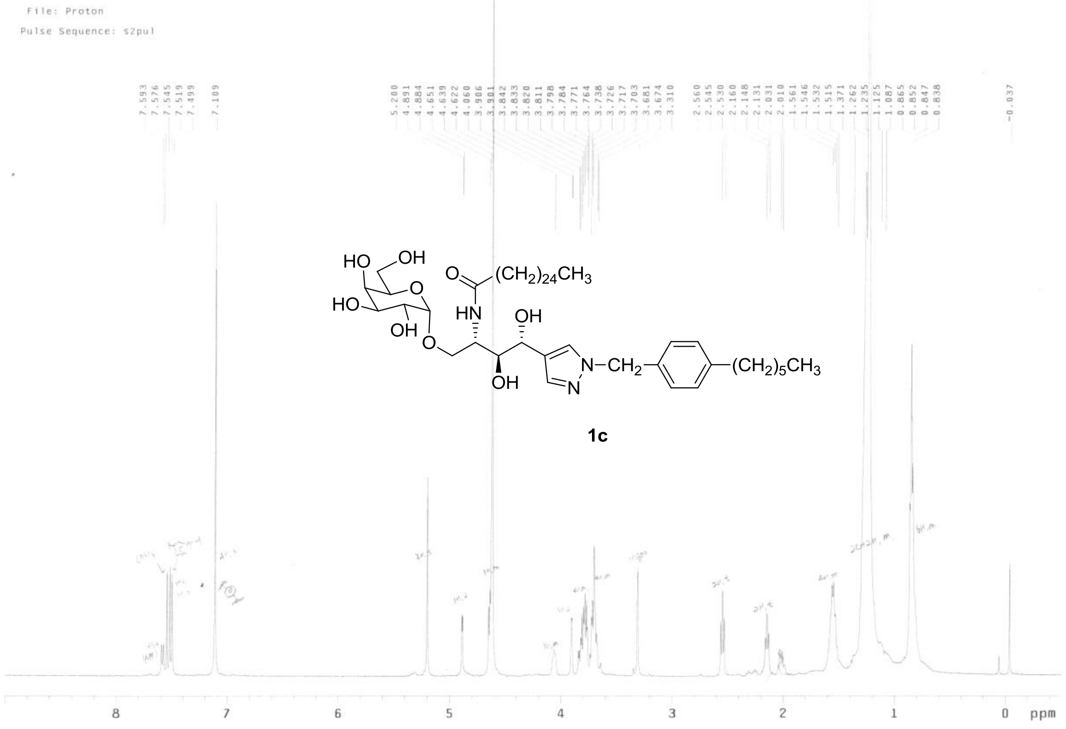
File: Proton
Pulse Sequence: s2pul
7.593 7.576 7.545 7.519 7.499 7.109
5.200 4.891 4.884 4.651 4.639 4.622 4.060 3.906 3.901 3.842 3.833 3.820 3.811 3.798 3.784 3.771 3.764 3.738 3.726 3.717 3.703 3.681 3.674 3.310
2.560 2.545 2.530 2.160 2.148 2.131 2.031 2.010 1.561 1.546 1.532 1.515 1.371 1.262 1.235 1.125 1.087 0.865 0.852 0.847 0.838
-0.037
(CH2)24CH3
(CH2)5CH3
1c
8 7 6 5 4 3 2 1 0 ppm

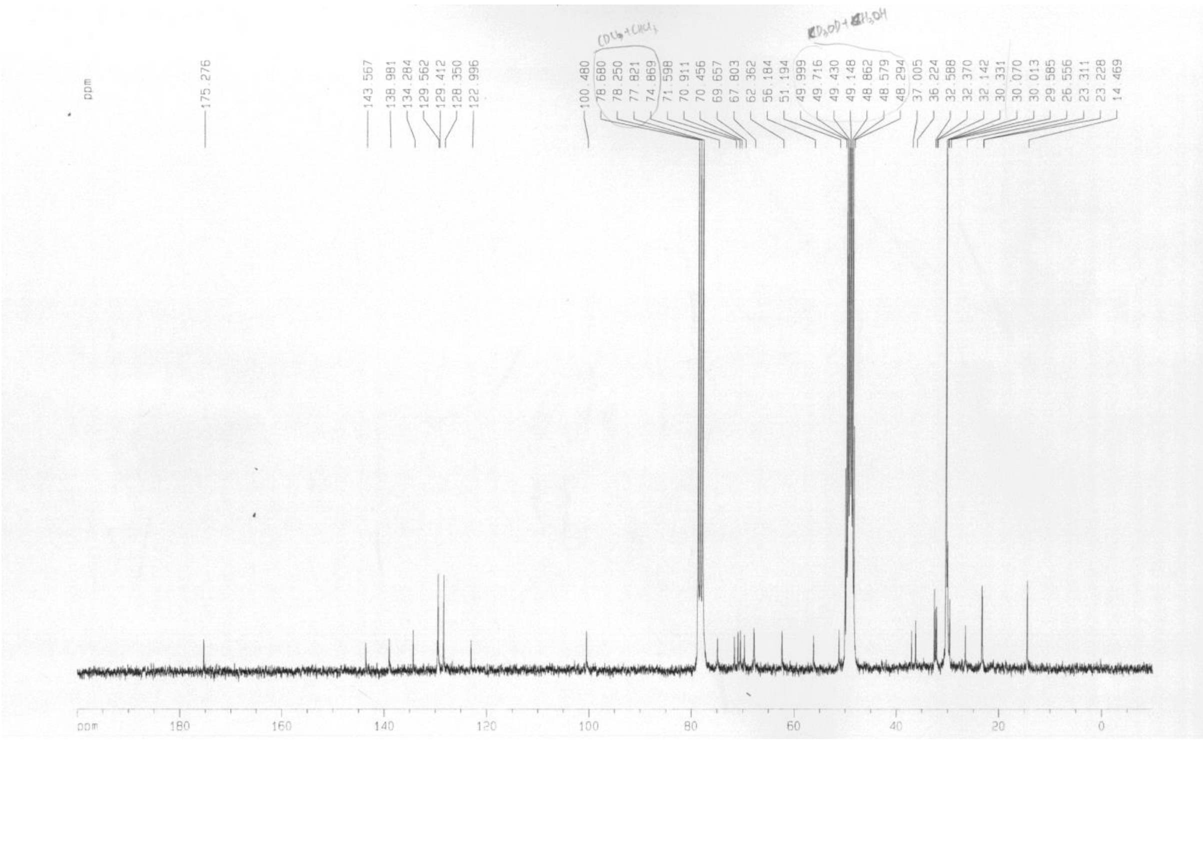
ppm
175.276
143.567 138.981 134.284 129.562 129.412 128.350 122.996
100.480 78.680 78.250 77.821 74.869 71.598 70.911 70.456 69.657 67.803 62.362 56.184 51.194 49.999 49.716 49.430 49.148 48.862 48.579 48.294
37.005 36.224 32.588 32.370 32.142 30.331 30.070 30.013 29.585 26.556 23.311 23.228 14.469
ppm 180 160 140 120 100 80 60 40 20 0

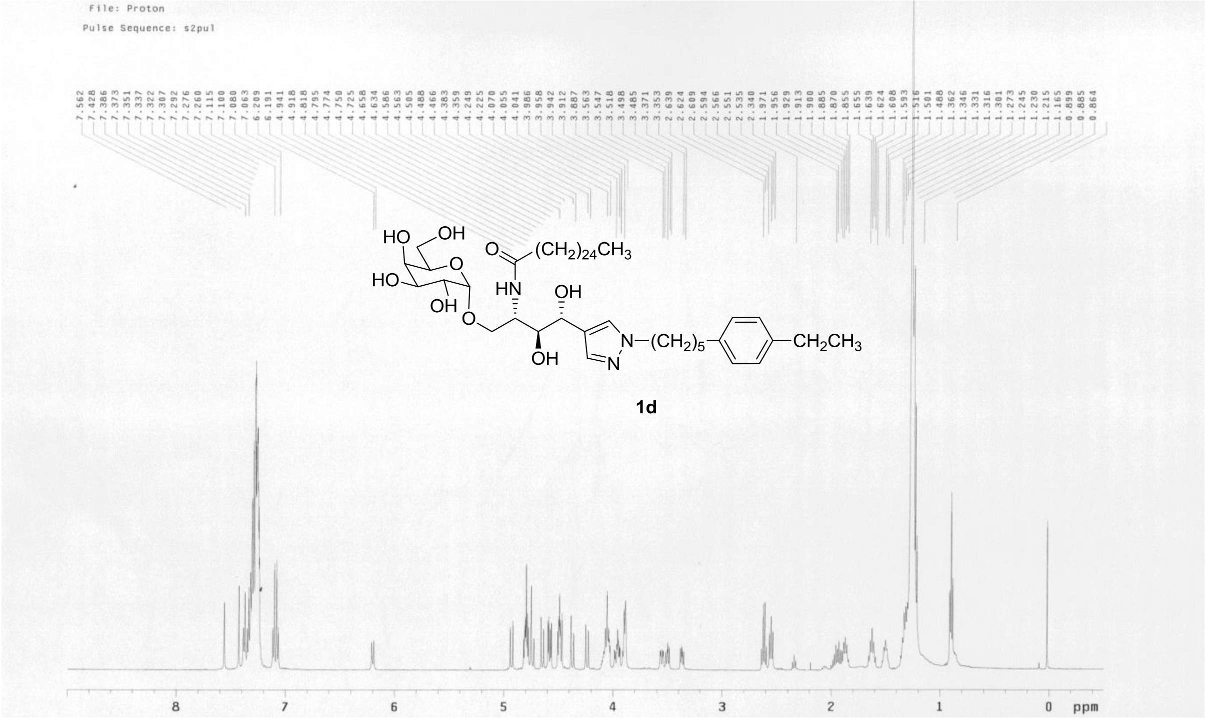
File: Proton
Pulse Sequence: s2pul
HO
OH
O
HO
OH
O
O
(CH2)24CH3
HN
OH
OH
N
N
(CH2)5
CH2CH3
1d
8
7
6
5
4
3
2
1
0
ppm

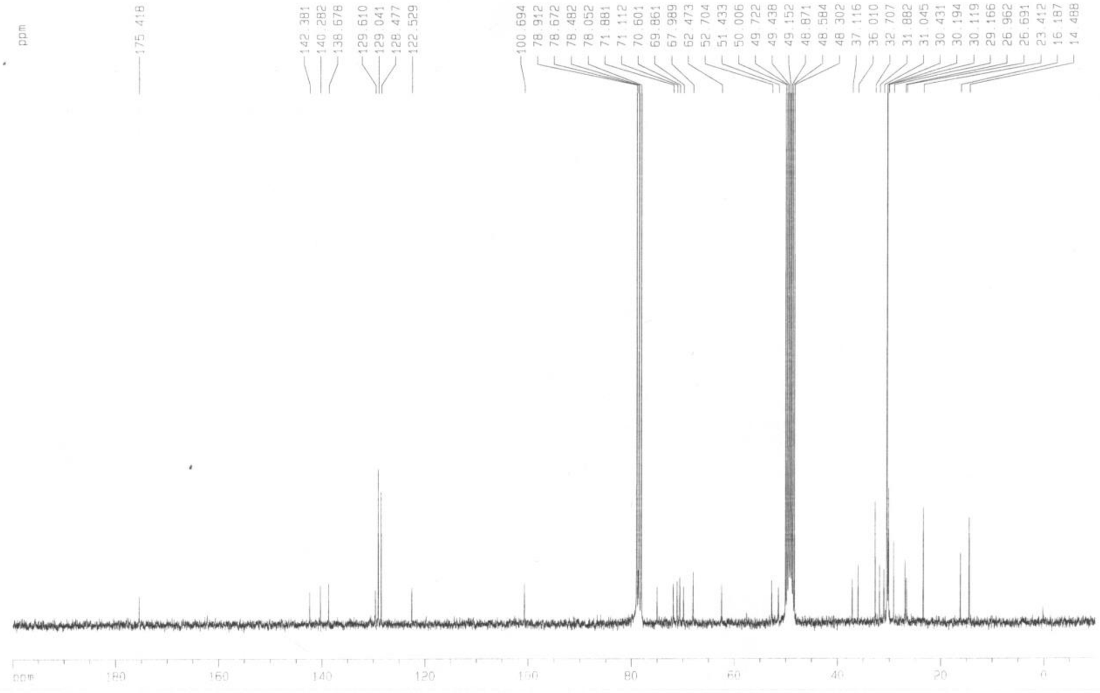
ppm
175.418
142.381
140.282
138.676
129.610
129.041
128.477
122.529
100.694
78.912
78.672
78.482
78.052
71.112
71.881
70.601
69.861
67.989
62.473
52.704
51.433
50.006
49.722
49.438
49.152
48.871
48.584
48.302
37.116
36.010
32.707
31.882
31.045
30.431
30.194
30.119
29.166
26.962
26.691
23.412
16.187
14.488
ppm
180
160
140
120
100
80
60
40
20
0

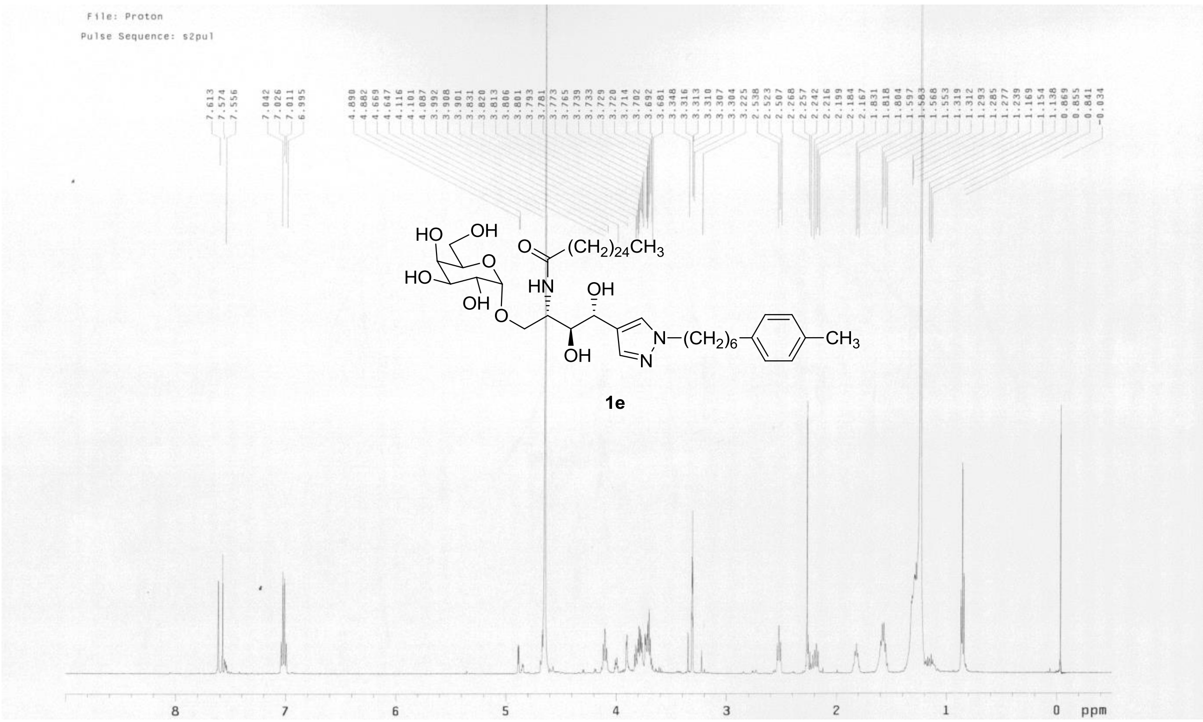
File: Proton
Pulse Sequence: s2pul
1e
8
7
6
5
4
3
2
1
0
ppm

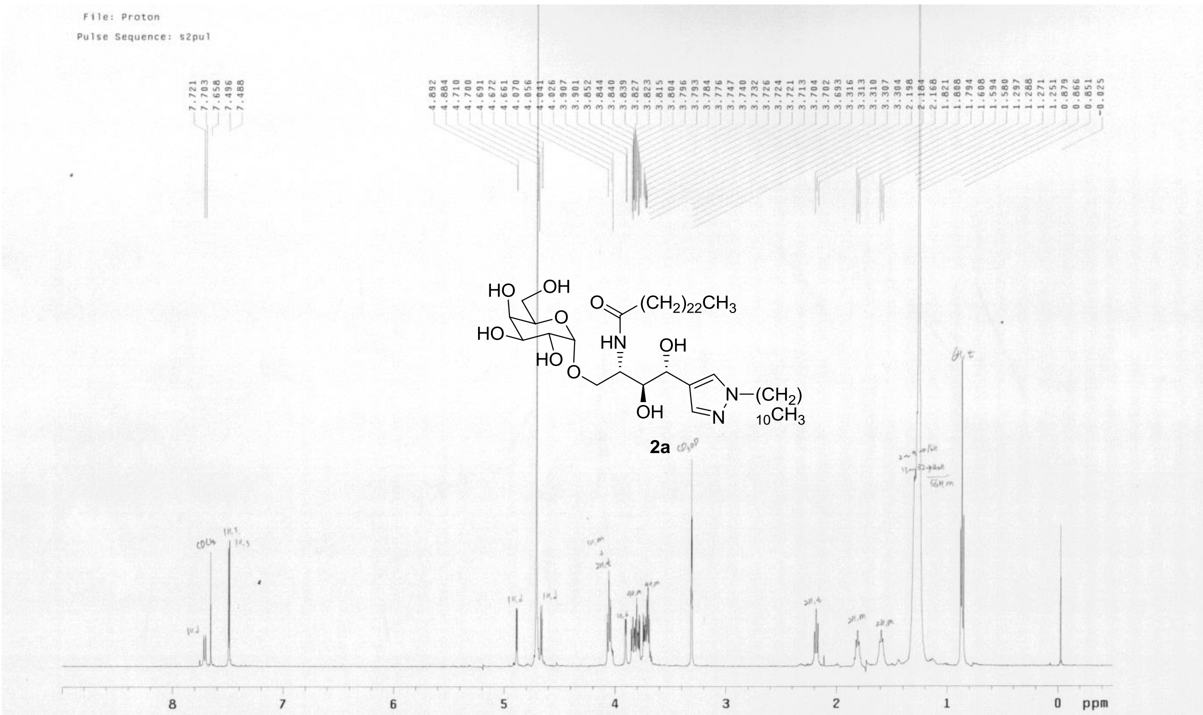
File: Proton
Pulse Sequence: s2pul
2a
8
7
6
5
4
3
2
1
0
ppm

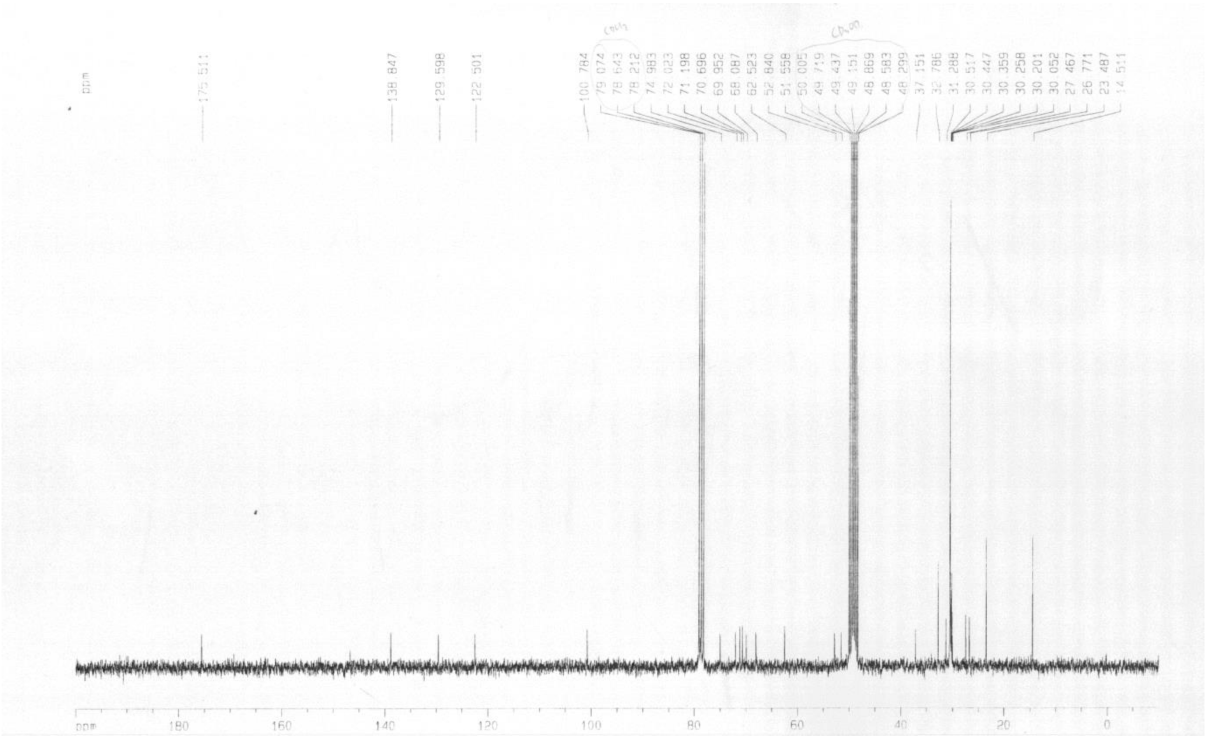
ppm
175.511
138.847
129.598
122.501
100.784
79.074
78.643
78.212
74.983
72.023
71.198
70.696
69.952
68.087
62.523
52.840
51.558
50.005
49.719
49.437
49.151
48.869
48.583
48.299
37.151
32.786
31.288
30.517
30.447
30.359
30.258
30.201
30.052
27.467
26.771
23.487
14.511
ppm 180 160 140 120 100 80 60 40 20 0

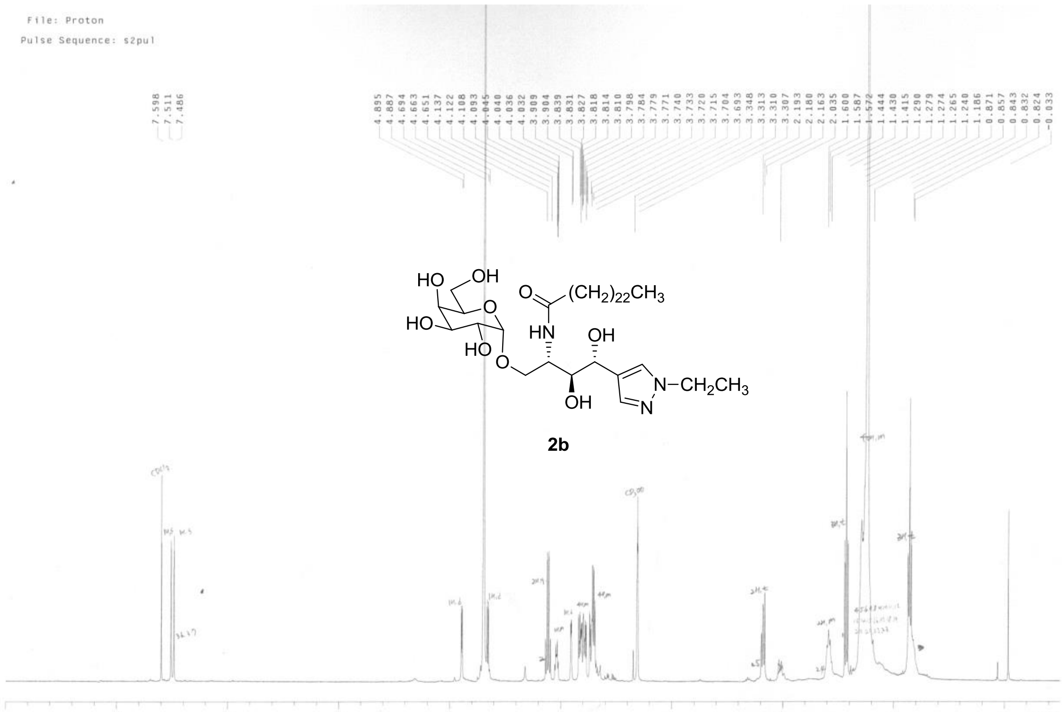
File: Proton
Pulse Sequence: s2pul
7.598
7.511
7.486
4.895
4.887
4.694
4.663
4.651
4.137
4.122
4.108
4.093
4.045
4.040
4.036
4.032
3.909
3.904
3.839
3.831
3.827
3.818
3.814
3.810
3.798
3.784
3.779
3.771
3.740
3.733
3.720
3.715
3.704
3.693
3.348
3.313
3.310
3.307
2.193
2.180
2.163
2.035
1.600
1.587
1.572
1.444
1.430
1.415
1.290
1.279
1.274
1.265
1.240
1.186
0.871
0.857
0.843
0.832
0.824
-0.033
HO
OH
O
HO
HO
O
O
(CH2)22CH3
HN
OH
OH
N-CH2CH3
N
8 7 6 5 4 3 2 1 0 ppm

2b

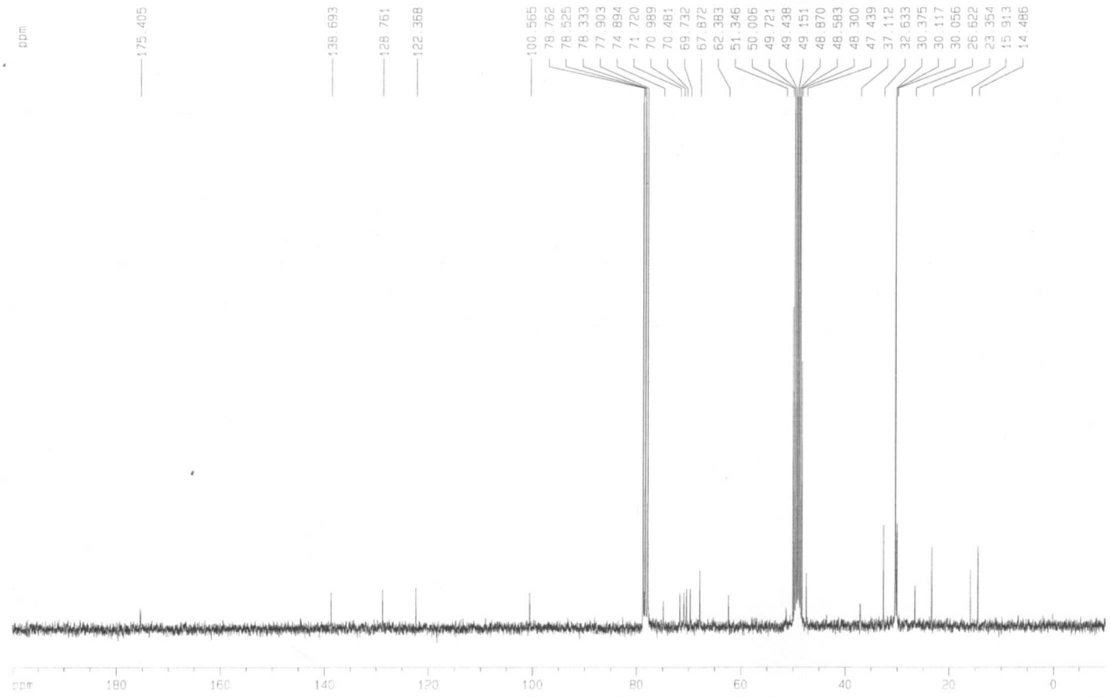
ppm
175.405
138.693
128.761
122.368
100.565
78.762
78.525
78.333
77.903
74.894
71.720
70.989
70.481
69.732
67.872
62.383
51.346
50.006
49.721
49.438
49.151
48.870
48.583
48.300
47.439
37.112
32.633
30.375
30.117
30.056
26.622
23.354
15.913
14.486
ppm 180 160 140 120 100 80 60 40 20 0

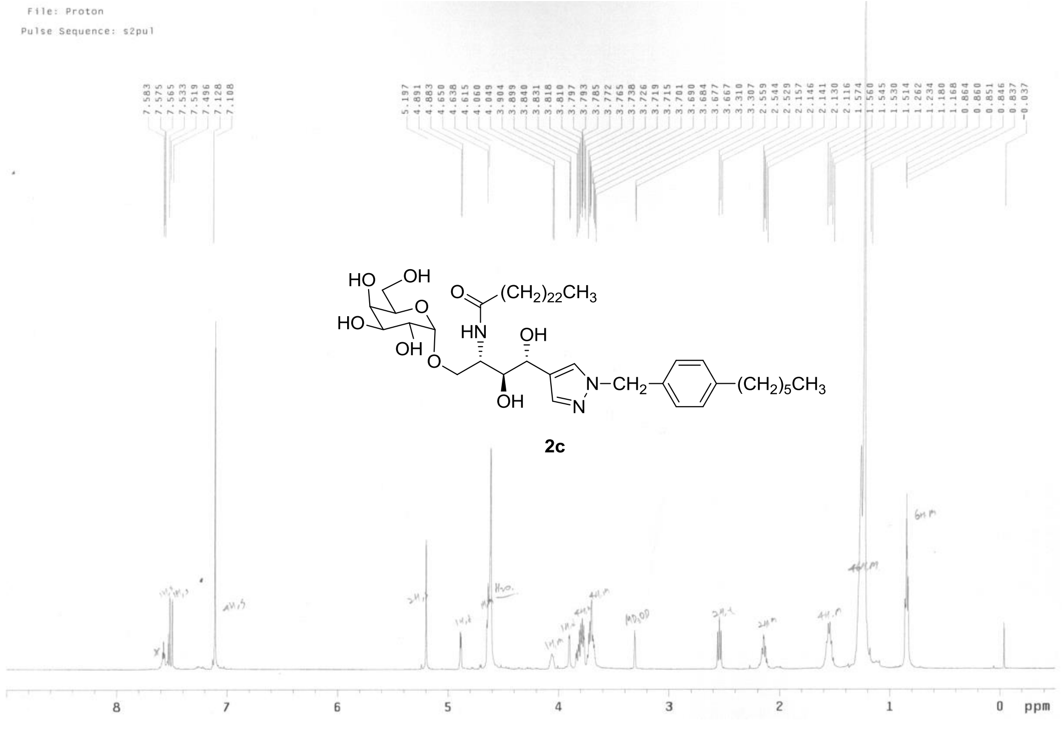
File: Proton
Pulse Sequence: s2pul
7.583
7.575
7.565
7.533
7.519
7.496
7.128
7.108
5.197
4.891
4.883
4.650
4.638
4.615
4.060
4.049
3.904
3.899
3.840
3.831
3.818
3.810
3.797
3.793
3.785
3.772
3.765
3.738
3.726
3.719
3.715
3.701
3.690
3.684
3.677
3.667
3.310
3.307
2.559
2.544
2.529
2.157
2.146
2.141
2.130
2.116
1.560
1.574
1.545
1.530
1.514
1.262
1.234
1.180
1.168
0.864
0.860
0.851
0.846
0.837
-0.037
HO
OH
HO
OH
O
O
O
(CH2)22CH3
HN
OH
OH
N
N
CH2
(CH2)5CH3
2c
8 7 6 5 4 3 2 1 0 ppm

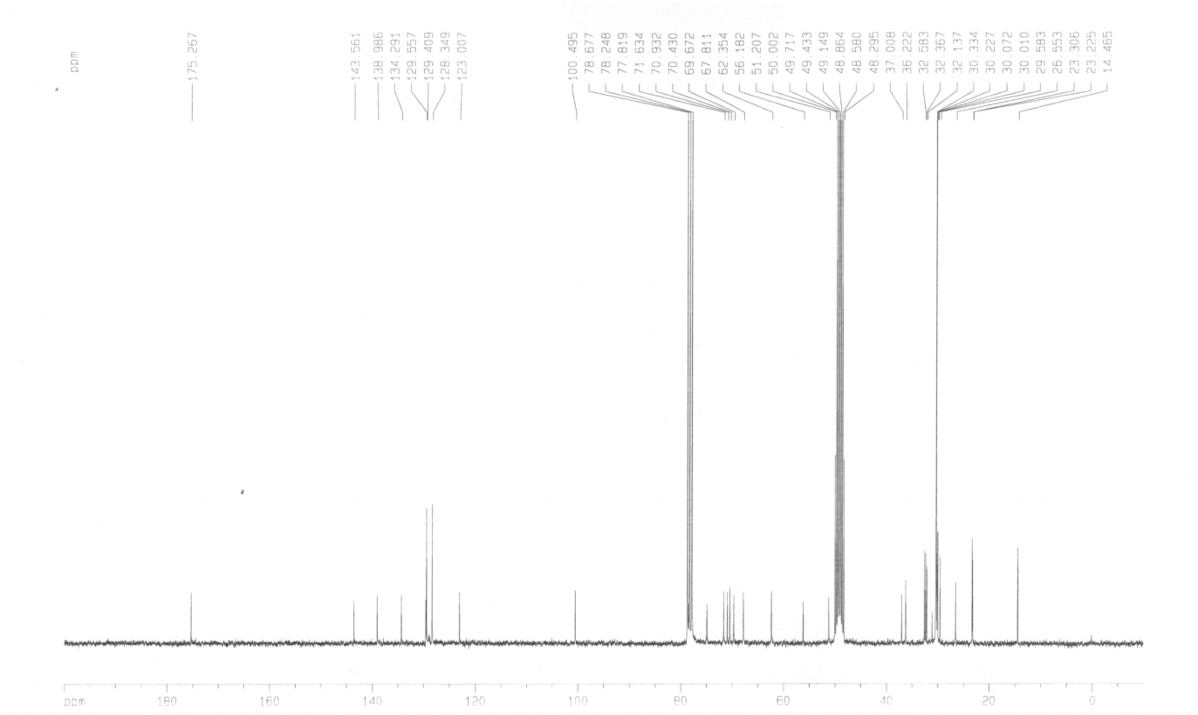
ppm
175.267
143.561
138.986
134.291
129.557
129.409
128.349
123.007
100.495
78.677
78.248
77.819
71.634
70.932
70.430
69.672
67.811
62.354
56.182
51.207
50.002
49.717
49.433
49.149
48.864
48.580
48.295
37.008
36.222
32.583
32.367
32.137
30.334
30.227
30.072
30.010
29.583
26.553
23.306
23.225
14.465
ppm 180 160 140 120 100 80 60 40 20 0

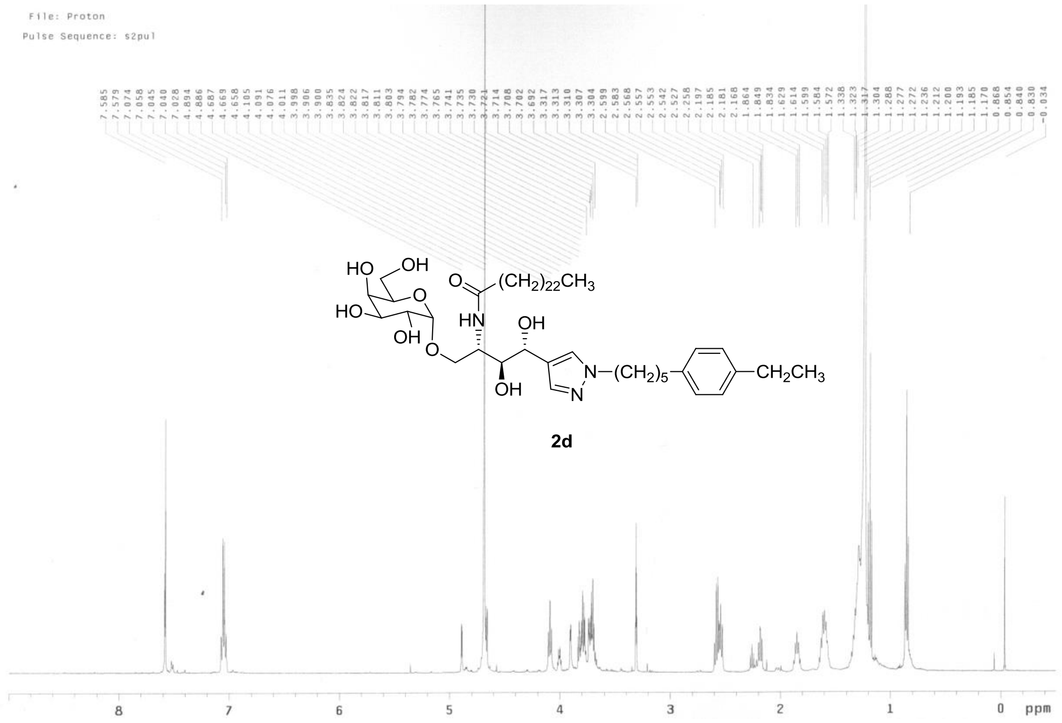
File: Proton
Pulse Sequence: s2pul
7.585 7.579 7.058 7.045 7.040 7.028 4.894 4.886 4.687 4.669 4.658 4.105 4.091 4.076 4.011 3.998 3.986 3.900 3.835 3.824 3.822 3.817 3.811 3.803 3.794 3.782 3.774 3.765 3.741 3.735 3.730 3.721 3.714 3.708 3.702 3.692 3.317 3.313 3.310 3.307 3.304 2.599 2.583 2.568 2.557 2.553 2.542 2.527 2.258 2.197 2.185 2.181 2.168 1.864 1.849 1.834 1.629 1.614 1.599 1.584 1.572 1.338 1.323 1.317 1.304 1.288 1.277 1.272 1.236 1.212 1.200 1.193 1.185 1.170 0.868 0.854 0.840 0.830 -0.034
HO
OH
HO
O
OH
O
O
(CH2)22CH3
HN
OH
OH
N
N
(CH2)5
CH2CH3
2d
8 7 6 5 4 3 2 1 0 ppm

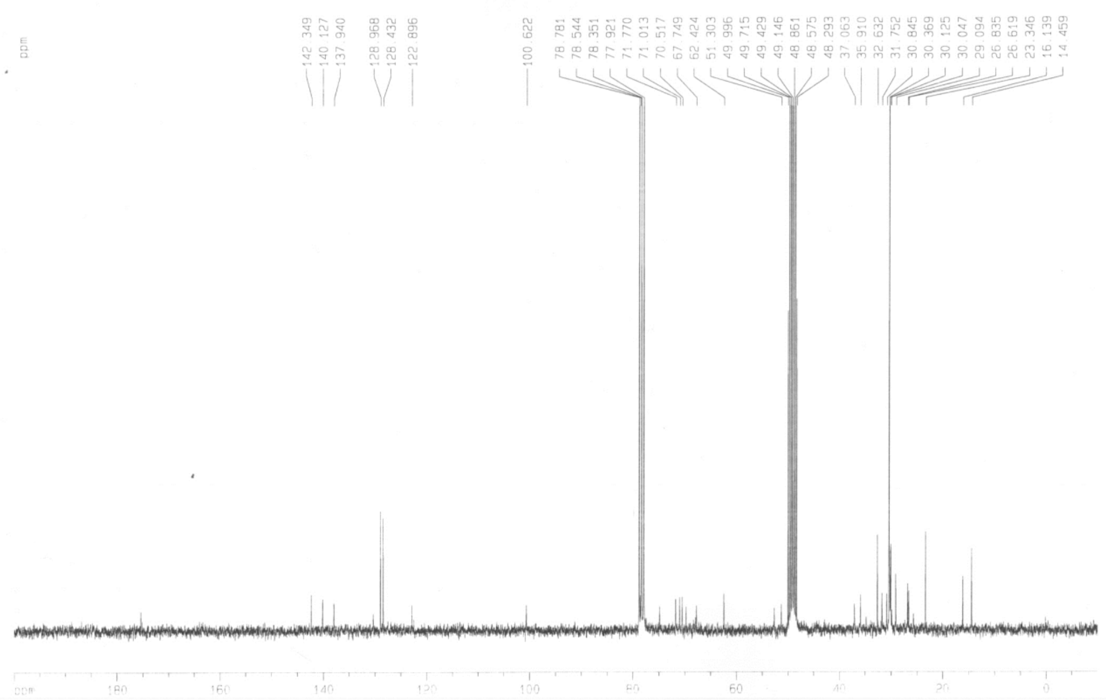

ppm
142.349
140.127
137.940
128.968
128.432
122.896
100.622
78.781
78.544
78.351
77.921
71.770
71.013
70.517
67.749
62.424
51.303
49.996
49.715
49.429
49.146
48.861
48.575
48.293
37.063
35.910
32.632
31.752
30.845
30.369
30.125
30.047
29.094
26.835
26.619
23.346
16.139
14.459
ppm 180 160 140 120 100 80 60 40 20 0

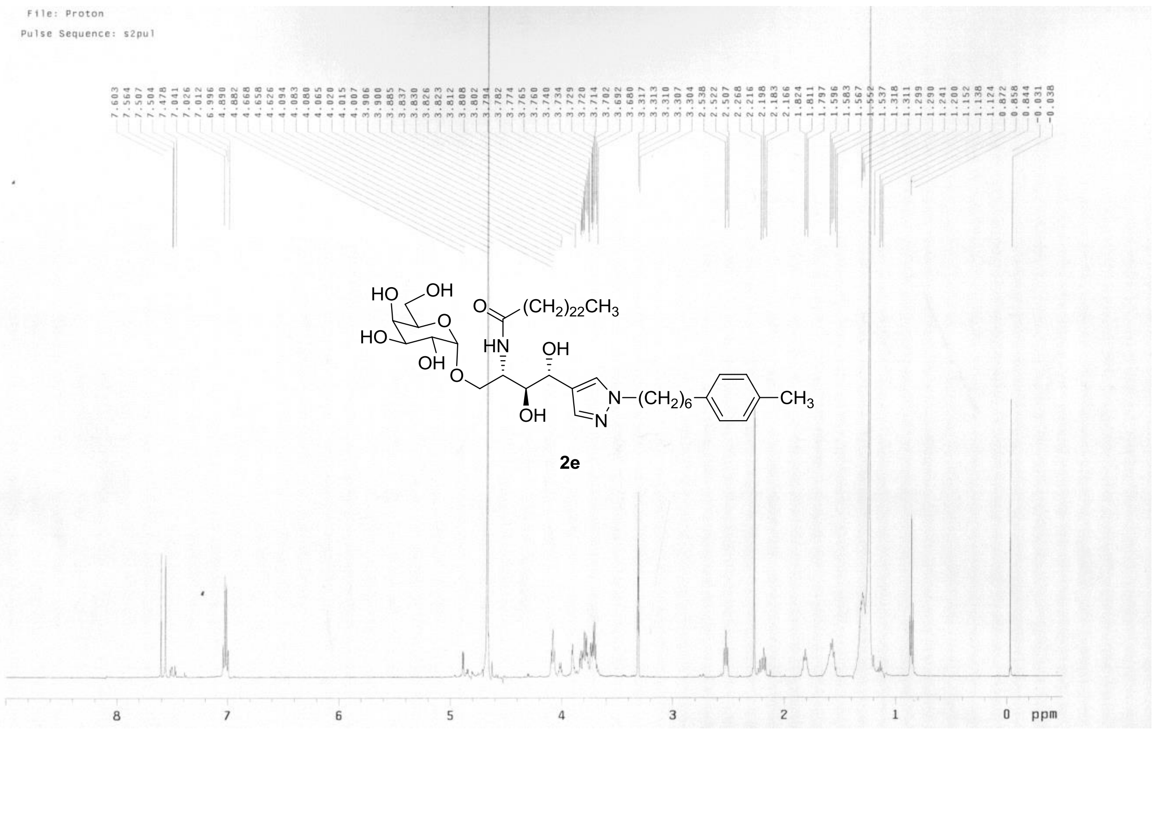

File: Proton
Pulse Sequence: s2pul
HO
OH
O
HO
OH
(CH2)22CH3
HN
OH
N
(CH2)6
CH3
2e
8 7 6 5 4 3 2 1 0 ppm

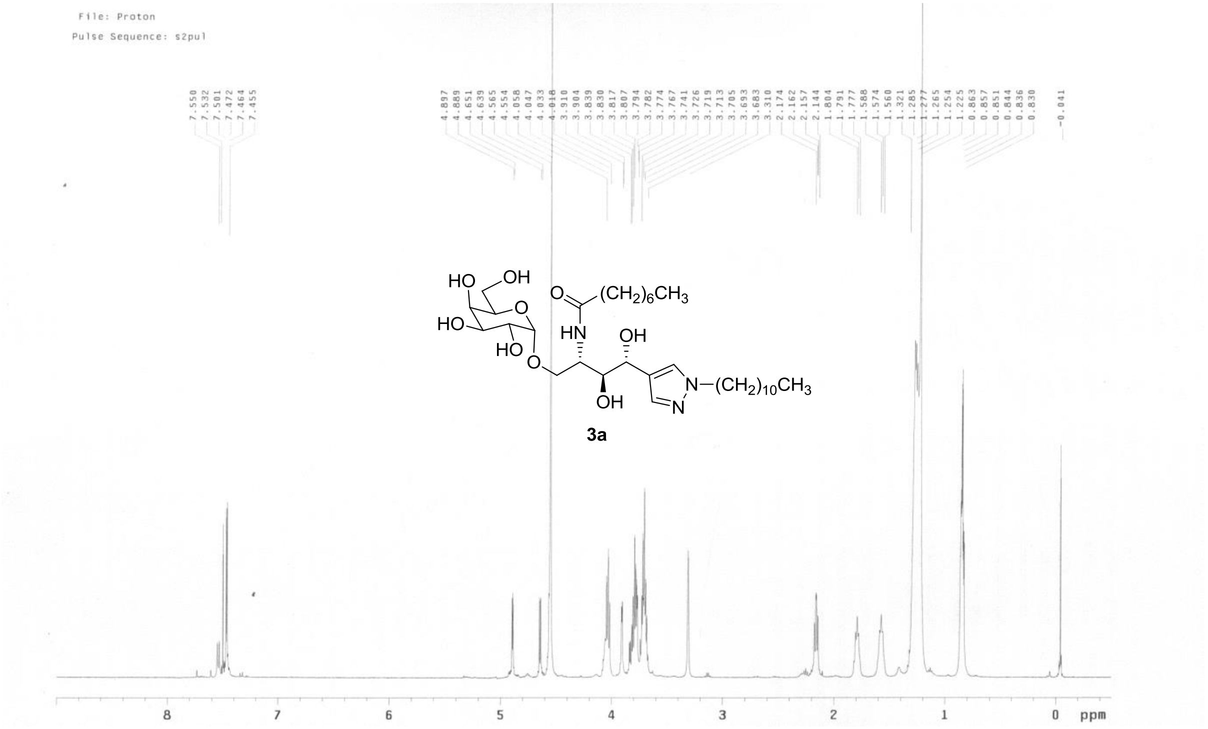
File: Proton
Pulse Sequence: s2pul
7.550 7.532 7.501 7.472 7.464 7.455
4.897 4.889 4.651 4.639 4.565 4.554 4.058 4.047 4.033 4.018 3.910 3.904 3.839 3.830 3.817 3.807 3.794 3.782 3.774 3.767 3.741 3.726 3.719 3.713 3.705 3.693 3.683 3.310 2.174 2.162 2.157 2.144 1.804 1.791 1.777 1.588 1.574 1.560 1.321 1.285 1.277 1.265 1.254 1.225 0.863 0.857 0.851 0.844 0.836 0.830
-0.041
HO
OH
HO
HO
O
O
(CH2)6CH3
HN
OH
OH
N
N
(CH2)10CH3
3a
8 7 6 5 4 3 2 1 0 ppm

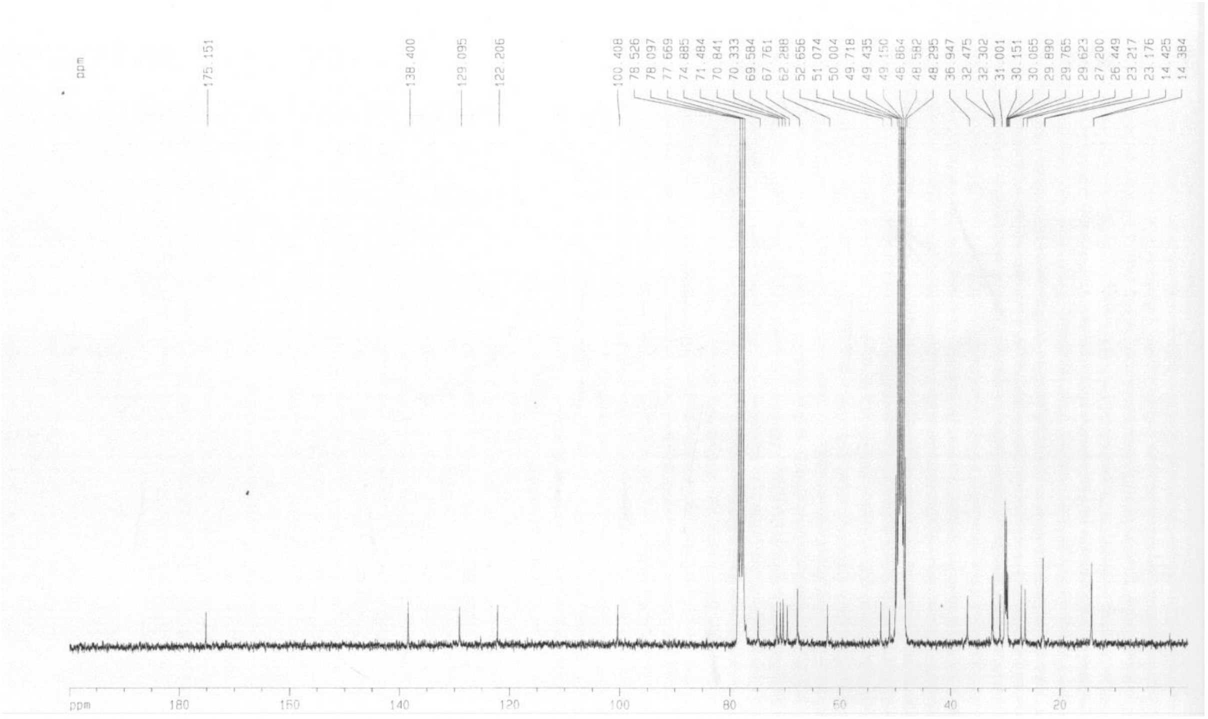
ppm
175.151
138.400
129.095
122.206
100.408 78.526 78.097 77.669 74.885 71.484 70.841 70.333 69.584 67.761 62.288 52.656 51.074 50.004 49.718 49.435 49.150 48.864 48.582 48.295 36.947 32.475 32.302 31.001 30.151 30.065 29.890 29.765 29.623 27.200 26.449 23.217 23.176 14.425 14.384
ppm 180 160 140 120 100 80 60 40 20

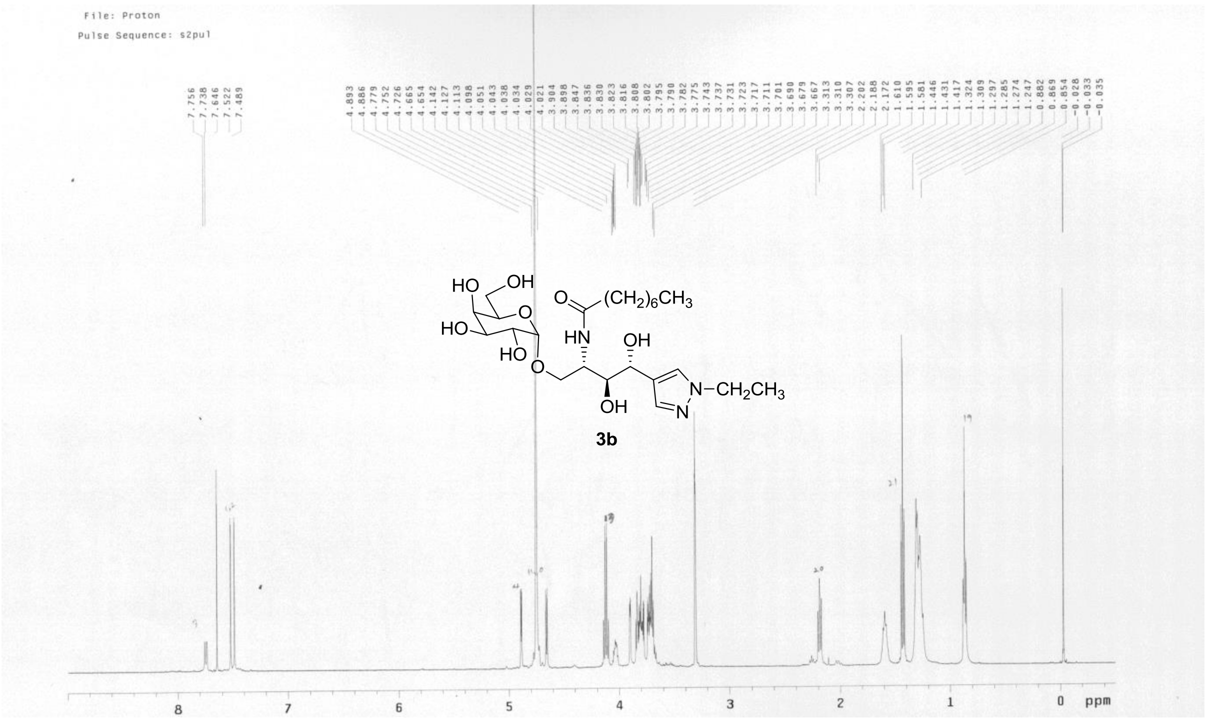
File: Proton
Pulse Sequence: s2pul
3b
ppm

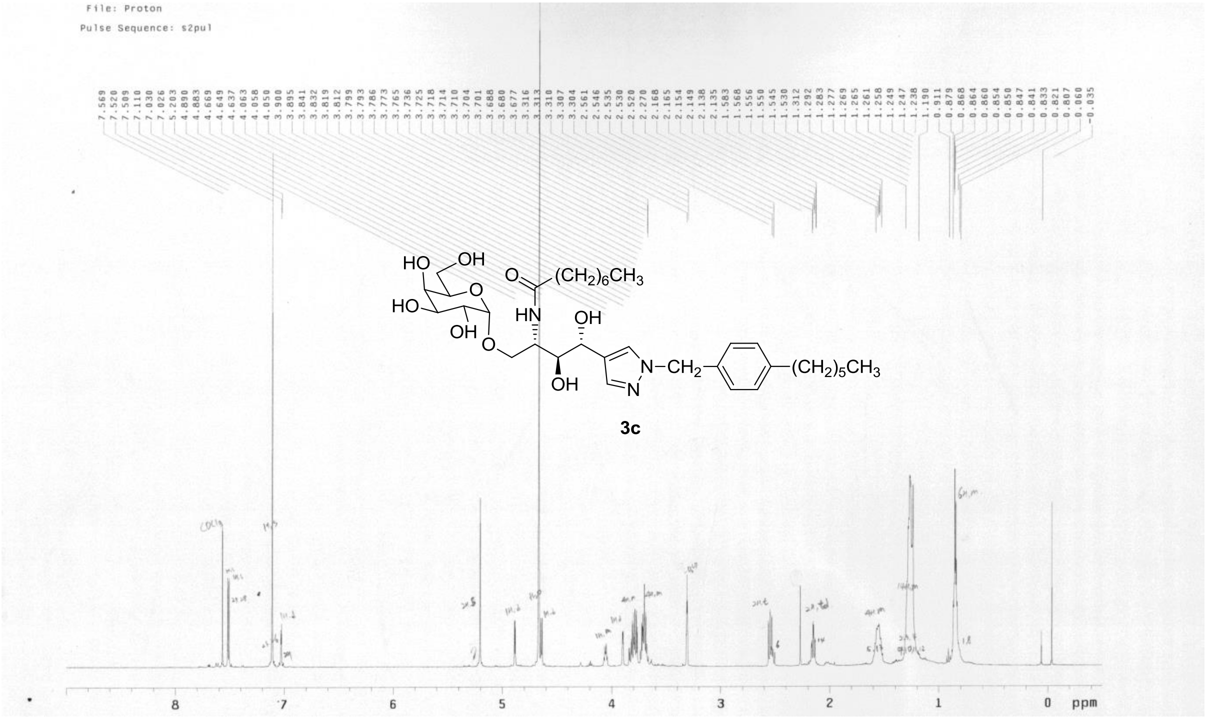
File: Proton
Pulse Sequence: s2pul
3c
ppm

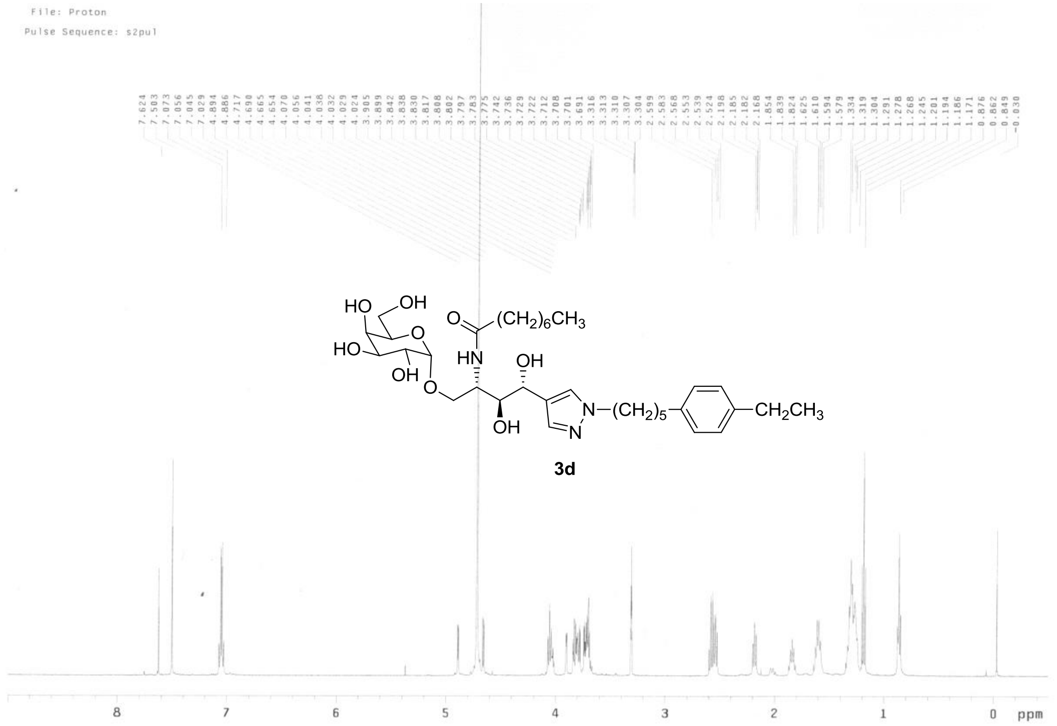
File: Proton
Pulse Sequence: s2pul
ppm

3d

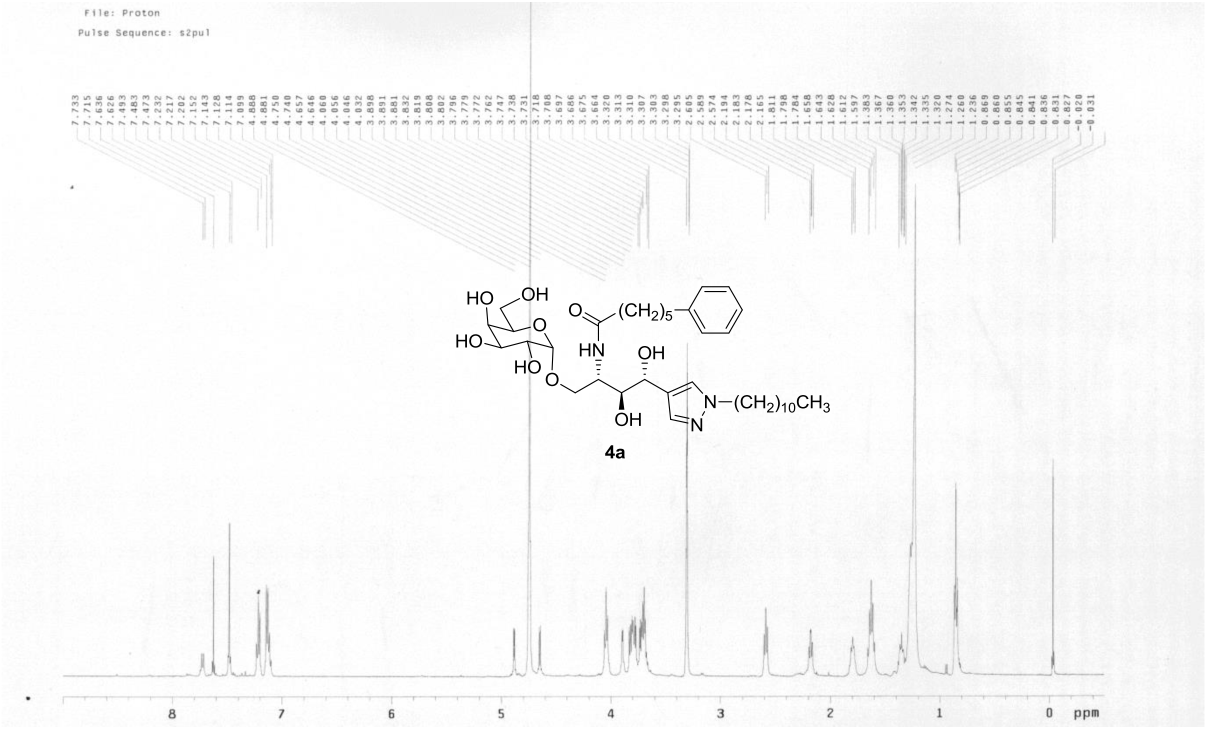
File: Proton
Pulse Sequence: s2pul
ppm

4a

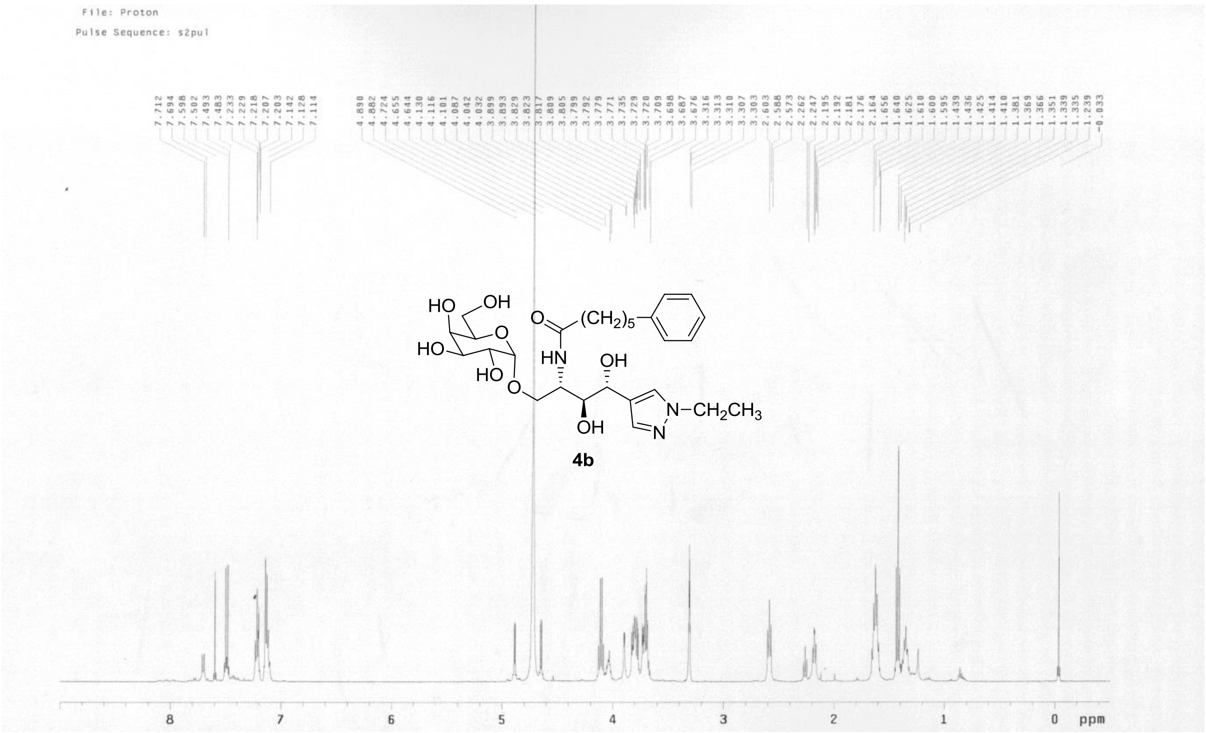
File: Proton
Pulse Sequence: s2pul
HO
OH
HO
HO
O
O
O
(CH2)5
HN
OH
OH
N-CH2CH3
N
8
7
6
5
4
3
2
1
0
ppm

4b

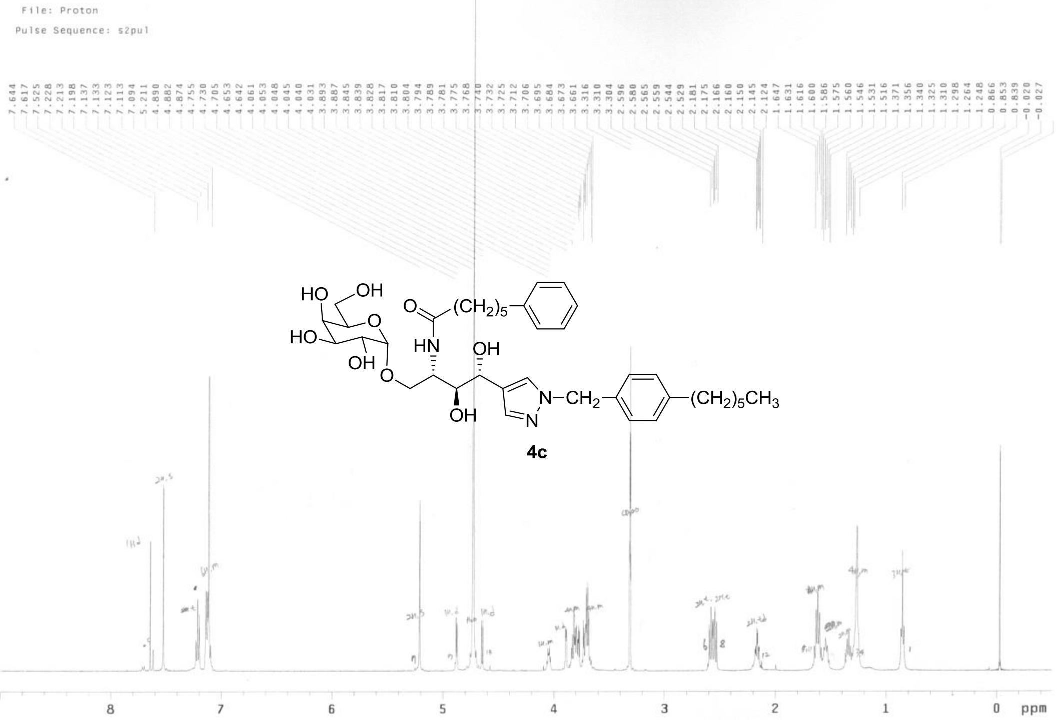
File: Proton
Pulse Sequence: s2pul
HO
OH
HO
OH
O
O
O
(CH2)5
HN
OH
OH
N-CH2
(CH2)5CH3
N
8
7
6
5
4
3
2
1
0
ppm

4c

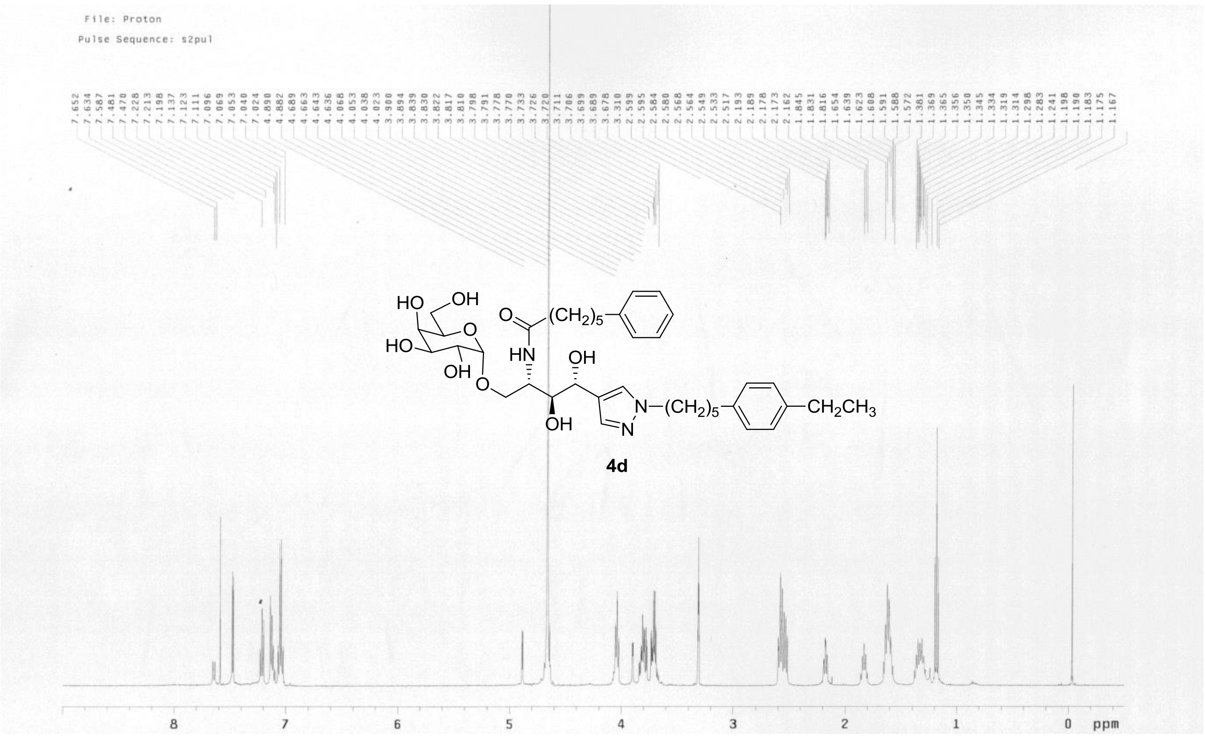
File: Proton
Pulse Sequence: s2pul
HO
OH
O
HO
OH
O
O
(CH2)5
HN
OH
OH
N
N
(CH2)5
CH2CH3
4d
8
7
6
5
4
3
2
1
0
ppm

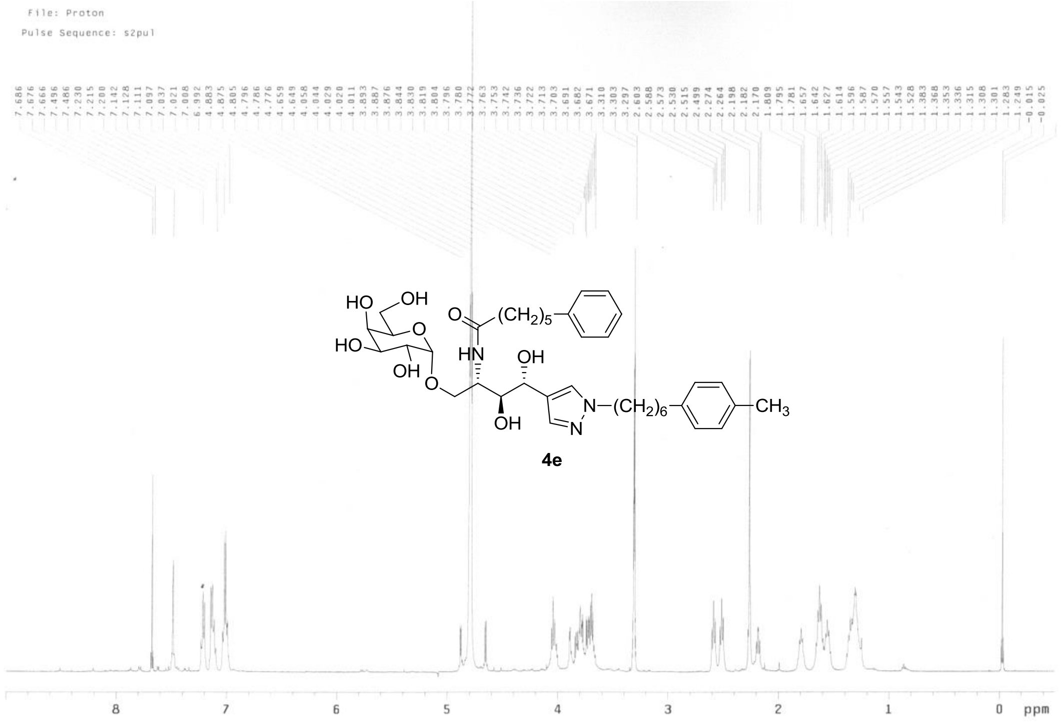
File: Proton
Pulse Sequence: s2pul
HO
OH
O
HO
OH
O
O
(CH2)5
HN
OH
OH
N
N
(CH2)6
CH3
4e
8
7
6
5
4
3
2
1
0
ppm

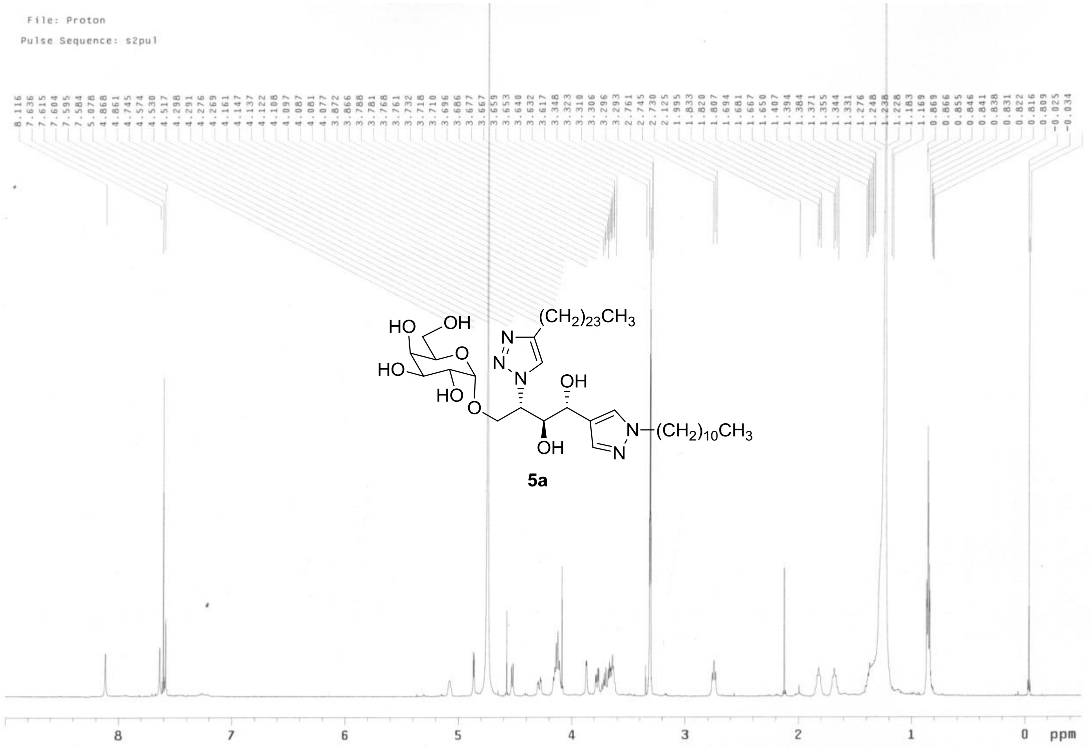
File: Proton
Pulse Sequence: s2pul
(CH2)23CH3
(CH2)10CH3
5a
ppm

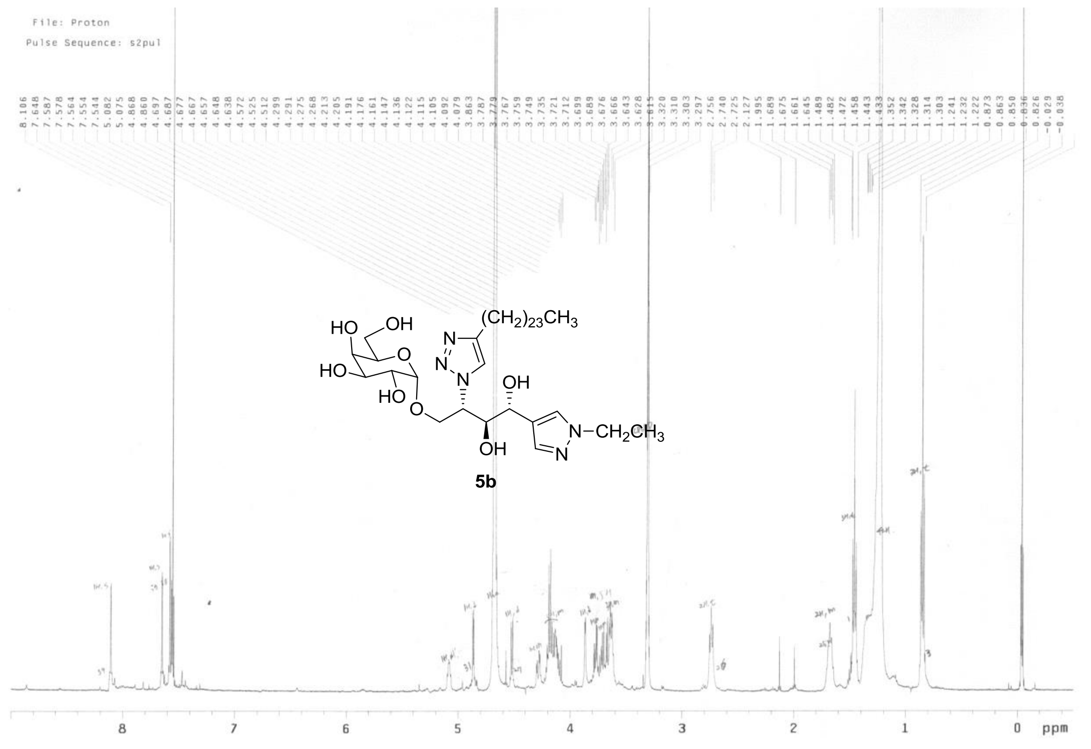
File: Proton
Pulse Sequence: s2pul
(CH2)23CH3
N-CH2CH3
5b
ppm

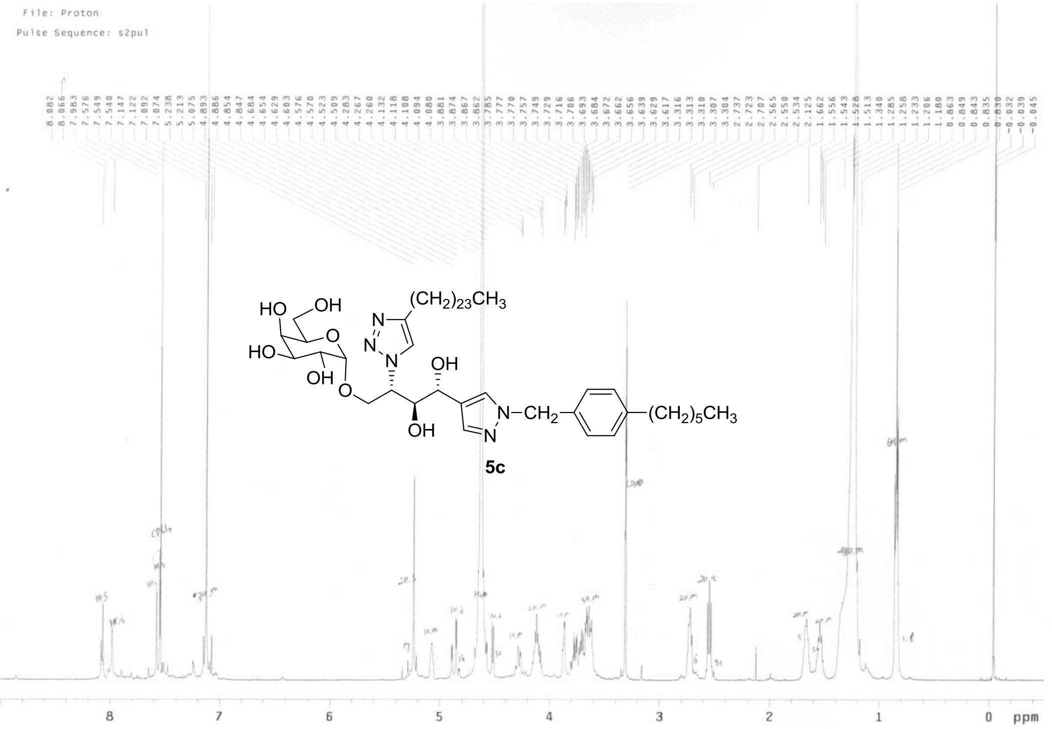
File: Proton
Pulse Sequence: s2pul
(CH2)23CH3
HO
OH
HO
OH
O
N
N
N
OH
OH
N
N
CH2
(CH2)5CH3
5c
8
7
6
5
4
3
2
1
0
ppm

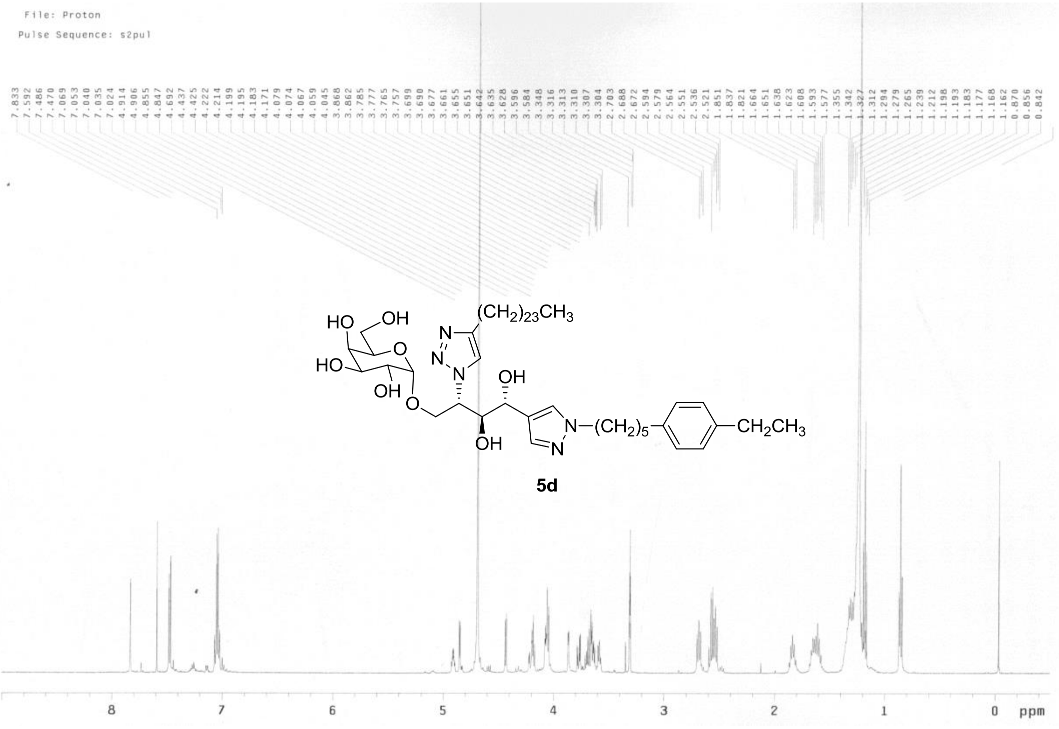
File: Proton
Pulse Sequence: s2pul
(CH2)23CH3
HO
OH
HO
OH
O
N
N
N
OH
OH
N
N
(CH2)5
CH2CH3
5d
8
7
6
5
4
3
2
1
0
ppm

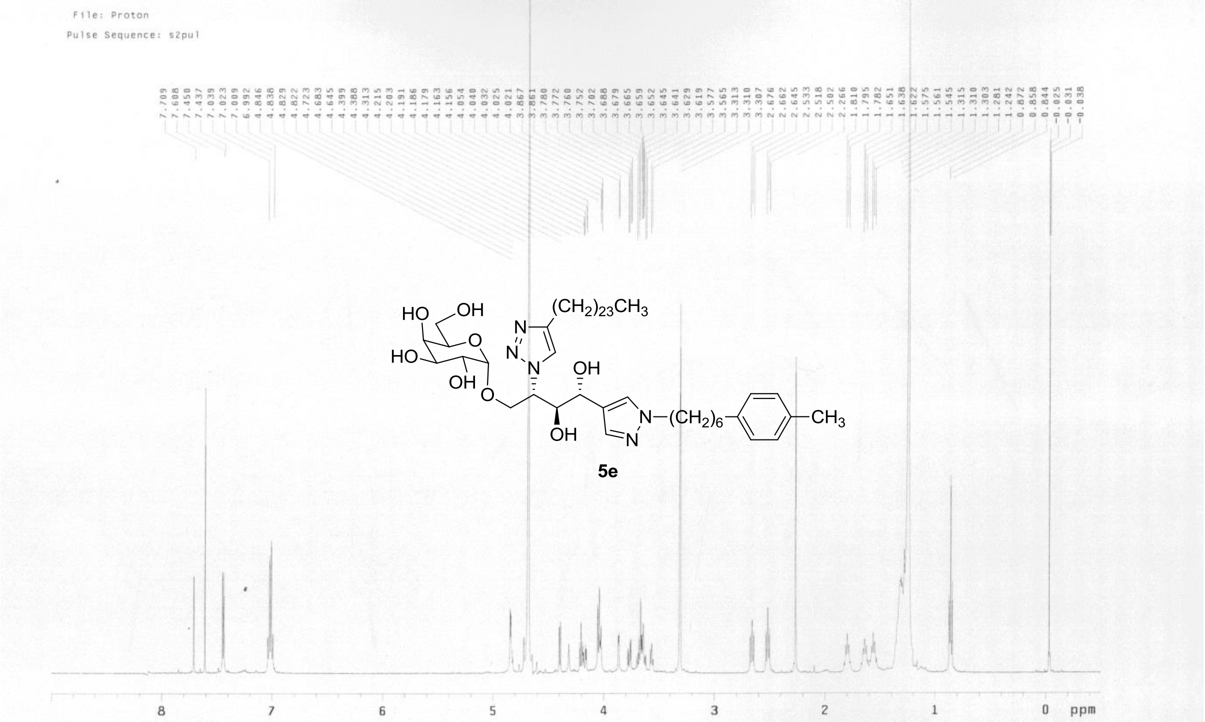
File: Proton
Pulse Sequence: s2pul
(CH2)23CH3
HO
OH
HO
OH
O
O
N
N
N
OH
OH
N
N
(CH2)6
CH3
5e
8
7
6
5
4
3
2
1
0
ppm
